# 6-*exo*-trig Michael addition-lactonizations for catalytic enantioselective chromenone synthesis†
†Electronic supplementary information (ESI) available: Experimental procedures; characterization data for novel compounds; ^1^H and ^13^C NMR spectra, HPLC traces,^12^ and X-ray crystallographic data files compounds **9** and **27** (CIF). CCDC 1510311 and 1510312. For ESI and crystallographic data in CIF or other electronic format see DOI: 10.1039/c6cc10178j



**DOI:** 10.1039/c6cc10178j

**Published:** 2017-01-19

**Authors:** Rifahath M. Neyyappadath, David B. Cordes, Alexandra M. Z. Slawin, Andrew D. Smith

**Affiliations:** a EaStCHEM, School of Chemistry , University of St Andrews , North Haugh , St Andrews , KY16 9ST , UK . Email: ads10@st-andrews.ac.uk

## Abstract

The catalytic enantioselective 6-*exo*-trig Michael addition-lactonization of enone-acid substrates to form *cis*-chromenones with high diastereo- and enantiocontrol was developed using the commercially available isothiourea tetramisole. An acidic workup proved necessary to minimize product epimerization and maximize product er, providing *cis*-chromenones in excellent yield, and with excellent diastereo- and enantioselectivity.

## Abstract 2

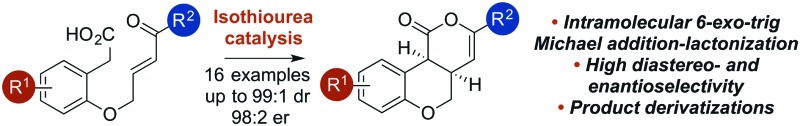
The catalytic enantioselective 6-*exo*-trig Michael addition-lactonization of enone-acid substrates to form *cis*-chromenones with high diastereo- and enantiocontrol was developed using the commercially available isothiourea tetramisole.

The development of catalytic processes that allow the preparation of valuable heterocyclic frameworks from readily prepared starting materials under mild conditions is of widespread importance.^1^ A range of enantioselective methods that fulfil these goals has been developed.^2^ In recent years the catalytic use of C(1)-ammonium enolates,^3^ particularly those using carboxylic acids as starting materials,^4^ has been popularized following the intramolecular enantioselective nucleophile-catalyzed aldol lactonization (NCAL) methodology developed by Romo for the synthesis of stereodefined β-lactones.^5^ In this area, 5-*exo*-ring closure to prepare the corresponding carbo- and heterocyclic ring systems is commonplace (Fig. 1a), and this strategy has been applied successfully for the construction of complex molecular targets.^6^ To date, only limited isolated examples of this approach for the formation of 6-membered ring systems have been developed,^7^ all of which use cinchona alkaloids as catalysts. In previous work we developed an isothiourea-catalyzed^8,9^ 5-*exo*-Michael addition-lactonization approach to 5-membered carbo- and heterocycle synthesis from enone acids (Fig. 1b).^10^ In this manuscript the application of this methodology for the preparation of 6-membered heterocycles is reported for the first time, allowing the synthesis of *cis*-chromenones^11^ in up to 99 : 1 dr and 98 : 2 er (Fig. 1c).

**Fig. 1:**
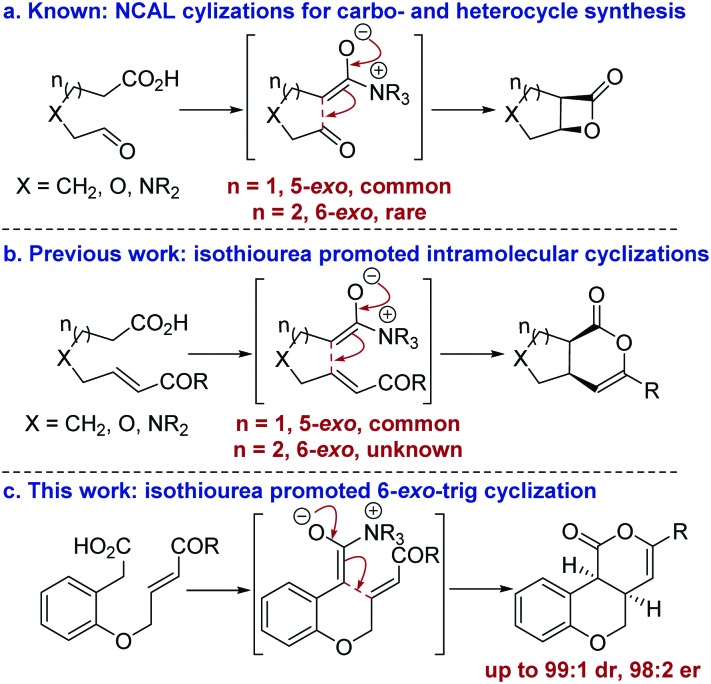
Summary of ammonium enolate promoted intramolecular catalytic enantioselective carbo- and heterocycle formation.

Initial model studies probed the cyclization of enone acid **1** to chromenone **2**, with **1** readily prepared in three steps from 2-hydroxyphenylacetic acid.^12^ Treatment of **1** with pivaloyl chloride and i-Pr_2_NEt gave the corresponding mixed anhydride *in situ*, which was subsequently treated with isothiourea catalysts **3** to **6** and evaluated for the proposed cyclization (Table 1, entries 1–4). Achiral DHPB^13^ gave the desired *cis*-chromenone **2** in 85% yield and >99 : 1 dr. Screening of a small range of chiral isothioureas **4–6** indicated the use of tetramisole **4** and its benzannulated counterpart, BTM **5**, showed promising enantioselectivity (∼87 : 13 er, entries 2 and 3). Subsequent optimization through variation of solvent, temperature and base^12^ showed that performing the reaction at 0 °C in CHCl_3_ with excess i-Pr_2_NEt (1.5 equiv. for mixed anhydride formation, followed by an additional 2.5 equiv.) gave highest observed dr and er (entries 6**–**9). Lowering the catalyst loading to 5 mol% using tetramisole **4** gave **2** in 85% yield, >99 : 1 dr and 93 : 7 er, with BTM **5** giving lower conversion and isolated product yield even after extended reaction times (entries 10 and 11).

**Table 1:** Reaction optimization

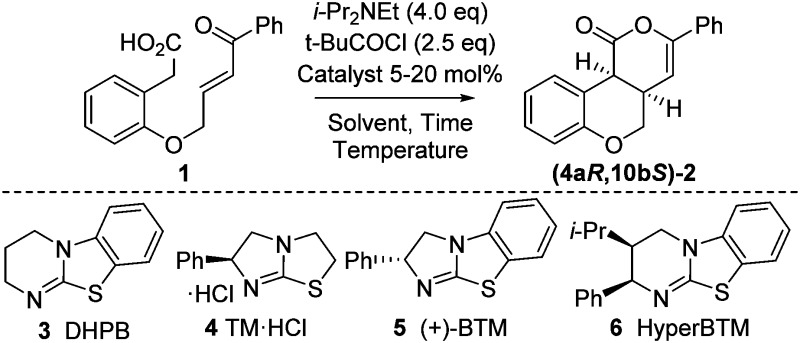						
Entry	Catalyst (mol%)	*T* (°C)	Time (h)	Yield^*a*^ (%)	dr^*b*^ (*cis *:* trans*)	er^*c*^ (4a*R*,10b*S *: 4a*S*,10b*R*)
1^*d*^	**3** (20)	rt	16	85	>99 : 1	Racemic
2^*d*^	**4** (20)	rt	16	84	>99 : 1	87 : 13
3^*d*^	**5** (20)	rt	16	84	>99 : 1	13 : 87
4^*d*^	**6** (20)	rt	16	69	>99 : 1	57 : 43
5^*d*^	—	rt	16	nil	—	—
6^*e*^	**5** (20)	rt	16	85	>99 : 1	7 : 93
7^*f*^	**5** (20)	rt	16	84	>99 : 1	7 : 93
8^*e*^	**5** (20)	0	4	87	>99 : 1	7 : 93
9^*e*^	**5** (20)	–10	16	83	>99 : 1	6 : 94
10^*e*^	**4** (5)	0	4	85	>99 : 1	93 : 7
11^*e*^	**5** (5)	0	16	65	>99 : 1	7 : 93

^*a*^Isolated yield.

^*b*^Measured by ^1^H NMR spectroscopy of crude reaction product.

^*c*^Measured by chiral HPLC (major *cis*-diastereoisomer).

^*d*^CH_2_Cl_2_ (0.1 M).

^*e*^CHCl_3_ (0.1 M).

^*f*^CHCl_3_ (0.05 M).

Further investigation monitored product dr and er with reaction conversion and time (Table 2). These studies indicated the dr of the product remained constant (92 : 8 dr *cis* : *trans*) up to full conversion, but increased to 99 : 1 upon extended reaction times. Furthermore, the er of the major *cis*-product decreased from 99 : 1 er (up to full conversion) to 93 : 7 er with time.^12^ These observations are consistent with base catalyzed-epimerization of the minor *trans*-diastereoisomer (4a*S*,10b*S*)-**7** to *ent-cis*-(4a*S*,10b*R*) **2**, resulting in increased product dr but lower product er. Consistent with this epimerization process, treatment of an 80 : 20 mixture of *trans*-**7** : *cis*-**2** with i-Pr_2_NEt gave *cis*-**2** in >99 : 1 dr.^14^

**Table 2:** Epimerization studies

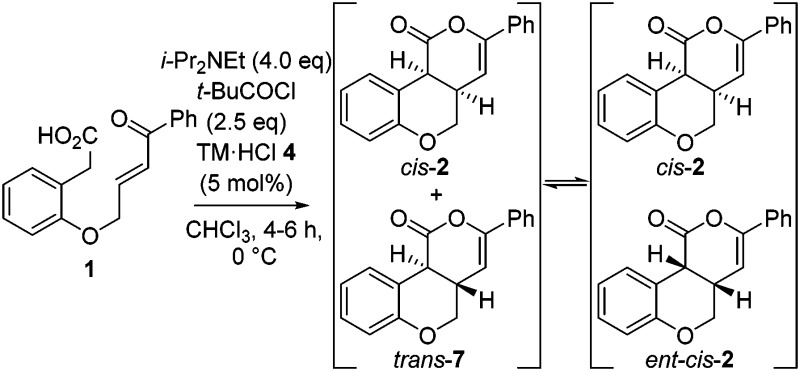				
Entry	Conversion^*a*^	Time (h)	dr^*a*^ (*cis *:* trans*)	er^*b*^ (4a*R*,10b*S *:* *4a*S*,10b*R*)
1	31%	0.5	92* *:* *8	99* *:* *1
2	63%	1.5	92* *:* *8	98.5* *:* *1.5
3	Quant	4.5	92* *:* *8	98* *:* *2
4	Quant	16	99* *:* *1	93* *:* *7

^*a*^Measured by ^1^H NMR spectroscopy of crude reaction product.

^*b*^Measured by chiral HPLC (major *cis*-diastereoisomer).

To circumvent product epimerization and maximize product er incorporation of an acidic aqueous work-up protocol was essential. For example, carrying the reaction out at 0 °C, followed by work up with H_2_O at rt gave **2** in 85% yield, >99 : 1 dr and 93 : 7 er. However, work up with 0.1 M HCl at 0 °C gave **2** in 93 : 7 dr, with purification giving **2** as a single diastereoisomer in 70% yield and 98 : 2 er (Scheme 1).

**Scheme 1:**
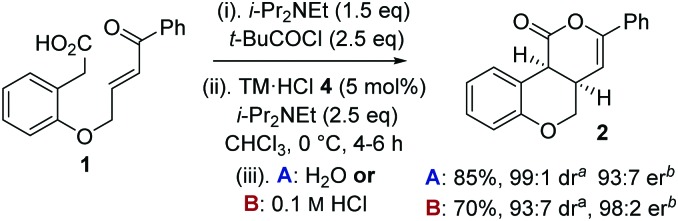
Optimized procedure. ^*a *^Measured by ^1^H NMR spectroscopy of crude reaction product. ^*b *^Measured by chiral HPLC (major *cis*-diastereoisomer).

With an optimized protocol established, the generality of this process was investigated (Table 3). The tolerance of this methodology to variation within the enone portion was initially probed, with all starting materials prepared from the corresponding 2-hydroxy arylacetic acid through *O*-allylation, ozonolysis and Wittig olefination.^12^ Using the 0.1 M HCl work up protocol generally high product er and dr was observed.^15^ Notable trends within this series showed that incorporation of halogen (4-FC_6_H_4_ **8** and 4-ClC_6_H_4_ **9**) substituents, as well as electron-donating (4-MeOC_6_H_4_ **10** and 4-MeC_6_H_4_ **11**) and 2-naphthyl substituents **15** gave the desired *cis*-chromenones in excellent enantioselectivity (97 : 3 to 98 : 2 er). Incorporation of electron-withdrawing 4-CF_3_C_6_H_4_or 3,5-(CF_3_)_2_C_6_H_3_ substituents was also tolerated, giving **12** with marginally reduced enantioselectivity and **13** in moderate 37% yield. Incorporation of an aliphatic enone led to decreased reactivity, requiring high catalyst loadings (20 mol%) to promote this transformation (29% isolated yield at 46% conversion), giving **14** as a single diastereoisomer in moderate 71 : 29 er.^16^ The relative and absolute configuration within **9** was unambiguously confirmed by X-ray crystal structure analysis,^17^ with the absolute configuration of all other products assigned by analogy.

**Table 3:** Reaction scope: variation of enone component

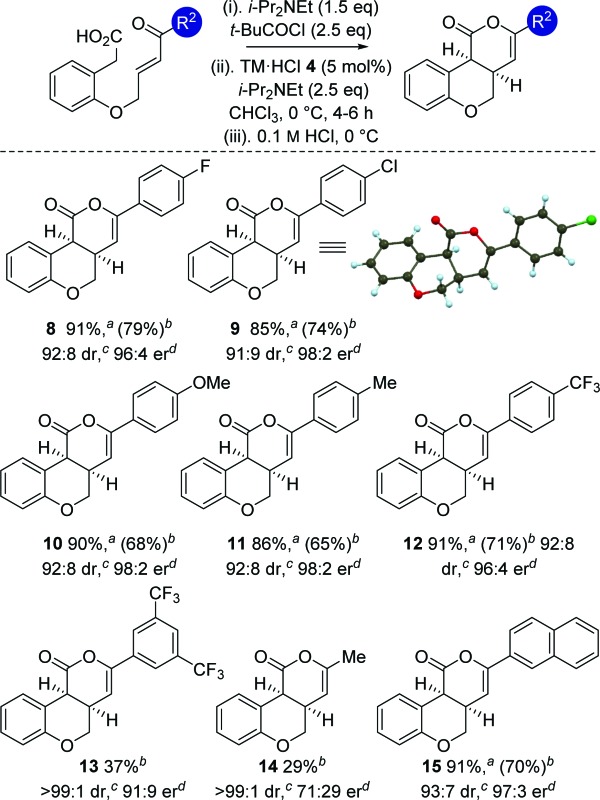

^*a*^Combined isolated yield of diastereoisomers.

^*b*^Isolated yield of major *cis*-diastereoisomer (>99 : 1 dr).

^*c*^Measured by ^1^H NMR spectroscopy of crude reaction product.

^*d*^Measured by chiral HPLC (major *cis*-diastereoisomer).

The generality of this methodology was further investigated using different substituents within the aromatic tether (Table 4). Variation of the aromatic tether, incorporating substitution with electron-donating (5-Me, 4-OMe), halogen (4-F) and naphthyl groups gave *cis*-chromenones **16–22** with excellent enantioselectivity (95 : 5 to 98 : 2 er). Notably, incorporation of 4-OMe substituents on the aromatic tether (to give **17** and **21**) showed decreased reactivity, with the reaction taking extended reaction times (12–14 h) to reach >98% conversion, but still proceeded with excellent enantioselectivity.

**Table 4:** Reaction scope: variation of aromatic tether

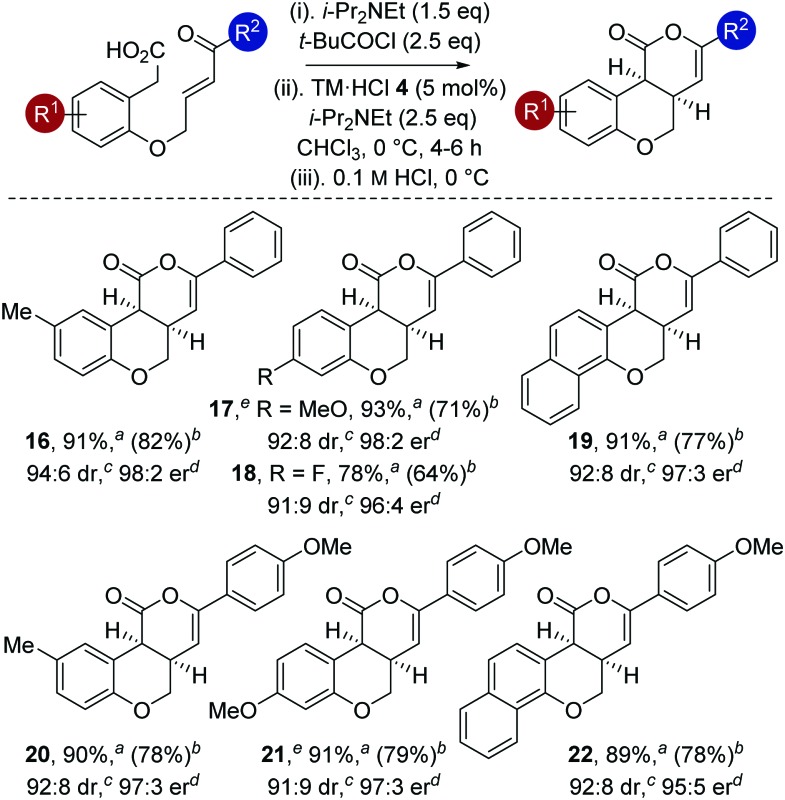

^*a*^Combined isolated yield of diastereoisomers.

^*b*^Isolated yield of major diastereoisomer (>99 : 1 dr).

^*c*^Measured by ^1^H NMR spectroscopy of crude reaction product.

^*d*^Measured by chiral HPLC (major *cis*-diastereoisomer).

^*e*^12–14 h reaction time.

Reaction scale-up and subsequent product derivatization was investigated. On a one-gram scale, complete conversion of **1** to **2** was observed using only 2.5 mol% catalyst within 6 h to give **2** in 86% isolated yield as a single diastereoisomer and 98 : 2 er.

The synthetic utility of the products was then explored through a range of derivatizations (Scheme 2). Ring-opening of **2** with either methanol, morpholine or benzylamine gave the corresponding *cis*-dihydrobenzopyrans **23–25** in excellent yield, dr and er. Treatment of *cis*-chromenone **2** with Pd/C and H_2_ (1 atm) led to hydrogenation and hydrogenolysis, giving acid **26** in excellent yield. Alternatively, treatment of a recrystallized sample of **2** (>99 : 1 er) with *m*-CPBA, followed by *p*-TSA, gave the 5-membered lactone^18^ **27** in excellent yield and stereocontrol [96 : 4 dr, >99 : 1 er]. Recrystallization from 10% EtOAc in hexane gave **27** in >99 : 1 dr, >99 : 1 er and 82% yield. The relative and absolute configuration of **27** was confirmed by single crystal X-ray structure analysis.^17^

**Scheme 2:**
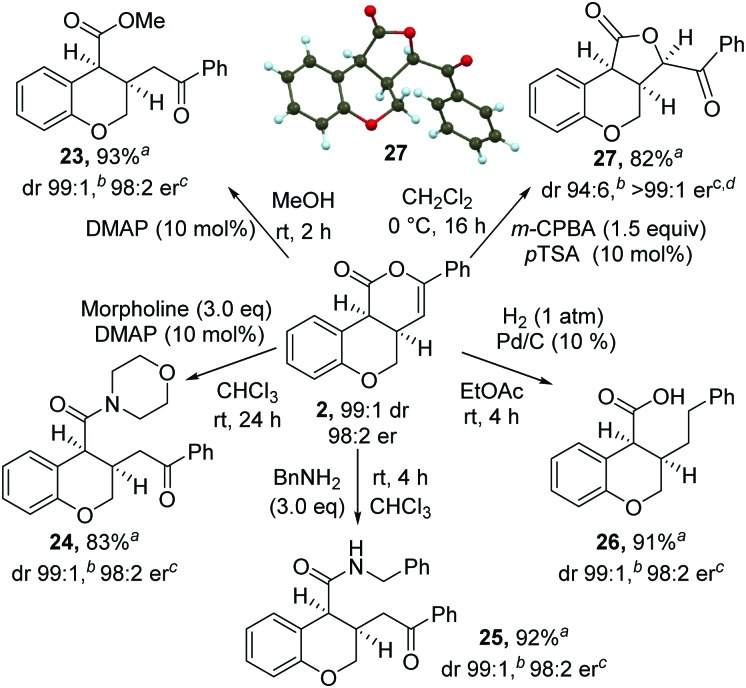
Product derivatization. ^*a *^Isolated yield of major diastereoisomer (>99 : 1 dr). ^*b *^Measured by ^1^H NMR spectroscopy of crude reaction product. ^*c *^Measured by chiral HPLC (major *cis*-diastereoisomer). ^*d*^ Starting material **2** was >99 : 1 dr and 99 : 1 er.

The mechanism of the isothiourea-catalyzed reaction, shown for the cyclization of enone-acid **1** to **2**, is postulated to proceed *via in situ* formation of mixed anhydride **28** (Scheme 3). Nucleophilic addition of isothiourea **4** to **28** gives acyl isothiouronium ion intermediate **29**, with deprotonation generating (*Z*)-ammonium enolate **30**. Subsequent intramolecular 6-*exo*-trig Michael addition to the tethered enone generates intermediate **31**, with lactonization giving *cis*-chromenone **2** and regenerating the catalyst **4**. A simplistic model to rationalize the observed diastereo- and enantiocontrol utilizes a stabilising n_0_ to σ_C–S_* interaction^19^ between the enolate oxygen and the sulfur of the isothiouronium ion to restrict the conformation of the (*Z*)-enolate,^20^ forcing the stereodirecting phenyl substituent to adopt a pseudoaxial orientation to minimize 1,2-strain. Subsequent 6-*exo*-trig Michael addition occurs *anti*- to this stereodirecting group as represented by pre-transition state assembly **32**, with the two-prostereogenic centres along the developing C–C bond adopting a staggered array to minimize non-bonding interactions.

**Scheme 3:**
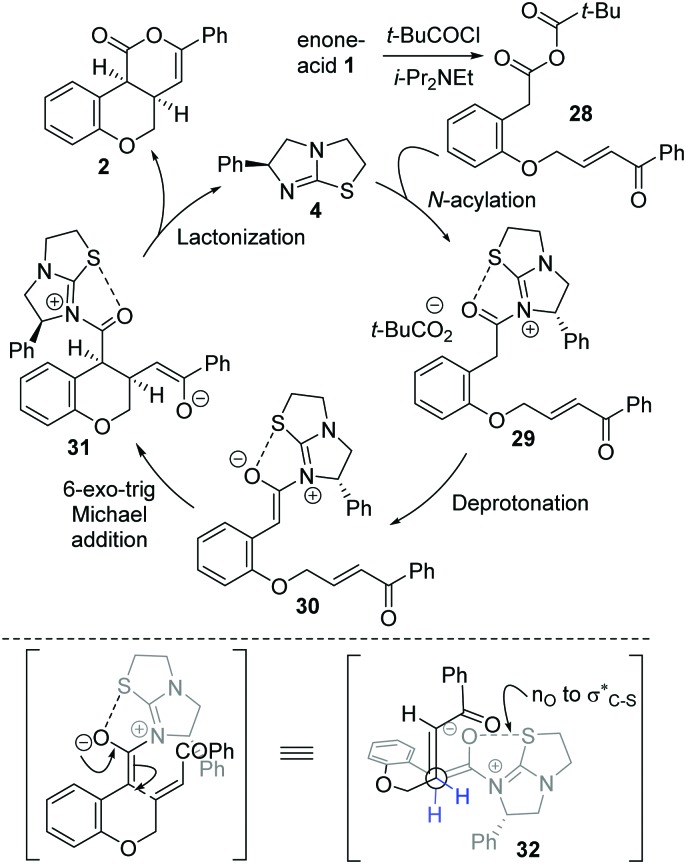
Proposed mechanism and stereochemical rationale.

In conclusion, the catalytic enantioselective synthesis of *cis*-chromenones has been achieved using commercially available tetramisole as a catalyst. This method provides a range of *cis*-chromenone derivatives in high yield with excellent diastereo- and enantiocontrol (up to 99 : 1 dr and 98 : 2 er). On-going studies in this laboratory are focused on further applications of Lewis base organocatalysts in enantioselective catalysis.

We thank the EPSRC Centre for Doctoral Training in Critical Resource Catalysis (CRITICAT, grant code EP/L016419/1, RMNP) for funding. The European Research Council under the European Union's Seventh Framework Programme (FP7/2007-2013) ERC Grant Agreement No. 279850 is also acknowledged. ADS thanks the Royal Society for a Wolfson Research Merit Award. We also thank the EPSRC UK National Mass Spectrometry Facility at Swansea University.
