# Supplementary material for: 6-exo-trig Michael addition-lactonizations for catalytic enantioselective chromenone synthesis
Source: Chem Commun (Camb). 2017 Jan 19;53(17):2555–8. doi: 10.1039/c6cc10178j (PMC5358503; doi:10.1039/c6cc10178j)

Electronic Supplementary Material (ESI) for ChemComm.
This journal is © The Royal Society of Chemistry 2017

6-*exo*-trig Michael Addition-Lactonisations for catalytic Enantioselective Chromenone Synthesis

Rifahath M. Neyyappadath, David B. Cordes, Alexandra M. Z. Slawin and Andrew D. Smith*

EaStCHEM School of Chemistry, University of St Andrews, North Haugh, St Andrews, KY16 9ST, U.K.

e-mail: ads10@st-andrews.ac.uk

Supporting Information

General Information.. S2

General Procedures.. S4

Synthesis of enone-acids, Scheme S1.. S5

Preparation of 2-Hydroxy phenyl acetic acids.. S5

Preparation of a 2-(2-(Allyloxy)phenyl)acetic acids.................................. S9

Preparation of phosphoranes.. S13

Preparation of enone-acids... S15

Optimization of Michael Addition-Lactonization....................................... S25

Isothiourea-Catalyzed Michael Addition-Lactonization............................ S29

Derivatisations.. S42

References... S46

^{1}H and ^{13}C{^{1}H}NMR Spectra & Chiral HPLC Analysis.......................... S47

General Information

All anhydrous reactions were carried out in glassware that was flame dried under vacuum. Anhydrous $CHCl_3$ was purchased from Sigma Aldrich. All other solvents were used without further purification. Pivaloyl chloride was distilled prior to use and stored in a desiccator. *i*-Pr_2NEt was distilled from KOH prior to use. All other reagents and solvents were used as received without further purification.

Room temperature (r.t) refers to 20-25 °C. Temperatures of 0 °C and −78 °C were obtained using ice/water and CO_2(s)/acetone baths respectively. Reaction temperatures over 20-25 °C were obtained using a heating mantle equipped with a contact thermometer. *In vacuo* refers to the use of a Büchi Rotavapor R-210 with a vacuum controller V-850, a IKA rotary evaporator IKA RV 10 basic with a vacuum controller Vacubrand CVC 3000 or a Heidolph Laborota 4001 rotary evaporator with a vacuum controller.

Thin layer chromatography (TLC) was carried out using aluminium plates coated with silica (Kieselgel 60 F254 silica) and visualisation was achieved using ultraviolet light (254 nm). Flash chromatography used Kieselgel 60 silica in the solvent system stated. Purifications using an automated Biotage® IsoleraTM 4 were performed using the solvent systems and column volumes (CV) stated.

Melting points were recorded on Electrothermal 100 apparatus and are uncorrected. Optical rotations were measured on a Perkin Elmer Precisely/Model-341 polarimeter operating at the sodium D line with a 100 mm path cell at r.t.

Infrared spectra (ν_{max}) were recorded on a Shimadzu IRAffinity-1 Fourier Transform ATIR spectrometer as thin films using a Pike MIRacle ATR accessory. Only the characteristic peaks are quoted.

HPLC analyses were obtained on a Shimadzu HPLC consisting of a DGU-20A5 degasser, LC-20AT liquid chromatography, SIL-20AHT autosampler, CMB-20A communications bus module, SPD-M20A diode array detector and a CTO-20A column oven which allowed the

temperature to be set from 25-40 °C. Separation was achieved using DAICEL CHIRALCEL OD-H column or DAICEL CHIRALPAK AD-H and IB columns. All chiral HPLC traces were compared to the authentic racemic spectrum.

^{1}H, ^{13}C{1H}, ^{19}F{1H} NMR spectrum were obtained on either a Bruker Avance II 400 (400 MHz ^{1}H, 101 MHz ^{13}C, 377 MHz ^{19}F) or a Bruker Avance 500 (500 MHz ^{1}H, 126 MHz ^{13}C, 471 Hz ^{19}F, 202 ^{31}P) spectrometer at r.t in the deuterated solvent stated. All chemical shifts are in ppm relative to residual solvent peak. All chemical shifts are quoted in parts per million (ppm) relative to the residual solvent as the internal standard. All coupling constants, *J*, are quoted in Hz. Multiplicities are indicated by: s (singlet), d (doublet), t (triplet), q (quartet), m (multiplet), dd (doublet of doublets), dt (doublet of triplets), dq (doublet of quartets). The abbreviation Ar is used to denote aromatic, Ph to denote phenyl, br to denote broad and *app* to denote apparent.

High resolution mass spectrometry (*m/z*) data was acquired at the EPSRC UK National Mass Spectrometry Facility at Swansea University. At the EPSRC National Mass Spectrometry Service Centre, low resolution NSI MS was carried out on a Micromass Quattro II spectrometer and high resolution NSI MS on a Thermofisher LTQ Orbitrap XL spectrometer.

General Procedures

<u>General Procedure A:</u> A solution of a 2-bromo-1-phenylethanone derivative (10 mmol, 1.0 equiv) and triphenylphosphine (1.0 equiv) was heated at reflux in anhydrous THF (30 mL) for 4 h. The reaction mixture was cooled to r.t and the the phosphonium salt was filtered and washed with Et_2O (2 × 20 mL). The phosponium salt was dissolved in H_2O:CH_2Cl_2 1.5:1 and 2 M aqueous NaOH (3.0 equiv) was added. The mixture was stirred overnight at r.t. and then extracted with CH_2Cl_2 (3 × 20 mL), washed with brine, dried over anhydrous $MgSO_4$ and concentrated *in vacuo* to afford the product as a solid.

<u>General Procedure B (ozonolysis followed by Wittig reaction):</u> A stream of O_3 in O_2 was bubbled through a solution of an allyl acid derivative (1.00 g, 1.0 equiv) in CH_2Cl_2 (20 mmol/L) at −78 °C. When the color changed to pale grey/light blue (10-15 min), the cooling bath was removed and dimethyl sulfide (2.0 equiv) was added and the reaction mixture stirred at r.t. for 30 min. The reaction mixture was concentrated *in vacuo* and the resulting oil dissolved in $CHCl_3$ (50 mL). A phosphorane derivative (1.1 equiv) was added and heated at reflux overnight under nitrogen. The reaction mixture was concentrated *in vacuo*, and the residue purified by column chromatography using Biotage® Isolera™ 4 with 0-20% EtOAc:hexane as eluent.

General Procedure C: In a flame-dried round bottom flask, the enone-acid (0.1 mmol, 1.0 equiv) was dissolved in anhydrous $CHCl_3$ (1.0 mL) at r.t. $(i\text{-Pr})_2NEt$ (1.5 equiv) was added followed by dropwise addition of pivaloyl chloride (2.0 equiv) over 1-2 min. The reaction was stirred for 1 h then cooled to 0 °C. The catalyst (5 - 20 mol%) and $(i\text{-Pr})_2NEt$ (2.5 equiv) were added, and the reaction stirred for 3-5 h at 0 °C. Upon completion, the reaction was washed with a 0 °C solution of 0.1 M aqueous HCl (2 × 12 mL $mmol^{-1}$). The aqueous layer was extracted with $CHCl_3$ (3 × 2 mL), and the combined organic fractions dried ($MgSO_4$), filtered and evaporated under reduced pressure at 30–35 °C. The residue was purified by Biotage® Isolera™ 4 [SNAP Ultra 25 g, 75 mL min^{-1}, hexane:EtOAc (100:0 2 CV, 100:0 to 92:8 40 CV)] to afford a major fraction of pure *cis*-product and a minor fraction of a mixture of *cis* and *trans* products, both as solids.

Synthesis of enone acids, Scheme S1.

Starting materials **S1 – S5** are commercially available and purchased from commercial suppliers Sigma Aldrich and Alfa Aesar.

S6- S9 were synthesized from 2-hydroxy acetophenone derivatives **S1-S4** *via* Wilgerodt-kindler reaction.[1] 2-(1-(Allyloxy)naphthalen-2-yl)acetic acid **S15** was prepared by allylation of corresponding ester **S10** followed by hydrolysis.

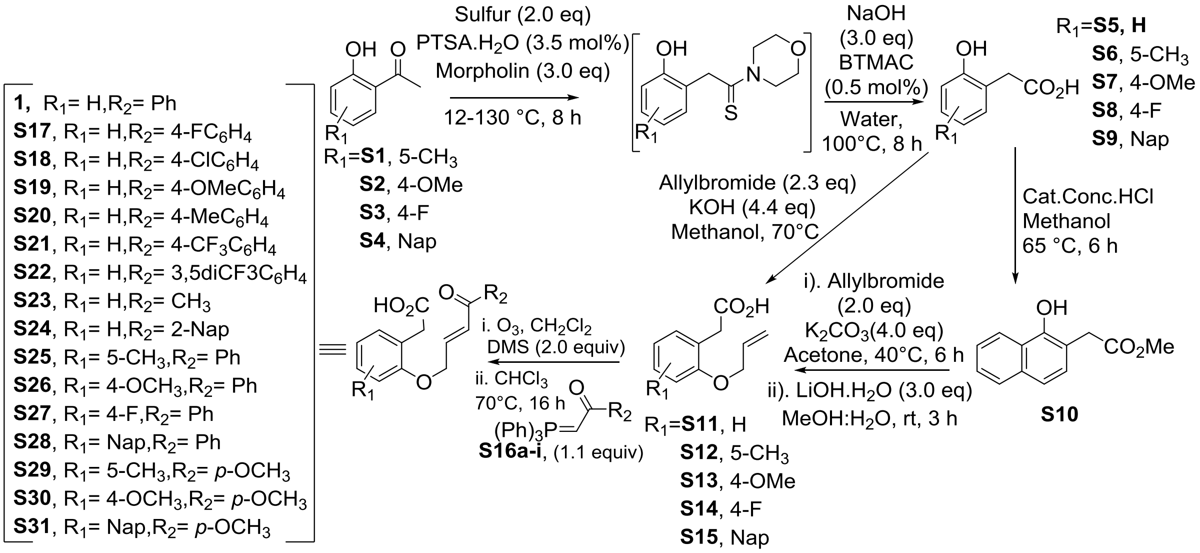

Scheme S1. Synthesis of enone acids.

Preparation of 2-Hydroxy phenyl acetic acids

2-(2-Hydroxy-5-methylphenyl) acetic acid (S6)

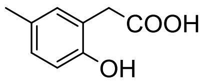

A mixture of 2-hydroxy-5-methylacetophenone (3.0g, 20 mmol, 1.0 equiv), sulfur (1.28g, 40 mmol, 2.0 equiv), morpholine (6 mL, 60 mmol, 3.0 equiv) and *p*-toluenesulfonic acid (0.12 g, 0.7 mmol, 3.5 mol%) were heated at reflux with stirring at 120–130 °C for 8 h. Upon completion, the reaction was cooled to 50–60 °C and 20% NaOH (28 mL) was added, followed by benzyltrimethylammonium chloride (BTMAC) (220 mg, 0.1 mmol, 0.5 mol%), and the reaction heated at 100 °C for 8 h. The reaction was cooled and filtered, and the filtrate was acidified to pH 6 with 6 M HCl and filtered. The filtrate was further acidified to pH 2. The solution was extracted with EtOAc (3 × 150 mL) and the combined organic layers washed with water (100 mL), brine (100 mL), and dried over anhydrous $MgSO_4$. The residue obtained after

evaporation of the solvent was purified by column chromatography over silica gel using EtOAc:hexane (10:90) as eluent to give the product as a colourless solid. (2.2 g, 66%). mp: 124-126 °C (lit.[2] mp: 128-130 °C); ^{1}H NMR (500 MHz, $CDCl_3$) δ_H: 2.26 (3H, s, C*H$_3$*), 3.66 (C*H$_2$*), 6.79 (1H, d, *J* 8.1, ArC(3)*H*), 6.94 (1H, d, *J* 2.1, ArC(6)*H*), 6.99 (1H, dd, *J* 8.2, 2.2, ArC(4)*H*); ^{13}C{^{1}H} NMR (126 MHz, $CDCl_3$) δ_C: 20.5 (*C*H$_3$), 37.0 (*C*H$_2$), 117.1 (Ar*C*(3)H), 120 (Ar*C*(5)), 129.9 (Ar*C*(4)H), 130.7 (Ar*C*(1)), 131.8 (Ar*C*(6)H), 152.2 (Ar*C*(2)), 178.5 (C=O). Data in accordance with literature.[2]

2-(2-Hydroxy-4-methoxyphenyl) acetic acid (S7)

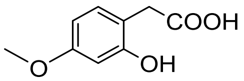

A mixture of 2-hydroxy-4-methoxyacetophenone (5.0g, 30 mmol, 1.0 equiv), sulfur (1.91g, 60 mmol, 2.0 equiv), morpholine (9 mL, 90 mmol, 3.0 equiv) and *p*-toluene sulfonic acid (0.18 g, 1.05 mmol, 3.5 mol%) were heated at reflux with stirring at 120–130 °C for 8 h. Upon completion, the reaction was cooled to 50–60 °C and 20% NaOH (43ml) was added, followed by benzyltrimethyl ammonium chloride (BTMAC) (330 mg, 0.15 mmol, 0.5 mol%) and the reaction heated at 100 °C for 8 h. The reaction was cooled and filtered, and the filtrate was acidified to pH 6 with 6 M HCl and filtered. The filtrate was further acidified to pH 2. The solution was extracted with EtOAc (3 × 150 mL) and the combined organic layers washed with water (100 mL), brine (100 mL), and dried over anhydrous $MgSO_4$. The residue obtained after evaporation of the solvent was purified by column chromatography over silica gel using EtOAc:hexane (10:90) as eluent to give the product as light yellow solid (3.5 g, 64%). mp: 127-129 °C (lit.[3] mp: 130-132 °C); ^{1}H NMR (500 MHz, $CDCl_3$) δ_H: 3.37 (2H, s, C*H*$_2$), 3.67 (3H, s, OC*H*$_3$), 6.32 (1H, dd, *J* 8.3, 2.5, ArC(5)*H*), 6.36 (1H, d, *J* 2.5, ArC(3)*H*), 6.97 (1H, d, *J* 8.3, ArC(6)*H*), 9.44 (1H, s, O*H*), 12.00 (1H, s, COO*H*); ^{13}C{^{1}H} NMR (126 MHz, $CDCl_3$) δ_C: 34.7 (*C*H$_2$), 54.9 (O*C*H$_3$), 101.0 (Ar*C*(3)H), 104.0 (Ar*C*(5)H), 114.3 (Ar*C*(1)), 131.4 (Ar*C*(6)H), 156.2 (Ar*C*(4)), 159.1 (Ar*C*(2)), 173.1 (*C*=O); m/z (NSI) $C_9H_9O_4$ ([M−H]$^-$, 100%) found 181.0510, requires 181.0506 (+2.2 ppm). Data in accordance with literature.[3]

2-(4-Fluoro-2-hydroxyphenyl)acetic acid (S8)

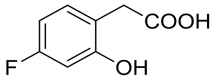

A mixture of 1-(4-fluoro-2-hydroxyphenyl)ethan-1-one (4.6g, 30 mmol, 1.0 equiv), sulfur (1.91g, 60 mmol, 2.0 equiv), morpholine (9 mL, 90 mmol, 3.0 equiv) and *p*-toluene sulfonic acid (0.18g, 1.05 mmol, 3.5 mol%) were heated at reflux with stirring at 125-135 °C. Upon completion, the reaction was cooled to r.t and 20% NaOH (43 ml) was added, followed by benzyltrimethyl ammonium chloride (BTMAC) (0.33 g, 0.15 mmol, 0.5 mol%) and the reaction heated at 100 °C for 8 h. The reaction was cooled and filtered, and the filtrate was acidified to pH 6 with 6 M HCl and filtered. The filtrate was further acidified to pH 2. The solution was extracted with EtOAc (3 × 150 mL) and the combined organic layer washed with water (100 mL), brine (100 mL), and dried over anhydrous $MgSO_4$. The residue obtained after evaporation of the solvent was purified by column chromatography over silica gel using EtOAc:hexane (10:90) as eluent to give the product as a pale yellow solid (0.83 g, 16%). mp: 139-141 °C; IR ν_{max} (film): 3340 (O-H), 3207 (COO-H), 1697 (C=O); ^{1}H NMR (400 MHz, DMSO-d$_6$) δ_H: 3.43 (2H, s, C*H*$_2$), 6.50-6.61 (2H, m, ArC(3,5)*H*), 7.06-7.14 (1H, m, ArC(6)*H*), 9.96 (1H, s, ArO*H*), 12.14 (1H, s, COO*H*); ^{13}C{^{1}H} NMR (101 MHz, CDCl$_3$) δ_C: 34.7 (*C*H$_2$), 101.9 (d, $^2J_{CF}$ 23.7, Ar*C*(3)H), 105.0 (d, $^2J_{CF}$ 21.1, Ar*C*(5)H), 118.4 (bs, Ar*C*(1)), 132.0 (d, $^3J_{CF}$ 10.3, Ar*C*(6)H), 156.7 (d, $^3J_{CF}$ 11.2, Ar*C*(2)), 161.6 (d, $^1J_{CF}$ 242.1, Ar*C*(4)F), 172.7 (*C*=O); ^{19}F NMR (376 MHz, CDCl$_3$) δ_F: −114.7; m/z (NSI) $C_8H_6FO_3$ ([M−H]$^-$, 100%) found 169.0307, requires 169.0306 (+0.3 ppm).

2-(1-Hydroxynaphthalen-2-yl)acetic acid (S9)

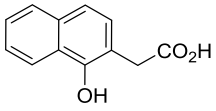

A mixture of 1-(1-hydroxynaphthalen-2-yl)ethan-1-one (9.31g, 50 mmol, 1.0 equiv), sulfur (3.2g, 100 mmol, 2.0 equiv), morpholine (15 mL, 150 mmol, 3.0 equiv) and *p*-toluene sulfonic acid (0.3 g, 1.75 mmol, 3.5 mol%) were heated at reflux with stirring at 120–130 °C for 8 h. Upon completion, the reaction was cooled to 50–60 °C and 20% NaOH (71.6 ml) was added, followed by benzyltrimethyl ammonium chloride (BTMAC) (0.57 g, 0.25 mmol, 0.5 mol%) and the reaction heated at 100 °C for 8 h. The reaction was cooled and filtered, and the filtrate was acidified to pH 6 with 6 M HCl and filtered. The filtrate was further acidified to pH 2. The solution was extracted with EtOAc (3 × 200 mL) and the combined organic layers washed with water (100 mL), brine (100 mL), and dried over anhydrous $MgSO_4$. The residue obtained after

evaporation of the solvent was purified by column chromatography over silica gel using EtOAc:hexane (10:90) as eluent to give the product as as light yellow solid. (7.5g, 75%). mp: 141-143 °C; ^{1}H NMR (400 MHz, DMSO-d$_6$) δ_H: 3.74 (2H, s, C*H*$_2$), 7.28 (1H, d, *J* 8.3, ArC(4)*H*), 7.36 (1H, d, *J* 8.3, ArC(3)*H*), 7.40-7.50 (2H, m, ArC(5,7)*H*), 7.76-7.86 (1H, m, ArC(6)*H*), 8.16-8.25 (1H, m, ArC(8)*H*), 9.35 (1H, s, O-H); ^{13}C{^{1}H} NMR (101 MHz, CDCl$_3$) δ_C: 35.7 (*C*H$_2$), 116.6 (Ar*C*(2)), 119.0 (Ar*C*(3)H), 122.0 (Ar*C*(4)H), 124.8 (Ar*C*(6)H), 125.2 (Ar*C*(4a)), 125.6 (Ar*C*(7)H), 127.5 (Ar*C*(5)H), 129.4 (Ar*C*(8)H), 133.5 (Ar*C*(8a)), 150.2 (Ar*C*(1)), 173.0 (*C*=O).

Methyl 2-(1-hydroxynaphthalen-2-yl)acetate (S10)

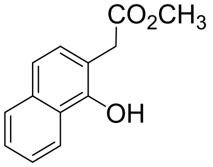

To a solution of 2-(1-hydroxynaphthalen-2-yl)acetic acid (7.0g, 34.6 mmol, 1.0 equiv) in methanol (60 mL) was added conc. HCl (0.15 mL). The solution was heated at 65 °C for 6 h. The solvent was removed by vacuum and the crude product was redissolved in dichloromethane (125 mL) which was subsequently washed with sat. aqueous $NaHCO_3$ (40 mL), water (40 mL), and brine (40 mL). The organic layer was dried over anhydrous $MgSO_4$, filtered, and concentrated to give methyl 2-(1-hydroxynaphthalen-2-yl)acetate as colourless oil (6.5 g, 87 %), and was used without further purification. ^{1}H NMR (400 MHz, CDCl$_3$) δ_H: 3.78 (3H, s, OC*H*$_3$), 3.84 (2H, s, C*H*$_2$), 7.19 (1H, d, *J* 8.4, ArC(4)*H*), 7.40 (1H, dd, *J* 8.4, 0.8, ArC(3)*H*), 7.45-7.54 (2H, m, ArC(5,7)*H*), 7.75-7.82 (1H, m, ArC(6)*H*), 8.31-8.39 (1H, m, ArC(8)*H*), 8.43 (1H, s, O-*H*); ^{13}C{^{1}H} NMR (101 MHz, CDCl$_3$) δ_C: 38.3 (Ar*C*H$_2$), 53.1 (O*C*H$_3$), 113.5 (Ar*C*(2)), 120.5 (Ar*C*(3)H), 122.4 (Ar*C*(4)H), 125.6 (Ar*C*(6)H), 126.1 (Ar*C*(4a)), 126.5 (Ar*C*(7)H), 127.4 (Ar*C*(5)H), 128.5 (Ar*C*(8)H), 134.4 (Ar*C*(8a)), 151.4 (Ar*C*(1)), 175.1 (*C*=O) ; m/z (NSI) $C_{13}H_{13}O_3$ ($[M+H]^+$, 100%) found 217.0858, requires 217.0859 (−0.6 ppm). Data in accordance with literature.[4]

Preparation of a 2-(2-(Allyloxy)phenyl)acetic acids

2-(2-(Allyloxy)phenyl)acetic acid (S11)

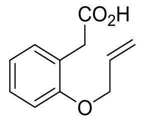

To a solution of KOH (4.1 g, 72.3 mmol, 2.22 equiv) in methanol (30 mL) was added 2-hydroxyphenyl acetate (5.0 g, 32.9 mmol, 1.0 equiv) followed by dropwise addition of allyl bromide (6.5 mL, 75.6 mmol, 2.3 equiv) with stirring. The reaction was heated at reflux for 2 h, then cooled to 50 °C. A solution of KOH (4.1 g, 72.30 mmol, 2.22 equiv) in methanol (20 mL) was added and the reaction heated at reflux for an additional 1 h. The reaction was cooled to r.t. and the methanol removed under reduced pressure. The aqueous mixture was washed with Et_2O (2 × 50 mL), and then acidified to pH 1-2 using 6.0 M aqueous HCl. The aqueous layer was extracted using Et_2O (2 × 50 mL), which was dried over anhydrous $MgSO_4$, filtered and concentrated under vacuum. The residue was purified by column chromatography over silica gel using EtOAc:hexane (10:90) as eluent to give the product as a colourless solid. (4.6 g, 73%). mp: 74-76 °C (lit.[5] 80-81 °C); ^{1}H NMR (500 MHz, $CDCl_3$) δ_H: 3.69 (2H, s, ArC*H₂*), 4.56 (2H, app dt, *J* 5.0, 1.7, OCH_2), 5.25 (1H, app dq, *J* 10.6, 1.5, CH=CH^AH^B), 5.41 (1H, app dq *J* 17.2, 1.7, CH=CH$^A H^B$), 5.96-6.06 (1H, m, C*H*=CH_2), 6.87 (1H, d, *J* 8.2, 1.0, ArC(3)*H*), 6.93 (1H, app td, *J* 7.5, 1.1, ArC(5)*H*), 7.20 (2H, dd, *J* 7.4, 1.7, ArC(4)*H*), 7.23-7.28 (1H, m, ArC(6)*H*); ^{13}C{^{1}H} NMR (126 MHz, $CDCl_3$) δ_C: 36.1 (Ar*C*H_2), 69.0 (O*C*H_2), 111.9 (*C*H=CH_2), 117.3 (CH=*C*H_2), 120.9 (Ar*C*(3)H), 122.8 (Ar*C*(1)), 128.9 (Ar*C*(5)H), 131.2 (Ar*C*(4)H), 133.1 (Ar*C*(6)H), 156.6 (Ar*C*(2)), 178.1 (*C*=O).

2-(2-(Allyloxy)-5-methylphenyl)acetic acid (S12)

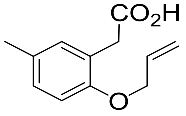

To a solution of KOH (1.5 g, 26.5 mmol, 2.22 equiv) in methanol (15 mL) was added 2-(2-hydroxy-5-methylphenyl)acetic acid (2.0 g, 12.0 mmol, 1.0 equiv) followed by dropwise addition of allyl bromide (2.4 mL, 27.7 mmol, 2.3 equiv) with stirring. The reaction was heated at reflux for 2 h, then cooled to 50 °C. A solution of KOH (1.5 g, 26.50 mmol, 2.22 equiv) in methanol (10 mL) was added and the reaction heated at reflux for an additional 1 h. The reaction

was cooled to r.t. and the methanol removed under reduced pressure. The aqueous mixture was washed with Et_2O (2 × 50 mL) and then acidified to pH 1-2 using 6.0 M aqueous HCl. The aqueous layer was extracted using Et_2O (2 × 50 mL), which was dried over anhydrous $MgSO_4$, filtered and concentrated under vacuum. The residue was purified by column chromatography over silica gel using EtOAc:hexane (10:90) as eluent to give the product as a colourless solid. (2.15g, 87%). mp: 82-84 °C; IR ν_{max} (film): 3136 (O-H), 2932 (C-H), 1683 (C=O), 1195 (C-O); ^{1}H NMR (500 MHz, $CDCl_3$) δ_H: 2.28 (3H, s, C*H₃*), 3.66 (2H, s, ArC*H₂*), 4.53 (2H, app dt, *J* 5.0, 1.7, OC*H₂*), 5.24 (1H, app dq, *J* 10.6, 1.5, CH=CH^A*H*^{B}), 5.39 (1H, app dq, *J* 17.3, 1.7, CH=C*H*^{A}H^B), 6.01 (1H, app ddt, *J* 17.3, 10.3, 5.0, C*H*=CH_2), 6.77 (1H, d, *J* 8.2, ArC(3)*H*), 6.98-7.07 (2H, m, ArC(4,6)*H*); ^{13}C{^{1}H} NMR (126 MHz, $CDCl_3$) δ_C: 20.6 (*C*H_3), 36.2 (Ar*C*H_2), 69.1 (O*C*H_2), 112.0 (CH_2=*C*H), 117.2 (CH=*C*H_2), 122.6 (Ar*C*(5)), 129.2 (Ar*C*(3)H), 130.2 (Ar*C*(1)), 131.9 (Ar*C*(4)H), 133.3 (Ar*C*(6)H), 154.5 (Ar*C*(2)), 178.0 (*C*=O); m/z (NSI) $C_{12}H_{18}NO_3$ ([M+NH_4]$^+$, 100%) found 224.1278, requires 224.1281 (−1.3 ppm).

2-(2-(Allyloxy)-4-methoxyphenyl)acetic acid (S13)

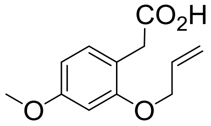

To a solution of KOH (1.91 g, 33.81 mmol, 2.22 equiv) in methanol (20 mL) added 2-(2-hydroxy-4-methoxyphenyl)acetic acid (2.8 g, 15.4 mmol, 1.0 equiv) followed by dropwise addition of allyl bromide (3.08 mL, 35.4 mmol, 2.3 equiv) with stirring. The reaction was heated at reflux for 2 h, then cooled to 50 °C. A solution of KOH (1.91 g, 33.81 mmol, 2.22 equiv) in methanol (15 mL) was added and the reaction heated at reflux for an additional 1 h. The reaction was cooled to r.t. and the methanol removed under reduced pressure. The aqueous mixture was washed with Et_2O (2 × 60 mL) and then acidified to pH 1-2 using 6.0 M aqueous HCl. The aqueous layer was extracted using Et_2O (2 × 60 mL), which was dried over anhydrous $MgSO_4$, filtered and concentrated under vacuum. The residue was purified by column chromatography over silica gel using EtOAc:hexane (10:90) as eluent to give the product as a colourless solid. (1.3 g, 38 %); mp: 84-86 °C; IR ν_{max} (film): 2902 (C-H),1695 (C=O); ^{1}H NMR (500 MHz, $CDCl_3$) δ_H: 3.62 (2H, s, ArC*H₂*), 3.79 (3H, s, OC*H₃*), 4.53 (2H, app dt, *J* 5.0, 1.7, OC*H₂*), 5.25 (1H, app dq, *J* 10.6, 1.5, CH=CH^A*H*^{B}), 5.40 (1H, app dq, *J* 17.3, 1.7, CH=CH^A*H*^{B}), 6.00 (1H, app

ddt, *J* 17.3, 10.3, 5.0, CH_2=C*H*), 6.43-6.49 (2H, m, ArC(3,5)*H*), 7.09 (1H, m, ArC(6)*H*); $^{13}C\{^1H\}$ NMR (126 MHz, $CDCl_3$) δ_C: 35.4 (Ar*C*H_2), 55.5 (O*C*H_3), 69.0 (O*C*H_2), 99.9 (CH_2=C*H*), 104.6 (Ar*C*(3)H), 115.2 (Ar*C*(1)), 117.4 (CH=*C*H_2), 131.4 (Ar*C*(5)H), 133.0 (Ar*C*(6)H), 157.4 (Ar*C*(2)), 160.4 (Ar*C*(4)), 178.1 (*C*=O); m/z (NSI) $C_{12}H_{15}O_4$ ($[M+H]^+$, 100%) found 223.0962, requires 223.0965 (−1.3 ppm).

2-(2-(Allyloxy)-4-fluorophenyl)acetic acid(S14)

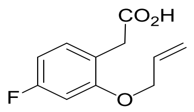

To a solution of KOH (0.54 g, 9.69 mmol, 2.22 equiv) in methanol (10 mL) was added 2-(4-fluoro-2-hydroxyphenyl)acetic acid (0.75 g, 4.4 mmol, 1.0 equiv) followed by dropwise addition of allyl bromide (0.88 mL, 10.1 mmol, 2.3 equiv) with stirring. The reaction was heated at reflux for 2 h, then cooled to 50 °C. A solution of KOH (0.54 g, 9.69 mmol, 2.22 equiv) in methanol (4 mL) was added and the reaction heated at reflux for an additional 1 h. The reaction was cooled to r.t. and the methanol removed under reduced pressure. The aqueous mixture was washed with Et_2O (2 × 25 mL), and then acidified to pH 1-2 using 6.0 M aqueous HCl. The aqueous layer was extracted using Et_2O (2 × 25 mL), which was dried over anhydrous $MgSO_4$, filtered and concentrated under vacuum. The residue was purified by column chromatography over silica gel using EtOAc:hexane (10:90) as eluent to give the product as a white colourless solid. (356 mg, 38 %); mp: 80-82 °C; IR ν_{max} (film): 2924(C-H), 1703(C=O); ^1H NMR (400 MHz, $CDCl_3$) δ_H: 3.64 (2H, s, ArC*H*$_2$), 4.53 (2H, app dt, *J* 5.0, 1.7, OC*H*$_2$), 5.27 (1H, app dq, *J* 10.6, 1.5, CH=CH^AH^B), 5.40 (1H, app dq, *J* 17.3, 1.7, CH=CH^AH^B), 5.99 (1H, app ddt, *J* 17.3, 10.6, 5.0, CH_2=C*H*), 6.55-6.69 (2H, m, ArC(3,5)*H*), 7.13 (1H, dd, *J* 8.3, 6.6, ArC(6)*H*); $^{13}C\{^1H\}$ NMR (101 MHz, $CDCl_3$) δ_C: 35.4 (Ar*C*H_2), 69.2 (O*C*H_2), 100.4 (d, $^2J_{CF}$ 24.3, Ar*C*(3)H,), 107.2 (d, $^2J_{CF}$ 21.6, Ar*C*(5)H,), 117.8 (CH=*C*H_2), 118.5 (d, $^4J_{CF}$ 3.2, Ar*C*(1)), 131.7 (d, $^3J_{CF}$ 9.9, Ar*C*(6)H), 132.5 (*C*H=CH_2), 157.6 (d, $^2J_{CF}$ 9.9, Ar*C*(2)), 163.2 (d, $^1J_{CF}$ 245.5, Ar*C*(4)F), 177.5 (*C*=O); ^{19}F NMR (376 MHz, $CDCl_3$) δ_F: −111.9; m/z (NSI) $C_{11}H_{10}FO_3$ ($[M-H]^-$, 100%) found 209.0618, requires 209.0619 (−0.7 ppm).

Methyl 2-(1-(allyloxy)naphthalen-2-yl)acetate (S15a)

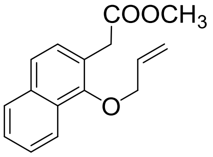

Following a procedure by Konopelski,[6] to a solution of methyl 2-(1-hydroxynaphthalen-2-yl)acetate (3.0 g, 13.9 mmol, 1.0 equiv) in acetone (30 mL) was added potassium carbonate (7.67 g, 55.5 mmol, 4.0 equiv) and allyl bromide (2.39 mL, 27.7 mmol, 2.0 equiv). The mixture was vigorously stirred for 6 h at 40 °C, cooled to r.t upon completion, and the solution filtered and then evaporated under reduced pressure. The residue was dissolved in ethyl acetate (50 mL), washed with water (25 mL) and brine (25 mL), dried over sodium sulfate and evaporated. The residue was purified by column chromatography over silica gel using EtOAc:hexane (5:95) as eluent to give the product, methyl 2-(1-(allyloxy)naphthalen-2-yl)acetate **(S15a)** as an oil (2.91g, 82%). ^{1}H NMR (400 MHz, $CDCl_3$) δ_H: 3.72 (3H, s, OCH_3), 3.89 (2H, s, ArCH_2), 4.55 (2H, app dt, *J* 5.5, 1.5, OCH_2), 5.34 (1H, app dq, *J* 10.4, 1.4, CH=CH^AH^B), 5.52 (1H, app dq, *J* 17.2, 1.6, CH=CH^AH^B), 6.21 (1H, app ddt, *J* 17.1, 10.7, 5.5, C*H*=CH$_2$), 7.38 (1H, d, *J* 8.5, ArC(4)*H*), 7.43-7.56 (2H, m, ArC(3,5)*H*), 7.62 (1H, m, ArC(6)*H*), 7.84 (1H, dd, *J* 8.0, 1.5, ArC(8)*H*), 8.05-8.14 (1H, m, ArC(7)*H*); ^{13}C{^{1}H} NMR (101 MHz, $CDCl_3$) δ_C: 35.6 (*C*H$_2$), 52.2 (O*C*H$_3$), 75.6 (O*C*H$_2$), 117.7 (Ar*C*(4a) & CH=*C*H$_2$), 122.4 (*C*H=CH$_2$), 123.2 (Ar*C*(8a)), 124.4 (Ar*C*(4)H), 126.1 (Ar*C*(3)H), 126.2 (Ar*C*(5)H), 128.2 (Ar*C*(6)H), 128.3 (Ar*C*(8)H), 133.8 (Ar*C*(7)H), 134.5 (Ar*C*(2)), 153.0 (Ar*C*(1)), 172.3 (*C*=O); m/z (NSI) $C_{16}H_{17}O_3$ ([M+H]$^+$, 100%) found 257.1171, requires 257.1172 (−0.5 ppm).

2-(1-(Allyloxy)naphthalen-2-yl)acetic acid (S15)

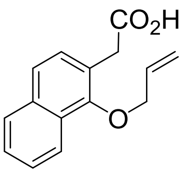

To a solution of methyl 2-(1-(allyloxy)naphthalen-2-yl)acetate (2.85 g, 11.1 mmol, 1.0 equiv) in methanol (20 mL) was added a solution of LiOH·H_2O (1.4 g, 33.4 mmol, 3.0 equiv) in water (10 mL) and stirred for 3 h at r.t. Upon completion the solvent was removed and the residue diluted

with water (50 mL). The aqueous solution was acidified to the pH 2-3 using 2 M aqueous HCl, and the product extracted using EtOAc (2 × 50 mL). The combined organic layers were dried over anhydrous $MgSO_4$ and concentrated under reduced pressure to give the product as a colourless solid (2.3 g, 86 %); mp: 81-83 °C; IR ν_{max} (film): 2889 (C-H), 1697(C=O); ^{1}H NMR (400 MHz, $CDCl_3$) δ_H: 3.91 (2H, s, CH_2), 4.56 (2H, app dt, *J* 5.5, 1.5, OCH_2), 5.33 (1H, app dq, *J* 10.4, 1.3, CH=C$H^A$$H^B$), 5.51 (1H, app dq, *J* 17.1, 1.6, CH=C$H^A$$H^B$), 6.20 (1H, app ddt, *J* 17.2, 10.7, 5.5, CH_2=C*H*), 7.38 (1H, d, *J* 8.4, ArC(4)*H*), 7.44-7.55 (2H, m, ArC(3,5)*H*), 7.58-7.66 (1H, m, ArC(6)*H*), 7.84 (1H, dd, *J* 8.0, 1.5, ArC(8)*H*), 8.05-8.12 (1H, m, ArC(7)*H*); ^{13}C{^{1}H} NMR (101 MHz, $CDCl_3$) δ_C: 35.6 (Ar*C*H_2), 75.7 (O*C*H_2), 117.9 (Ar*C*(4a) & CH=*C*H_2), 122.4 (C*H*=CH_2), 122.5 (Ar*C*(8a)), 124.5 (Ar*C*(4)H), 126.3 (Ar*C*(3,5)H), 128.2 (Ar*C*(6,8)H), 133.7 (Ar*C*(7)H), 134.7 (Ar*C*(2)), 153.1 (Ar*C*(1)), 178.0 (*C*=O); m/z (NSI) $C_{15}H_{15}O_3$ ($[M+H]^+$, 100%) found 243.1017, requires 243.1016 (+0.5 ppm).

Preparation of phosphoranes

Phosphoranes **S16a-f** were available in the lab and had been previously synthesized and characterized.[7,8,9]

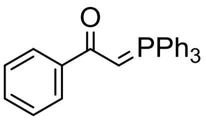

S16a

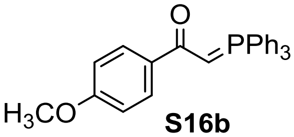

S16b

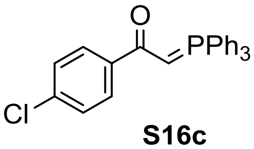

S16c

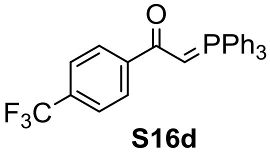

S16d

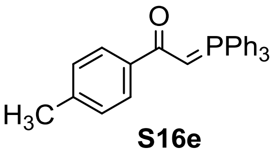

S16e

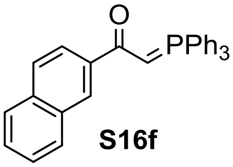

S16f

1-(Triphenylphosphoranylidene)propan-2-one (S16g)

1-(Triphenylphosphoranylidene)propan-2-one was commercially available and purchased from Aldrich.

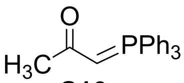

S16g

1-(4-Fluorophenyl)-2-(triphenyl-λ^5-phosphanylidene)ethan-1-one (S16h)

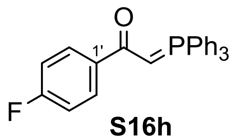

S16h

Following general procedure A, a solution of 2-bromoacetophenone (2.0 g, 9.21mmol, 1.0 equiv) and triphenylphosphine (2.4 g, 9.21mmol, 1.0 equiv) in anhydrous THF (30 mL) gave the phosphonium salt which was treated with H_2O:CH_2Cl_2 1.5:1 (18 mL:12 mL) and 2 M. aq. NaOH (14.0 mL, 27.63 mmol, 3.0 equiv) to give the product as a colourless solid (2.9g, 79 %). ^{1}H NMR (400 MHz, $CDCl_3$) δ_H : 4.36 (1H, d, *J* 23.6, Ph_3P-C*H*), 6.97-7.06 (2H, m, ArC(3',5')*H*), 7.43-7.52 (6H, m, 3×ArC(3,5)*H*), 7.53-7.61 (3H, m, 3×ArC(4)*H*), 7.66-7.76 (6H, m, 3×ArC(2,6)*H*), 7.91-7.99 (2H, m, ArC(2',6')*H*). Data in accordance with literature.[10]

1-(3,5-Bis(trifluoromethyl)phenyl)-2-(triphenyl-λ^5-phosphanylidene)ethan-1-one (S16i)

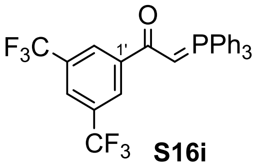

S16i

Following general procedure A, a solution of 1-(3,5-bis(trifluoromethyl)phenyl)-2-bromoethan-1-one (4.38g, 13.00 mmol, 1.0 equiv) and triphenylphosphine (3.43 g, 13.00 mmol, 1.0 equiv) in anhydrous THF (40 mL) gave the phosphonium salt which was treated with H_2O: CH_2Cl_2 1.5:1 (23 mL:15 mL) and 2 M. aq. NaOH (20 mL, 39.00 mmol, 3.0 equiv) to give the product as a colourless solid (3.88 g, 57%). mp 209-210 °C; IR ν_{max} (film): 1622 (C=O); ^{1}H NMR (500 MHz, $CDCl_3$) δ_H: 4.49 (1H, d, *J* 22.6, Ph_3P-C*H*), 7.48-7.54 (6H, m, 6×ArC(3,5)*H*), 7.58, 7.63 (3H, m, 3×ArC(4)*H*), 7.69-7.75 (6H, m, 6×ArC(2,6)*H*), 7.85 (1H, br s, ArC(4')*H*), 8.40 (2H, br s, ArC(2',6')*H*); ^{13}C NMR (125 MHz, $CDCl_3$) δ_C: 52.9 (d, $^1J_{CP}$ 111.9, *C*(2)), 122.7 (m, Ar*C*(4')H), 123.7 (q, $^1J_{CF}$ 272.8, *C*F_3), 126.2 (d, $^1J_{CP}$ 91.7, Ar*C*(1)), 127.3 (bs, Ar*C*(2',6')H) , 129.2 (d, $^3J_{CP}$ 12.4, Ar*C*(3,5)H), 131.1 (q, $^2J_{CF}$ 33.1, Ar*C*(3',5')), 132.6 (d, $^4J_{CP}$ 2.8, Ar*C*(4)H), 133.3 (d, $^2J_{CP}$ 10.3, Ar*C*(2,6)), 143.3 (d, $^3J_{CF}$ 15.5, *C*(1')), 180.8 (*C*(1)); ^{19}F NMR (376 MHz, $CDCl_3$) δ_F: −62.6; ^{31}P NMR (202 MHz, $CDCl_3$) δ_P: 17.1; m/z (NSI) $C_{28}H_{20}OF_6P$ [M+H]$^+$, found 517.1148 requires 517.1150 (−0.5 ppm).

Preparation of enone-acids

(*E*)-2-(2-((4-Oxo-4-phenylbut-2-en-1-yl)oxy)phenyl)acetic acid (1)

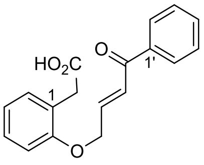

Following general procedure B: 2-(2-(Allyloxy)phenyl)acetic acid **S11** (2.35g, 12.23 mmol, 1.0 equiv) dimethyl sulfide (1.79 mL, 24.46 mmol, 2.0 equiv) in CH_2Cl_2 (612 mL), followed by 1-phenyl-2-(triphenyl-λ^5-phosphanylidene)ethan-1-one **S16a** (5.11g, 2.45 mmol, 1.1 equiv) in $CHCl_3$ (100 mL) gave the product **1** as a colourless solid (1.0g, 28%); mp: 115-117 °C; IR ν_{max} (film): 3039 (O-H), 2914 (C-H), 1705 (HOC=O), 1674 (C=O); ^{1}H NMR (500 MHz, $CDCl_3$) δ_H: 3.73 (2H, s, C*H*$_2$), 4.70 (2H, dd, *J* 3.4, 2.2, OC*H*$_2$), 6.79 (1H, dd, *J* 8.2, 1.0, ArC(3)*H*), 6.96 (1H, app td, *J* 7.4, 1.0, ArC(5)*H*), 7.13 (1H, dt, *J* 15.4, 3.4, C(3)*H*), 7.20 (1H, dd, *J* 7.5, 1.7, ArC(6)*H*), 7.23-7.29 (1H, m, ArC(4)*H*), 7.31-7.41 (3H, m, C(2)*H* & ArC(3',5')*H*), 7.43-7.52 (1H, m, ArC(4')*H*), 7.92-7.97 (2H, m, ArC(2',6')*H*); ^{13}C{^{1}H} NMR (126 MHz, $CDCl_3$) δ_C: 36.5 (Ar*C*H$_2$), 67.0 (O*C*H$_2$), 111.6 (Ar*C*(3)H), 121.4 (Ar*C*(5)H), 122.8 (Ar*C*(1)), 124.7 (Ar*C*(4)H), 128.78 (Ar*C*(6)H & C(3)H), 128.80 Ar*C*(3',5')H 129.1 (Ar*C*(6')H), 131.5 (Ar*C*(2')H), 133.2 (Ar*C*(4')H), 137.5 (Ar*C*(1')), 142.1 (*C*(2)H), 156.0 (Ar*C*(2)), 177.6 (HO*C*=O), 189.9 (Ar*C*=O); m/z (NSI) $C_{18}H_{15}O_4$ ([M−H]$^-$, 100%) found 295.0972, requires 295.0976 (−1.3 ppm).

(*E*)-2-(2-((4-(4-Fluorophenyl)-4-oxobut-2-en-1-yl)oxy)phenyl)acetic acid (S17)

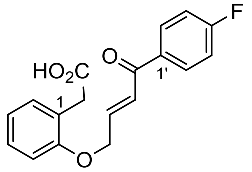

Following general procedure B: 2-(2-(Allyloxy)phenyl)acetic acid **S11** (1.25 g, 6.50 mmol, 1.0 equiv) dimethyl sulfide (0.96 mL, 13.00 mmol, 2.0 equiv) in CH_2Cl_2 (325 mL), followed by 1-(4-fluorophenyl)-2-(triphenyl-λ^5-phosphanylidene)ethan-1-one **S16h** (2.84 g, 7.15 mmol, 1.1 equiv) in $CHCl_3$(50 mL) gave the product **S17** as a colourless solid (442 mg, 22%); mp: 127-129 °C; IR ν_{max} (film): 3047 (O-H), 2914 (C-H), 1705 (HOC=O), 1674 (C=O); ^{1}H NMR (400 MHz, $CDCl_3$) δ_H : 3.72 (2H, s, C*H*$_2$), 4.68 (2H, dd, *J* 3.2, 2.2, OC*H*$_2$), 6.76 (1H, dd, *J* 8.2, 1.0, ArC(3)*H*), 6.91-7.07 (3H, m, ArC(5)*H* & ArC(3',5')*H*), 7.13 (1H, dt, *J* 15.3, 3.2, C(3)*H*), 7.18-

7.28 (2H, m, ArC(4,6)*H*), 7.32 (1H, dt, *J* 15.3, 2.2, C(2)*H*), 7.91-8.02 (2H, m, Ar(2',6')*H*); $^{13}C\{^1H\}$ NMR (101 MHz, $CDCl_3$) δ_C: 36.7 (Ar*C*H_2), 66.8 (O*C*H_2), 111.4 (Ar*C*(3)H), 115.8 (d, $^2J_{CF}$ 22.0, Ar*C*(3',5')H,), 121.4 (Ar*C*(5)H), 122.7 (Ar*C*(1)), 124.0 (Ar*C*(4)H), 129.2 (Ar*C*(6)H), 131.4 (d, $^3J_{CF}$ 9.4, Ar*C*(2',6')H), 131.6 (*C*(3)H), 133.8 (Ar*C*(1')), 142.3 (*C*(2)H), 155.9 (Ar*C*(2)O), 165.9 (d, $^1J_{CF}$ 257.0, Ar*C*(4')F), 178.0 (HO*C*=O), 188.2 (*C*=O); ^{19}F NMR (376 MHz, $CDCl_3$) δ_F: −105.0; m/z (NSI) $C_{18}H_{14}FO_4$ ($[M-H]^-$,100%) found 313.0878, requires 313.0882 (−1.2 ppm).

(*E*)-2-(2-((4-(4-Chlorophenyl)-4-oxobut-2-en-1-yl)oxy)phenyl)acetic acid (S18)

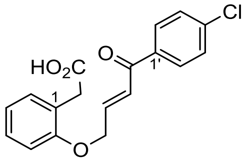

Following general procedure B: 2-(2-(Allyloxy)phenyl)acetic acid **S11** (1.30 g, 6.76 mmol, 1.0 equiv) dimethyl sulfide (0.99 mL, 13.53 mmol, 2.0 equiv) in CH_2Cl_2 (338 mL), followed by 1-(4-chlorophenyl)-2-(triphenyl-λ^5-phosphanylidene)ethan-1-one **S16c** (3.08 g, 7.43 mmol, 1.1 equiv) in $CHCl_3$ (50 mL) gave the product **S18** as a colouless solid (477 mg, 22%); mp: 120-122 °C; IR ν_{max} (film): 3041 (O-H), 2845 (C-H), 1703 (HOC=O), 1668 (C=O); ^1H NMR (500 MHz, $CDCl_3$) δ_H : 3.73 (2H, s, CH_2), 4.68 (2H, dd, *J* 3.3, 2.2, OCH_2), 6.76 (1H, dd, *J* 8.2, 1.0, ArC(3)*H*), 6.97 (1H, td, *J* 7.4, 1.0, ArC(5)*H*), 7.14 (1H, dt, *J* 15.3, 3.3, C(3)*H*), 7.21 (1H, dd, *J* 7.4, 1.7, ArC(4)*H*), 7.23-7.29 (1H, m, ArC(6)*H*), 7.29-7.37 (3H, m, C(2)*H* & ArC(3',5')*H*), 7.85-7.90 (2H, m, Ar(2',6')*H*); $^{13}C\{^1H\}$ NMR (126 MHz, $CDCl_3$) δ_C: 36.7 (*C*H_2), 66.8 (O*C*H_2), 111.4 (Ar*C*(3)H), 121.5 (Ar*C*(5)H), 122.6 (Ar*C*(1)), 123.9 (*C*(3)H), 129.1 (Ar*C*(3',5')H), 129.2 (Ar*C*(4)H), 130.2 (Ar*C*(2',6')H), 131.6 (Ar*C*(6)H), 135.8 (Ar*C*(1')), 139.6 (Ar*C*(4')), 142.7 (*C*(2)H), 155.9 (Ar*C*(2)), 177.9 (HO*C*=O), 188.5 (*C*=O), m/z (NSI) $C_{18}H_{14}{}^{35}ClO_4$ ($[M-H]^-$, 100%) found 329.0582, requires 329.0586 (−1.2 ppm).

(*E*)-2-(2-((4-(4-Methoxyphenyl)-4-oxobut-2-en-1-yl)oxy)phenyl)acetic acid (S19)

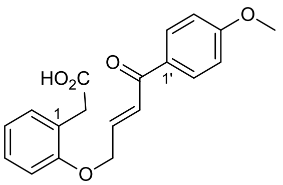

Following general procedure B: 2-(2-(Allyloxy)phenyl)acetic acid **S11** (0.90 g, 4.68 mmol, 1.0 equiv) dimethyl sulfide (0.67 mL, 9.36 mmol, 2.0 equiv) in CH_2Cl_2 (235 mL), followed by 1-(4-methoxyphenyl)-2-(triphenyl-λ^5-phosphanylidene)ethan-1-one (2.10 g, 5.15 mmol, 1.1 equiv) in $CHCl_3$ (50 mL) gave the product as a colourless solid (450 mg, 30%); mp: 103-105 °C; IR ν_{max} (film): 3032 (O-H), 2845 (C-H), 1714 (HOC=O), 1664 (C=O); ^{1}H NMR (500 MHz, $CDCl_3$) δ_H: 3.73 (2H, s, CH_2), 3.75 (3H, s, OCH_3), 4.58 (2H, app t, *J* 2.8, OC*H*$_2$), 6.72 (1H, d, *J* 8.2, ArC(3)*H*), 6.79-6.89 (2H, m, ArC(3',5')*H*), 6.94 (1H, app t, *J* 7.4, ArC(5)*H*), 7.08 (1H, dt, *J* 15.3, 3.3, C(3)*H*), 7.15-7.28 (2H, m, ArC(4,6)*H*), 7.32 (1H, dt, *J* 15.3, 2.2, C(2)*H*), 7.90-8.02 (2H, m, ArC(2',6')*H*); ^{13}C{^{1}H} NMR (126 MHz, $CDCl_3$) δ_C: 36.7 (*C*H$_2$), 55.5 (O*C*H$_3$), 66.9 (O*C*H$_2$), 111.4 (Ar*C*(3)H), 114.0 (Ar*C*(3',5')H), 121.2 (Ar*C*(5)H), 122.7 (Ar*C*(1)), 124.1 (Ar*C*(4)H), 129.1 (Ar*C*(6)H), 130.4 (Ar*C*(1')), 131.1 (Ar*C*(2',6')H), 131.5 (*C*(3)H), 141.2 (*C*(2)H), 156.1 (Ar*C*(2)), 163.6 (Ar*C*(4')), 177.9 (HO*C*=O), 188.1 (*C*=O); m/z (NSI) $C_{19}H_{17}O_5$ ([M−H]$^-$, 100%) found 325.1078, requires 325.1081 (−1.1 ppm).

(*E*)-2-(2-((4-Oxo-4-(p-tolyl)but-2-en-1-yl)oxy)phenyl)acetic acid (S20)

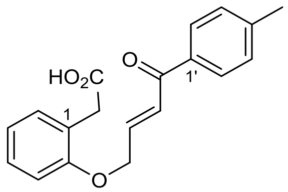

Following general procedure B: 2-(2-(Allyloxy)phenyl)acetic acid **S11** (1.00 g, 5.20 mmol, 1.0 equiv) dimethyl sulfide (0.76 mL, 10.4 mmol, 2.0 equiv) in CH_2Cl_2 (260 mL), followed by 1-(p-tolyl)-2-(triphenyl-λ^5-phosphanylidene)ethan-1-one **S16e** (2.17 g, 5.72 mmol, 1.1 equiv) in $CHCl_3$ (50 mL) gave the product **S20** as a colourless solid (430 mg, 26%); mp: 104-106 °C; IR ν_{max} (film): 3030 (O-H), 2908 (C-H), 1705 (HOC=O), 1664 (C=O); ^{1}H NMR (500 MHz, $CDCl_3$) δ_H: 2.35 (3H, s, C*H*$_3$), 3.73 (2H, s, C*H*$_2$), 4.64 (2H, dd, *J* 3.4, 2.2, OC*H*$_2$), 6.76 (1H, dd, *J* 8.3, 1.0, ArC(3)*H*), 6.96 (1H, app td, *J* 7.4, 1.0. ArC(5)*H*), 7.10 (1H, dt, *J* 15.4, 3.4, C(3)*H*), 7.15-7.28 (4H, m, ArC(4,6)*H* & ArC(3',5')*H*), 7.33 (1H, dt, *J* 15.4, 2.2, C(2)*H*), 7.82-7.92 (2H, m, ArC(2',6')*H*); ^{13}C{^{1}H} NMR (126 MHz, $CDCl_3$) δ_C: 21.8, (*C*H$_3$), 36.6 (*C*H$_2$), 67.0 (O*C*H$_2$), 111.5 (Ar*C*(3)H), 121.3 (Ar*C*(5)H), 122.8 (Ar*C*(1)), 124.6 (*C*(3)H), 128.9 (Ar*C*(3',5')H), 129.1 (Ar*C*(4)H), 129.5 (Ar*C*(2',6')H), 131.5 (Ar*C*(6)H), 135.0 (Ar*C*(1')), 141.7 (*C*(2)H), 144.0

(ArC(4')), 156.1 (ArC(2)), 177.5 (HOC=O), 189.4 (C=O); m/z (NSI) $C_{19}H_{17}O_4$ ([M−H]$^-$, 100%) found 309.1129, requires 309.1132 (−1.1 ppm).

(*E*)-2-(2-((4-Oxo-4-(4-(trifluoromethyl)phenyl)but-2-en-1-yl)oxy)phenyl)acetic acid (S21)

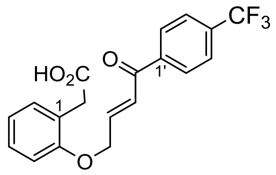

Following general procedure B: 2-(2-(Allyloxy)phenyl)acetic acid **S11** (0.90 g, 4.68 mmol, 1.0 equiv) dimethyl sulfide (0.67 mL, 9.36 mmol, 2.0 equiv) in CH_2Cl_2 (235 mL), followed by 1-(4-(trifluoromethyl)phenyl)-2-(triphenyl-λ^5-phosphanylidene)ethan-1-one **S16d** (2.3g, 5.15 mmol, 1.1 equiv) in $CHCl_3$ (50 mL) gave the product **S21** as a colourless solid (285 mg, 17%); mp: 106-108 °C; IR ν_{max} (film): 3030 (O-H), 2852 (C-H), 1697 (HOC=O), 1676 (C=O); ^{1}H NMR (400 MHz, $CDCl_3$) δ_H: 3.72 (2H, s, CH_2), 4.67-4.74 (2H, m, OCH_2), 6.71-6.79 (1H, m, ArC(3)H), 6.95 (1H, app td, J 7.4, 1.1, ArC(5)H), 7.13-7.29 (3H, m, C(3)H & ArC(4,6)H), 7.36 (1H, dt, J 15.4, 2.2, C(2)H), 7.63 (2H, d, J 8.2, ArC(3'5')H), 8.01 (2H, d, J 8.1, ArC(2',6')H); ^{13}C{^{1}H} NMR (101 MHz, $CDCl_3$) δ_C: 36.8 (CH$_2$), 66.8 (OCH$_2$), 111.3 (ArC(3)H), 121.5 (ArC(5)H), 122.7 (ArC(1)), 123.8 (q, $^1J_{CF}$ 273.6, CF$_3$), 123.9 (C(3)H), 125.8 (q, $^3J_{CF}$ 4.5, ArC(3',5')H), 129.0 (ArC(2',6')H), 129.2 (ArC(4)H), 131.6 (ArC(6)H), 134.3 (q, $^2J_{CF}$ 32.6, ArC(4')), 140.2 (ArC(1')), 143.5 (C(2)H), 155.8 (ArC(2)), 178.5 (HOC=O), 188.9 (C=O); ^{19}F NMR (376 MHz, $CDCl_3$) δ_F: −63.0; m/z (NSI) $C_{19}H_{14}F_3O_4$ ([M−H]$^-$, 100%) found 363.0844, requires 363.0850 (−1.6 ppm).

(*E*)-2-(2-((4-(3,5-Bis(trifluoromethyl)phenyl)-4-oxobut-2-en-1-yl)oxy)phenyl)acetic acid (S22).

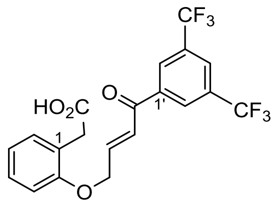

Following general procedure B: 2-(2-(Allyloxy)phenyl)acetic acid **S11** (0.65 g, 3.38 mmol, 1.0 equiv) dimethyl sulfide (0.50 mL, 6.76 mmol, 2.0 equiv) in CH_2Cl_2 (170 mL), followed by 1-

(3,5-bis(trifluoromethyl)phenyl)-2-(triphenyl-λ^5-phosphanylidene)ethan-1-one **S16i** (1.92 g, 3.72 mmol, 1.1 equiv) in $CHCl_3$ (50 mL) gave the product **S22** as a coloureless solid (346 mg, 24%); mp: 122-124 °C; IR ν_{max} (film): 3034 (O-H), 2922 (C-H), 1708 (HOC=O), 1680 (C=O); ^{1}H NMR (400 MHz, $CDCl_3$) δ_H: 3.70 (2H, s, CH_2), 4.83 (2H, dd, *J* 3.2, 2.2, OCH_2), 6.83 (1H, dd, *J* 8.3, 1.1, ArC(3)*H*), 6.95 (1H, app td, *J* 7.5, 1.1, ArC(5)*H*), 7.19 (1H, dd, *J* 7.4, 1.7, ArC(6)*H*), 7.23-7.34 (2H, m, C(3)*H* & ArC(4)*H*), 7.45 (1H, dt, *J* 15.3, 2.2, C(2)*H*), 8.02 (1H, m, ArC(4')*H*), 8.42 (2H, m, ArC(2',6')*H*); ^{13}C{^{1}H} NMR (126 MHz, $CDCl_3$) δ_C: 36.6 (*C*H_2), 66.7 (O*C*H_2), 111.4 (Ar*C*(3)H), 121.7 (Ar*C*(5)H), 122.8 (Ar*C*(1)), 123.1 (q, $^1J_{CF}$ 272.0, 2×*C*F_3), 123.3 (Ar*C*(4)), 126.3 (m, Ar*C*(4')H) 128.9 (m, Ar*C*(2',6')), 129.1 (Ar*C*(6)H), 131.7 (*C*(3)H), 132.4 (q, $^2J_{CF}$ 32.0, Ar*C*(3',5')), 138.9 (Ar*C*(1')), 144.7 (*C*(2)H), 155.7 (Ar*C*(2)), 176.4 (HO*C*=O), 186.9 (*C*=O); ^{19}F NMR (376 MHz, $CDCl_3$) δ_F: −62.9; m/z (NSI) $C_{20}H_{13}F_6O_4$ ([M−H]$^-$, 100%) found 431.0711, requires 431.0724 (−2.9 ppm).

(*E*)-2-(2-((4-Oxopent-2-en-1-yl)oxy)phenyl)acetic acid (S23)

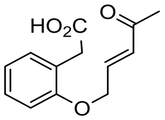

Following general procedure B: 2-(2-(Allyloxy)phenyl)acetic acid **S11** (1.00 g, 5.20 mmol, 1.0 equiv) dimethyl sulfide (0.76 mL, 10.4 mmol, 2.0 equiv) in CH_2Cl_2 (260 mL), followed by 1-(Triphenylphosphoranylidene)propan-2-one **S16g** (1.82 g, 5.72 mmol, 1.1 equiv) in $CHCl_3$ (50 mL) gave the product **S23** as a colourless solid (350 mg, 29%); mp: 129-130 °C; IR ν_{max} (film): 3022 (O-H), 2908 (C-H), 1716 (HOC=O), 1624 (C=O); ^{1}H NMR (500 MHz, $CDCl_3$) δ_H: 2.24 (3H, s, CH_3), 3.70 (2H, s, CH_2), 4.73 (2H, m, OCH_2), 6.45 (1H, dt, *J* 16.0, 2.0, C(3)*H*), 6.83 (1H, dd, *J* 8.2, 1.1, Ar*C*(3)*H*), 6.88 (1H, dt, *J* 16.0, 4.0, C(2)*H*), 6.97 (1H, app td, *J* 7.4, 1.1, Ar*C*(5)*H*), 7.22 (1H, dd, *J* 7.5, 1.7, Ar*C*(6)*H*), 7.24-7.31 (1H, m, ArC(4)*H*); ^{13}C{^{1}H} NMR (126 MHz, $CDCl_3$) δ_C: 27.1 (CH_3), 35.9 (*C*H_2), 66.2 (O*C*H_2), 111.7 (Ar*C*(3)H), 120.7 (Ar*C*(5)H), 124.0 (Ar*C*(1)), 128.3 (*C*(3)H), 129.4 (Ar*C*(4)H), 131.3 (Ar*C*(6)H), 142.6 (*C*(2)H), 155.8 (Ar*C*(2)), 172.6 (HO*C*=O), 197.8 (*C*=O); m/z (NSI) $C_{13}H_{13}O_4$ ([M−H]$^-$, 100%) found 233.0821, requires 233.0819 (+0.7 ppm).

(*E*)-2-(2-((4-(Naphthalen-2-yl)-4-oxobut-2-en-1-yl)oxy)phenyl)acetic acid (S24)

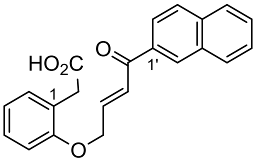

Following general procedure B: 2-(2-(Allyloxy)phenyl)acetic acid **S11** (1.3 g, 6.76 mmol, 1.0 equiv) dimethyl sulfide (0.99 mL, 13.53 mmol, 2.0 equiv) in CH_2Cl_2 (340 mL), followed by 1-(naphthalen-2-yl)-2-(triphenyl-λ^5-phosphanylidene)ethan-1-one **S16f** (3.2 g, 7.43 mmol, 1.1 equiv) in $CHCl_3$ (50 mL) gave the product **S24** as a colourless solid (330 mg, 14%); mp: 157-159 °C; IR ν_{max} (film): 3211 (O-H), 2920 (C-H), 1741 (HOC=O), 1660 (C=O); ^{1}H NMR (500 MHz, $CDCl_3$) δ_H: 3.68 (2H, s, C*H*$_2$), 4.95 (2H, dd, *J* 3.6, 2.1, OC*H*$_2$), 6.94 (1H, app td, *J* 7.4, 1.0, ArC(5)*H*), 6.99-7.07 (1H, m, ArC(3)*H*), 7.18 (1H, dt, *J* 15.4, 3.5, C(3)*H*), 7.27 (2H, m, 2×ArC*H*), 7.59-7.74 (3H, m, C(2)*H* & 2×ArC*H*), 7.98-8.09 (3H, m, 3×ArC*H*), 8.10-8.19 (1H, m, ArC*H*), 8.77 (1H, s, ArC*H*), 12.36 (1H, s, COO-*H*); ^{13}C{^{1}H} NMR (126 MHz, $CDCl_3$) δ_C: 36.5 (*C*H$_2$), 66.6 (O*C*H$_2$), 111.6 (Ar*C*(3)H), 120.7 (Ar*C*(5)H), 123.8(Ar*C*H), 123.9 (*C*(3)H), 124.1 (Ar*C*(1)), 127.0 (Ar*C*H), 127.7 (Ar*C*H), 128.3 (Ar*C*H), 128.6 (Ar*C*H), 128.9 (Ar*C*H), 129.8 (Ar*C*H), 130.6 (Ar*C*H), 131.4 (Ar*C*H), 132.3 (Ar*C*(8a')), 134.3 (Ar*C*(1')), 135.1 (Ar*C*(4a'), 143.3 (*C*(2)H), 155.8 (Ar*C*(2)), 173.0 (HO*C*=O), 188.6 (*C*=O); m/z (NSI) $C_{22}H_{17}O_4$ ([M−H]$^-$, 100%) found 345.1127, requires 345.1132 (−1.5 ppm).

(*E*)-2-(5-Methyl-2-((4-oxo-4-phenylbut-2-en-1-yl)oxy)phenyl)acetic acid (S25)

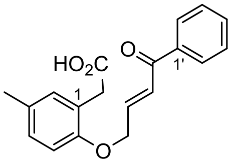

Following general procedure B: 2-(2-(Allyloxy)-5-methylphenyl)acetic acid **S12** (1.00 g, 4.85 mmol, 1.0 equiv) dimethyl sulfide (0.71 mL, 9.60 mmol, 2.0 equiv) in CH_2Cl_2 (240 mL), followed by 1-phenyl-2-(triphenyl-λ^5-phosphanylidene)ethan-1-one **S16a** (2.03 g, 5.34 mmol, 1.1 equiv) in $CHCl_3$ (50 mL) gave the product **S25** as a colourless solid (350 mg, 23%); mp: 122-124 °C; IR ν_{max} (film): 3020 (O-H), 2914 (C-H), 1707 (HOC=O), 1674 (C=O); ^{1}H NMR (500 MHz, $CDCl_3$) δ_H: 2.28 (3H, s, C*H*$_3$), 3.69 (2H, s, C*H*$_2$), 4.67 (2H, d, *J* 3.4, 2.2, OC*H*$_2$), 6.69 (1H, d, *J* 8.3, ArC(3)*H*), 7.01 (1H, d, *J* 2.2, ArC(6)*H*), 7.04 (1H, dd, *J* 8.3, 2.2, ArC(4)*H*), 7.12 (1H,

dt, *J* 15.4, 3.3, C(3)*H*), 7.29-7.40 (3H, m, C(2)*H* & ArC(3',5')*H*), 7.43-7.51 (1H, m, ArC(4')*H*), 7.90-7.98 (2H, m, ArC(2',6')*H*); $^{13}C\{^1H\}$ NMR (126 MHz, $CDCl_3$) δ_C: 20.5 (CH_3), 36.4 (CH_2), 67.0 (OCH_2), 111.4 (Ar*C*(3)H), 122.3 (Ar*C*(1)), 124.4 (*C*(3)H), 128.6 (Ar*C*(3',5')H), 128.7 (Ar*C*(2',6')H), 129.2 (Ar*C*(4)H), 130.5 (Ar*C*(5)), 132.1 (Ar*C*(6)H), 133.0 (Ar*C*(4')H), 137.4 (Ar*C*(1')), 142.3 (*C*(2)H), 153.8 (Ar*C*(2)), 178.1 (HO*C*=O), 189.8 (*C*=O); m/z (NSI) $C_{19}H_{17}O_4$ ($[M-H]^-$, 100%) found 309.1133, requires 309.1132 (+0.3 ppm).

(*E*)-2-(4-Methoxy-2-((4-oxo-4-phenylbut-2-en-1-yl)oxy)phenyl)acetic acid (S26)

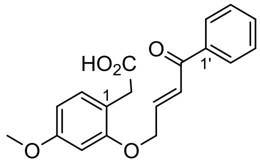

Following general procedure B: 2-(2-(Allyloxy)-4-methoxyphenyl)acetic acid **S13** (0.80 g, 3.60 mmol, 1.0 equiv) dimethyl sulfide (0.53 mL, 7.20 mmol, 2.0 equiv) in CH_2Cl_2 (180 mL), followed by 1-phenyl-2-(triphenyl-λ^5-phosphanylidene)ethan-1-one **S16a** (1.51 g, 3.96 mmol, 1.1 equiv) in $CHCl_3$ (50 mL) gave the product as a colourless solid (329 mg, 28%) ; mp: 168-170 °C; IR ν_{max} (film): 2835 (C-H), 1703 (HOC=O), 1676 (C=O); ^1H NMR (500 MHz, $CDCl_3$) δ_H: 3.54 (2H, s, C*H*₂), 3.74 (3H, s, OCH_3), 4.90 (2H, dd, *J* 3.6, 2.1, OC*H₂*), 6.50 (1H, dd, *J* 8.2, 2.4, Ar*C*(5)*H*), 6.58 (1H, d, *J* 2.4, Ar*C*(3)*H*), 7.05-7.17 (2H, m, C(3)*H* & Ar*C*(6)*H*), 7.45 (1H, dt, *J* 15.5, 2.1, C(2)*H*), 7.56 (2H, m, ArC(3'5')*H*), 7.62-7.71 (1H, m, Ar*C*(4')*H*), 7.97-8.08 (2H, m, Ar*C*(2',6')*H*), 12.23 (1H, s, COO-*H*); $^{13}C\{^1H\}$ NMR (126 MHz, $CDCl_3$) δ_C: 35.7 (CH_2), 55.2 (OCH_3), 66.6 (OCH_2), 99.2 (Ar*C*(3)H), 104.8 (Ar*C*(5)H), 116.3 (Ar*C*(1)), 123.8 (*C*(3)H), 128.5 (Ar*C*(3',5')H), 128.9 (Ar*C*(2',6')H), 131.6 (Ar*C*(6)H), 133.4 (Ar*C*(4')H), 137.0 (Ar*C*(1')), 143.4 (*C*(2)H), 156.6 (Ar*C*(2)), 159.6 (Ar*C*(4)), 173.2 (HO*C*=O), 188.8 (*C*=O); m/z (NSI) $C_{19}H_{19}O_5$ ($[M+H]^+$, 100%) found 327.1230, requires 327.1227 (+0.9 ppm).

(*E*)-2-(4-Fluoro-2-((4-oxo-4-phenylbut-2-en-1-yl)oxy)phenyl)acetic acid(S27)

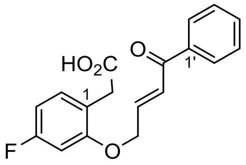

Following general procedure B: 2-(2-(Allyloxy)-4-fluorophenyl)acetic acid **S14** (0.30 g, 1.43 mmol, 1.0 equiv) dimethyl sulfide (0.21 mL, 2.86 mmol, 2.0 equiv) in CH_2Cl_2 (75 mL), followed by 1-phenyl-2-(triphenyl-λ^5-phosphanylidene)ethan-1-one **S16a** (0.60g, 1.57 mmol, 1.1 equiv) in $CHCl_3$ (25 mL) gave the product **S27** as a colourless solid (120 mg, 27%); mp: 158-160 °C; IR ν_{max} (film): 2916 (C-H), 1703 (HOC=O), 1672 (C=O); ^{1}H NMR (500 MHz, $CDCl_3$) δ_H: 3.61 (2H, s, C*H*$_2$), 4.94 (2H, dd, *J* 3.6, 2.1, OC*H*$_2$), 6.75 (1H, app td, *J* 8.4, 2.5, ArC(5)*H*), 6.94 (1H, dd, *J* 11.2, 2.5, ArC(3)*H*), 7.09 (1H, dt, *J* 15.5, 3.6, C(3)*H*), 7.23-7.30 (1H, m, ArC(6)*H*), 7.42 (1H, dt, *J* 15.5, 2.1, C(2)*H*), 7.56 (2H, m, ArC(3',5')*H*), 7.63-7.73 (1H, m, ArC(4')*H*), 7.97-8.09 (2H, m, ArC(2',6')*H*), 12.36 (1H, s, COO-*H*); ^{13}C{^{1}H} NMR (101 MHz, $CDCl_3$) δ_C: 35.6 (*C*H$_2$), 66.9 (O*C*H$_2$), 100.1 (d, $^2J_{CF}$ 26.3, Ar*C*(3)H), 106.6 (d, $^2J_{CF}$ 21.1, Ar*C*(5)H), 120.2 (d, $^4J_{CF}$ 3.3, Ar*C*(1)), 123.9 (*C*(3)H), 128.5 (Ar*C*(3',5')H), 128.9 (Ar*C*(2',6')H), 132.1 (d, $^3J_{CF}$ 10.2, Ar*C*(6)H), 133.4 (Ar*C*(4')H), 137.0 (Ar*C*(1')), 142.9 (C(2)*H*), 156.8 (d, $^3J_{CF}$ 10.2, Ar*C*(2)), 162.1 (d, $^1J_{CF}$ 240.1, Ar*C*(4)F), 172.7 (HO*C*=O), 188.8 (*C*=O); ^{19}F NMR (376 MHz, CDCl3) δ_F: −112.9; m/z (NSI) $C_{18}H_{14}FO_4$ ([M−H]$^-$, 100%) found 313.0874, requires 313.0882 (−2.5 ppm).

(*E*)-2-(1-((4-Oxo-4-phenylbut-2-en-1-yl)oxy)naphthalen-2-yl)acetic acid(S28)

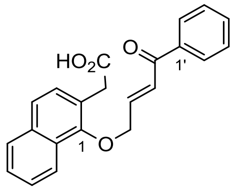

Following general procedure B: 2-(1-(Allyloxy)naphthalen-2-yl)acetic acid **S15** (0.50 g, 2.06 mmol, 1.0 equiv) dimethyl sulfide (0.30 mL, 9.60 mmol, 2.0 equiv) in CH_2Cl_2 (100 mL), followed by 1-phenyl-2-(triphenyl-λ^5-phosphanylidene)ethan-1-one **S16a** (0.86 g, 2.27 mmol, 1.1 equiv) in $CHCl_3$ (30 mL) gave the product **S28** as a colourless solid (160 mg, 22%) ; mp: 103-105 °C; IR ν_{max} (film): 2868 (C-H), 1705 (HOC=O), 1676 (C=O); ^{1}H NMR (400 MHz, $CDCl_3$) δ_H: 3.91 (2H, s, C*H*$_2$), 4.83 (2H, dd, *J* 3.8, 2.1, OC*H*$_2$), 7.21-7.30 (1H, dt, *J* 15.4, 3.8, C(3)*H*), 7.39 (1H, d, *J* 8.4, ArC*H*), 7.43-7.63 (6H, m, C(2)*H* & 5×ArC*H*), 7.65 (1H, d, *J* 8.4, ArC*H*), 7.82-7.89 (1H, m, ArC*H*), 7.99-8.08 (3H, m, 3×ArC*H*); ^{13}C{^{1}H} NMR (101 MHz, $CDCl_3$) δ_C: 35.4 (*C*H$_2$), 73.5 (O*C*H$_2$), 121.9 (Ar*C*(4)H), 122.7 (Ar*C*(8a)), 125.0 (2×Ar*C*H), 126.5 (*C*(3)H), 126.7 (Ar*C*H), 127.9 (Ar*C*(2)), 128.3 (Ar*C*H), 128.4 (Ar*C*H), 128.8 (Ar*C*(3',5')H), 128.9 (Ar*C*(2',6')H), 133.2 (Ar*C*(4')H), 134.7 (Ar*C*(4a)), 137.7 (Ar*C*(1')), 142.9 (*C*(2)H), 152.7

(Ar*C*(1)), 176.7 (HO*C*=O), 190.3 (*C*=O); m/z (NSI) $C_{22}H_{17}O_4$ ($[M-H]^-$, 100%) found 345.1132, requires 345.1126 (+1.7 ppm).

(*E*)-2-(2-((4-(4-Methoxyphenyl)-4-oxobut-2-en-1-yl)oxy)-5-methylphenyl)acetic acid (S29)

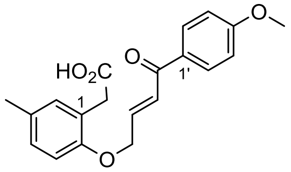

Following general procedure B: 2-(2-(Allyloxy)-5-methylphenyl)acetic acid **S12** (1.00 g, 4.85 mmol, 1.0 equiv) dimethyl sulfide (0.71 mL, 9.60 mmol, 2.0 equiv) in CH_2Cl_2 (240 mL), followed by 1-(4-methoxyphenyl)-2-(triphenyl-λ^5-phosphanylidene)ethan-1-one **S16b** (2.03 g, 5.34 mmol, 1.1 equiv) in $CHCl_3$ (50 mL) gave the product **S29** as a colourless solid (240 mg, 20%) ; mp: 146-148 °C; IR ν_{max} (film): 2914 (C-H), 1699 (HOC=O), 1668 (C=O); ^{1}H NMR (400 MHz, $CDCl_3$) δ_H: 2.27 (3H, d, *J* 0.7, ArCH_3), 3.68 (2H, s, CH_2), 3.77 (3H, s, OCH_3), 4.58 (2H, dd, *J* 3.3, 2.2, OCH_2), 6.63 (1H, d, *J* 8.1, ArC(3)*H*), 6.81-6.89 (2H, m, Ar(3',5')*H*), 6.98-7.04 (2H, m, ArC(4,6)*H*), 7.08 (1H, dt, *J* 15.4, 3.3, C(3)*H*), 7.32 (1H, *J* 15.4, 2.2, C(2)*H*), 7.92-8.00 (2H, m, ArC(2',6')*H*); ^{13}C{^{1}H} NMR (101 MHz, $CDCl_3$) δ_C: 20.5 (Ar*C*H$_3$), 36.7 (*C*H$_2$), 55.5 (O*C*H$_3$), 67.1 (O*C*H$_2$), 111.4 (Ar*C*(3)H), 114.0 (Ar*C*(3',5')H), 122.5 (Ar*C*(1)), 124.2 (*C*(3)H), 129.3 (Ar*C*(4)H), 130.5 (Ar*C*(5) & Ar*C*(1')), 131.2 (Ar*C*(2',6')H), 132.2 (Ar*C*(6)H), 141.5 (*C*(2)H), 154.0 (Ar*C*(2)), 163.6 (Ar*C*(4')), 177.8 (HO*C*=O), 188.2 (*C*=O); m/z (NSI) $C_{20}H_{21}O_5$ ($[M+H]^+$, 100%) found 341.1386, requires 341.1384 (+0.7 ppm).

(*E*)-2-(4-Methoxy-2-((4-(4-methoxyphenyl)-4-oxobut-2-en-1-yl)oxy)phenyl)acetic acid (S30)

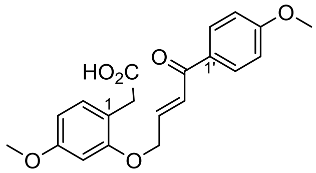

Following general procedure B: 2-(2-(Allyloxy)-5-methylphenyl)acetic acid **S13** (0.80 g, 3.60 mmol, 1.0 equiv) dimethyl sulfide (0.53 mL, 7.20 mmol, 2.0 equiv) in CH_2Cl_2 (180 mL), followed by 1-(4-methoxyphenyl)-2-(triphenyl-λ^5-phosphanylidene)ethan-1-one **S16b** (1.51 g, 3.96 mmol, 1.1 equiv) in $CHCl_3$ (50 mL) gave the product **S30** as a colourless solid (180 mg, 25%); mp: 161-163 °C; IR ν_{max} (film): 2902 (C-H), 1701 (HOC=O), 1668 (C=O); ^{1}H NMR (400 MHz, $CDCl_3$) δ_H: 3.54 (2H, s, CH_2), 3.74 (3H, s, OCH_3), 3.85 (3H, s, OCH_3), 4.88 (2H, dd, *J* 3.7,

2.1, OCH_2), 6.50 (1H, dd, *J* 8.3, 2.4, ArC(5)*H*), 6.58 (1H, d, *J* 2.4, Ar(3)*H*), 6.99-7.10 (3H, m, ArC(3',5')*H* & C(3)*H*), 7.13 (1H, d, *J* 8.3, ArC(6)*H*), 7.45 (1H, dt, *J* 15.4, 2.1, C(2)*H*), 7.95-8.15 (2H, m, ArC(2',6')*H*), 12.22 (1H, s, COO-*H*); ^{13}C{^{1}H} NMR (101 MHz, $CDCl_3$) δ_C: 35.7 (*C*H_2), 55.2 (O*C*H_3), 55.6 (O*C*H_3), 66.6 (O*C*H_2), 99.2 (Ar*C*(3)H), 104.8 (Ar*C*(5)H), 114.1 (Ar*C*(3',5')H), 116.2 (Ar*C*(1)), 123.7 (*C*(3)H), 129.9 (Ar*C*(1')), 130.8 (Ar*C*(2',6')H), 131.6 (Ar*C*(6)H), 142.2 (*C*(2)H), 156.6 (Ar*C*(2)), 159.6 (Ar*C*(4)), 163.3 (Ar*C*(4')), 173.1 (HO*C*=O), 186.9 (*C*=O); m/z (NSI) $C_{20}H_{19}O_6$ ([M−H]$^-$, 100%) found 355.1179, requires 355.1187 (−2.3 ppm).

(*E*)-2-(1-((4-(4-Methoxyphenyl)-4-oxobut-2-en-1-yl)oxy)naphthalen-2-yl)acetic acid (S31)

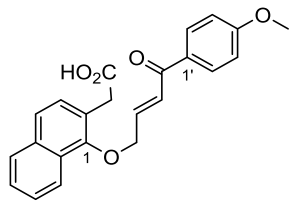

Following general procedure B: 2-(1-(allyloxy)naphthalen-2-yl)acetic acid **S15** (605 mg, 2.68 mmol, 1.0 equiv) dimethyl sulfide (0.39 mL, 5.37 mmol, 2.0 equiv) in CH_2Cl_2 (135 mL), followed by 1-(4-methoxyphenyl)-2-(triphenyl-λ^5-phosphanylidene)ethan-1-one **S16b** (1.21 g, 2.951 mmol, 1.1 equiv) in $CHCl_3$ (30 mL) gave the product **S31** as a colourless solid (197 mg, 20%); mp: 154-156 °C; IR ν_{max} (film): 2931 (C-H), 1699 (OHC=O), 1668 (C=O); ^{1}H NMR (400 MHz, $CDCl_3$) δ_H: 3.82 (2H, s, CH_2), 3.87 (3H, s, OCH_3), 4.81 (2H, dd, *J* 4.4, 1.9, OCH_2), 7.03-7.21 (3H, m, ArC(3',5')*H* & C(3)*H*), 7.45 (1H, d, *J* 8.4, ArC(4)*H*), 7.49-7.63 (3H, m, ArC(6,7)*H* & *C*(2)H), 7.72 (1H, d, *J* 8.4, ArC(3)*H*), 7.95 (1H, dd, *J* 7.6, 1.7, ArC(5)*H*), 8.00-8.12 (3H, m, ArC(8)*H* & ArC(2',6')*H*), 12.46 (1H, s, COO-*H*); ^{13}C{^{1}H} NMR (101 MHz, $CDCl_3$) δ_C: 35.4 (*C*H_2), 55.6 (OCH_3), 73.2 (O*C*H_2), 114.2 (Ar*C*(3',5')H), 121.6 (Ar*C*(4)H), 124.1 (Ar*C*(3)H), 124.4 (Ar*C*(2)), 124.8 (*C*(3)H), 126.1 (Ar*C*(6)H), 126.4 (Ar*C*(7)H), 127.4 (Ar*C*(8a)), 128.1 (Ar*C*(5)H), 129.1 (Ar*C*(8)H), 129.9 (Ar*C*(4a)), 130.9 (Ar*C*(2',6')H), 133.8 (Ar*C*(1')), 142.4 (*C*(2)H), 152.1 (Ar*C*(1)), 163.4 (Ar*C*(4')), 172.7 (HO*C*=O), 187.4 (*C*=O); m/z (NSI) $C_{23}H_{19}O_5$ ([M−H]$^-$, 100%) found 375.1229, requires 375.1238 (−2.3 ppm).

Optimization of Michael Addition-Lactonization

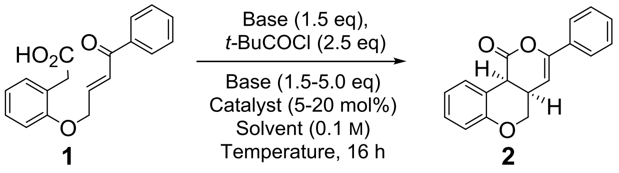

Entry	Catalyst (mol%)	Base (eq)	solvent	temp	yield[a]	er[b] (4a*R*,10b*S*:4a *S*,10b*R*)	dr[c]
1	(+) BTM (20)	DIPEA (1.5+2.5)	CH_2Cl_2	r.t	84%	13:87	>99:1
2	(+) BTM (20)	DIPEA (1.5+2.5)	Toluene	r.t	Not isolated	ND	>99:1
3	(+) BTM (20)	DIPEA (1.5+2.5)	Et_2O	r.t	Not isolated	ND	>99:1
4	(+) BTM (20)	DIPEA (1.5+2.5)	THF	r.t	62%	23:77	>99:1
5	(+) BTM (20)	DIPEA (1.5+2.5)	MeCN	r.t	66%	22:78	>99:1
6	(+) BTM (20)	DIPEA (1.5+2.5)	DCE	r.t	70%	15:85	>99:1
7	(+) BTM (20)	DIPEA (1.5+2.5)	$CHCl_3$	r.t	85%	7:93	>99:1
8[d]	(+) BTM (20)	DIPEA (1.5+2.5)	$CHCl_3$	r.t	84%	7:93	>99:1
9	(+) BTM (20)	DIPEA (1.5+2.5)	$CHCl_3$	0°C	87%	7:93	>99:1
10	(+) BTM (20)	DIPEA (1.5+2.5)	$CHCl_3$	- 10°C	83%	6:94	>99:1
11	(+) BTM (20)	DIPEA (1.5+2.5)	$CHCl_3$	-78 - 0°C	67%	6:94	>99:1
12	(+) BTM (20)	DIPEA (1.5+2.5)	$CHCl_3$	40 - 45°C	87%	7:93	>99:1
13	(+) BTM (20)	DIPEA (1.5+2.5)	$CHCl_3$	0°C	85%	7:93	>99:1
14	(+) BTM (20)	DIPEA (1.5+5.0)	$CHCl_3$	0°C	85%	5:95	>99:1
15[e]	(+) BTM (20)	DIPEA (1.5+1.5)	$CHCl_3$	0°C	62%	7:93	>99:1
16	(−) TM.HCl (20)	DIPEA (1.5+2.5)	$CHCl_3$	0°C	87%	7:93	>99:1
17[f]	(−) TM.HCl (10)	DIPEA (1.5+2.5)	$CHCl_3$	0°C	85%	6:94	>99:1
18[f]	(+) BTM (10)	DIPEA (1.5+2.5)	$CHCl_3$	0°C	86%	7:93	>99:1
19[f]	(−) TM.HCl (5)	DIPEA (1.5+2.5)	$CHCl_3$	0°C	85%	7:93	>99:1
20[f]	(+) BTM (5)	DIPEA (1.5+2.5)	$CHCl_3$	0°C	65%	7:93	>99:1
21[f]	(−) TM.HCl (2.5)	DIPEA (1.5+2.5)	$CHCl_3$	0°C	60%	6:94	>99:1

a. Isolated yield; *b*. Determined by chiral HPLC analysis; *c*. Determined by ^{1}H NMR spectroscopic analysis of the crude reaction product; *d.* 0.05 M solution in $CHCl_3$ instead of 0.1 M $CHCl_3$; *e*. Reaction conversion only 70-80% from TLC; *f*. Reaction time was 5-6 h

Investigation of change in dr and er over time

To probe the observed change in dr and er over time, the reaction was monitored by ^{1}H NMR spectroscopy at 0 °C, using toluene as an internal standard. The reaction was monitored every 5 minutes until 1 h (Figure S1). From the ^{1}H NMR spectra it was observed that, along with the major *cis*- chromenone **2**, a minor diastereomer *trans*-**7** (7-8%) was also formed. Upon warming to rt over a further 2 h, the signal assigned to the minor diastereomer (4.50 ppm, blue H in Figure S1), *trans*-chromenone had disappeared and the signal assigned to the major *cis*-chromenone (4.18 ppm, pink H in Figure S1) had increase in size. This experiment indicates that the minor *trans*-diastereoisomer was converted to the major *cis*-diastereoisomer**,** presumably *via* base-mediated epimerization of the hydrogen adjacent to the carbonyl (C(10b)H)to afford thermodynamically-favoured *cis*-chromenone **2**. Assuming catalyst control of the C(10b)H stereocentre, this epimerization would result in the formation of the opposite enantiomer of the major *cis*-diastereoisomer, and could therefore account for the drop in er observed over the course of the reaction.

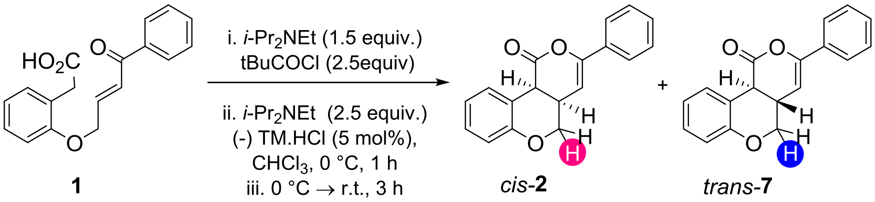

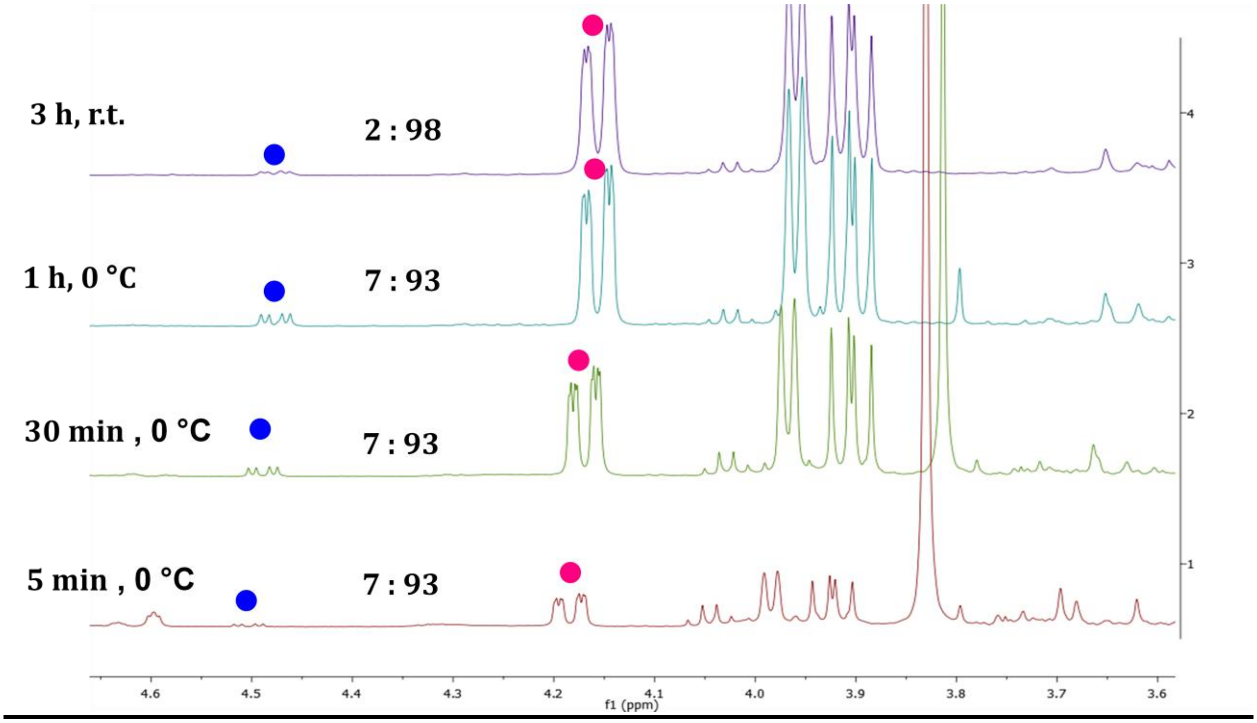

Figure S1. $CDCl_3$, 500 MHz: Up to 1 h full conversion with 93:7 dr, but after warming to rt and keeping for 3 h showed an increase in diastereomeric ratio of 98:2 dr

^{1}H NMR spectroscopy experiment on base-mediated epimerization.

In order to investigate if the erosion in er occurred *via* a base-mediated epimerization of *trans*-(4a*S*, 10b*S*) chromenone *trans*-**7** to at the C(10b)H stereocentre to give *cis*-(4a*S*, 10b*R*) chromenone *ent*-*cis*-**2**, a 80:20 mixture of *trans:cis* was treated with *i*-Pr_2NEt in $CDCl_3$ and ^{1}H NMR spectra recorded at different intervals at r.t. (Scheme S2). It was found that the protons at 5.79 (green) and 4.58 (blue) assigned to *trans*-chromenone *trans*-**7** converted to the corresponding protons of *cis*-**2** at 5.83 (orange) and 4.26 (pink) (>99:1 dr and 38:62 er (4a*R*,10b*S*:4a*S*,10b*R*)). The major enantiomer of the *cis*-chromenone obtained was 4a*S*,10b*R* (*ent-cis*-**2**), indicating the configuration of *trans*-**7** as (4a*S*, 10b*S*), assuming epimerization at the C(4a) centre. A small amount of impurities present in the mixture was not affected.

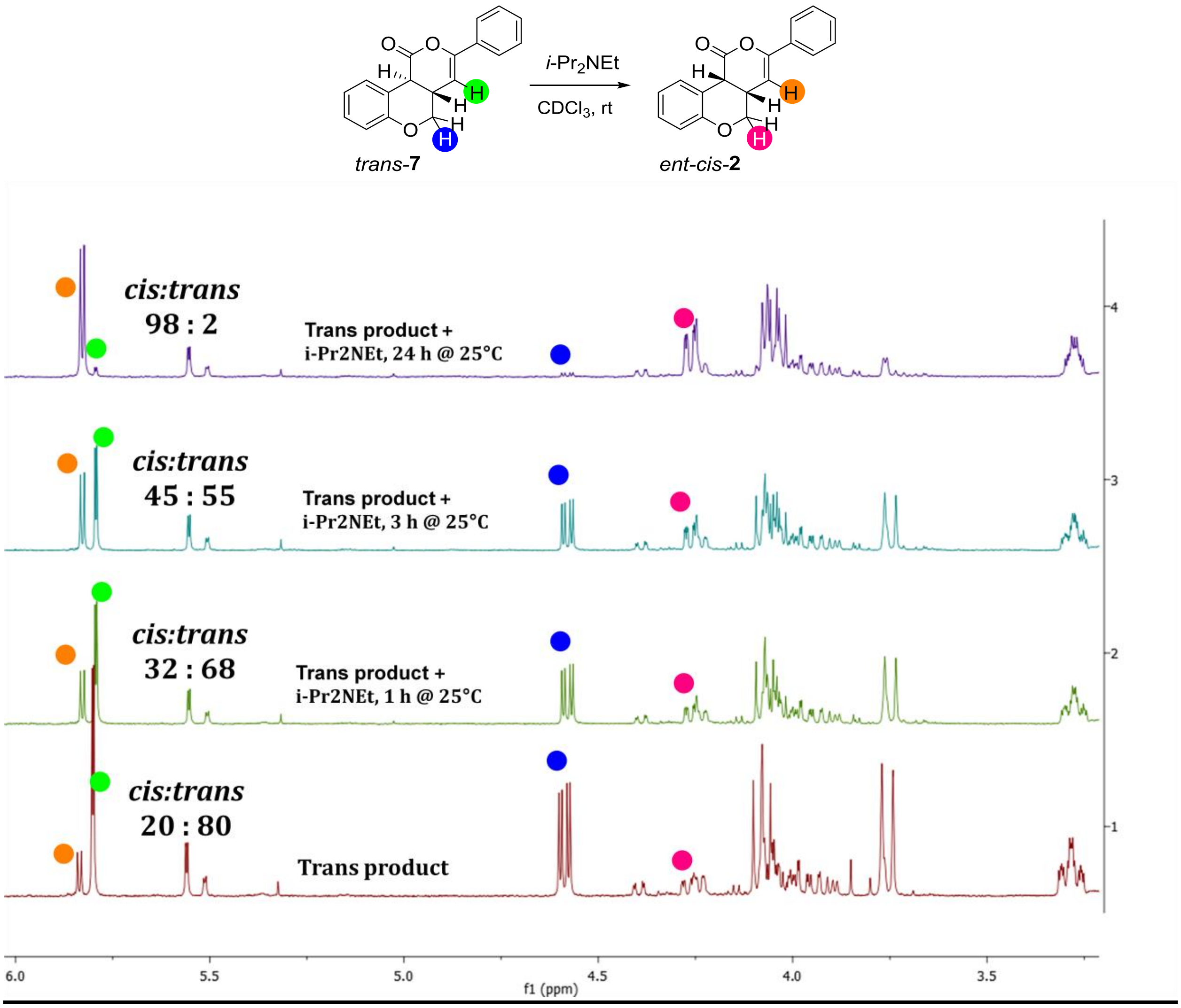

Figure S2. $CDCl_3$, 500 MHz: ^{1}HNMR monitoring of Base mediated epimerization.

Work up modification to achieve high product er and dr.

A reaction was divided in to three portions upon complete conversion after 4 h (98:2 er). The first portion was evaporated under vacuum to remove $CHCl_3$ and subjected to ^{1}H NMR spectroscopic and HPLC analysis. This showed a single diastereomer with 93:7 er. The second portion was purified imediately using column chromatography, however once again HPLC analysis determined a 93:7 er. The third portion was analyzed after a water work up and also showed a single diastereomer with 93:7 er. Then we postulated that addition of aqueous acid to the mixture upon reaction completion would protonate both base and catalyst and prevent

epimerization. This was readily achieved by treatment of the reaction mixture with a cold 0.1 M aqu.HCl wash upon completion of the reaction at 0 °C. This procedure resulted in diastereomeric mixture of 93:7 dr from ^{1}H NMR analysis, with the major diastereoisomer *cis*-(4aR, 10bS) isolated in 98:2 er.

Isothiourea-Catalyzed Michael Addition-Lactonization

(4a*R*,10b*S*)-3-Phenyl-4a,10b-dihydropyrano[4,3-*c*]chromen-1(5*H*)-one (2)

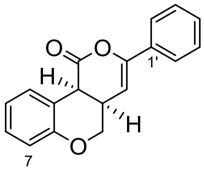

Following general procedure C: (*E*)-2-(2-((4-oxo-4-phenylbut-2-en-1-yl)oxy)phenyl)acetic acid (**1)** (59 mg, 0.20 mmol, 1.0 equiv), $CHCl_3$ (2.0 mL), (*i*-Pr)$_2$NEt (53 µL, 0.30 mmol, 1.5 equiv), pivaloyl chloride (62 µL, 0.50 mmol, 2.5 equiv), followed by TM·HCl (2.4 mg, 5 mol%), (*i*-Pr)$_2$NEt (87 µL, 0.50 mmol, 2.5 equiv). Washed with 0.1 M aqu.HCl (2 × 2.4 mL) to give (4a*R*, 10b*S*)-3-phenyl-4a,10b-dihydropyrano[4,3-*c*]chromen-1(5*H*)-one **2** as a colourless solid (39 mg, 70%), and a mixture of diastereomers (9 mg, 16%); Data for pure *cis*-product: mp: 99-101 °C; $[\alpha]_D^{20}$ +1.67 (*c* 1.0, $CHCl_3$); Chiral HPLC analysis, Chiralpak AD-H (95:5 hexane:IPA, flow rate 1.00 mLmin^{-1}, 254 nm, 40 °C) t_R (4a*R*,10b*S*): 20.0 min, t_R (4a*S*,10b*R*): 14.3 min, 98:2 er; IR ν_{max} (film): 2879 (C-H), 1761(C=O); ^{1}H NMR (500 MHz, $CDCl_3$) δ_H: 3.20-3.29 (1H, m, C(4a)*H*), 3.97-4.10 (2H, m, OC(5)H^AH^B & C(10b)*H*), 4.24 (1H, ddd, *J* 11.3, 3.1, 1.2, OC(5)H^AH^B), 5.81 (1H, d, *J* 5.0, C(4)*H*), 6.87 (1H, dd, *J* 8.2, 1.2, ArC(7)*H*), 6.98 (1H, app td, *J* 7.5, 1.3, ArC(9)*H*), 7.22 (1H, m, ArC(4')*H*), 7.30-7.44 (4H, m, ArC(3',5')*H* & ArC(8,10)*H*), 7.55-7.72 (2H, m, ArC(2',6')*H*); ^{13}C{^{1}H} NMR (126 MHz, $CDCl_3$) δ_C: 31.0 (*C*(4a)H), 40.0 (*C*(10b)H), 65.9 (OCH_2), 98.7 (*C*(4)H), 115.4 (Ar*C*(10a)), 117.3 (Ar*C*(7)H), 121.2 (Ar*C*(9)H), 124.8 (Ar*C*(3',5')H), 128.6 (Ar*C*(2',6')H), 129.4 (Ar*C*(8)H), 129.6 (Ar*C*(4')H), 131.2 (Ar*C*(10)H), 131.8 (Ar*C*(1')), 151.7 (*C*(3)), 153.9 (Ar*C*(6a)), 167.7 (C=O); m/z (NSI) $C_{18}H_{15}O_3$ ($[M+H]^+$, 100%) found 279.1019, requires 279.1016 (+1.2 ppm).

(4a*R*,10b*S*)-3-(4-Fluorophenyl)-4a,10b-dihydropyrano[4,3-*c*]chromen-1(5*H*)-one (8)

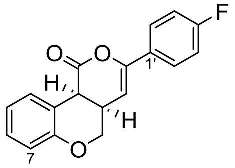

Following general procedure C: (*E*)-2-(2-((4-(4-Fluorophenyl)-4-oxobut-2-en-1-yl)oxy)phenyl)acetic acid (94 mg, 0.30 mmol, 1.0 equiv), $CHCl_3$ (3.0 mL), (*i*-Pr)$_2$NEt (80 μL, 0.15 mmol, 1.5 equiv), pivaloyl chloride (93 μL, 0.25 mmol, 2.5 equiv), followed by TM·HCl (3.6 mg, 5 mol %), (*i*-Pr)$_2$NEt (131 μL, 0.50 mmol, 2.5 equiv), 0.1M aqu.HCl (2 × 3.6 mL), to give (4a*R*,10b*S*)-3-(4-fluorophenyl)-4a,10b-dihydropyrano[4,3-*c*]chromen-1(5*H*)-one **8** as a light yellow solid (71 mg, 79%) and a mixture of diastereomers (10 mg, 11%); Data for pure *cis*-product: mp: 138-140 °C; $[\alpha]_D^{20}$ +1.77 (*c* 1.0, $CHCl_3$); Chiral HPLC analysis, Chiralpak AD-H (95:5 hexane:IPA, flow rate 1.00 mLmin^{-1}, 254 nm, 40 °C) t_R (4a*R*,10b*S*): 20.2 min, t_R (4a*S*,10b*R*): 16.0 min, 96:4 er; IR ν_{max} (film): 2924 (C-H), 1761 (C=O); ^{1}H NMR (400 MHz, $CDCl_3$) δ_H: 3.20-3.30 (1H, m, C(4a)*H*), 3.95-4.09 (2H, m, OC(5)H^AH^B & C(10b)*H*), 4.24 (1H, ddd, *J* 11.3, 3.1, 1.2, OC(5)H^AH^B), 5.73 (1H, d, *J* 4.9, C(4)*H*), 6.86 (1H, dd, *J* 8.2, 1.2, ArC(7)*H*), 6.98 (1H, app td, *J* 7.5, 1.2, Ar(9)*H*), 7.01-7.10 (2H, m, ArC(3',5')*H*), 7.22 (1H, m, Ar(8)*H*), 7.29-7.35 (1H, m, ArC(10)*H*), 7.55-7.65 (2H, m, ArC(2',6')*H*); ^{13}C{^{1}H} NMR (126 MHz, $CDCl_3$) δ_C: 31.0 (C(4a)*H*), 40.0 (C(10b)*H*), 65.9 (OCH_2), 98.4 (C(4)*H*), 115.3 (Ar*C*(10a)), 115.7 (d, $^2J_{CF}$ 21.5, Ar*C*(3',5')H), 117.3 (Ar*C*(7)H), 121.3 (Ar*C*(9)H), 126.9 (d, $^3J_{CF}$ 8.2, Ar*C*(2',6')H), 128.0 (Ar*C*(1')), 129.5 (Ar*C*(8)H), 131.2 (Ar*C*(10)H), 151.0 (*C*(3)), 153.9 (Ar*C*(6a)), 163.5 (d, $^1J_{CF}$ 245.2, Ar*C*(4')F), 167.6 (C=O); ^{19}F NMR (500 MHz, $CDCl_3$) δ_F: −111.2; m/z (NSI) $C_{18}H_{14}O_3F$ ([M+H]$^+$, 100%) found 297.0922, requires 297.0927 (−1.7 ppm).

(4a*R*,10b*S*)-3-(4-Chlorophenyl)-4a,10b-dihydropyrano[4,3-*c*]chromen-1(5*H*)-one (9)

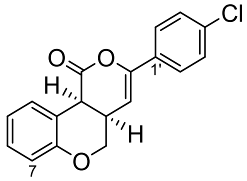

Following general procedure C: (*E*)-2-(2-((4-(4-Chlorophenyl)-4-oxobut-2-en-1-yl)oxy)phenyl)acetic acid (330 mg, 0.20 mmol, 1.0 equiv), $CHCl_3$ (2.0 mL), (*i*-Pr)$_2$NEt (53 μL,

0.30 mmol, 1.5 equiv), pivaloyl chloride (62 µL, 0.50 mmol, 2.5 equiv), followed by TM·HCl (2.4 mg, 5 mol%), (*i*-Pr)$_2$NEt (87 µL, 0.50 mmol, 2.5 equiv), 0.1 M aqu.HCl (2 × 2.4 mL) to give (4a*R*,10b*S*)-3-(4-chlorophenyl)-4a,10b-dihydropyrano[4,3-c]chromen-1(5*H*)-one **9** as a pale yellow solid (46 mg, 74%) and a mixture of diastereomers (7 mg, 11%); Data for pure *cis*-product: mp: 128-130 °C; $[\alpha]_D^{20}$ +1.70 (*c* 1.0, CHCl$_3$); Chiral HPLC analysis, Chiralpak AD-H (95:5 hexane:IPA, flow rate 1.00 mLmin^{-1}, 254 nm, 40 °C) t_R (4a*R*,10b*S*): 17.8 min, t_R (4a*S*,10b*R*): 14.2 min, 98:2 er; IR ν_{max} (film): 2924 (C-H), 1761 (C=O); ^{1}H NMR (400 MHz, CDCl$_3$) δ_H: 3.21-3.30 (1H, m, C(4a)*H*), 3.98-4.09 (2H, m, OC(5)H^AH^B & C(10b)*H*), 4.24 (1H, ddd, *J* 11.3, 3.1, 1.2, OC(5)H^AH^B), 5.79 (1H, d, *J* 4.7, C(4)*H*), 6.86 (1H, dd, *J* 8.2, 1.2, ArC(7)*H*), 6.98 (1H, app td, *J* 7.5, 1.2, ArC(9)*H*), 7.22 (1H, m, Ar(8)*H*), 7.29-7.37 (3H, m, ArC(10)*H* & ArC(3',5')*H*), 7.50-7.58 (2H, m, ArC(2',6')*H*); ^{13}C{^{1}H} NMR (126 MHz, CDCl$_3$) δ_C: 31.0 (*C*(4a)H), 39.9 (*C*(10b)H), 65.9 (O*C*H$_2$), 99.2 (*C*(4)H), 115.2 (Ar*C*(10a)), 117.4 (Ar*C*(7)H), 121.3 (Ar*C*(9)H), 126.1 (Ar*C*(3',5')H), 128.9 (Ar*C*(2',6')H), 129.5 (Ar*C*(8)H), 130.3 (Ar*C*(1')), 131.1 (Ar*C*(10)H), 135.5 (Ar*C*(4')), 150.8 (*C*(3)), 153.8 (Ar*C*(6a)), 167.5 (C=O); m/z (NSI) $C_{18}H_{14}O_3{}^{35}Cl$ ([M+H]$^+$, 100%) found 313.0630, requires 313.0626 (+1.3 ppm).

(4a*R*,10b*S*)-3-(4-Methoxyphenyl)-4a,10b-dihydropyrano[4,3-*c*]chromen-1(5*H*)-one (10)

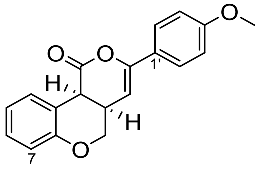

Following general procedure C: (*E*)-2-(2-((4-(4-Methoxyphenyl)-4-oxobut-2-en-1-yl)oxy)phenyl)acetic acid (98 mg, 0.30 mmol, 1.0 equiv), CHCl$_3$ (3.0 mL), (*i*-Pr)$_2$NEt (80 µL, 0.45 mmol, 1.5 equiv), pivaloyl chloride (93 µL, 0.75 mmol, 2.5 equiv), followed by TM·HCl (3.6 mg, 5 mol%), (*i*-Pr)$_2$NEt (131 µL, 0.75 mmol, 2.5 equiv). 0.1 M aqu.HCl (2 × 3.6 mL), to give (4a*R*,10b*S*)-3-(4-methoxyphenyl)-4a,10b-dihydropyrano[4,3-*c*]chromen-1(5*H*)-one **10** as a pale yellow solid (63 mg, 68%) and a mixture of diastereomers (20 mg, 22%); Data for pure *cis*-product : mp: 116-118 °C; $[\alpha]_D^{20}$ +1.70 (*c* 1.0, CHCl$_3$); Chiral HPLC analysis, Chiralpak AD-H (95:5 hexane:IPA, flow rate 1.00 mLmin^{-1}, 254 nm, 30 °C) t_R (4a*R*,10b*S*): 27.6 min, t_R (4a*S*,10b*R*): 23.0 min, 98:2 er; IR ν_{max} (film): 2933 (C-H), 1759 (C=O); ^{1}H NMR (400 MHz, CDCl$_3$) δ_H: 3.17-3.27 (1H, m, C(4a)*H*), 3.82 (3H, s, OCH_3), 3.93-4.06 (2H, m, OC(5)H^AH^B &

C(10b)*H*), 4.23 (1H, ddd, *J* 11.3, 3.1, 1.1, OC(5)H^A*H^B*), 5.66 (1H, d, *J* 5.1, C(4)*H*), 6.81-6.93 (3H, m, ArC(7)*H* & ArC(3',5')*H*), 6.97 (1H, app td, *J* 7.5, 1.2, ArC(9)*H*), 7.16-7.25 (1H, m, ArC(8)*H*), 7.32 (1H, app dt, *J* 7.7, 1.1, ArC(10)*H*), 7.50-7.61 (2H, m, ArC(2',6')*H*); $^{13}C\{^1H\}$ NMR (126 MHz, $CDCl_3$) δ_C: 30.9 (*C*(4a)H), 40.1 (*C*(10b)H), 55.5 (O*C*H_3), 66.1 (O*C*H_2), 96.6 (*C*(4)H), 114.0 (Ar*C*(3',5')H), 115.5 (Ar*C*(10a)), 117.3 (Ar*C*(7)H), 121.2 (Ar*C*(9)H), 124.4 (Ar*C*(1')), 126.3 (Ar*C*(2',6')H), 129.4 (Ar*C*(8)H), 131.3 (Ar*C*(10)H), 151.6 (*C*(3)), 153.9 (Ar*C*(6a)), 160.7 (Ar*C*(4')), 167.9 (C=O); m/z (NSI) $C_{19}H_{17}O_4$ ($[M+H]^+$, 100%) found 309.1123, requires 309.1121 (+0.5 ppm).

(4a*R*,10b*S*)-3-(*p*-Tolyl)-4a,10b-dihydropyrano[4,3-*c*]chromen-1(5*H*)-one (11)

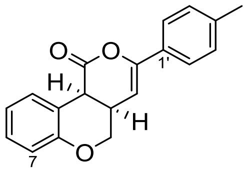

Following general procedure C: (*E*)-2-(2-((4-Oxo-4-(p-tolyl)but-2-en-1-yl)oxy)phenyl)acetic acid (31 mg, 0.10 mmol, 1.0 equiv), $CHCl_3$ (1.0 mL), (*i*-Pr)$_2$NEt (27 µL, 0.15 mmol, 1.5 equiv), pivaloyl chloride (31 µL, 0.25 mmol, 2.5 equiv), followed by TM·HCl (1.2 mg, 5 mol %), (*i*-Pr)$_2$NEt (44 µL, 0.25 mmol, 2.5 equiv), 0.1 M aqu.HCl (2 × 1.2 mL), to give (4a*R*,10b*S*)-3-(*p*-tolyl)-4a,10b-dihydropyrano[4,3-*c*]chromen-1(5*H*)-one **11** as a pale yellow solid (19 mg, 65%) and a mixture of diastereomers (6 mg, 21%); Data for pure *cis*-product: mp: 124-126 °C; $[\alpha]_D^{20}$ +1.47 (*c* 1.0, $CHCl_3$); Chiral HPLC analysis, Chiralpak AD-H (95:5 hexane:IPA, flow rate 1.00 mLmin^{-1}, 254 nm, 40 °C) t_R (4a*R*,10b*S*): 17.8 min, t_R (4a*S*,10b*R*): 14.2 min, 98:2 er; IR ν_{max} (film): 2922 (C-H), 1768 (C=O); ^1H NMR (500 MHz, $CDCl_3$) δ_H: 2.36 (3H, s, C*H_3*), 3.18-3.28 (1H, m, C(4a)*H*), 3.93-4.06 (2H, m, OC(5)*H^A*H^B & C(10b)*H*), 4.23 (1H, ddd, *J* 11.4, 3.1, 1.3, OC(5)H^A*H^B*), 5.75 (1H, d, *J* 5.2, C(4)*H*), 6.82-6.90 (1H, m, ArC(7)*H*), 6.97 (1H, app td, *J* 7.5, 1.3, Ar(9)*H*), 7.11-7.25 (3H, m, ArC(8)*H* & ArC(3',5')*H*), 7.32 (1H, dd, *J* 7.8, 1.7, ArC(10)*H*), 7.51 (2H, d, *J* 8.0, ArC(2',6')*H*); $^{13}C\{^1H\}$ NMR (126 MHz, $CDCl_3$) δ_C: 21.4 (*C*H_3), 30.9 (*C*(4a)H), 40.0 (*C*(10b)H), 66.0 (O*C*H_2), 97.7 (*C*(4)H), 115.5 (Ar*C*(10a)), 117.3 (Ar*C*H), 121.2 (Ar*C*H), 124.7 (Ar*C*(3',5')H), 129.0 (Ar*C*(1')), 129.3 (Ar*C*(2',6')H), 129.4 (Ar*C*H), 131.3 (Ar*C*H), 139.7 (Ar*C*(4')), 151.8 (*C*(3)), 153.9 (Ar*C*(6a)), 167.9 (C=O); m/z (NSI) $C_{19}H_{17}O_3$ ($[M+H]^+$, 100%) found 293.1170, requires 293.1172 (−0.8 ppm).

(4a*R*,10b*S*)-3-(4-(Trifluoromethyl)phenyl)-4a,10b-dihydropyrano[4,3-*c*]chromen-1(5*H*)-one (12)

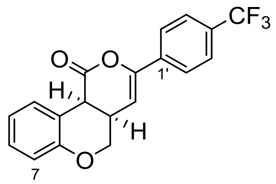

Following general procedure C: (*E*)-2-(2-((4-Oxo-4-(4-(trifluoromethyl)phenyl)but-2-en-1-yl)oxy)phenyl)acetic acid (72.8 mg, 0.20 mmol, 1.0 equiv), $CHCl_3$ (2.0 mL), (*i*-Pr)$_2$NEt (53 μL, 0.30 mmol, 1.5 equiv), pivaloyl chloride (62 μL, 0.50 mmol, 2.5 equiv), followed by TM·HCl (2.4 mg, 5 mol%), (*i*-Pr)$_2$NEt (87 μL, 0.50 mmol, 2.5 equiv), 0.1 M aqu.HCl (2 × 2.4 mL), to give (4a*R*,10b*S*)-3-(4-(trifluoromethyl)phenyl)-4a,10b-dihydropyrano[4,3-c]chromen-1(5*H*)-one **12** as a pale yellow solid (49 mg, 71% (95% pure from ^{1}H NMR, residual solvents present)) and a mixture of diastereomers (14 mg, 20%); Data for pure *cis*-product: mp: 108-110 °C; $[\alpha]_D^{20}$ +1.12 (*c* 1.0, $CHCl_3$); Chiral HPLC analysis, Chiralpak AD-H (95:5 hexane:IPA, flow rate 1.00 mLmin^{-1}, 254 nm, 40 °C) t_R (4a*R*,10b*S*): 17.8 min, t_R (4a*S*,10b*R*): 15.6 min, 96:4 er; IR ν_{max} (film): 2924 (C-H), 1766 (C=O); ^{1}H NMR (400 MHz, $CDCl_3$) δ_H: 3.26-3.35 (1H, m, C(4a)*H*), 4.01-4.14 (2H, m, OC(5)H^AH^B & C(10b)*H*), 4.26 (1H, ddd, *J* 11.3, 3.0. 1.1, OC(5)H^AH^B), 5.93 (1H, d, *J* 4.8, C(4)*H*), 6.86 (1H, dd, *J* 8.2, 1.2, ArC(7)*H*), 6.98 (1H, app td, *J* 7.5, 1.3, ArC(9)*H*), 7.22 (1H, m, ArC(8)*H*), 7.30-7.36 (1H, m, ArC(10)*H*), 7.59-7.65 (2H, m, ArC(3',5')*H*), 7.69-7.76 (2H, m, ArC(2',6')*H*); ^{13}C{^{1}H} NMR (126 MHz, $CDCl_3$) δ_C: 31.1 (*C*(4a)H), 39.9 (*C*(10b)H), 65.8 (O*C*H_2), 101.1 (*C*(4)H), 115.0 (Ar*C*(10a)), l17.4 (Ar*C*(7)H), 121.4 (Ar*C*(9)H), 123.9 (q, $^1J_{CF}$ 272.2, *C*F_3), 125.1 (Ar*C*(2',6')H), 125.7 (q, $^3J_{CF}$ 3.9, Ar*C*(3',5')H), 129.6 (Ar*C*(8)H), 131.0 (Ar*C*(10)H), 131.4 (q, $^2J_{CF}$ 32.8, Ar*C*(4')), 135.1 (Ar*C*(1')), 150.5 (*C*(3)), 153.8 (Ar*C*(6a)), 167.3 (C=O); ^{19}F NMR (376 MHz, $CDCl_3$) δ_F: −62.8; m/z (NSI) $C_{19}H_{14}O_3F_3$ ($[M+H]^+$, 100%) found 347.0893, requires 347.0895 (−0.2 ppm).

(4a*R*,10b*S*)-3-(3,5-Bis(trifluoromethyl)phenyl)-4a,10b-dihydropyrano[4,3-*c*]chromen-1(5*H*)-one (13)

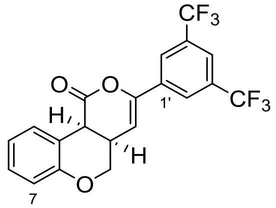

Following general procedure C: (*E*)-2-(2-((4-(3,5-Bis(trifluoromethyl)phenyl)-4-oxobut-2-en-1-yl)oxy)phenyl)acetic acid (86.4 mg, 0.20 mmol, 1.0 equiv), $CHCl_3$ (2.0 mL), $(i\text{-Pr})_2NEt$ (53 µL, 0.30 mmol, 1.5 equiv), pivaloyl chloride (62 µL, 0.50 mmol, 2.5 equiv), followed by TM·HCl (2.4 mg, 5 mol%), $(i\text{-Pr})_2NEt$ (87 µL, 0.50 mmol, 2.5 equiv), 0.1 M aqu.HCl (2 × 2.4 mL), to give (4aR,10bS)-3-(3,5-bis(trifluoromethyl)phenyl)-4a,10b-dihydropyrano[4,3-*c*]chromen-1(5H)-one **13** as a light yellow solid (31 mg, 37%, single diastereomer (95% pure from ^{1}H NMR, residual solvents present)); mp: 78-80 °C; $[\alpha]_D^{20}$ +0.37 (*c* 0.5, $CHCl_3$); Chiral HPLC analysis, Chiralpak OD-H (97:3 hexane:IPA, flow rate 1.00 mLmin^{-1}, 220 nm, 40 °C) t_R (4a*R*,10b*S*): 19.9 min, t_R (4a*S*,10b*R*): 17.4 min, 91:9 er; IR ν_{max} (film): 2924(C-H), 1701 (C=O); ^{1}H NMR (400 MHz, $CDCl_3$) δ_H: 3.31-3.40 (1H, m, C(4a)*H*), 4.08-4.16 (2H, m, OC(5)H^AH^B & C(10b)*H*), 4.28 (1H, ddd, *J* 11.3, 2.9, 1.0, OC(5)H^AH^B), 6.03 (1H, d, *J* 4.5, C(4)*H*), 6.88 (1H, dd, *J* 8.2, 1.2, ArC(7)*H*), 7.00 (1H, app td, *J* 7.5, 1.3, ArC(9)*H*), 7.20-7.26 (1H, m, ArC(8)*H*), 7.33 (1H, app dt, *J* 7.7, 1.3, ArC(10)*H*), 7.85 (1H, bs, ArC(4')*H*), 8.04 (2H, bs, ArC(2',6')*H*); $^{13}C\{^1H\}$ NMR (126 MHz, $CDCl_3$) δ_C: 31.2 (*C*(4a)H), 39.8 (*C*(10b)H), 65.7 (O*C*H_2), 102.3 (*C*(4)H), 114.6 (Ar*C*(10a)), 117.6 (Ar*C*(7)H), 121.6 (Ar*C*(9)H), 123.0 (m, Ar*C*(4')H), 123.1 (q, $^1J_{CF}$ 272.9, 2×*C*F_3), 124.9 (bs, $^3J_{CF}$ Ar*C*(2',6')H), 129.8 (Ar*C*(8)H), 130.8 (Ar*C*(10)H), 132.3 (q, $^2J_{CF}$ 33.6, Ar*C*(3',5')), 133.9 (Ar*C*(1')), 149.1 (*C*(3)), 153.8 (Ar*C*(6a)), 166.8 (*C*=O); ^{19}F NMR (376 MHz, $CDCl_3$) δ_F: −62.9; m/z (NSI) $C_{20}H_{13}O_3F_6$ ($[M+H]^+$, 100%) found 415.0764, requires 415.0769 (−1.2 ppm).

(4a*R*,10b*S*)-3-Methyl-4a,10b-dihydropyrano[4,3-*c*]chromen-1(5*H*)-one (14)

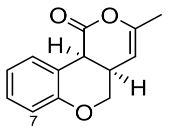

Following general procedure C: (*E*)-2-(2-((4-Oxopent-2-en-1-yl)oxy)phenyl)acetic acid (93 mg, 0.40 mmol, 1.0 equiv), $CHCl_3$ (4.0 mL), $(i\text{-Pr})_2NEt$ (106 µL, 0.60 mmol, 1.5 equiv), pivaloyl

chloride (124 µL, 1.00 mmol, 2.5 equiv), followed by TM·HCl (19.2 mg, 20 mol%), (*i*-Pr)$_2$NEt (174 µL, 1.00 mmol, 2.5 equiv), 0.1 M aqu.HCl (2 × 4.8 mL) at 0°C, to give (4a*R*,10b*S*)-3-methyl-4a,10b-dihydropyrano[4,3-*c*]chromen-1(5*H*)-one **14** as a pale yellow solid (25 mg, 29%, single diastereomer); mp: 86-88 °C; $[\alpha]_D^{20}$ +0.60 (*c* 1.0, CHCl$_3$); Chiral HPLC analysis, Chiralpak AD-H (95:5 hexane:IPA, flow rate 1.00 mLmin^{-1}, 254 nm, 40 °C) t_R (4a*R*,10b*S*): 9.8 min, t_R (4a*S*,10b*R*): 8.7 min, 71:29 er; IR ν_{max} (film): 2922 (C-H), 1757 (C=O), 1219 (C-O); ^{1}H NMR (500 MHz, CDCl$_3$) δ_H: 1.89 (3H, t, *J* 1.3, C*H*$_3$), 2.97-3.04 (1H, m, C(4a)*H*), 3.80-3.95 (2H, m, OC(5)H^AH^B & C(10b)*H*), 4.13 (1H, ddd, *J* 11.2, 3.1, 1.3, OC(5)H^AH^B), 5.01 (1H, dq, *J* 4.9, 1.3, C(4)*H*), 6.84 (1H, dd, *J* 8.2, 1.3, ArC(7)*H*), 6.96 (1H, app td, *J* 7.6, 1.3, ArC(9)*H*), 7.21 (1H, m, ArC(8)*H*), 7.28 (1H, ddd, *J* 7.6, 1.7, 0.8, ArC(10)*H*); ^{13}C{^{1}H} NMR (126 MHz, CDCl$_3$) δ_C: 18.9 (*C*H$_3$), 30.5 (*C*(4a)H), 39.8 (*C*(10b)H), 66.0 (O*C*H$_2$), 98.9 (*C*(4)H), 115.6 (Ar*C*(10a), 117.2 (Ar*C*(7)H), 121.1 (Ar*C*(9)H), 129.3 (Ar*C*(8)H), 131.3 (Ar*C*(10)H), 151.4 (*C*(3)), 153.9 (Ar*C*(6a)), 168.2 (*C*=O); m/z (NSI) $C_{13}H_{13}O_3$ ([M+H]$^+$, 100%) found 217.0860, requires 217.0859 (+0.4 ppm).

(4a*R*,10b*S*)-3-(Naphthalen-2-yl)-4a,10b-dihydropyrano[4,3-*c*]chromen-1(5*H*)-one (15)

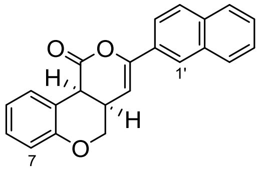

Following general procedure C: (*E*)-2-(2-((4-(Naphthalen-2-yl)-4-oxobut-2-en-1-yl)oxy)phenyl)acetic acid (69.2 mg, 0.20 mmol, 1.0 equiv), CHCl$_3$ (2.0 mL), (*i*-Pr)$_2$NEt (53 µL, 0.30 mmol, 1.5 equiv), pivaloyl chloride (62 µL, 0.50 mmol, 2.5 equiv), followed by TM·HCl (2.4 mg, 5 mol%), (*i*-Pr)$_2$NEt (87 µL, 0.50 mmol, 2.5 equiv), 0.1 M aqu.HCl (2 × 2.4 mL), to give (4a*R*,10b*S*)-3-(naphthalen-2-yl)-4a,10b-dihydropyrano[4,3-*c*]chromen-1(5*H*)-one **15** as a pale yellow solid (46 mg, 70%) and a mixture of diastereomers (14 mg, 21%); Data for pure *cis*-product: mp: 115-117 °C; $[\alpha]_D^{20}$ +1.47 (*c* 1.0, CHCl$_3$); Chiral HPLC analysis, Chiralpak AD-H (95:5 hexane:IPA, flow rate 1.00 mLmin^{-1}, 254 nm, 40 °C) t_R (4a*R*,10b*S*): 26.8 min, t_R (4a*S*,10b*R*): 22.9 min, 97:3 er; IR ν_{max} (film): 2922(C-H), 1757 (C=O); ^{1}H NMR (400 MHz, CDCl$_3$) δ_H: 3.25-3.34 (1H, m, C(4a)*H*), 4.01-4.12 (2H, m, OC(5)H^AH^B & C(10b)*H*), 4.28 (1H, app ddt, *J* 11.4, 3.1, 1.5, OC(5)H^AH^B), 5.94 (1H, d, *J* 4.8, C(4)*H*), 6.88 (1H, dd, *J* 8.2, 1.4,

ArC(7)*H*), 7.00 (1H, app td, *J* 7.5, 1.3, ArC(9)*H*), 7.19-7.26 (1H, m, ArC*H*), 7.36 (1H, dd, *J* 7.7, 1.7, ArC*H*), 7.51 (2H, m, 2×ArC*H*), 7.65 (1H, dd, *J* 8.7, 1.8, ArC*H*), 7.79-7.89 (3H, m, 3×ArC*H*), 8.16 (1H, d, *J* 1.8, ArC*H*); $^{13}C\{^1H\}$ NMR (101 MHz, $CDCl_3$) δ_C: 31.1 (*C*(4a)H), 40.0 (*C*(10b)H), 66.0 (O*C*H_2), 99.2 (*C*(4)H), 115.4 (Ar*C*(10a)), 117.3 (Ar*C*(7)H), 121.3 (Ar*C*(9)H), 122.0 (Ar*C*H), 124.4 (Ar*C*H), 126.8 (Ar*C*H), 127.0 (Ar*C*H), 127.7 (Ar*C*H), 128.4 (Ar*C*H), 128.7 (Ar*C*(8a')), 128.8 (Ar*C*H), 129.5 (Ar*C*H), 131.2 (Ar*C*H), 133.1 (Ar*C*(4a')), 133.7 (Ar*C*(2')), 151.6 (*C*(3)), 153.9 (Ar*C*(6a)), 167.8 (*C*=O); m/z (NSI) $C_{22}H_{17}O_3$ ($[M+H]^+$, 100%) found 329.1173, requires 329.1178 (−1.5 ppm).

(4a*R*,10b*S*)-9-Methyl-3-phenyl-4a,10b-dihydropyrano[4,3-*c*]chromen-1(5*H*)-one (16)

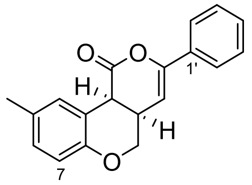

Following general procedure C: (*E*)-2-(5-Methyl-2-((4-oxo-4-phenylbut-2-en-1-yl)oxy)phenyl)acetic acid (62 mg, 0.20 mmol, 1.0 equiv), $CHCl_3$ (2.0 mL), (*i*-Pr)$_2$NEt (53 µL, 0.30 mmol, 1.5 equiv), pivaloyl chloride (62 µL, 0.50 mmol, 2.5 equiv), followed by TM·HCl (2.4 mg, 5 mol%), (*i*-Pr)$_2$NEt (87 µL, 0.50 mmol, 2.5 equiv), 0.1 M aqu.HCl (2 × 2.4 mL) to give (4a*R*,10b*S*)-9-methyl-3-phenyl-4a,10b-dihydropyrano[4,3-*c*]chromen-1(5*H*)-one **16** as a colourless solid (48 mg, 82%) and a mixture of diastereomers (5 mg, 9%); Data for pure *cis*-product: mp: 120-122 °C; $[\alpha]_D^{20}$ +1.86 (*c* 1.0, $CHCl_3$); Chiral HPLC analysis, Chiralpak AD-H (95:5 hexane:IPA, flow rate 1.00 $mLmin^{-1}$, 254 nm, 40 °C) t_R (4a*S*,10b*R*): 12.9 min, t_R (4a*R*,10b*S*): 15.2 min, 98:2 er; IR ν_{max} (film): 2922 (C-H), 1761 (C=O); ^1H NMR (400 MHz, $CDCl_3$) δ_H: 2.29 (3H, s, CH_3), 3.18-3.27 (1H, m, C(4a)*H*), 3.96-4.07 (2H, m, OC(5)H^AH^B & C(10b)*H*), 4.20 (1H, ddd, *J* 11.3, 3.0, 1.2, OC(5)H^AH^B), 5.80 (1H, d, *J* 4.8, C(4)*H*), 6.76 (1H, d, *J* 8.3, ArC(7)*H*), 7.01 (1H, m, ArC(8)*H*), 7.12 (1H, m, ArC(10)*H*), 7.30-7.42 (3H, m, ArC(3',4',5')*H*), 7.57-7.69 (2H, m, ArC(2',6')*H*); $^{13}C\{^1H\}$ NMR (101 MHz, $CDCl_3$) δ_C: 20.7 (*C*H_3), 31.1 (*C*(4a)H), 40.0 (*C*(10b)H), 66.0 (O*C*H_2), 98.9 (*C*(4)H), 114.9 (Ar*C*(10a)), 117.0 (Ar*C*(7)H), 124.8 (Ar*C*(3',5')H), 128.6 (Ar*C*(2',6')H), 129.5 (Ar*C*(4')H), 130.2 (Ar*C*(8)H), 130.5 (Ar*C*(9)), 131.1 (Ar*C*(10)H, 131.8 (Ar*C*(1')), 151.6 (*C*(3)), 151.7 (Ar*C*(6a)), 167.9 (*C*=O); m/z (NSI) $C_{19}H_{17}O_3$ ($[M+H]^+$, 100%) found 293.1175, requires 293.1172 (+1.0 ppm).

(4a*R*,10b*S*)-8-Methoxy-3-phenyl-4a,10b-dihydropyrano[4,3-*c*]chromen-1(5*H*)-one) (17)

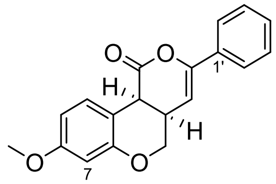

Following general procedure C: (*E*)-2-(4-Methoxy-2-((4-oxo-4-phenylbut-2-en-1-yl)oxy)phenyl)acetic acid (65.2 mg, 0.20 mmol, 1.0 equiv), $CHCl_3$ (2.0 mL), (*i*-Pr)$_2$NEt (53 µL, 0.30 mmol, 1.5 equiv), pivaloyl chloride (62 µL, 0.50 mmol, 2.5 equiv), followed by TM·HCl (2.4 mg, 5 mol%), (*i*-Pr)$_2$NEt (87 µL, 0.50 mmol, 2.5 equiv), 0.1 M aqu.HCl (2 × 2.4 mL), to give (4a*R*,10b*S*)-8-methoxy-3-phenyl-4a,10b-dihydropyrano[4,3-*c*]chromen-1(5*H*)-one) **17** as a solid (44 mg, 71%) and a mixture of diastereomers (13 mg, 21%); Data for pure *cis*-product: mp: 116-118 °C; $[\alpha]_D^{20}$ +0.36 (*c* 0.5, $CHCl_3$); Chiral HPLC analysis, Chiralpak AD-H (95:5 hexane:IPA, flow rate 1.00 mLmin^{-1}, 254 nm, 40 °C) t_R (4a*S*,10b*R*): 19.7 min, t_R (4a*R*,10b*S*): 29.7 min, 98:2 er; IR ν_{max} (film): 2933 (C-H), 1763 (C=O); ^{1}H NMR (400 MHz, $CDCl_3$) δ_H: 3.17-3.27 (1H, m, C(4a)*H*), 3.76 (3H, s, OCH_3), 3.94-4.04 (2H, m, OC(5)H^AH^B & C(10b)*H*), 4.21 (1H, ddd, *J* 11.2, 3.1, 1.2, OC(5)H^AH^B), 5.78 (1H, d, *J* 5.0, C(4)*H*), 6.40 (1H, d, *J* 2.6, ArC(7)*H*), 6.57 (1H, dd, *J* 8.6, 2.6, ArC(9)*H*), 7.22 (1H, dd, *J* 8.6, 0.8, ArC(10)*H*), 7.32-7.42 (3H, m, ArC(3',4',5')*H*), 7.59-7.65 (2H, m, ArC(2',6')*H*); ^{13}C{^{1}H} NMR (101 MHz, $CDCl_3$) δ_C: 31.1 (*C*(4a)H), 39.4 (*C*(10b)H), 55.4 (O*C*H_3), 66.0 (O*C*H_2), 98.7 (*C*(4)H), 101.9 (Ar*C*(7)H), 107.5 (Ar*C*(10a)), 108.3 (Ar*C*(9)H), 124.8 (Ar*C*(3',5')H), 128.6 (Ar*C*(2',6')H), 129.5 (Ar*C*(4')H), 131.7 (Ar*C*(10)H), 131.8 (Ar*C*(1')), 151.6 (*C*(3)), 154.8 (Ar*C*(6a)), 160.5 (Ar*C*(8)), 168.1 (*C*=O); m/z (NSI) $C_{19}H_{17}O_4$ ($[M+H]^+$, 100%) found 309.1124, requires 309.1121 (+0.9 ppm).

(4a*R*,10b*S*)-8-Fluoro-3-phenyl-4a,10b-dihydropyrano[4,3-*c*]chromen-1(5*H*)-one (18)

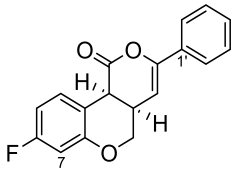

Following general procedure C: (*E*)-2-(4-Fuoro-2-((4-oxo-4-phenylbut-2-en-1-yl)oxy)phenyl)acetic acid (31 mg, 0.10 mmol, 1.0 equiv), $CHCl_3$ (1.0 mL), (*i*-Pr)$_2$NEt (26 µL, 0.15 mmol, 1.5 equiv), pivaloyl chloride (31 µL, 0.25 mmol, 2.5 equiv), followed by TM·HCl

(1.2 mg, 5 mol%), (i-Pr)$_2$NEt (44 µL, 0.25 mmol, 2.5 equiv), 0.1 M aqu.HCl (2 × 1.2 mL), to give (4a*R*,10b*S*)-8-fluoro-3-phenyl-4a,10b-dihydropyrano[4,3-*c*]chromen-1(5*H*)-one **18** as a light yellow solid (19 mg, 64%) and a mixture of diastereomers (4 mg, 14%); Data for pure *cis*-product: mp: 108-110 °C; $[\alpha]_D^{20}$ +1.88 (*c* 1.0, $CHCl_3$); Chiral HPLC analysis, Chiralpak AD-H (95:5 hexane:IPA, flow rate 1.00 mLmin^{-1}, 254 nm, 40 °C) t_R (4a*R*,10b*S*): 21.1 min, t_R (4a*S*,10b*R*): 13.0 min, 96:4 er; IR ν_{max} (film): 2922 (C-H), 1759 (C=O); ^{1}H NMR (400 MHz, $CDCl_3$) δ_H: 3.19-3.29 (1H, m, C(4a)*H*), 3.94-4.02 (2H, m, OC(5)H^AH^B & C(10b)*H)*, 4.24 (1H, ddd, *J* 11.3, 3.2, 1.3, OC(5)H^AH^B), 5.79 (1H, d, *J* 5.1, C(4)*H*), 6.58 (1H, dd, *J* 10.0, 2.6, ArC(7)*H*), 6.71 (1H, app td, *J* 8.4, 2.6, ArC(9)*H*), 7.24-7.32 (1H, m, ArC(10)*H*), 7.35-7.42 (3H, m, ArC(3',4',5')*H*), 7.57-7.66 (2H, m, ArC(2',6')*H*); ^{13}C{^{1}H} NMR (101 MHz, $CDCl_3$) δ_C: 30.8 (*C*(4a)H), 39.5 (*C*(10b)H), 66.0 (O*C*H_2), 98.3 (*C*(4)H), 104.4 (d, $^2J_{CF}$ 24.2, Ar*C*(7)H), 108.8 (d, $^2J_{CF}$ 22.0, Ar*C*(9)H), 111.3 (Ar*C*(10a)), 124.9 (Ar*C*(3',5')H), 128.7 (Ar*C*(2',6')H), 129.7 (Ar*C*(4')H), 131.7 (Ar*C*(1')), 132.4 (d, $^3J_{CF}$ 10.1, Ar*C*(10)H), 151.9 (*C*(3)), 155.0 (d, $^3J_{CF}$ 9.9, Ar*C*(6a)), 163.1 (d, $^1J_{CF}$ 245.0 Ar*C*(8)F), 167.6 (*C*=O); ^{19}F NMR (400 MHz, $CDCl_3$) δ_F: −112.1; m/z (NSI) $C_{18}H_{14}O_3F$ ([M+H]$^+$, 100%) found 297.0923, requires 297.0927 (−1.3 ppm).

(4a*S*,12a*R*)-2-Phenyl-12,12a-dihydrobenzo[*h*]pyrano[4,3-*c*]chromen-4(4a*H*)-one (19)

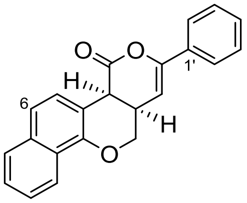

Following general procedure C: (*E*)-2-(1-((4-Oxo-4-phenylbut-2-en-1-yl)oxy)naphthalen-2-yl)acetic acid (52 mg, 0.15 mmol, 1.0 equiv), $CHCl_3$ (1.5 mL), (i-Pr)$_2$NEt (39 ul, 0.23 mmol, 1.5 equiv), pivaloyl chloride (46 ul, 0.38 mmol, 2.5 equiv), followed by TM·HCl (1.8 mg, 5 mol%), (i-Pr)$_2$NEt (65 ul, 0.38 mmol, 2.5 equiv), 0.1 M aqu.HCl (2 × 1.8 mL), to give (4a*S*,12a*R*)-2-phenyl-12,12a-dihydrobenzo[*h*]pyrano[4,3-*c*]chromen-4(4a*H*)-one **19** as a colourless solid (38 mg, 77%) and a mixture of diastereomers (7 mg, 14%); Data for pure *cis*-product: mp: 134-136 °C; $[\alpha]_D^{20}$ +0.43 (*c* 1.0, $CHCl_3$); Chiral HPLC analysis, Chiralpak AD-H (95:5 hexane:IPA, flow rate 1.00 mLmin^{-1}, 254 nm, 40 °C) t_R (4a*S*,12a*R*): 28.8 min, t_R (4a*R*,12a*S*): 15.0 min, 97:3 er; IR ν_{max} (film): 2908 (C-H), 1763 (C=O); ^{1}H NMR (400 MHz, $CDCl_3$) δ_H: 3.28-3.38 (1H, m,

C(12a)*H*), 4.10-4.20 (2H, m, OC(12)H^AH^B & C(4a)*H*), 4.54 (1H, ddd, *J* 11.2, 3.2, 1.4, OC(12)H^AH^B), 5.85 (1H, d, *J* 5.2, C(1)*H*), 7.34-7.43 (4H, m, 4×ArC*H*), 7.44-7.53 (3H, m, 3×ArC*H*), 7.60-7.68 (2H, m, 2×ArC*H*), 7.75-7.82 (1H, m, ArC*H*), 8.14-8.21 (1H, m, ArC*H*); $^{13}C\{^1H\}$ NMR (101 MHz, $CDCl_3$) δ_C: 31.0 (*C*(12a)H), 40.1 (*C*(4a)H), 66.3 (O*C*H_2), 98.5 (*C*(1)H), 109.3 (Ar*C*(4b)), 120.6 (Ar*C*H), 121.8 (Ar*C*H), 124.8 (Ar*C*(3',5')H), 125.0 (Ar*C*(10a)), 125.7 (Ar*C*H), 126.8 (Ar*C*H), 127.7 (Ar*C*H), 128.0 (Ar*C*H), 128.7 (Ar*C*(2',6')H), 129.6 (Ar*C*(4')H), 131.8 (Ar*C*(1')), 134.2 (Ar*C*(6a)), 149.5 (*C*(2)), 151.8 (Ar*C*(10b)), 168.0 (*C*=O); m/z (NSI) $C_{22}H_{17}O_3$ ($[M+H]^+$, 100%) found 329.1171, requires 329.1178 (−2.1 ppm).

(4a*R*,10b*S*)-3-(4-Methoxyphenyl)-9-methyl-4a,10b-dihydropyrano[4,3-*c*]chromen-1(5*H*)-one (20)

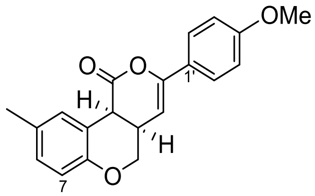

Following general procedure C: (*E*)-2-(2-((4-(4-Methoxyphenyl)-4-oxobut-2-en-1-yl)oxy)-5-methylphenyl)acetic acid (68 mg, 0.20 mmol, 1.0 equiv), $CHCl_3$ (2.0 mL), (*i*-Pr)$_2$NEt (52 µL, 0.30 mmol, 1.5 equiv), pivaloyl chloride (62 µL, 0.50 mmol, 2.5 equiv), followed by TM·HCl (2.4 mg, 5 mol%), (*i*-Pr)$_2$NEt (87 µL, 0.50 mmol, 2.5 equiv), 0.1 M aqu.HCl (2 × 2.4 mL), to give (4a*R*,10b*S*)-3-(4-methoxyphenyl)-9-methyl-4a,10b-dihydropyrano[4,3-*c*]chromen-1(5*H*)-one **20** as a colourless solid (50 mg, 78%) and a mixture of diastereomers (8 mg, 12%); Data for pure *cis*-product: mp: 116-118 °C; $[\alpha]_D^{20}$ +1.53 (*c* 1.0, $CHCl_3$); Chiral HPLC analysis, Chiralpak OD-H (95:5 hexane:IPA, flow rate 1.00 mLmin^{-1}, 254 nm, 40 °C) t_R (4a*R*,10b*S*): 28.2 min, t_R (4a*S*,10b*R*): 22.6 min, 97:3 er; IR ν_{max} (film): 2929 (C-H), 1763 (C=O); ^1H NMR (400 MHz, $CDCl_3$) δ_H: 2.29 (3H, s, CH_3), 3.15-3.24 (1H, m, C(4a)*H*), 3.82 (3H, s, OCH_3), 3.94-4.01 (2H, m, OC(5)H^AH^B & C(10b)*H*), 4.19 (1H, ddd, *J* 11.2, 3.0, 1.2, OC(5)H^AH^B), 5.65 (1H, d, *J* 4.9, C(4)*H*), 6.75 (1H, d, *J* 8.3, ArC(7)*H*), 6.85-6.91 (2H, m, ArC(3',5')*H*), 6.98-7.01 (1H, ddt, *J* 8.3, 2.2, 0.7, ArC(8)*H*), 7.11 (1H, m, ArC(10)*H*), 7.52-7.58 (2H, m, ArC(2',6')*H*); $^{13}C\{^1H\}$ NMR (101 MHz, $CDCl_3$) δ_C: 20.7(*C*H_3), 31.0 (*C*(4a)H), 40.1 (*C*(10b)H), 55.5 (O*C*H_3), 66.1 (O*C*H_2), 96.8 (*C*(4)H), 114.0 (Ar*C*(2',6')H), 115.0 (Ar*C*(10a)), 117.0 (Ar*C*(7)H), 124.4 (Ar*C*(9)), 126.3 (Ar*C*(2',6')H), 130.2 (Ar*C*(8)H), 130.4 (Ar*C*(1')), 131.2 (Ar*C*(10)H), 151.4 (*C*(3)), 151.7

(Ar*C*(6a)), 160.6 (Ar*C*(4')), 168.0 (*C*=O); m/z (NSI) $C_{20}H_{19}O_4$ ([M+H]$^+$, 100%) found 323.1277, requires 323.1278 (−0.3 ppm).

(4a*R*,10b*S*)-8-Methoxy-3-(4-methoxyphenyl)-4a,10b-dihydropyrano[4,3-*c*]chromen-1(5*H*)-one (21)

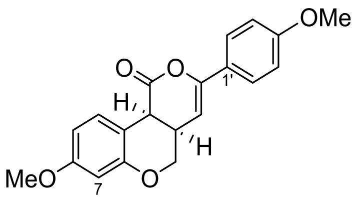

Following general procedure C: (*E*)-2-(4-Methoxy-2-((4-(4-methoxyphenyl)-4-oxobut-2-en-1-yl)oxy)phenyl)acetic acid (53.4 mg, 0.15 mmol, 1.0 equiv), $CHCl_3$ (1.5 mL), (*i*-Pr)$_2$NEt (39 ul, 0.23 mmol, 1.5 equiv), pivaloyl chloride (46 ul, 0.38 mmol, 2.5 equiv), followed by TM·HCl (1.8 mg, 5 mol%), (*i*-Pr)$_2$NEt (65 ul, 0.38 mmol, 2.5 equiv), 0.1 M aqu.HCl (2 × 1.8 mL), to give (4a*R*,10b*S*)-8-methoxy-3-(4-methoxyphenyl)-4a,10b-dihydropyrano[4,3-*c*]chromen-1(5*H*)-one **21** as a colourless solid (40 mg, 79%) and a mixture of diastereomers (6 mg, 12%); Data for pure *cis*-product: mp: 118-120 °C; $[\alpha]_D^{20}$ +0.83 (*c* 1.0, $CHCl_3$); Chiral HPLC analysis, Chiralpak OD-H (95:5 hexane:IPA, flow rate 1.00 mLmin^{-1}, 220 nm, 40 °C) t_R (4a*R*,10b*S*): 45.5 min, t_R (4a*S*,10b*R*): 39.8 min, 97:3 er; IR ν_{max} (film): 2936 (C-H), 1767 (C=O); ^{1}H NMR (400 MHz, $CDCl_3$) δ_H: 3.15-3.24 (1H, m, C(4a)*H*), 3.76 (3H, s, OC*H*$_3$), 3.82 (3H, s, OC*H*$_3$), 3.91-4.00 (2H, m, OC(5)H^AH^B & C(10b)*H*), 4.20 (1H, ddd, *J* 11.3, 3.1, 1.3, OC(5)H^AH^B), 5.64 (1H, *J* 5.1, C(4)*H*), 6.39 (2H, d, *J* 2.6, ArC(7)*H*), 6.57 (1H, dd, *J* 8.6, 2.6, ArC(9)*H*), 6.85-6.92 (2H, m, ArC(3',5')*H*), 7.21 (1H, dd, *J* 8.6, 0.8, ArC(10)*H*), 7.51-7.59 (2H, m, ArC(2',6')*H*); ^{13}C{^{1}H} NMR (101 MHz, $CDCl_3$) δ_C: 31.0 (*C*(4a)H), 39.5 (*C*(10b)H), 55.4 (O*C*H$_3$), 55.5 (O*C*H$_3$), 66.2 (O*C*H$_2$), 96.6 (*C*(4)H), 101.9 (Ar*C*(7)H), 107.7 (Ar*C*(10a)), 108.3 (Ar*C*(9)H), 114.0 (Ar*C*(3',5')H), 124.4 (Ar*C*(1')), 126.3 (Ar*C*(2',6')H), 131.9 (Ar*C*(10)H), 151.5 (*C*(3)), 154.8 (Ar*C*(6a)), 160.5 (Ar*C*(4')), 160.7 (Ar*C*(8)), 168.3 (*C*=O); m/z (NSI) $C_{20}H_{19}O_5$ ([M+H]$^+$, 100%) found 339.1229, requires 339.1227 (+0.6 ppm).

(4a*S*,12a*R*)-2-(4-Methoxyphenyl)-12,12a-dihydrobenzo[*h*]pyrano[4,3-*c*]chromen-4(4a*H*)-one (22)

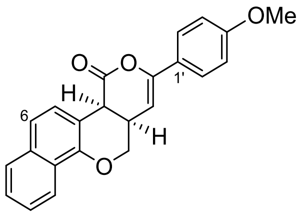

Following general procedure C: (*E*)-2-(1-((4-(4-Methoxyphenyl)-4-oxobut-2-en-1-yl)oxy)naphthalen-2-yl)acetic acid (75 mg, 0.20 mmol, 1.0 equiv), $CHCl_3$ (2.0 mL), (i-Pr)$_2$NEt (52 µL, 0.30 mmol, 1.5 equiv), pivaloyl chloride (62 µL, 0.50 mmol, 2.5 equiv), followed by TM·HCl (2.4 mg, 5 mol%), (i-Pr)$_2$NEt (87 µL, 0.50 mmol, 2.5 equiv), 0.1 M aqu.HCl (2 × 2.4 mL), to give (4a*S*,12a*R*)-2-(4-methoxyphenyl)-12,12a-dihydrobenzo[*h*]pyrano[4,3-*c*]chromen-4(4a*H*)-one **22** as a pale yellow solid (56 mg, 78%) and a mixture of diastereomers (8 mg, 11%); Data for pure *cis*-product: mp: 178-180 °C; $[\alpha]_D^{20}$ +0.67 (*c* 1.0, $CHCl_3$); Chiral HPLC analysis, Chiralpak AD-H (95:5 hexane:IPA, flow rate 1.50 mLmin^{-1}, 270 nm, 40 °C) t_R (4a*S*,12a*R*): 38.4 min, t_R (4a*R*,12a*S*): 23.7 min, 97:3 er; IR ν_{max} (film): 2924 (C-H), 1768 (C=O); ^{1}H NMR (400 MHz, $CDCl_3$) δ_H: 3.23-3.33 (1H, m, C(12a)*H*), 3.82 (3H, s, OCH_3) 4.06-4.18 (2H, m, OC(12)H^AH^B & C(4a)*H*), 4.43 (1H, ddd, *J* 11.2, 3.2, 1.4, OC(12)H^AH^B), 5.70 (1H, d, *J* 5.3, C(1)*H*), 6.86-6.92 (2H, m, ArC(3',5')*H*), 7.37-7.43 (1H, m, ArC*H*), 7.43-7.52 (3H, m, 3×ArC*H*), 7.54-7.61 (2H, m, ArC(2',6')*H*), 7.74-7.83 (1H, m, ArC*H*), 8.14-8.22 (1H, m, ArC*H*); ^{13}C{^{1}H} NMR (101 MHz, $CDCl_3$) δ_C: 31.0 (*C*(12a)H), 40.2 (*C*(4a)H), 55.5 (O*C*H_3), 66.4 (O*C*H_2), 96.4 (*C*(1)H), 109.5 (Ar*C*(4b)), 114.0 (Ar*C*(3',5')H), 120.5 (Ar*C*H), 121.8 (Ar*C*H), 124.4 (Ar*C*(1')), 125.0 (Ar*C*(10a)), 125.7 (Ar*C*H), 126.3 (Ar*C*(2',6')H), 126.8 (Ar*C*H), 127.6 (Ar*C*H), 128.2 (Ar*C*H), 134.1 (Ar*C*(6a)), 149.5 (*C*(2)), 151.6 (Ar*C*(10b)), 160.7 (Ar*C*(4')), 168.1 (*C*=O); m/z (NSI) $C_{23}H_{19}O_4$ ([M+H]$^+$, 100%) found 359.1280, requires 359.1278 (+0.6 ppm).

Derivatisation

Methyl (3*R*,4*S*)-3-(2-oxo-2-phenylethyl)chromane-4-carboxylate (23)

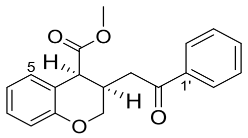

A solution of (4a*R*, 10b*S*)-3-phenyl-4a,10b-dihydropyrano[4,3-*c*]chromen-1(5*H*)-one **2** (56 mg, 0.20 mmol, 1.0 equiv) and DMAP (2.4 mg, 10 mol%) in methanol (2 mL) was stirred for 2 h at r.t. Upon completion of the reaction, the solvent was removed under reduced pressure, and the residue purified by Biotage® Isolera™ 4 [SNAP Ultra 25 g, 75 mL min^{-1}, petroleum ether:EtOAc (100:0 2 CV, 100:0 to 90:10 40 CV)] to give methyl (3*R*,4*S*)-3-(2-oxo-2-phenylethyl)chromane-4-carboxylate **23** as a colourless solid (58 mg, 93 %); mp: 70-72 °C; $[\alpha]_D^{20}$ −0.80 (*c* 1.0, $CHCl_3$); Chiral HPLC analysis, Chiralpak AD-H (95:5 hexane:IPA, flow rate 1.00 $mLmin^{-1}$, 254 nm, 40 °C) t_R (3*R*,4*S*): 13.5 min, t_R (3*S*,4*R*): 14.9 min, 98:2 er; IR ν_{max} (film): 2951 (C-H), 1722 ($CH_3OC{=}O$), 1680 (C=O); ^1H NMR (400 MHz, $CDCl_3$) δ_H: 2.95-3.10 (2H, m, CH^AH^BCOAr & C(3)*H*), 3.14-3.25 (1H, m, CH^AH^BCOAr), 3.65 (3H, s, OCH_3), 4.07 (1H, d, *J* 5.2, C(4)*H*), 4.21 (1H, ddd, *J* 10.8, 3.6, 1.6, OC(2)H^AH^B), 4.36-4.44 (1H, m, OC(2)H^AH^B), 6.84-6.92 (2H, m, ArC(6,8)*H*), 7.13-7.22 (2H, m, ArC(5,7)*H*), 7.43-7.52 (2H, m, ArC(3',5')*H*), 7.55-7.62 (1H, m, ArC(4')*H*), 7.92-8.00 (2H, m, ArC(2'6')*H*); $^{13}C\{^1H\}$ NMR (101 MHz, $CDCl_3$) δ_C: 30.3 (*C*(3)H), 37.2 (CH_2COAr), 43.9 (*C*(4)H), 52.2 (OCH_3), 66.8 (*C*(2)H_2), 117.4 (Ar*C*(8)H), 118.5 (Ar*C*(4a)), 120.6 (Ar*C*(6)H), 128.2 (Ar*C*(3',5')H), 128.8 (Ar*C*(2',6')H), 129.0 (Ar*C*(7)H), 130.1 (Ar*C*(5)H), 133.5 (Ar*C*(4')H), 136.8 (Ar*C*(1')), 154.2 (Ar*C*(8a)), 173.2 ($O{=}COCH_3$), 197.6 (*C*=O); m/z (NSI) $C_{19}H_{19}O_4$ ($[M+H]^+$, 100%) found 311.1277, requires 311.1278 (−0.3 ppm).

2-((3*R*,4*S*)-4-(Morpholine-4-carbonyl)chroman-3-yl)-1-phenylethan-1-one (24)

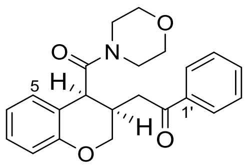

To a solution of (4a*R*, 10b*S*)-3-phenyl-4a,10b-dihydropyrano[4,3-*c*]chromen-1(5*H*)-one **2** (28mg, 0.10 mmol, 1.0 equiv) in $CHCl_3$ (1 mL) was added morpholine (26 µL, 0.3 mmol, 3.0

equiv) and the reaction stirred for 8 h at r.t. Upon completion, the solvent was removed under reduced pressure and the residue purified by Biotage® Isolera™ 4 [SNAP Ultra 25 g, 75 mL min^{-1}, hexane:EtOAc (100:0 2 CV, 100:0 to 70:30 40 CV)] to give 2-((3*R*,4*S*)-4-(morpholine-4-carbonyl)chroman-3-yl)-1-phenylethan-1-one **24** as a pale yellow solid (30 mg, 82 %); mp: 136-138 °C; $[\alpha]_D^{20}$ −1.48 (*c* 1.0, $CHCl_3$); Chiral HPLC analysis, Chiralpak IB (93:7 hexane:IPA, flow rate 1.00 mLmin^{-1}, 220 nm, 40 °C) t_R (3*R*,4*S*): 35.8 min, t_R (3*S*,4*R*): 22.6 min, 98:2 er; IR ν_{max} (film): 2922 (C-H), 1681 (C=O), 1633 (NHC=O); ^{1}H NMR (400 MHz, $CDCl_3$) δ_H: 2.90-3.02 (2H, m, CH^AH^BCO & C(3)*H*), 3.25 (1H, dd, *J* 19.0, 10.4, CH^AH^BCO), 3.30-3.76 (8H, m, 2×NCH_2 & 2×OCH_2), 4.12 (1H, dd, *J* 10.5, 3.4, C(2)H^AH^B), 4.57 (1H, d, *J* 5.6, C(4)*H*), 4.65 (1H, dd, *J* 10.5, 9.4, C(2)H^AH^B), 6.82-6.95 (3H, m, ArC(5,6,8)*H*), 7.15 (1H, ddd, *J* 8.4, 6.9, 1.9, ArC(7)*H*), 7.47 (2H, dd, *J* 8.4, 7.1, ArC(3',5')*H*), 7.56-7.63 (1H, m, ArC(4')*H*), 7.92-7.99 (2H, m, ArC(2',6')*H*); ^{13}C{^{1}H} NMR (101 MHz, $CDCl_3$) δ_C: 30.7 (*C*(3)H), 37.1 (*C*H_2COAr), 37.4 (*C*(4)H), 42.3 (N*C*H_2), 47.2 (N*C*H_2), 66.8 (O*C*H_2), 67.0 (O*C*H_2), 67.4 (*C*(2)H_2), 117.6 (Ar*C*(8)H), 120.1 (Ar*C*(4a)), 120.7 (Ar*C*(6)H), 128.1 (Ar*C*(3',5')H), 128.5 (Ar*C*(5)H), 128.9 (Ar*C*(2',6')H), 129.2 (Ar*C*(7)H), 133.7 (Ar*C*(4')H), 136.6 (Ar*C*(1')), 154.8 (Ar*C*(8a)), 171.6 (HN*C*=O), 198.4 (*C*=O); m/z (NSI) $C_{22}H_{24}O_4N$ ([M+H]$^+$, 100%) found 366.1703, requires 366.1705 (−0.5 ppm).

(3*R*,4*S*)-*N*-Benzyl-3-(2-oxo-2-phenylethyl)chromane-4-carboxamide (25)

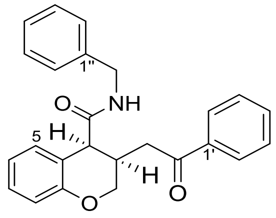

To a solution of (4a*R*, 10b*S*)-3-phenyl-4a,10b-dihydropyrano[4,3-*c*]chromen-1(5*H*)-one **2** (56 mg, 0.10 mmol, 1.0 equiv) in $CHCl_3$ (2 mL) was added benzylamine (66 µL, 0.60 mmol, 3.0 equiv). Stirred for 4.0 h at r.t. Upon completion, the solvent was under reduced pressure and the residue was purified by Biotage® Isolera™ 4 [SNAP Ultra 25 g, 75 mL min−1, petroleum ether:EtOAc (100:0 2 CV, 100:0 to 70:30 40 CV)] to afford (3*R*,4*S*)-*N*-benzyl-3-(2-oxo-2-phenylethyl)chromane-4-carboxamide **25** as a pale yellow solid (71 mg, 92 %); mp: 96-98 °C; $[\alpha]_D^{20}$ −1.08 (*c* 1.0, $CHCl_3$); Chiral HPLC analysis, Chiralpak AD-H (90:10 hexane:IPA, flow rate

1.00 mLmin^{-1}, 254 nm, 40 °C) t_R (3*R*,4*S*): 28.3 min, t_R (3*S*,4*R*): 32.5 min, 98:2 er; IR ν_{max} (film): 3269 (N-H), 2924 (C-H), 1685 (C=O), 1635 (NHC=O); ^{1}H NMR (400 MHz, $CDCl_3$) δ_H: 2.89-3.01 (2H, m, CH^AH^BCO & C(3)*H*), 3.24-3.36 (1H, m, CH^AH^BCO), 3.93 (1H, d, *J* 5.2, C(4)*H*), 4.17 (1H, ddd, *J* 10.6, 3.6, 1.4, C(2)H^AH^B), 4.27-4.42 (3H, m, NCH_2 & C(2)H^AH^B), 6.17 (1H, t, *J* 5.9, N*H*), 6.84-6.92 (2H, m, ArC(6,8)*H*), 7.03-7.13 (3H, m, ArC(7)*H* & ArC(2'',6'')*H*), 7.13-7.23 (4H, m, ArC(5)*H* & ArC(3'',4'',5'')*H*), 7.46 (2H, m, ArC(3',5')*H*), 7.55-7.62 (1H, m, ArC(4')*H*), 7.91 (2H, m, ArC(2',6')*H*); ^{13}C{^{1}H} NMR (101 MHz, $CDCl_3$) δ_C : 30.9 (*C*(3)H), 37.3 (*C*H_2COAr), 43.7 (N*C*H_2), 45.4 (*C*(4)H), 66.9 (*C*(2)H_2), 117.5 (Ar*C*(8)H), 119.4 (Ar*C*(4a)), 120.8 (Ar*C*(6)H), 127.5 (Ar*C*(4'')H), 127.6 (Ar*C*(3'',5'')H), 128.2 (Ar*C*(2'',6'')H), 128.7 (Ar*C*(3',5')H), 128.8 (Ar*C*(2',6')H), 129.1 (Ar*C*(7)H), 129.9 (Ar*C*(5)H), 133.5 (Ar*C*(4')H), 136.7 (Ar*C*(1'')), 138.0 (Ar*C*(1')), 154.5 (Ar*C*(8a)), 172.1 (NH*C*=O), 198.4 (*C*=O); m/z (NSI) $C_{25}H_{24}O_3N$ ([M+H]$^+$, 100%) found 386.1750, requires 386.1751 (−0.2 ppm).

(3*R*,4*S*)-3-Phenethylchromane-4-carboxylic acid (26)

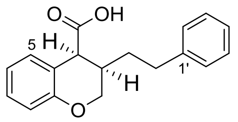

To a solution of (4a*R*, 10b*S*)-3-phenyl-4a,10b-dihydropyrano[4,3-*c*]chromen-1(5*H*)-one **2** (28 mg, 0.1 mmol, 1.0 equiv) in EtOAc (1.0 mL) was added 10% Pd on carbon (2.8 mg, 10% w/w). Stirred at 1.0 bar hydrogen pressure (baloon pressure) for 4 h at r.t. Upon completion the solution was filtered through celite. The filtrate was concentrated under reduced pressure to give (3*R*,4*S*)-3-phenethylchromane-4-carboxylic acid **26** as a colourless solid (26 mg, 92 %); mp: 84-86 °C; $[\alpha]_D^{20}$ −1.76 (*c 1.0*, $CHCl_3$); Chiral HPLC analysis, Chiralpak AD-H (97:3 hexane:IPA, flow rate 1.00 mLmin^{-1}, 211 nm, 30 °C) t_R (3*R*,4*S*): 25.3 min, t_R (3*S*,4*R*): 20.6 min, 98:2 er; IR ν_{max} (film): 3034 (O-H), 2943 (C-H), 1724 (C=O); ^{1}H NMR (400 MHz, $CDCl_3$) δ_H: 1.70-1.90 (2H, m, C(3)C*H*$_2$), 2.16-2.30 (1H, m, C(3)*H*), 2.70-2.94 (2H, m, C(1')C*H*$_2$), 3.88 (1H, d, *J* 5.4, C(4)*H*), 4.19 (1H, ddd, *J* 11.0, 4.0, 1.4, C(2)H^AH^B), 4.38 (1H, app t, *J* 11.0, C(2)H^AH^B), 6.90 (2H, m, ArC(6,8)*H*), 7.19-7.27 (5H, m, ArC(2',3',4',5',6')*H*), 7.28-7.35 (2H, m, ArC(5,7)*H*); ^{13}C{^{1}H} NMR (101 MHz, $CDCl_3$) δ_C: 30.5 (C(3)*C*H_2), 33.2 (C(1')*C*H_2), 33.7 (*C*(3)H), 44.3 (*C*(4)H), 66.2 (*C*(2)H_2), 117.4 (Ar*C*(8)H), 118.2 (Ar*C*(4a)), 120.4 (Ar*C*(6)H), 126.3 (Ar*C*(7)H), 128.4 (Ar*C*(2',6')H), 128.7 (Ar*C*(3',5')H), 129.3 (Ar*C*(4')H), 130.0 (Ar*C*(5)H), 141.2 (Ar*C*(1')), 154.4

(Ar*C*(8a)), 179.1 (*C*=O); m/z (NSI) $C_{18}H_{17}O_3$ ($[M-H]^-$, 100%) found 281.1184, requires 281.1183 (+0.3 ppm).

(3*R*,3a*R*,9b*S*)-3-Benzoyl-3a,9b-dihydro-3*H*-furo[3,4-*c*]chromen-1(4*H*)-one (27)

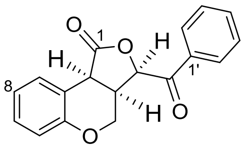

To a solution of (4a*R*, 10b*S*)-3-phenyl-4a,10b-dihydropyrano[4,3-*c*]chromen-1(5*H*)-one **2** (28 mg, 0.10 mmol, 1.0 equiv) in CH_2Cl_2 (1.0 mL) at 0 °C was added *m*-CPBA (33.6 mg, 77% purity, 0.15 mmol, 1.5 equiv) and the mixture stirred for 16 h at 0 °C. Upon completion, *p*-TSA (1.9 mg, 0.01 mmol, 0.1 equiv) was added and the reaction stirred for another 20 min at r.t. Aqueous saturated $NaHCO_3$ (1.0 mL) solution was added and the aqueous layer extracted with CH_2Cl_2 (2 × 2 mL). The combined organic layers were washed with brine, dried ($MgSO_4$), filtered and concentrated under reduced pressure. The residue was washed with 5% EtOAc in pentane to afford pure product as a colourless solid (24 mg, 82 %); mp: 152-154 °C ; $[\alpha]_D^{20}$ +0.95 (*c* 1.0, $CHCl_3$); Chiral HPLC analysis, Chiralpak AD-H (90:10 hexane:IPA, flow rate 1.00 mLmin^{-1}, 254 nm, 40 °C) t_R (3*R*,3a*R*,9b*S*): 38.2 min, t_R (3*S*,3a*S*,9b*R*): 53.4 min, 100:0 er; IR ν_{max} (film): 2962 (C-H), 1780 (O=COCH), 1701 (C=O); ^{1}H NMR (400 MHz, $CDCl_3$) δ_H: 3.41-3.50 (1H, m, C(3a)*H*), 3.62 (1H, app t, *J* 11.4, C(4)H^AH^B), 3.93 (1H, d, *J* 7.5, C(9b)*H*), 4.02 (1H, ddd, *J* 11.4, 4.6, 1.4, C(4)H^AH^B), 5.97 (1H, d, *J* 6.3, C(3)*H*), 6.83 (1H, dd, *J* 8.2, 1.2, ArC(6)*H*), 7.03 (1H, app td, *J* 7.5, 1.3, ArC(8)*H*), 7.20 (1H, m, ArC(7)*H*), 7.51-7.59 (3H, m, ArC(9)*H* & ArC(3',5')*H*), 7.65-7.72 (1H, m, ArC(4')*H*), 7.98-8.04 (2H, m, ArC(2',6')*H*); $^{13}C\{^1H\}$ NMR (101 MHz, $CDCl_3$) δ_C: 36.4 (*C*(3a)H), 40.2 (*C*(9b)H), 62.2 (*C*(4)H_2), 79.3 (O*C*(3)H), 115.5 (Ar*C*(9a)), 117.1 (Ar*C*(6)H), 122.1 (Ar*C*(8)H), 128.4 (Ar*C*(3',5')H), 129.1 (Ar*C*(7)H), 129.4 (Ar*C*(2',6')H), 130.6 (Ar*C*(9)H), 134.5 (Ar*C*(1')), 134.8 (Ar*C*(4')H), 154.2 (Ar*C*(5a)), 173.8 (O=*C*OCH), 192.2 (*C*=O); m/z (NSI) $C_{18}H_{15}O_4$ ($[M+H]^+$, 100%) found 295.0966, requires 295.0965 (+0.4 ppm).

References :

1. M. M. Alam and S. R Adapa, *Synthetic Communications,* 2003, **33**, 59.
2. S. Venkateswarlu, G. K. Panchagnula, M. B. Guraiah, and G. V. Subbaraju, *Tetrahedron,* 2006, **62**, 9855.
3. (a) S. Venkateswarlu, G. K. Panchagnula, M. B. Guraiah and G. V. Subbaraju, *Tetrahedron,* 2005, **61**, 3013-3017; (b) J. N. Chatterjea, *J. Indian Chem. Soc*., 1957, **34**, 279.
4. M. Kitamura, K. Araki, H. Matsuzaki and T. Okauchi, *Eur. J. Org. Chem*., 2013, **23**, 5045.
5. S. P. Breukelman, G.D. Meakins and A. M. Roe, *J. Chem. Soc., Perkin Trans. 1: Organic and Bio-Organic Chemistry*. 1985, 1627.
6. J. Lin, B. S. Gerstenberger, N. Y. T. Stessman and J. P. Konopelski, *Org. Lett*., 2008, **10**, 3969.
7. D. Belmessieri, L. C. Morrill, C. Simal, A. M. Z. Slawin and A. D. Smith, *J. Am. Chem. Soc*., 2011, **133**, 2714.
8. D. G. Stark, L. C. Morril, P.-P. Yeh, A. M. Z. Slawin, T. J. C. O'Riordan and A. D. Smith, *Angew. Chem. Int. Ed*., 2013, **52**, 11642.
9. D. Belmessieri, D. B. Cordes, A. M. Z. Slawin and A. D. Smith, *Org. Lett*., 2013, **15**, 3472.
10. E. Venkateswararao, M.-S. Kim, V. K. Sharma, K.-C. Lee, S. Subramanian, E. Roh, Y. Kim and S.-H. Jung, *Eur. J. Med. Chem*., 2013, **59**, 31.

^{1}H and $^{13}C\{H\}$ NMR and chiral HPLC datas

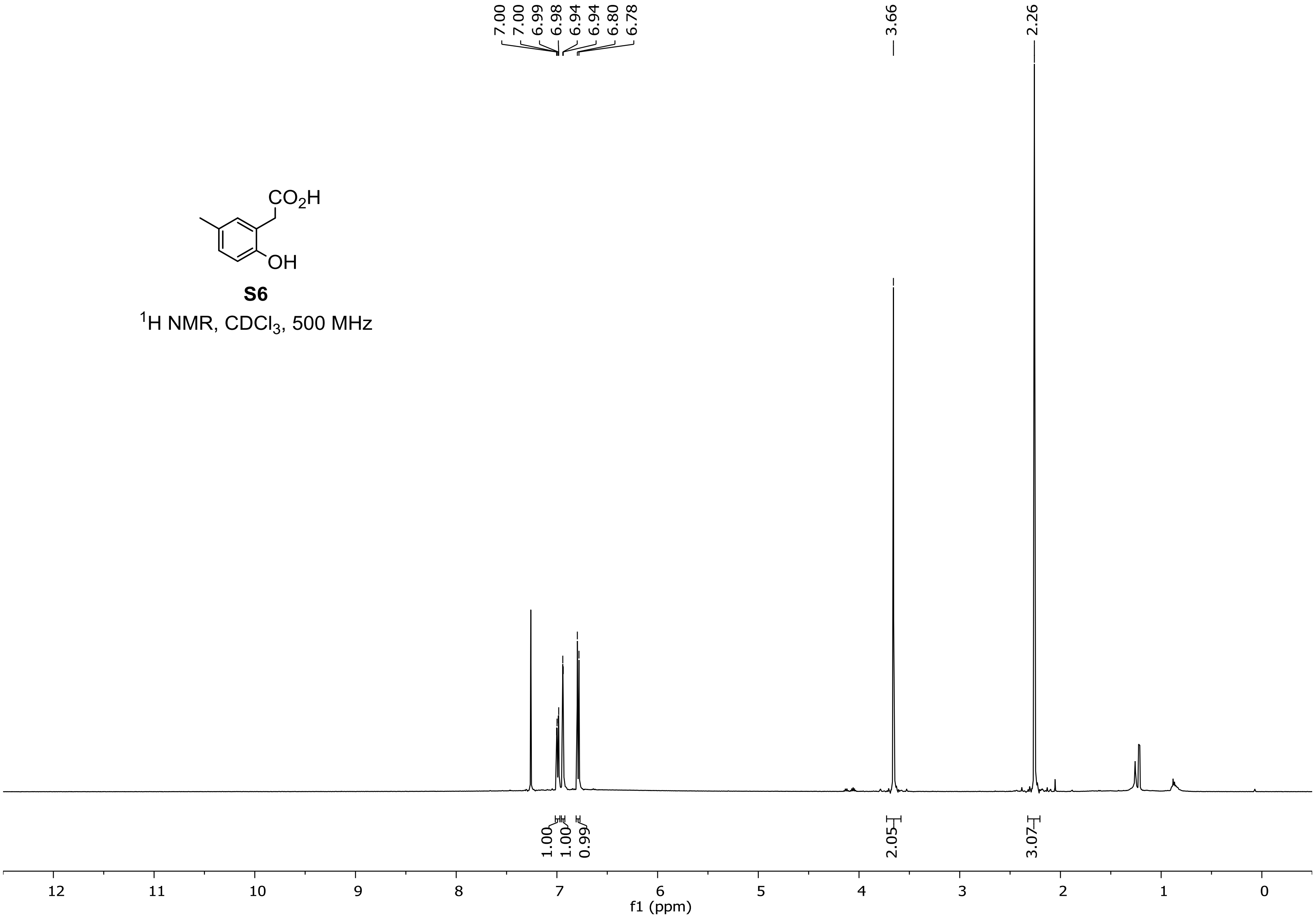

CO2H
OH
S6
1H NMR, CDCl3, 500 MHz
7.00
7.00
6.99
6.98
6.94
6.94
6.80
6.78
3.66
2.26
1.00
1.00
0.99
2.05
3.07
12
11
10
9
8
7
6
5
4
3
2
1
0
f1 (ppm)

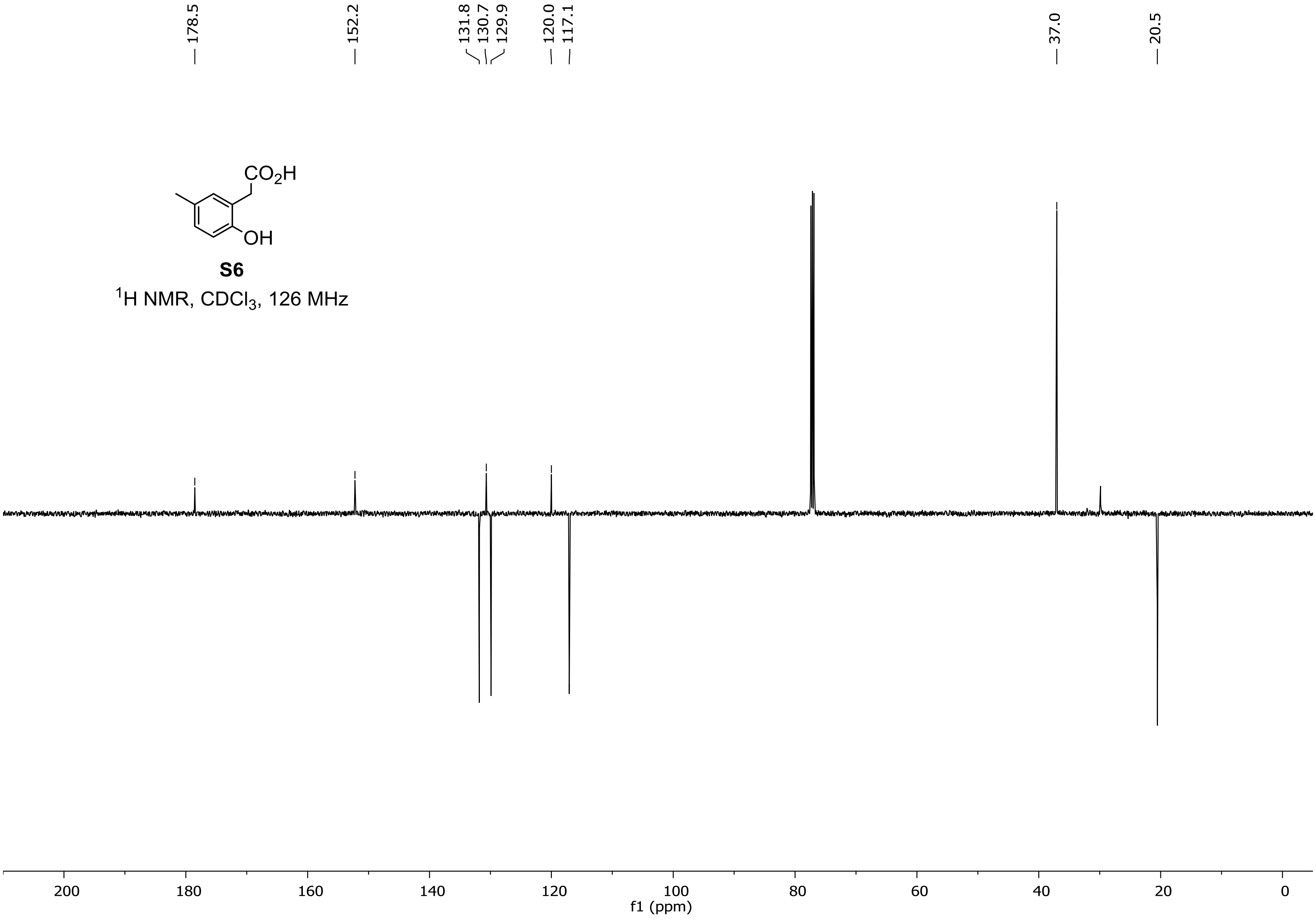

S6

^{1}H NMR, $CDCl_3$, 126 MHz

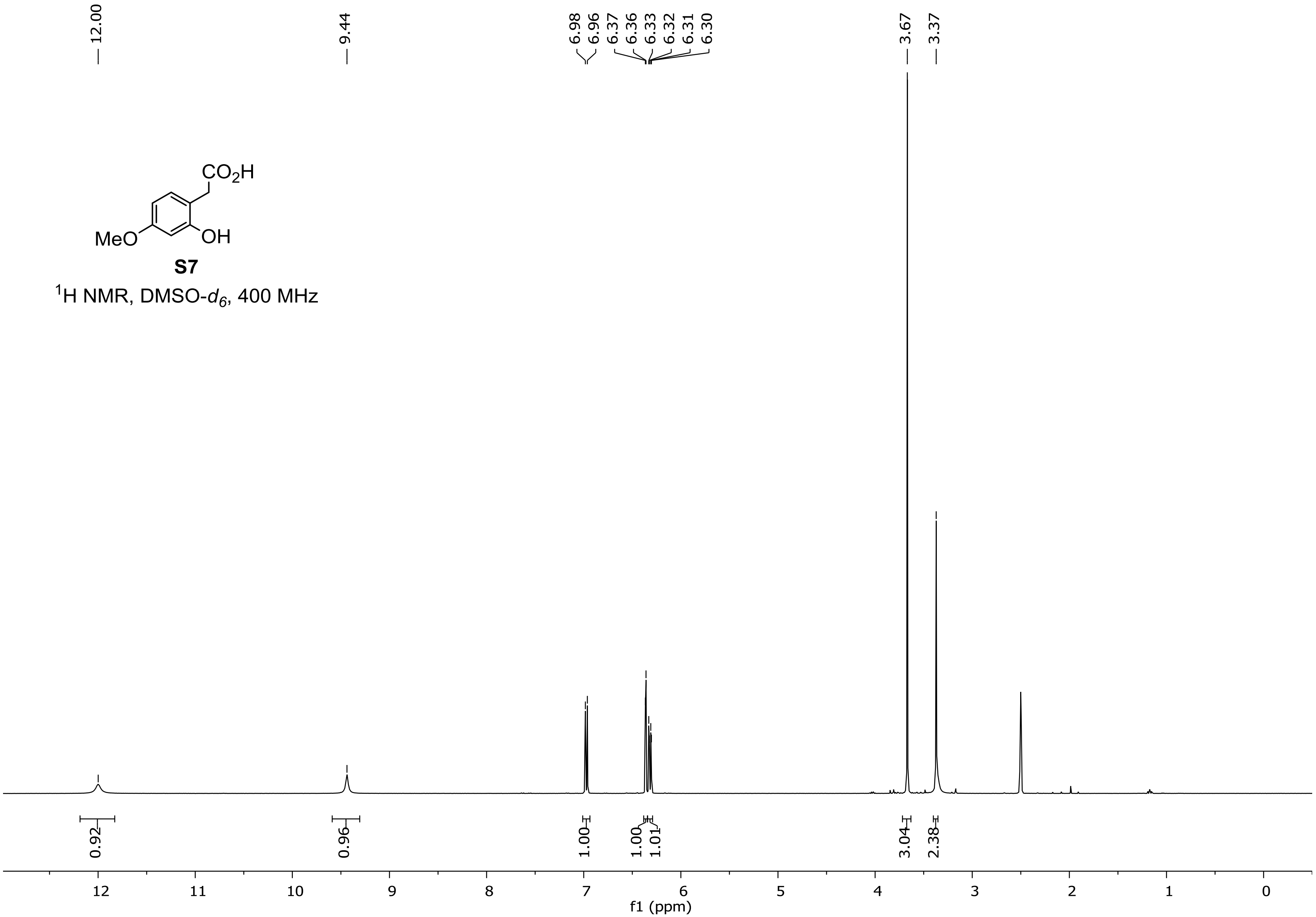
12.00
9.44
6.98
6.96
6.37
6.36
6.33
6.32
6.31
6.30
3.67
3.37
CO2H
MeO
OH
S7
1H NMR, DMSO-d6, 400 MHz
0.92
0.96
1.00
1.00
1.01
3.04
2.38
12
11
10
9
8
7
6
5
4
3
2
1
0
f1 (ppm)

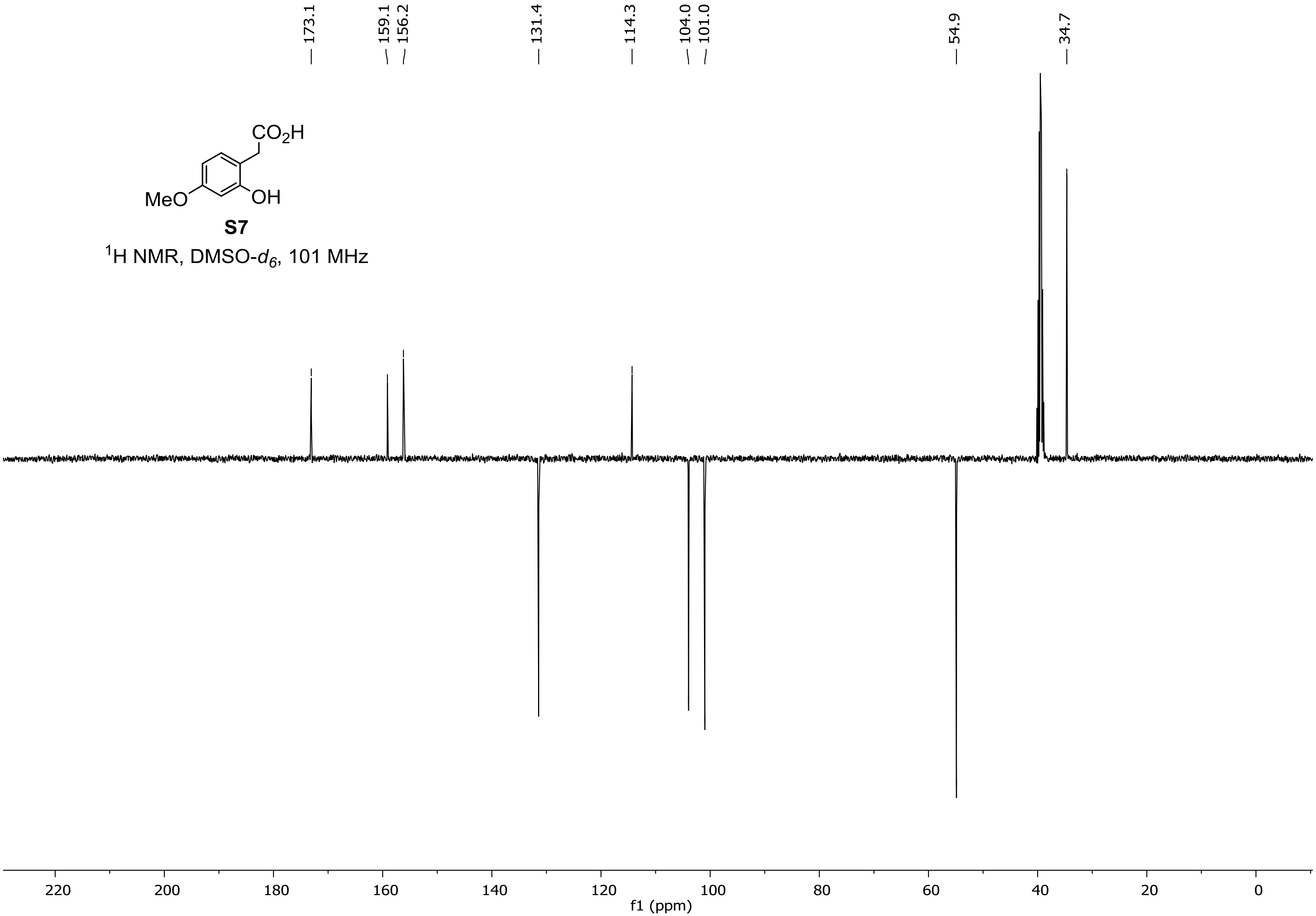
173.1
159.1
156.2
131.4
114.3
104.0
101.0
54.9
34.7
CO2H
MeO
OH
S7
1H NMR, DMSO-d6, 101 MHz
220
200
180
160
140
120
100
80
60
40
20
0
f1 (ppm)

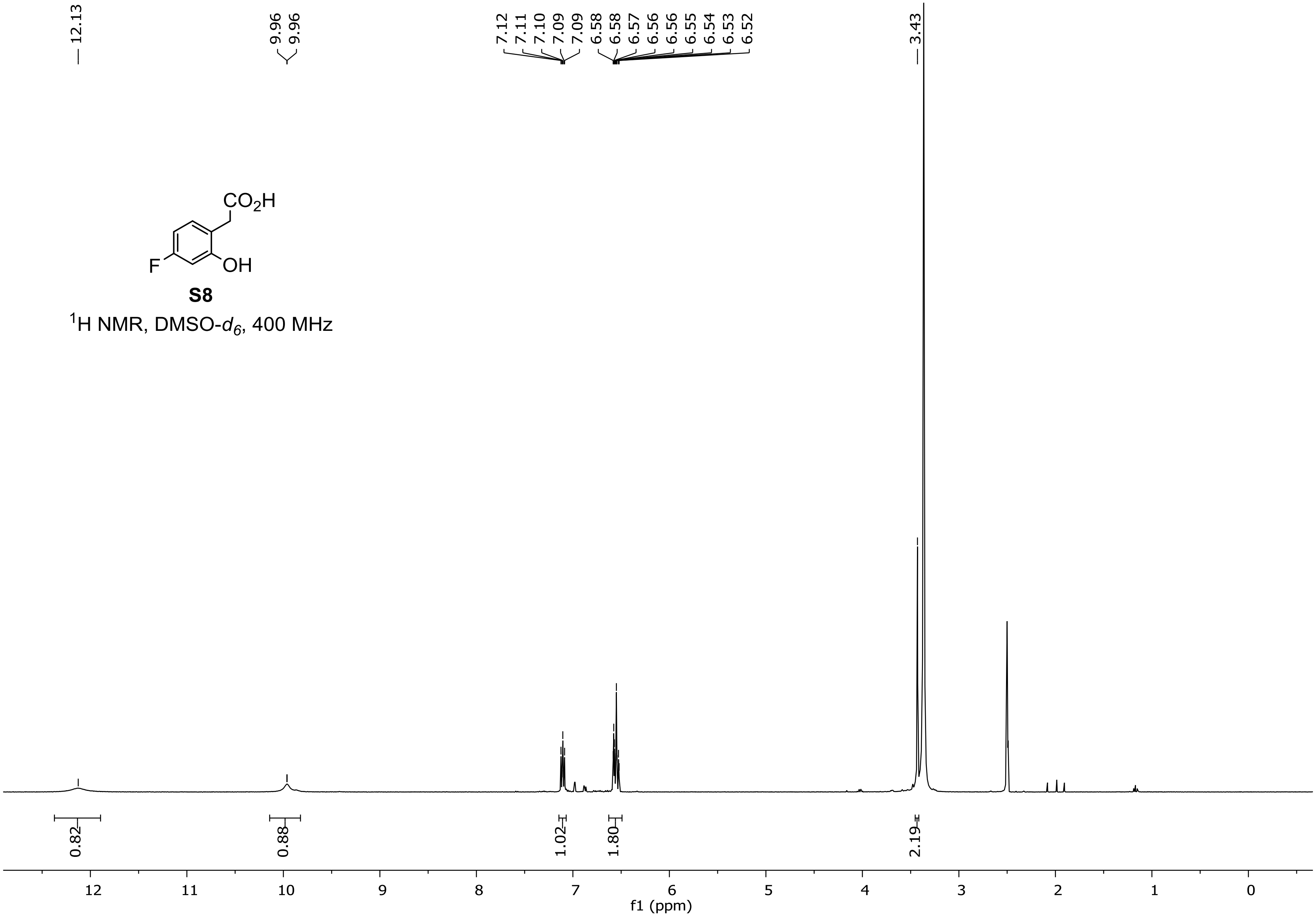
12.13
9.96
9.96
7.12
7.11
7.10
7.09
7.09
6.58
6.58
6.57
6.56
6.56
6.55
6.54
6.53
6.52
3.43
CO2H
F
OH
S8
1H NMR, DMSO-d6, 400 MHz
0.82
0.88
1.02
1.80
2.19
12
11
10
9
8
7
6
5
4
3
2
1
0
f1 (ppm)

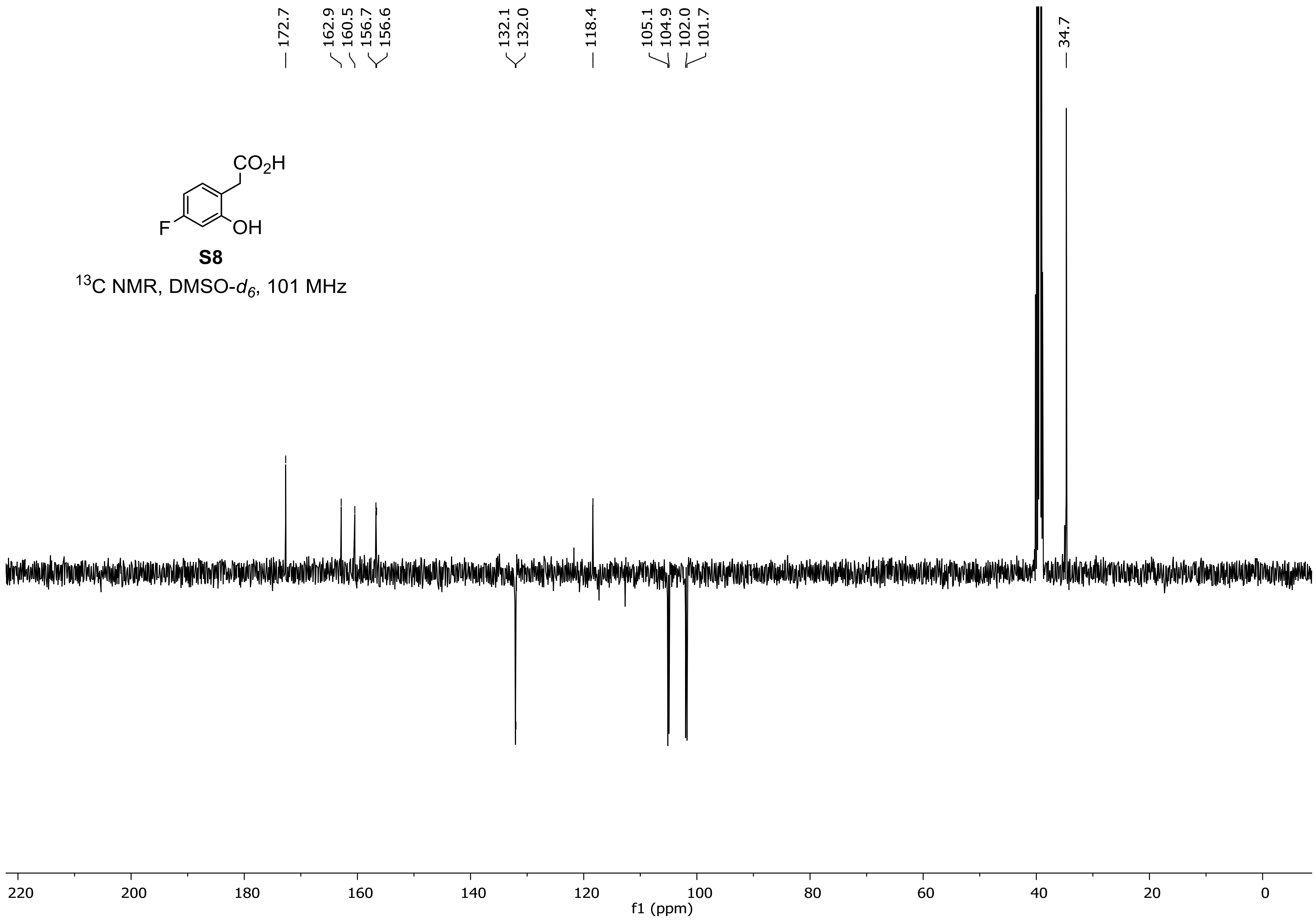
172.7
162.9
160.5
156.7
156.6
132.1
132.0
118.4
105.1
104.9
102.0
101.7
34.7
CO2H
F
OH
S8
13C NMR, DMSO-d6, 101 MHz
220
200
180
160
140
120
100
80
60
40
20
0
f1 (ppm)

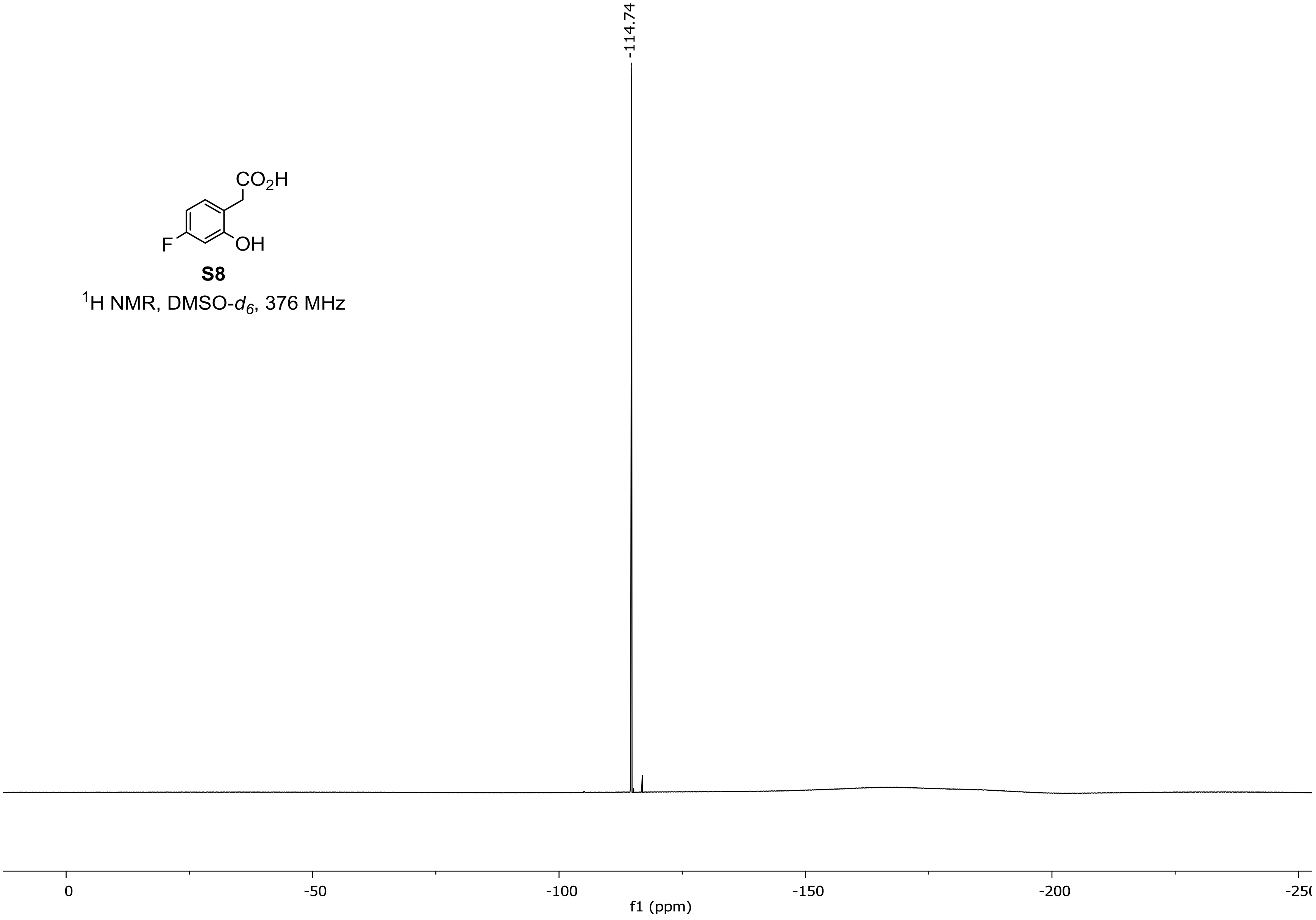

S8

^{1}H NMR, DMSO-d_6, 376 MHz

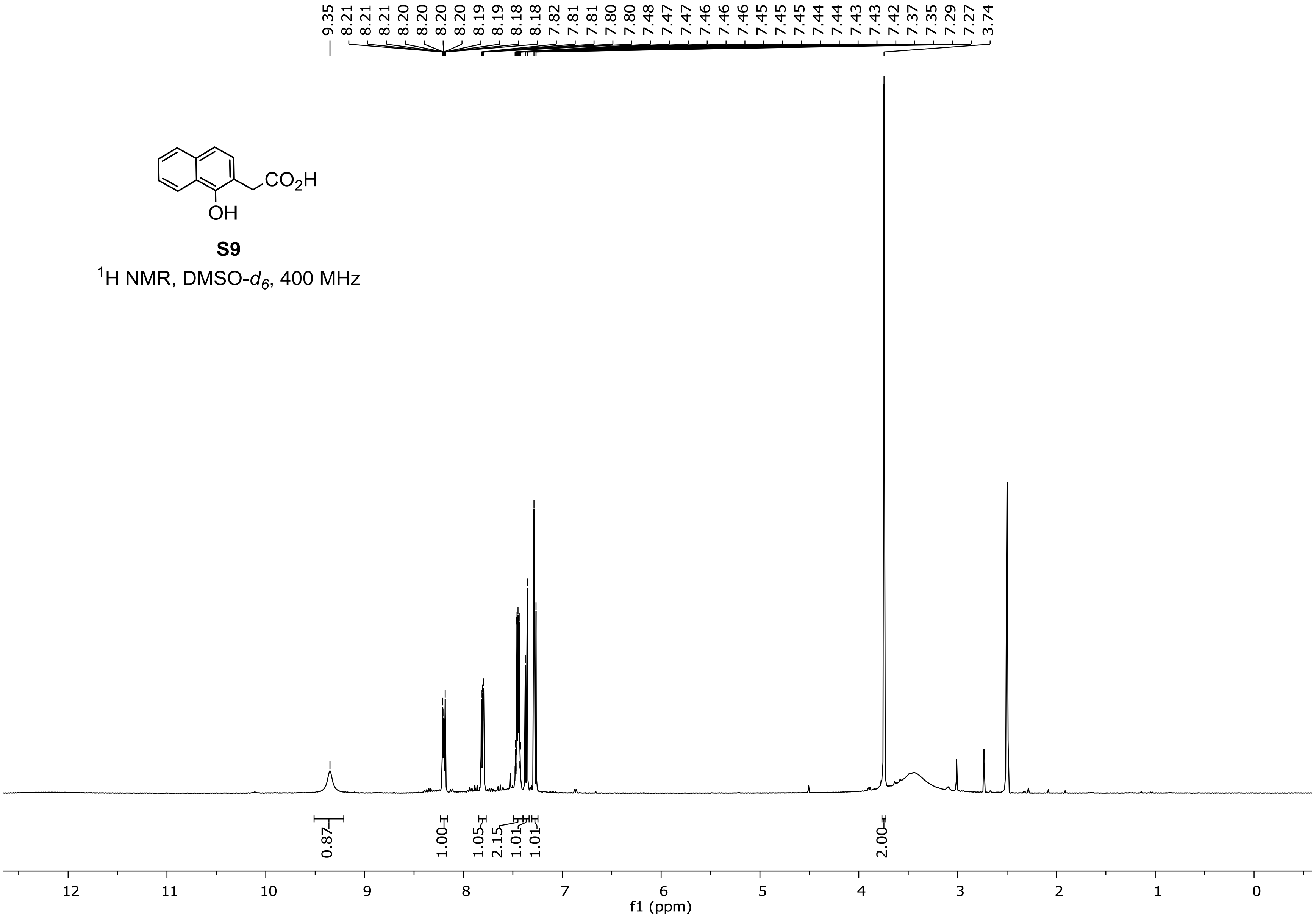

S9

^{1}H NMR, DMSO-d_6, 400 MHz

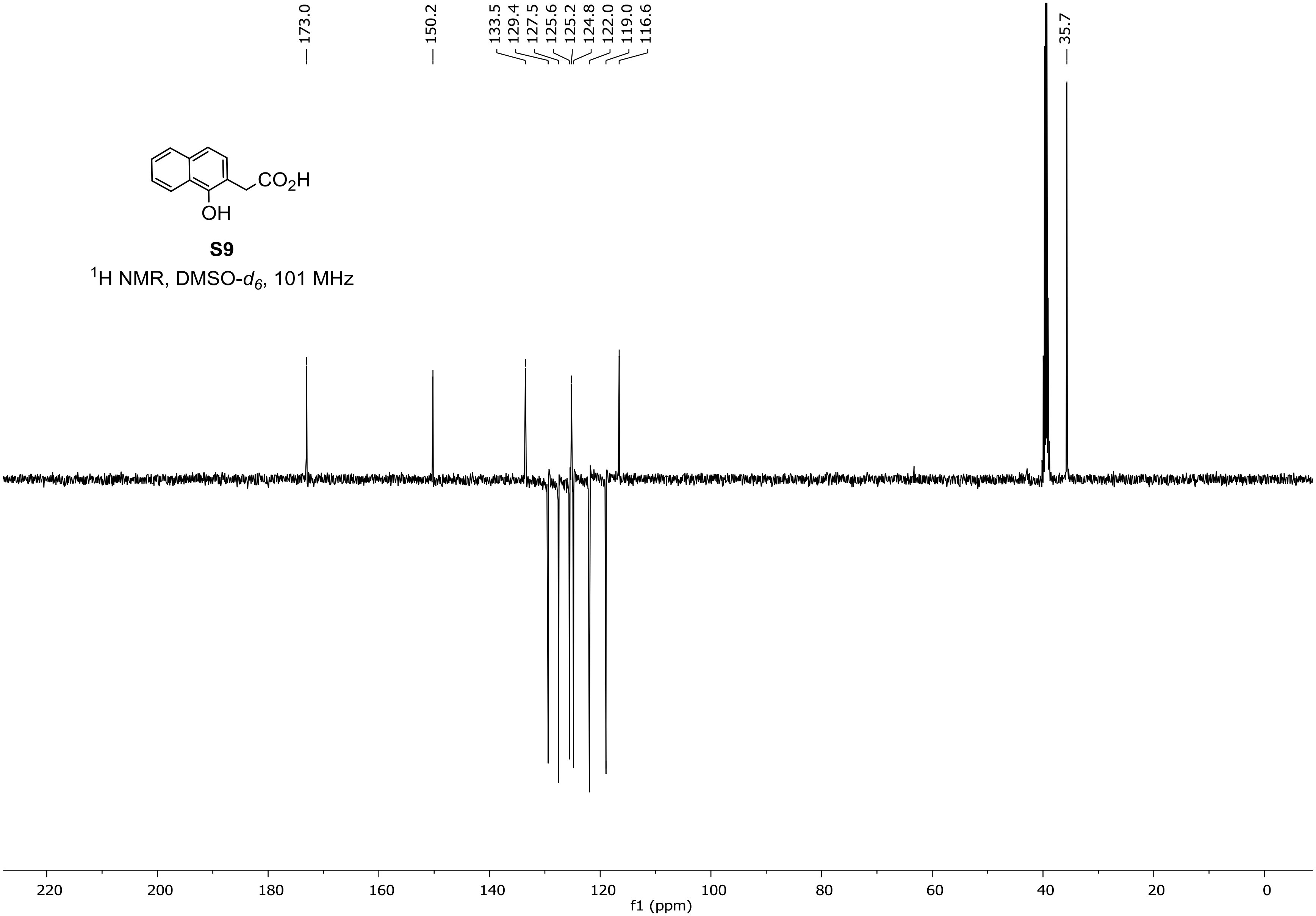
173.0
150.2
133.5
129.4
127.5
125.6
125.2
124.8
122.0
119.0
116.6
35.7
CO2H
OH
S9
1H NMR, DMSO-d6, 101 MHz
220
200
180
160
140
120
100
80
60
40
20
0
f1 (ppm)

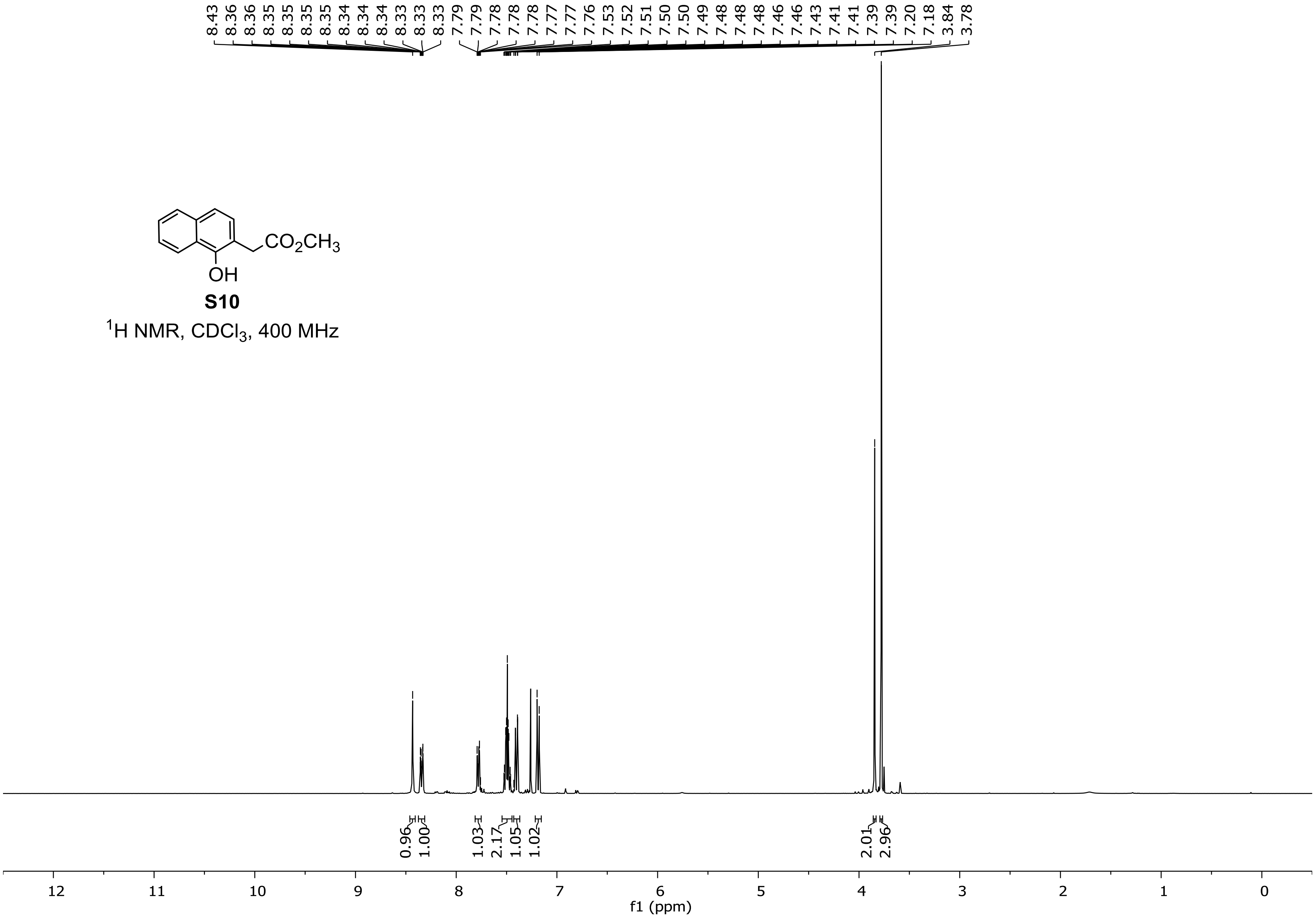

8.43
8.36
8.36
8.35
8.35
8.35
8.35
8.34
8.34
8.34
8.33
8.33
8.33
7.79
7.79
7.78
7.78
7.78
7.77
7.77
7.76
7.53
7.52
7.51
7.50
7.50
7.49
7.48
7.48
7.48
7.46
7.46
7.43
7.41
7.41
7.39
7.39
7.20
7.18
3.84
3.78
CO2CH3
OH
S10
1H NMR, CDCl3, 400 MHz
0.96
1.00
1.03
2.17
1.05
1.02
2.01
2.96
12
11
10
9
8
7
6
5
4
3
2
1
0
f1 (ppm)

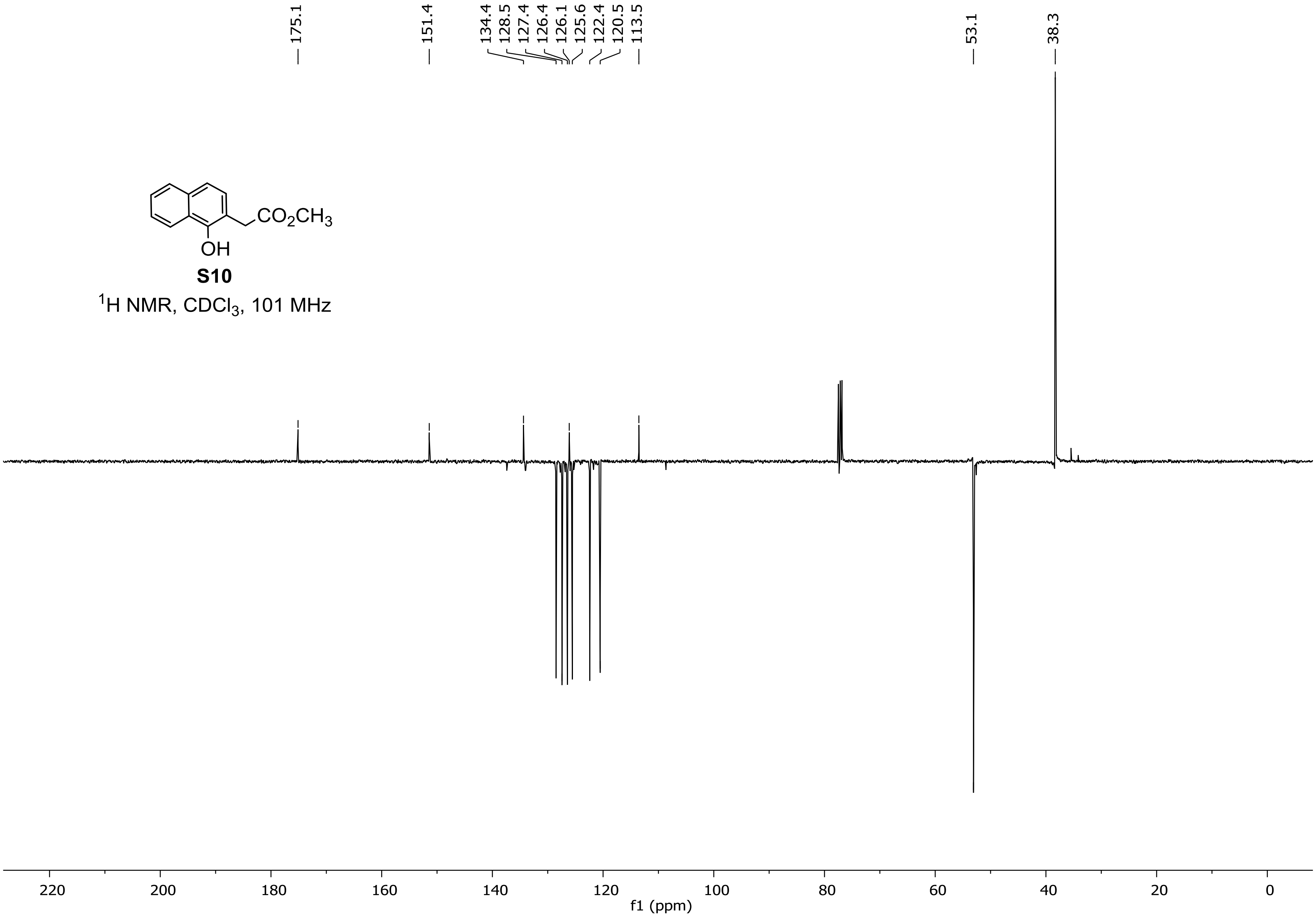
175.1
151.4
134.4
128.5
127.4
126.4
126.1
125.6
122.4
120.5
113.5
53.1
38.3
CO_2CH_3
OH
S10
^{1}H NMR, $CDCl_3$, 101 MHz
220
200
180
160
140
120
100
80
60
40
20
0
f1 (ppm)

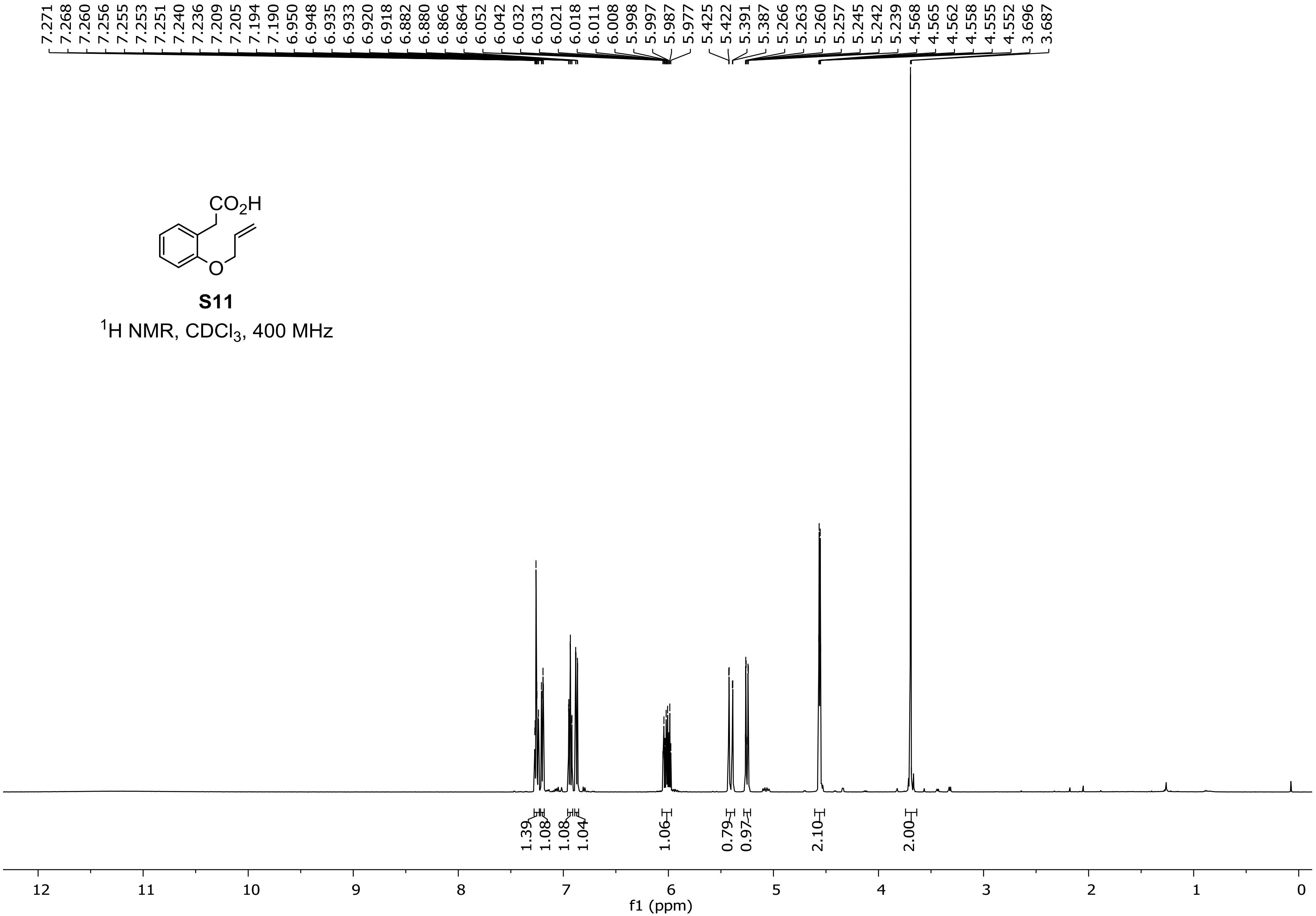

S11

^1H NMR, $CDCl_3$, 400 MHz

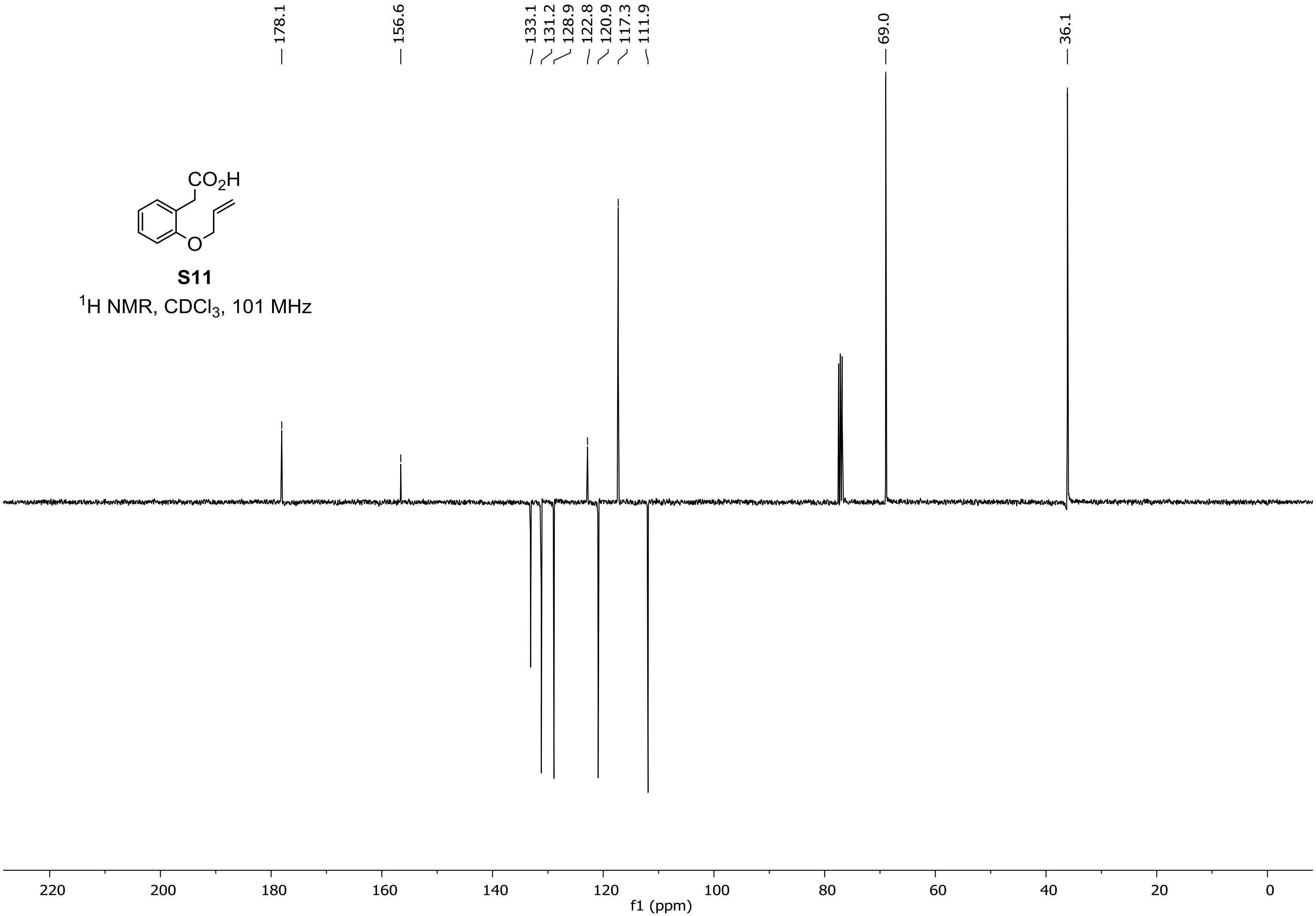

S11

^{1}H NMR, $CDCl_3$, 101 MHz

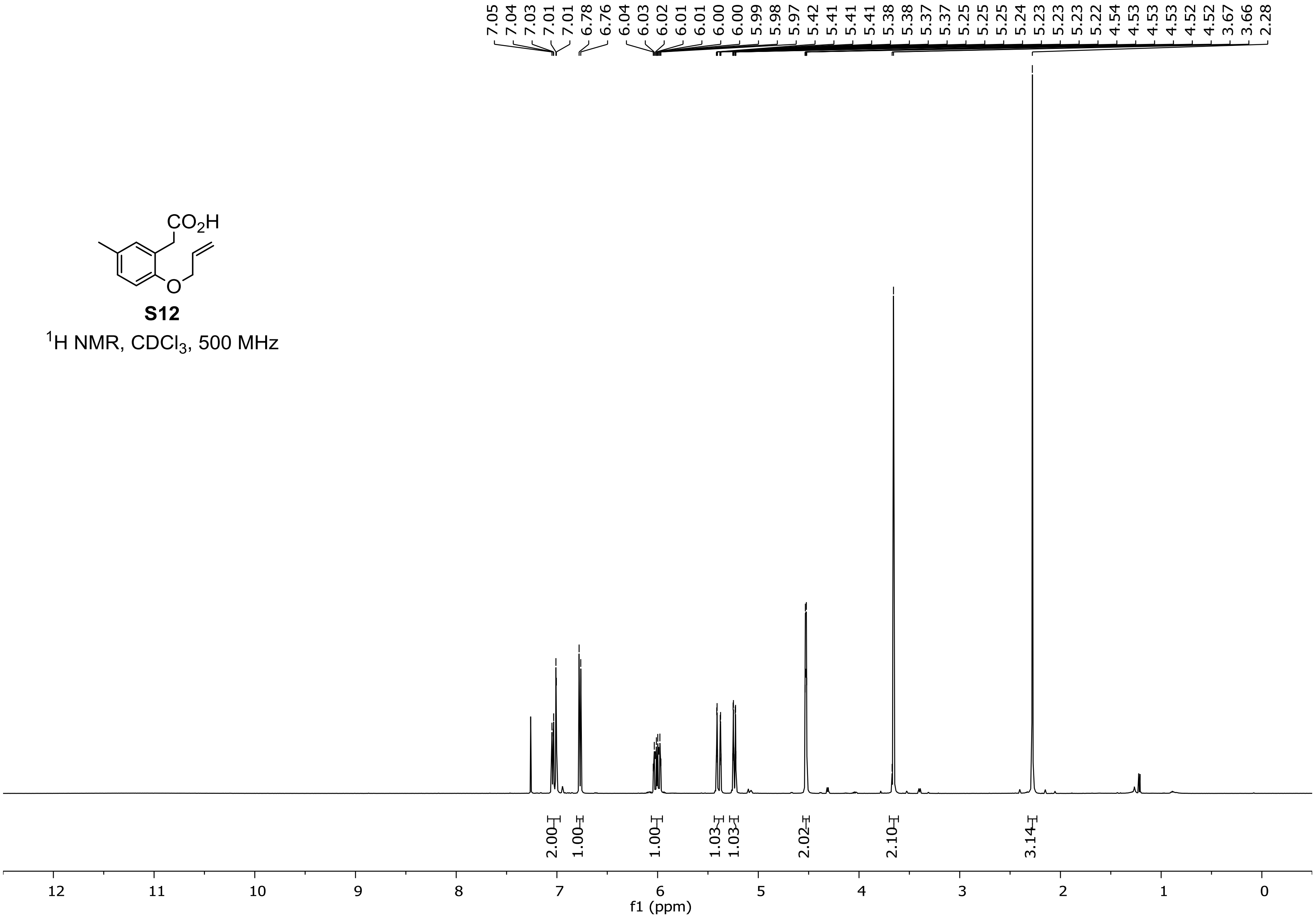

S12
^{1}H NMR, $CDCl_3$, 500 MHz
7.05 7.04 7.03 7.01 7.01 6.78 6.76 6.04 6.03 6.02 6.01 6.01 6.00 6.00 5.99 5.98 5.97 5.42 5.41 5.41 5.41 5.38 5.38 5.37 5.37 5.25 5.25 5.25 5.24 5.23 5.23 5.23 5.22 4.54 4.53 4.53 4.53 4.52 4.52 3.67 3.66 2.28
2.00 1.00 1.00 1.03 1.03 2.02 2.10 3.14
12 11 10 9 8 7 6 5 4 3 2 1 0
f1 (ppm)

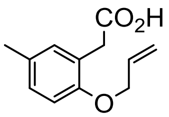

S12

^{13}C NMR, $CDCl_3$, 126 MHz

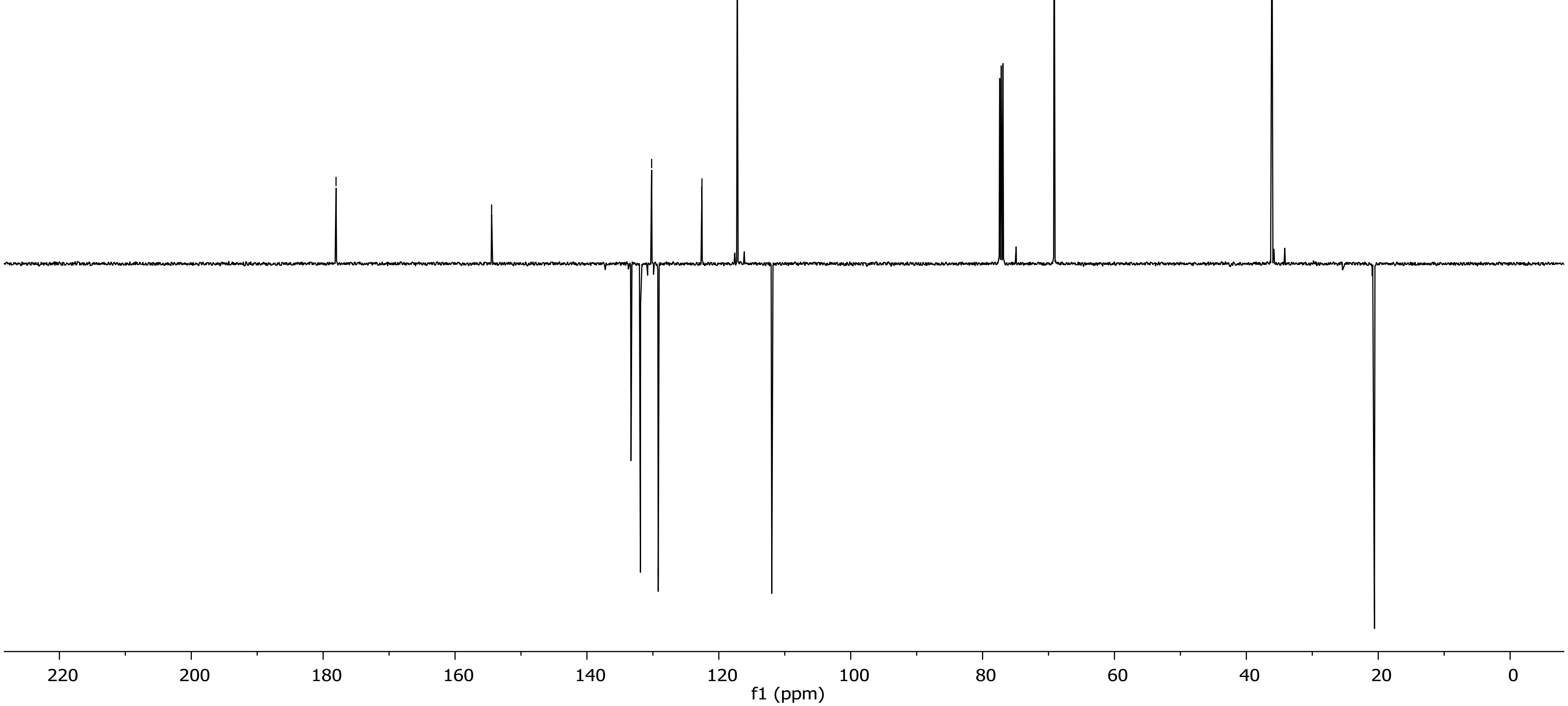

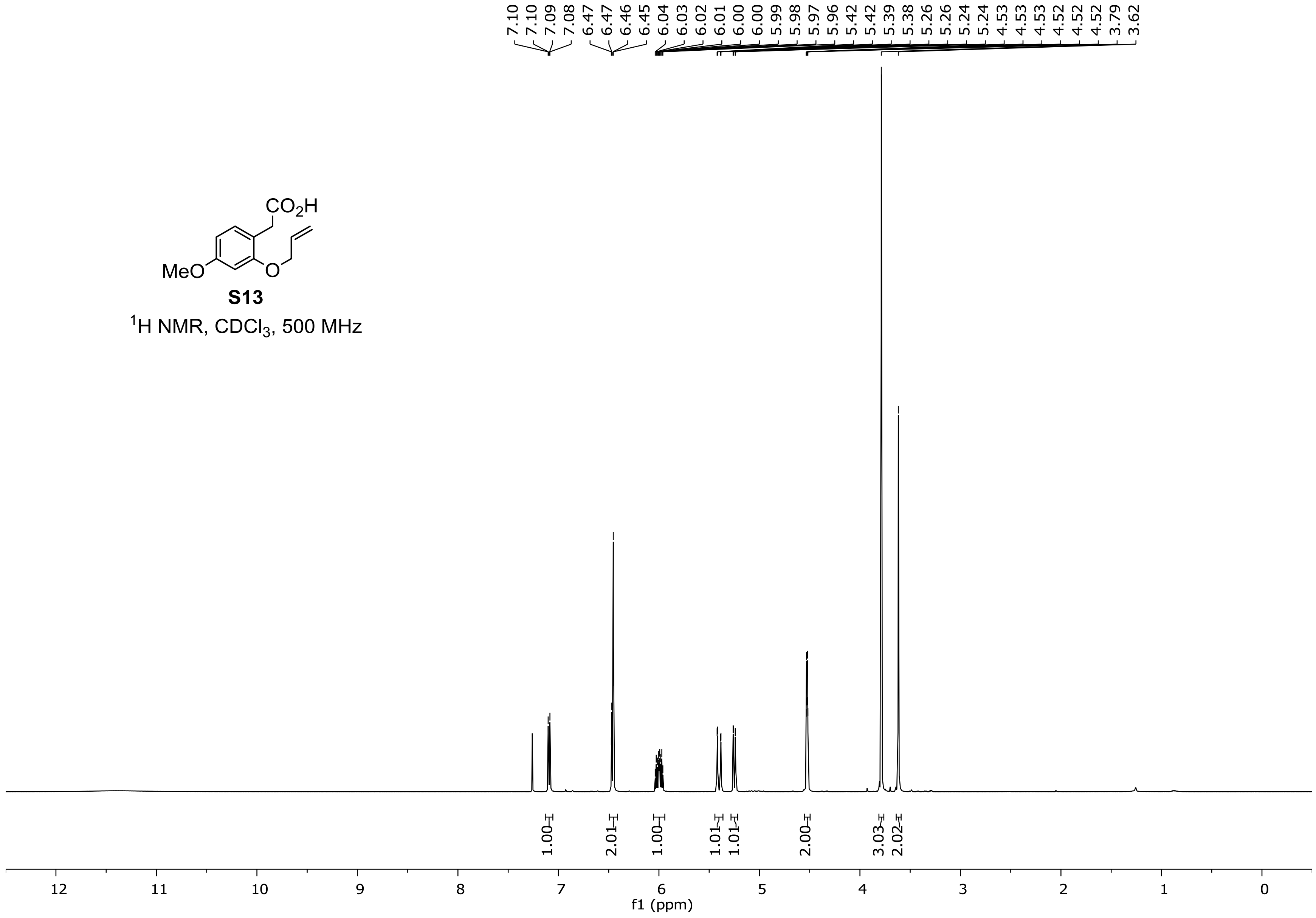

S13

^{1}H NMR, $CDCl_3$, 500 MHz

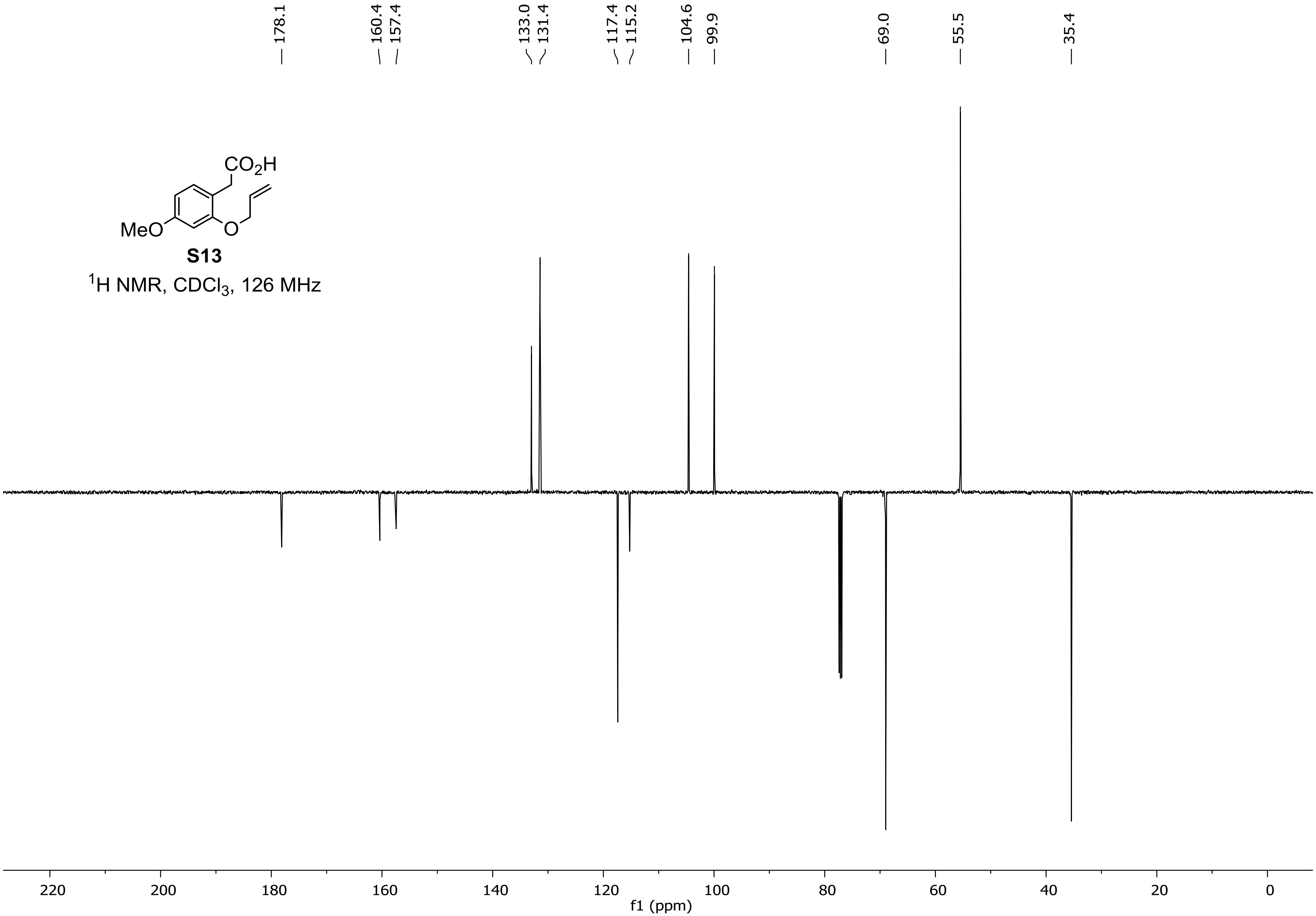
178.1
160.4
157.4
133.0
131.4
117.4
115.2
104.6
99.9
69.0
55.5
35.4
CO2H
MeO
O
S13
1H NMR, CDCl3, 126 MHz
220
200
180
160
140
120
100
80
60
40
20
0
f1 (ppm)

06132016-15-ads-rmnp-M.11.1.1r
1H Observe
2-66 P2, 4-F Allyl Acid

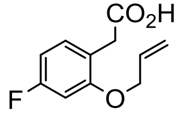

S14

^{1}H NMR, $CDCl_3$, 400 MHz

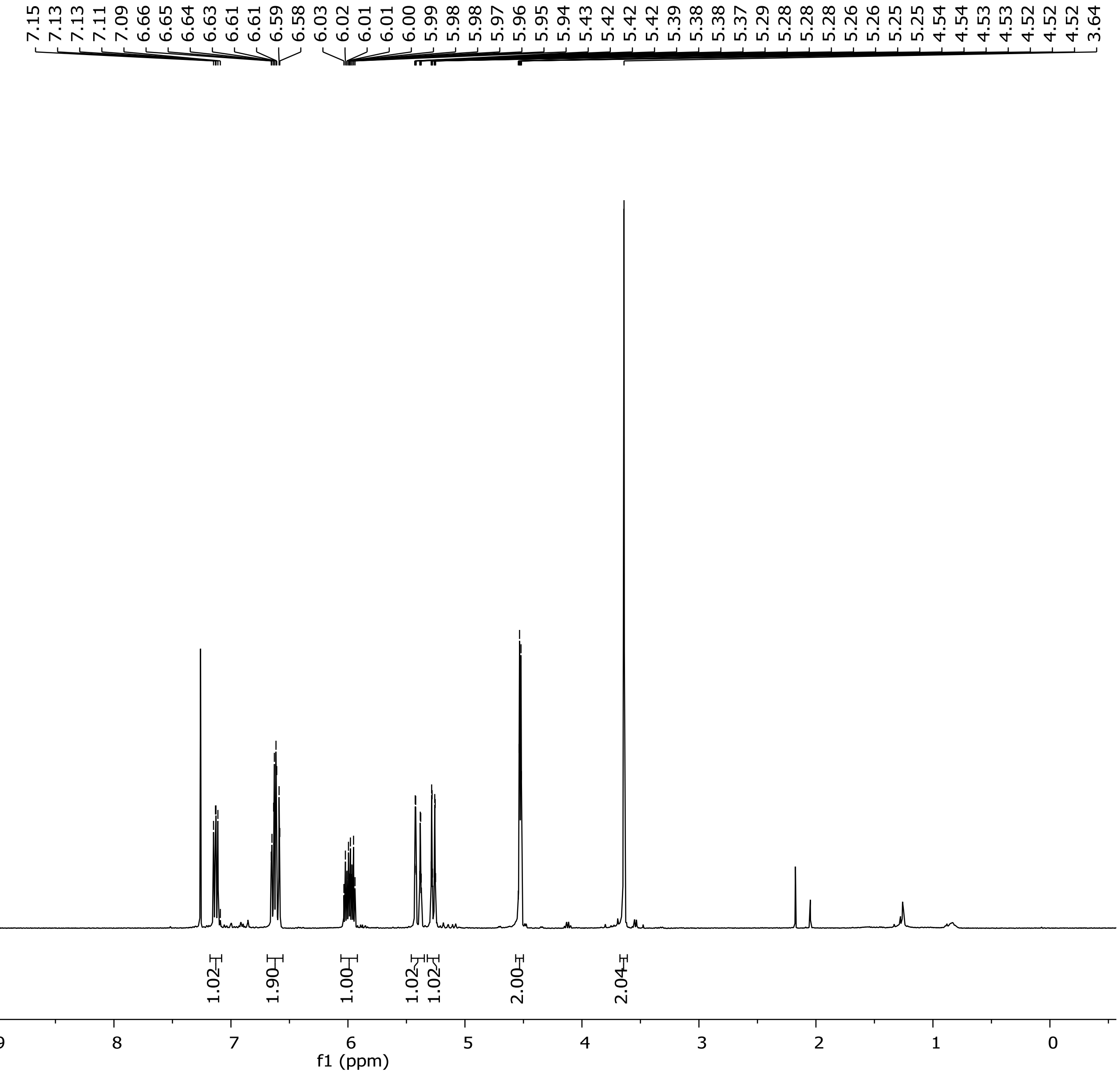

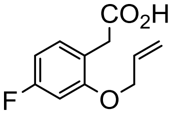

S14

^{1}H NMR, $CDCl_3$, 101 MHz

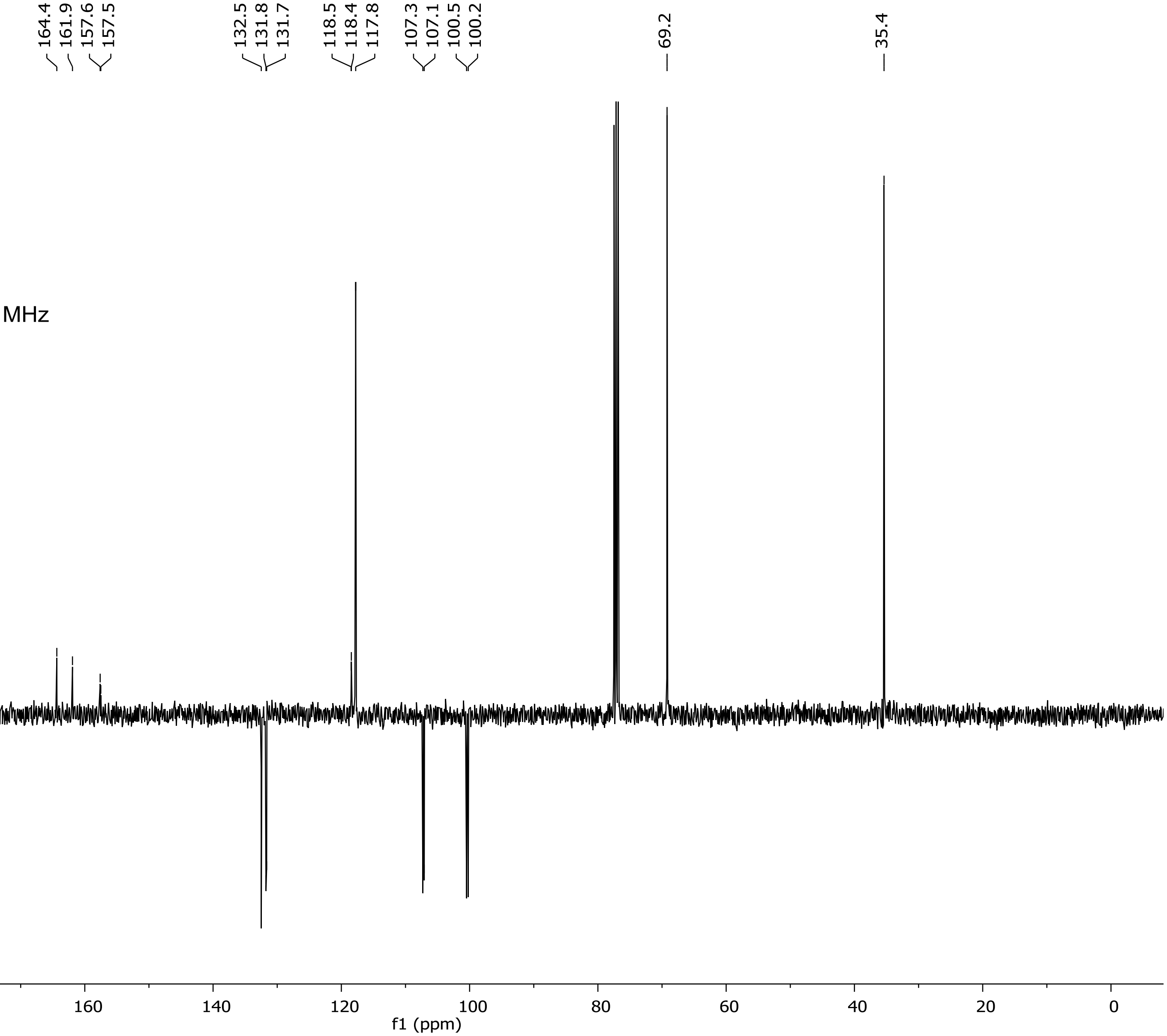

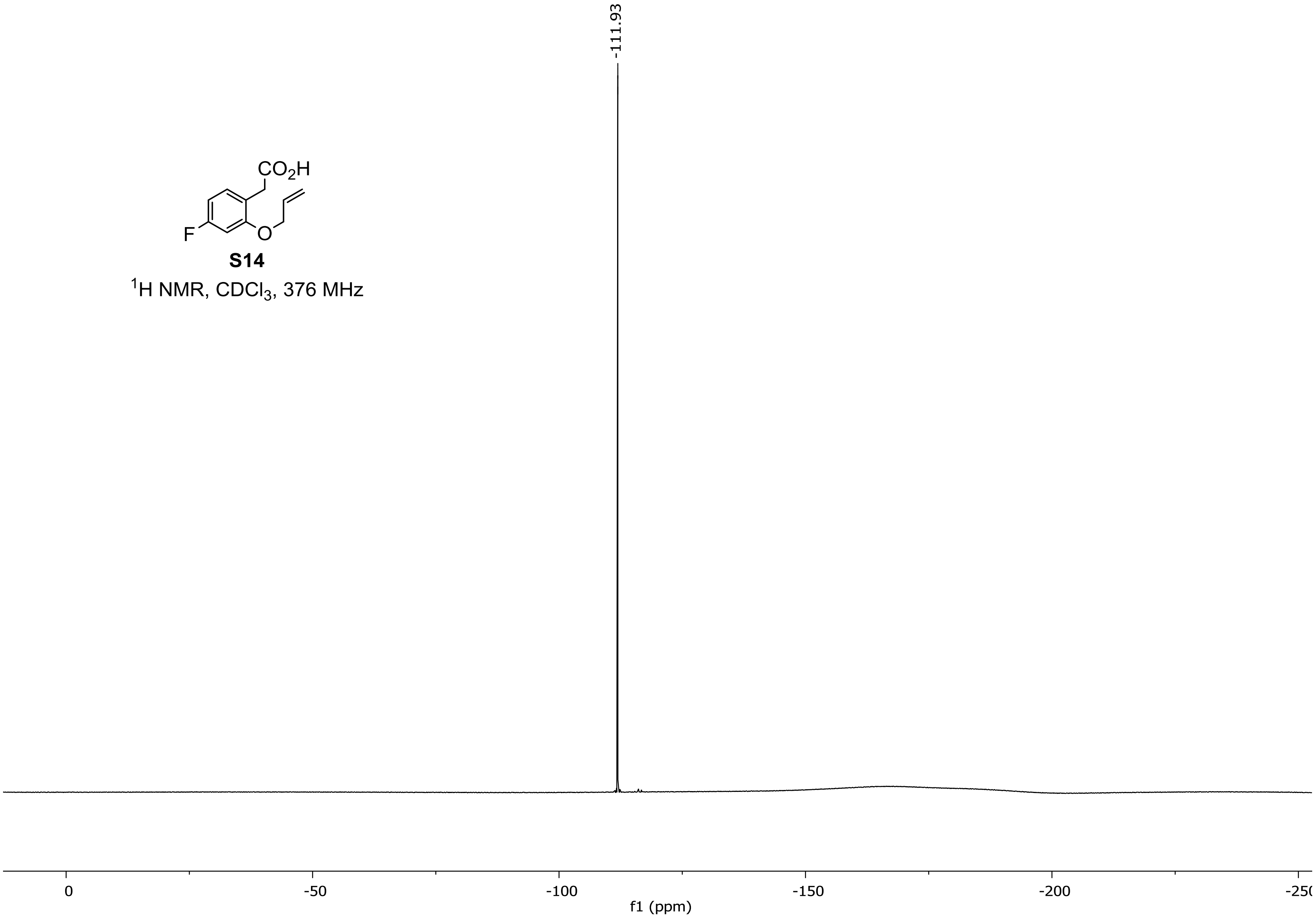
-111.93
CO_2H
F
O
S14
^1H NMR, $CDCl_3$, 376 MHz
0
-50
-100
-150
-200
-25(
f1 (ppm)

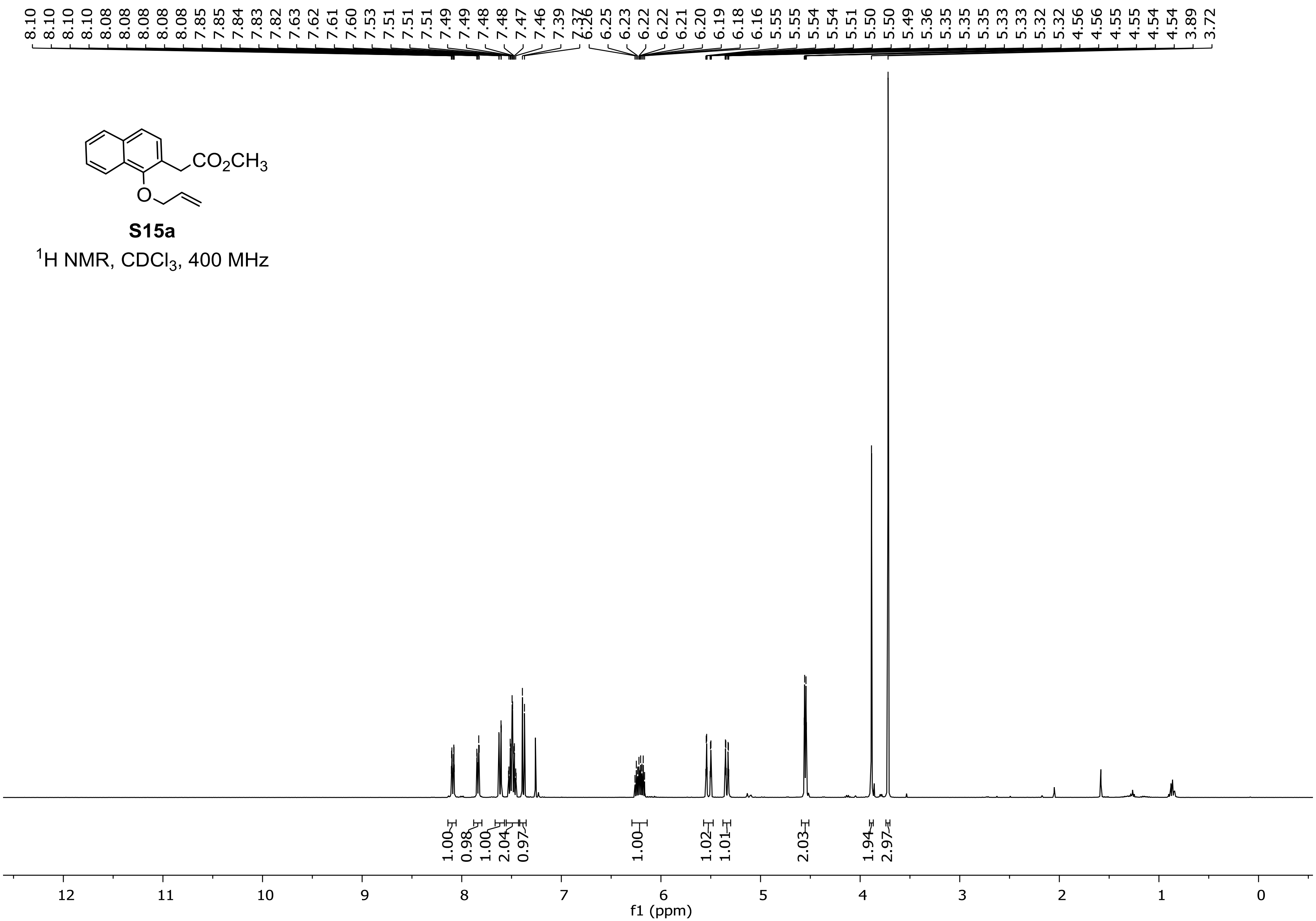
CO2CH3
O
S15a
1H NMR, CDCl3, 400 MHz
f1 (ppm)

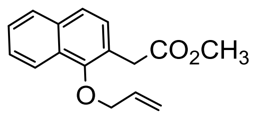

S15a

^{1}H NMR, $CDCl_3$, 101 MHz

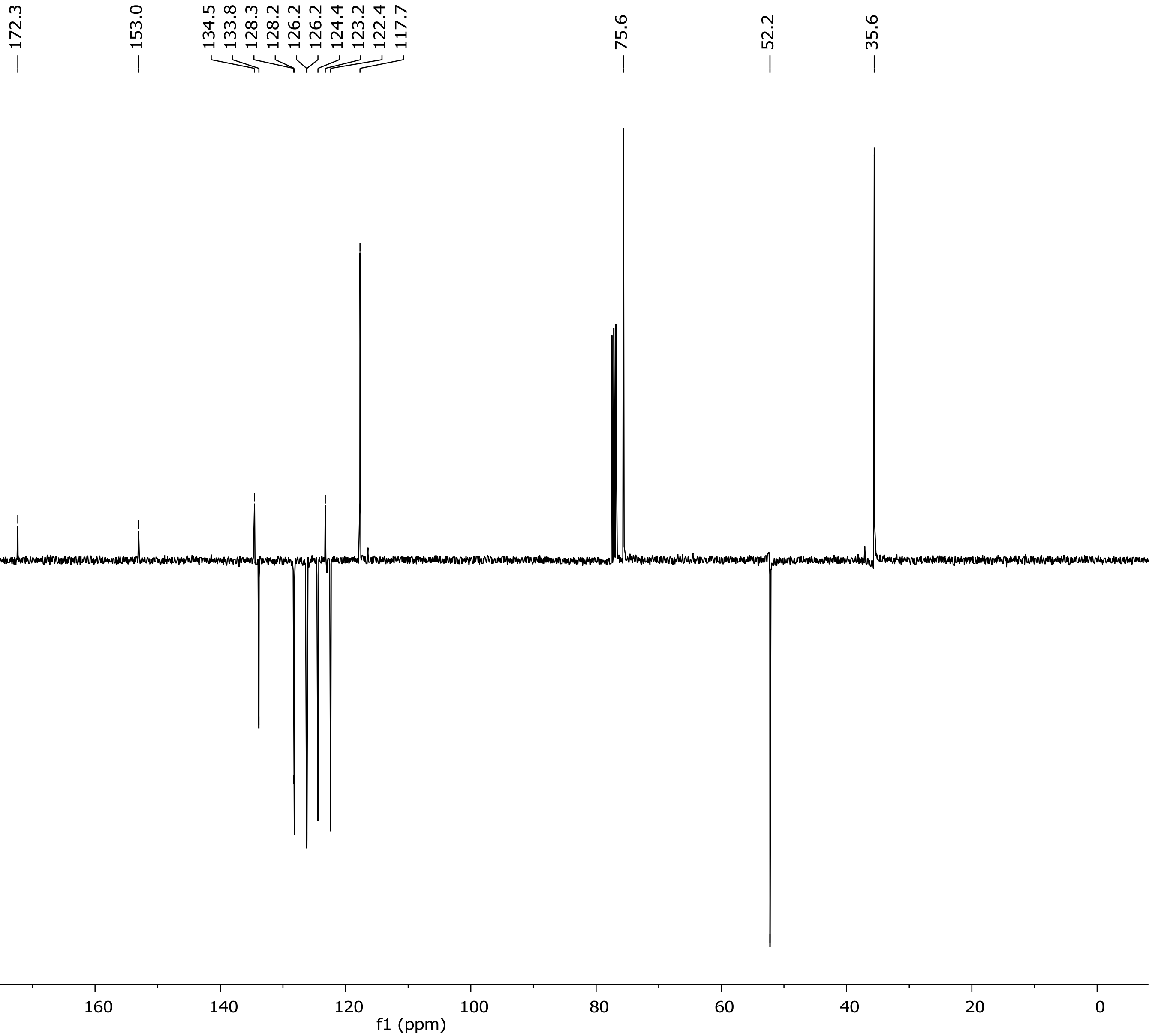

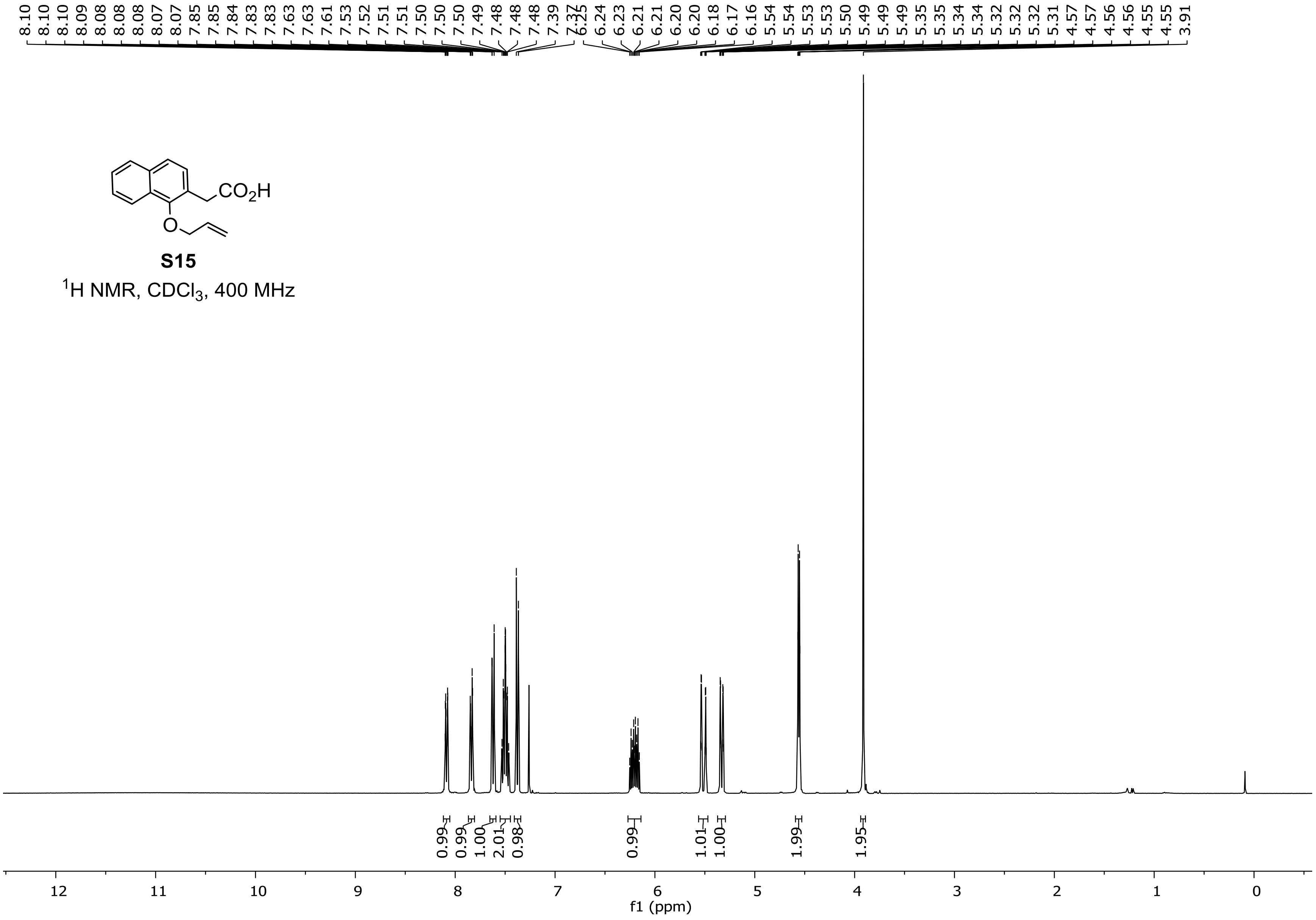
8.10 8.10 8.10 8.09 8.08 8.08 8.08 8.07 7.85 7.85 7.84 7.83 7.83 7.63 7.63 7.61 7.53 7.52 7.51 7.51 7.50 7.50 7.50 7.49 7.48 7.48 7.48 7.39 7.37 6.25 6.24 6.23 6.21 6.21 6.20 6.20 6.18 6.17 6.16 5.54 5.54 5.53 5.53 5.50 5.49 5.49 5.49 5.35 5.35 5.34 5.34 5.32 5.32 5.32 5.31 4.57 4.57 4.56 4.56 4.55 4.55 3.91
CO2H
O
S15
1H NMR, CDCl3, 400 MHz
0.99 0.99 1.00 2.01 0.98 0.99 1.01 1.00 1.99 1.95
12 11 10 9 8 7 6 5 4 3 2 1 0
f1 (ppm)

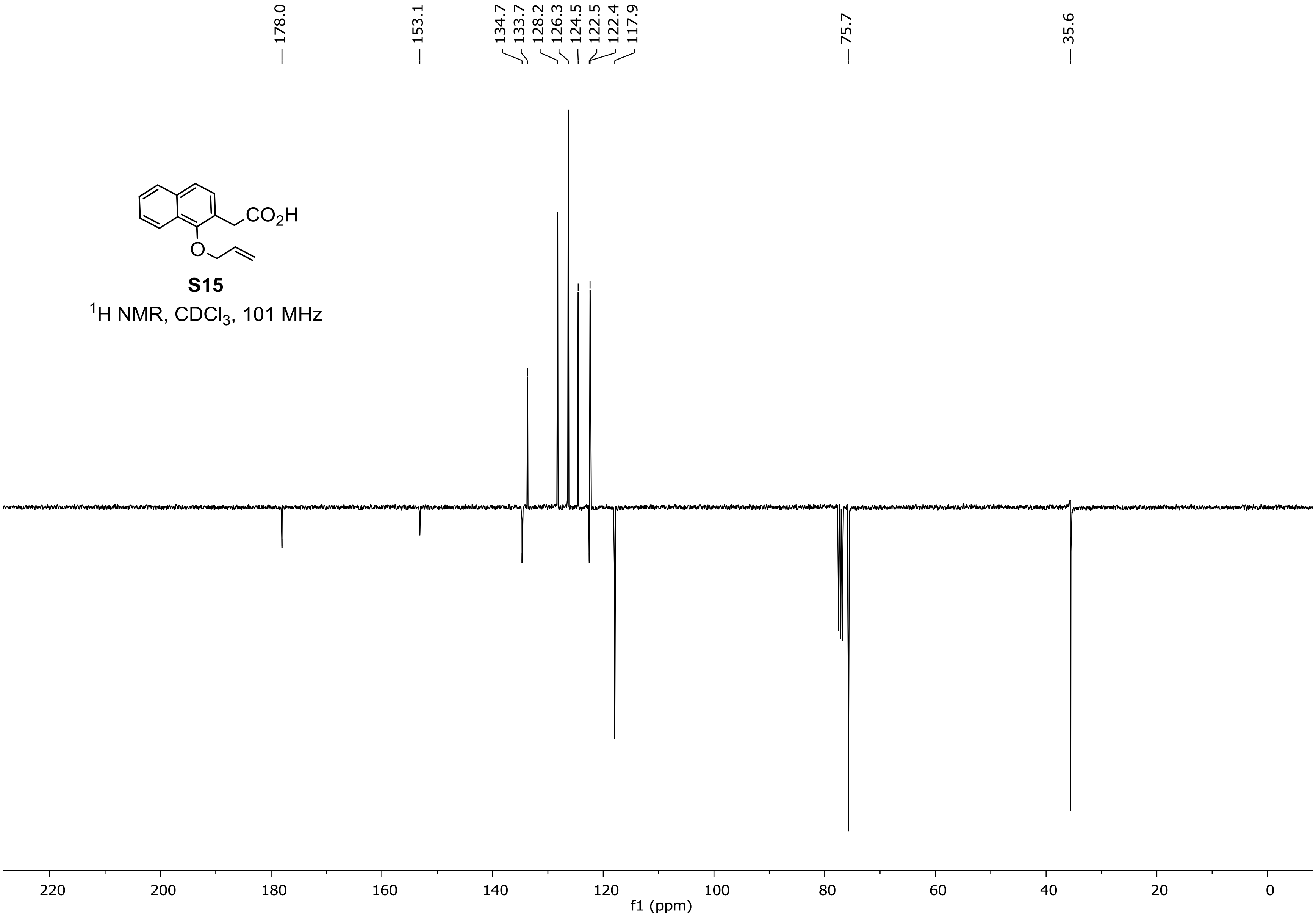
178.0
153.1
134.7
133.7
128.2
126.3
124.5
122.5
122.4
117.9
75.7
35.6
CO2H
O
S15
1H NMR, CDCl3, 101 MHz
220 200 180 160 140 120 100 80 60 40 20 0
f1 (ppm)

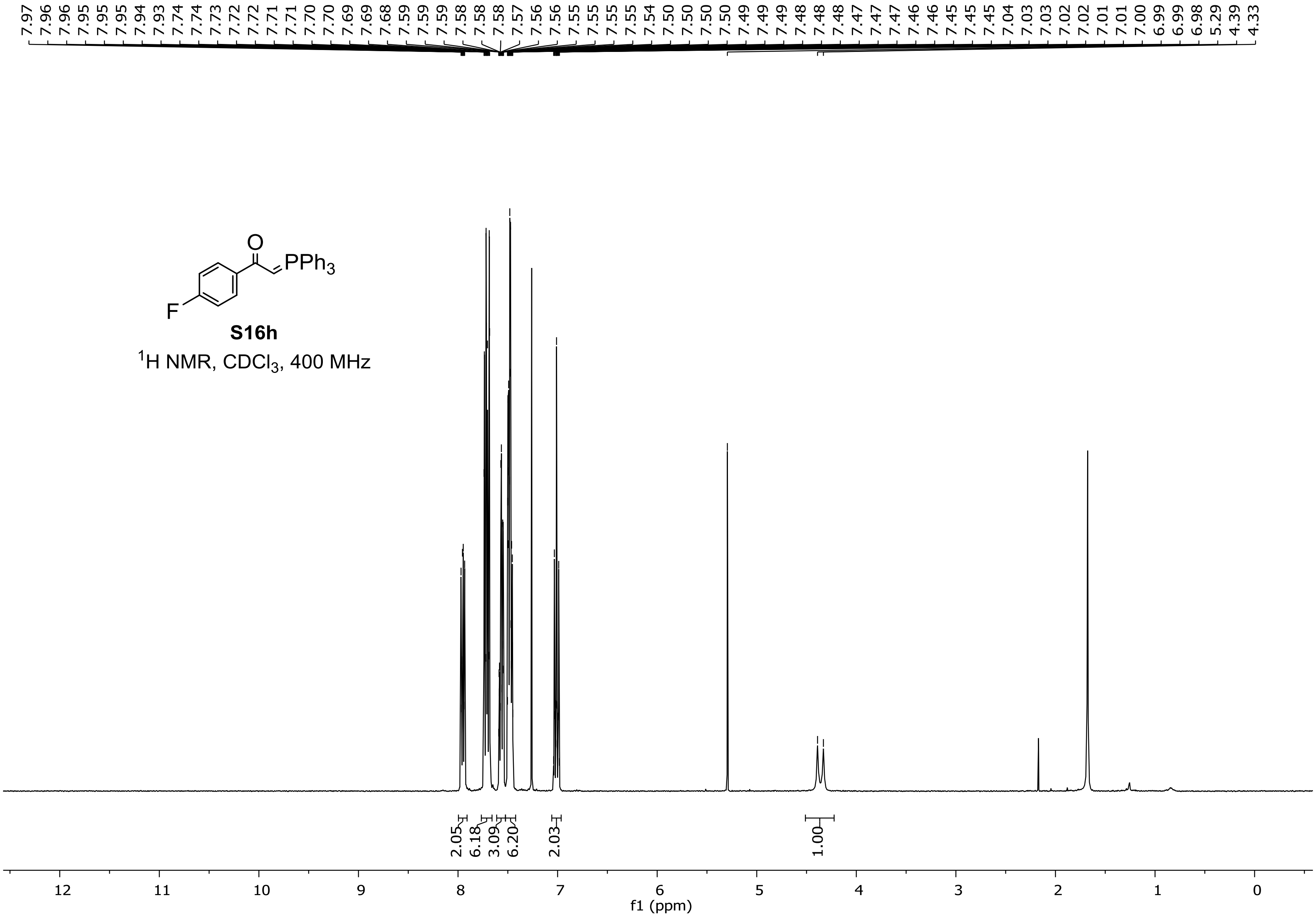
S16h
^{1}H NMR, $CDCl_3$, 400 MHz
f1 (ppm)

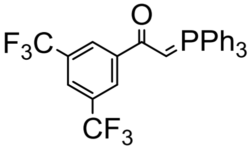

S16i

^{1}H NMR, $CDCl_3$, 500 MHz

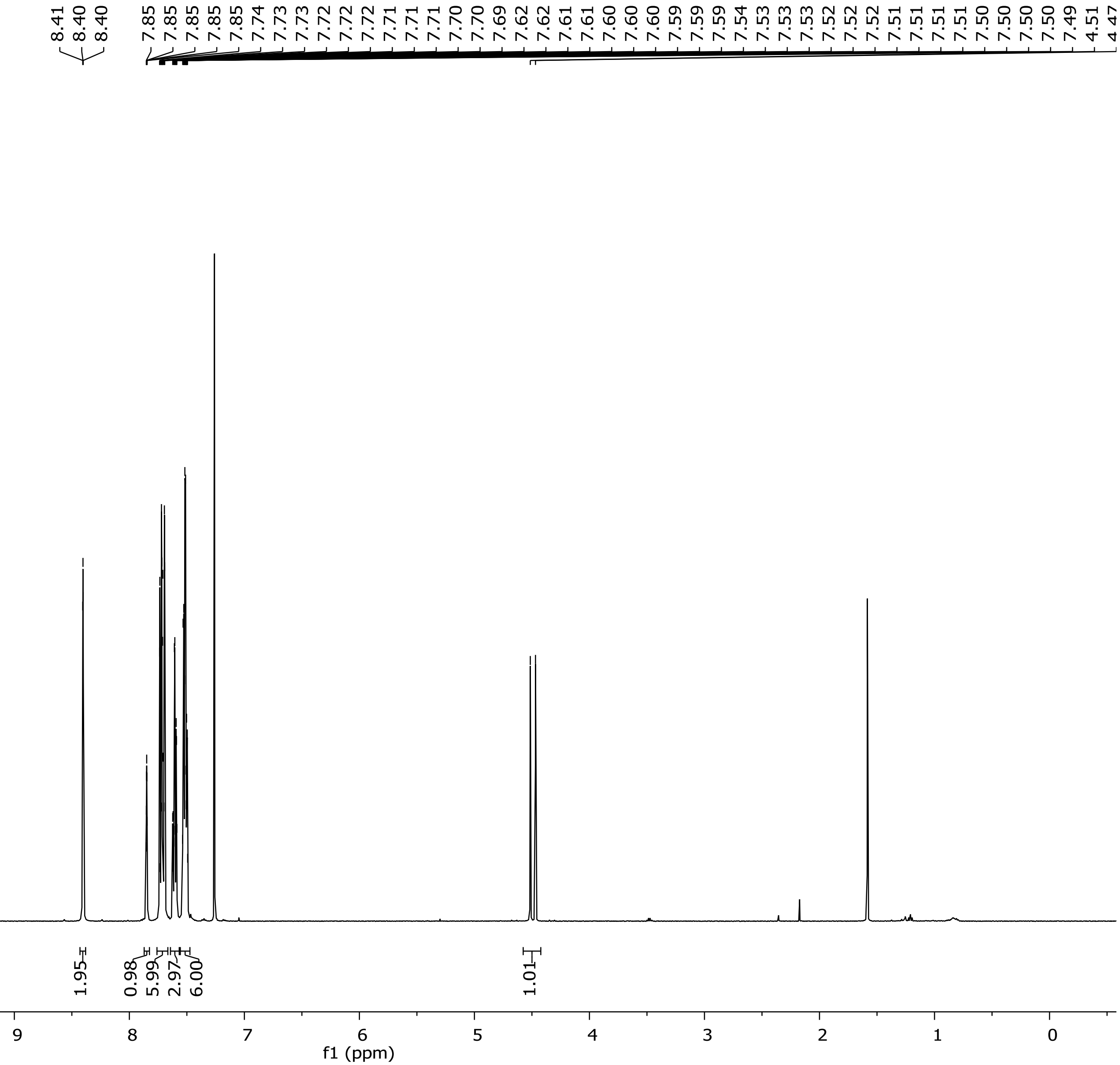

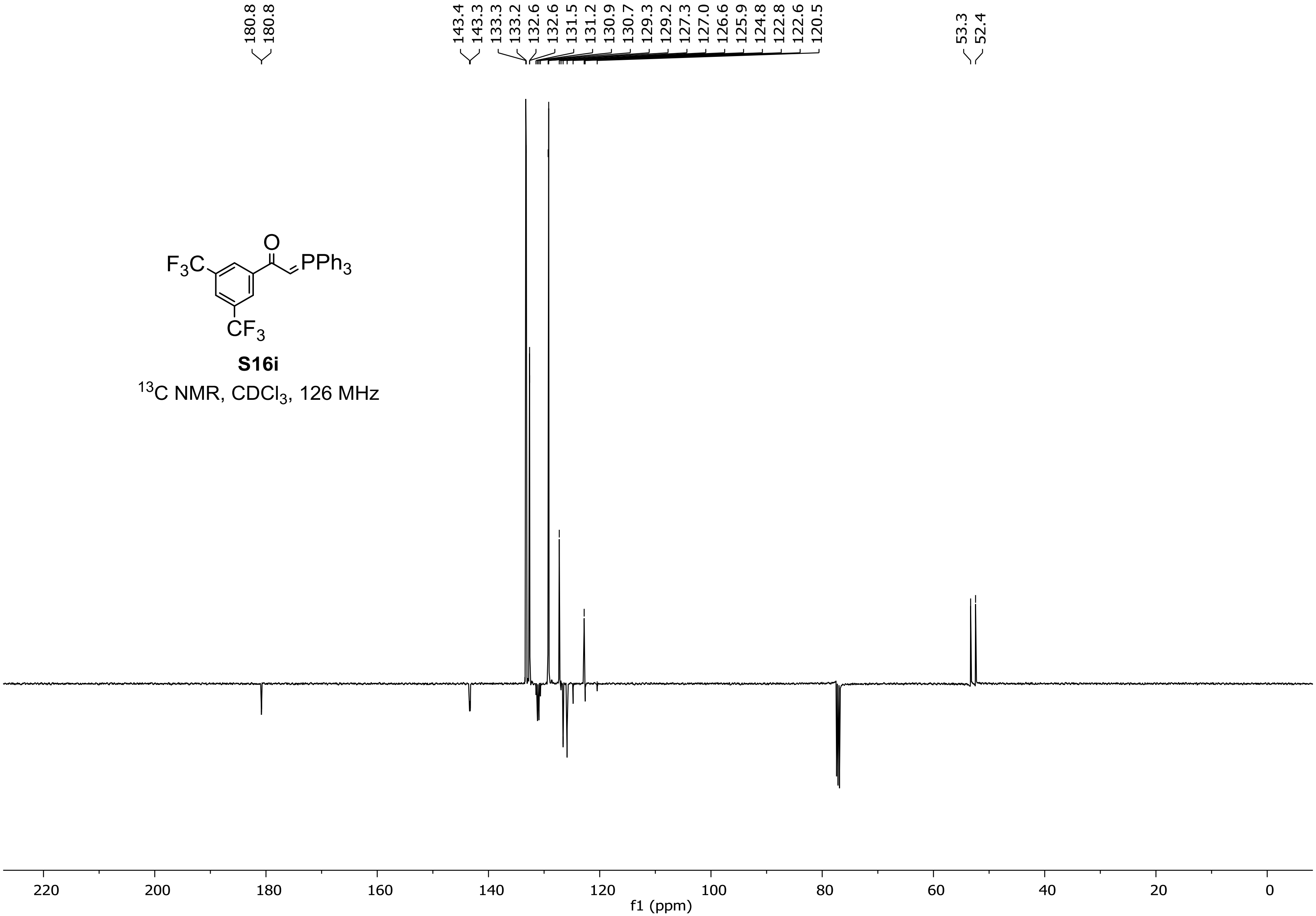
180.8
180.8
143.4
143.3
133.3
133.2
132.6
132.6
131.5
131.2
130.9
130.7
129.3
129.2
127.3
127.0
126.6
125.9
124.8
122.8
122.6
120.5
53.3
52.4
O
F_3C
PPh_3
CF_3
S16i
^{13}C NMR, $CDCl_3$, 126 MHz
220
200
180
160
140
120
100
80
60
40
20
0
f1 (ppm)

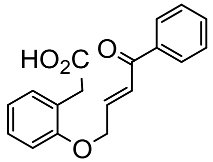

1

^1H NMR, $CDCl_3$, 500 MHz

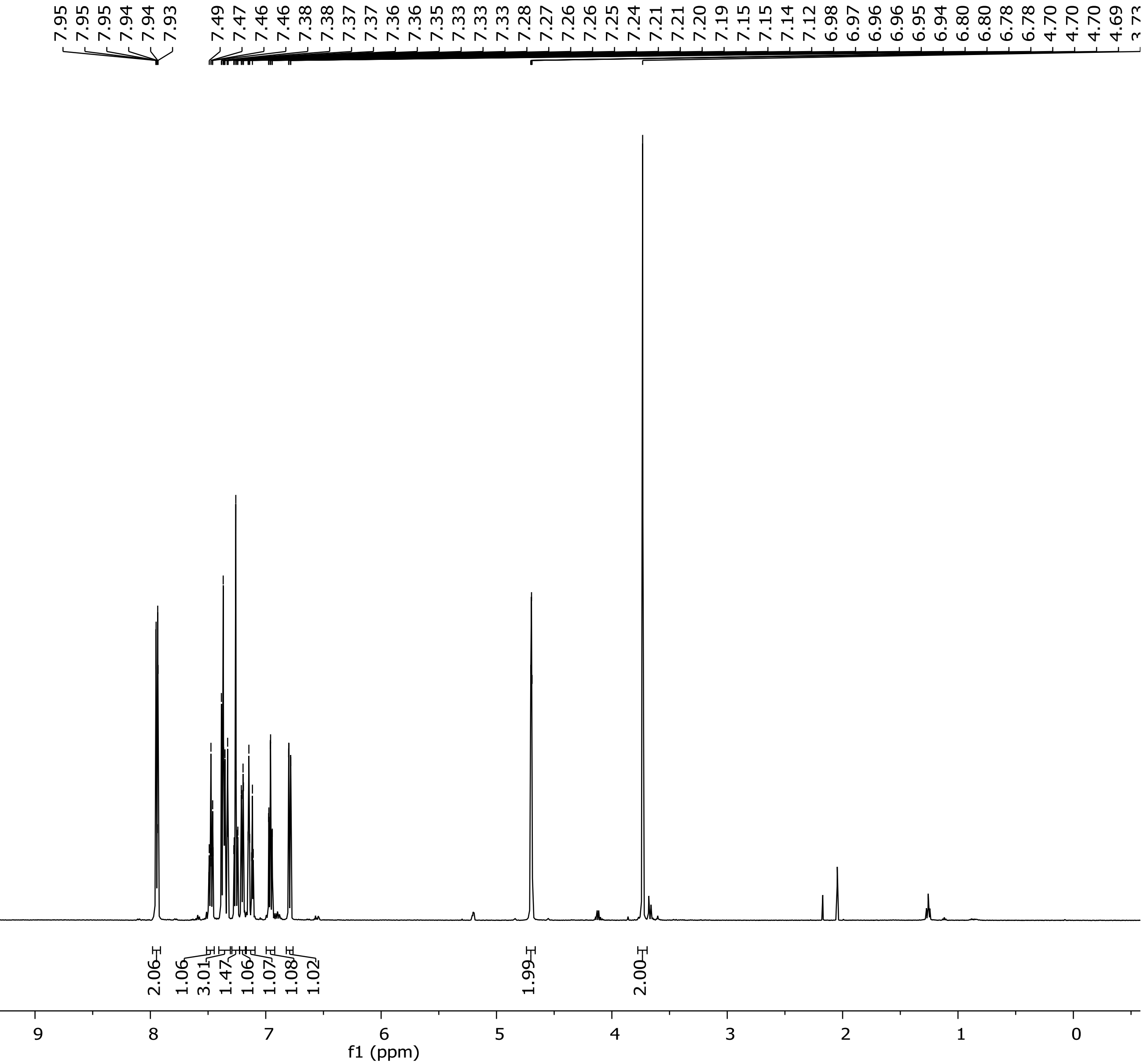

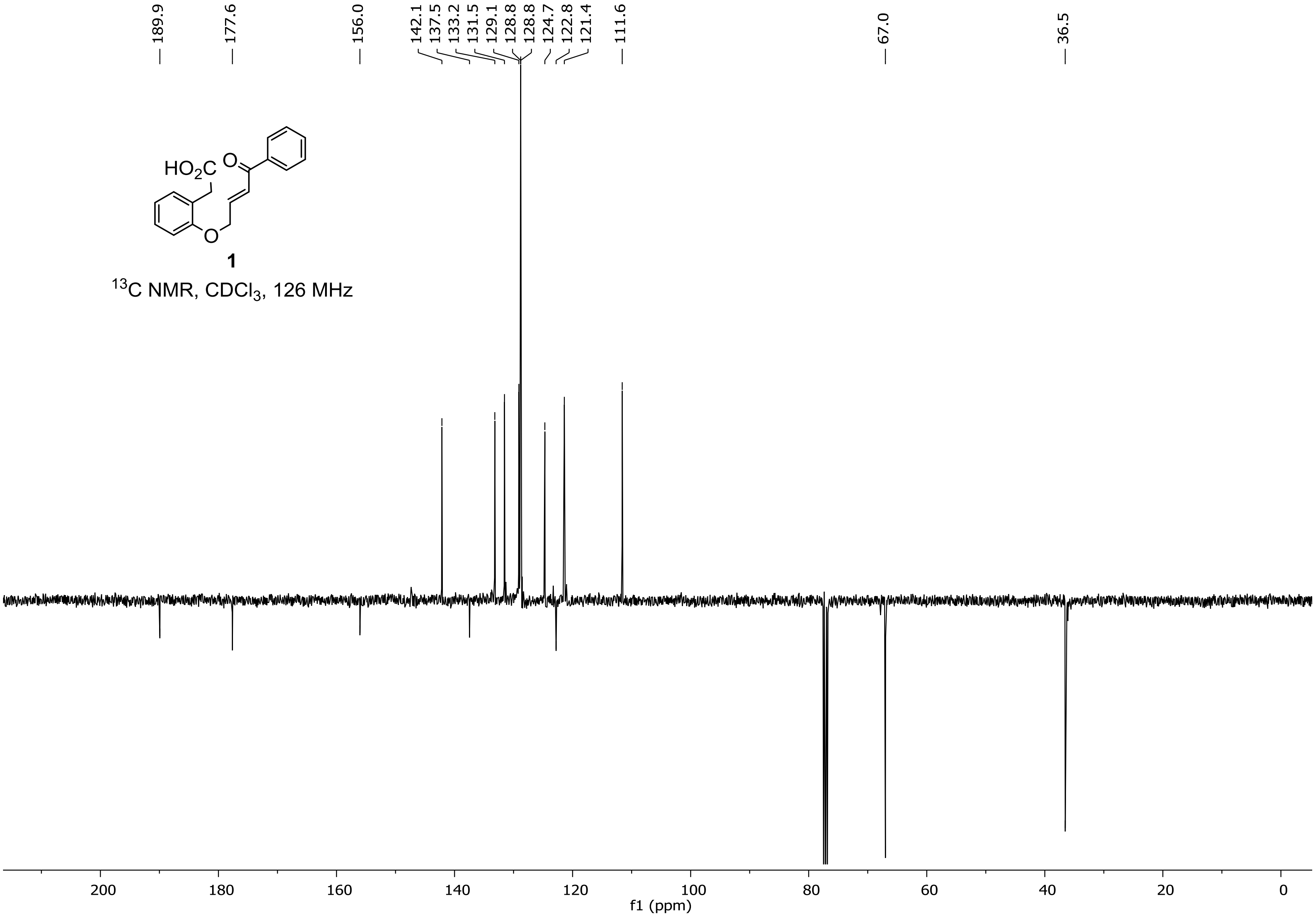
189.9
177.6
156.0
142.1
137.5
133.2
131.5
129.1
128.8
128.8
124.7
122.8
121.4
111.6
67.0
36.5
HO2C
O
O
1
13C NMR, CDCl3, 126 MHz
200
180
160
140
120
100
80
60
40
20
0
f1 (ppm)

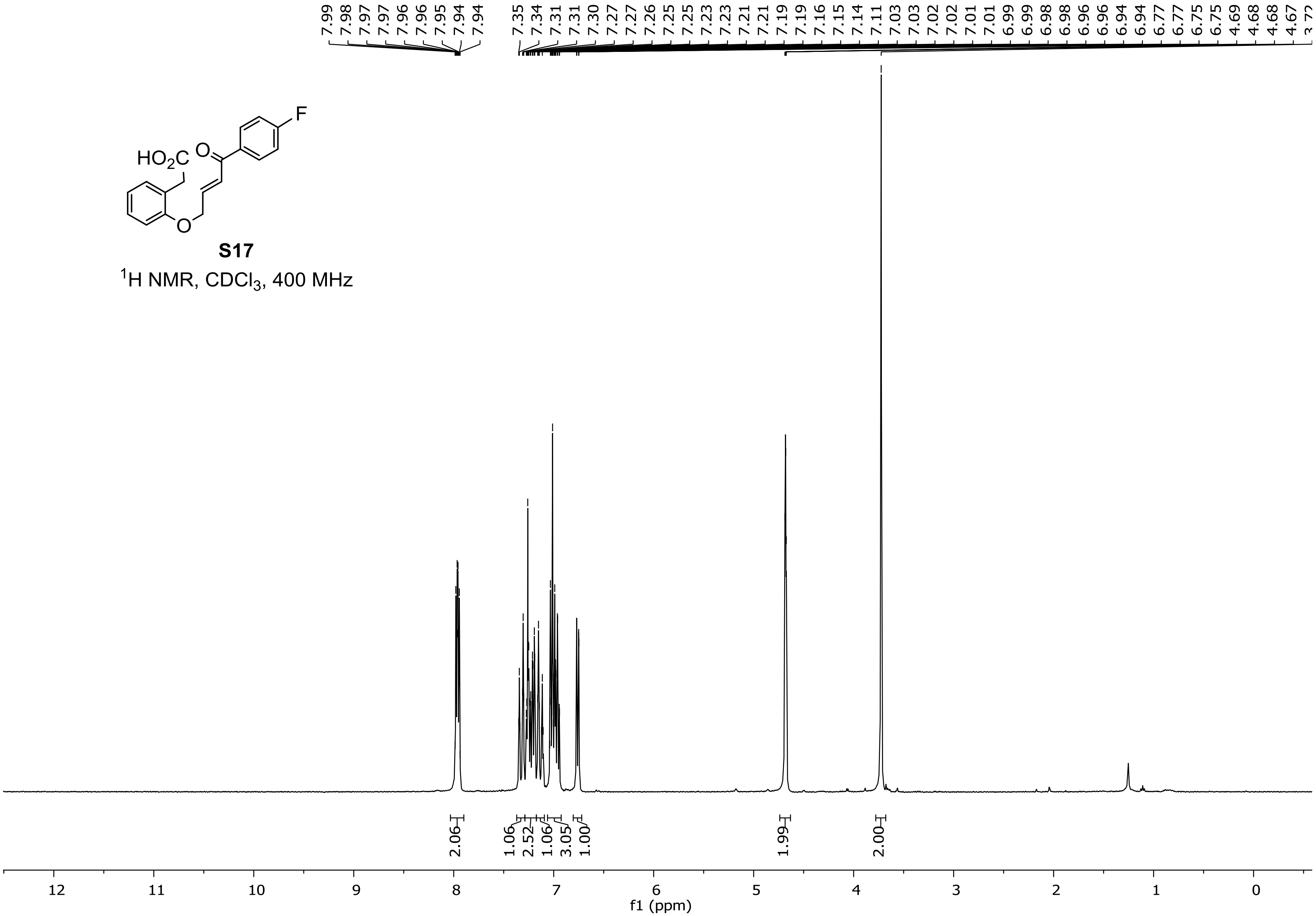
7.99 7.98 7.97 7.97 7.96 7.96 7.95 7.94 7.94
7.35 7.34 7.31 7.31 7.30 7.27 7.27 7.26 7.25 7.25 7.23 7.23 7.21 7.21 7.19 7.19 7.16 7.15 7.14 7.11 7.03 7.03 7.02 7.02 7.01 7.01 6.99 6.99 6.98 6.98 6.96 6.96 6.94 6.94 6.77 6.77 6.75 6.75 4.69 4.68 4.68 4.67
HO2C
O
F
O
S17
1H NMR, CDCl3, 400 MHz
2.06
1.06
2.52
1.06
3.05
1.00
1.99
2.00
12 11 10 9 8 7 6 5 4 3 2 1 0
f1 (ppm)

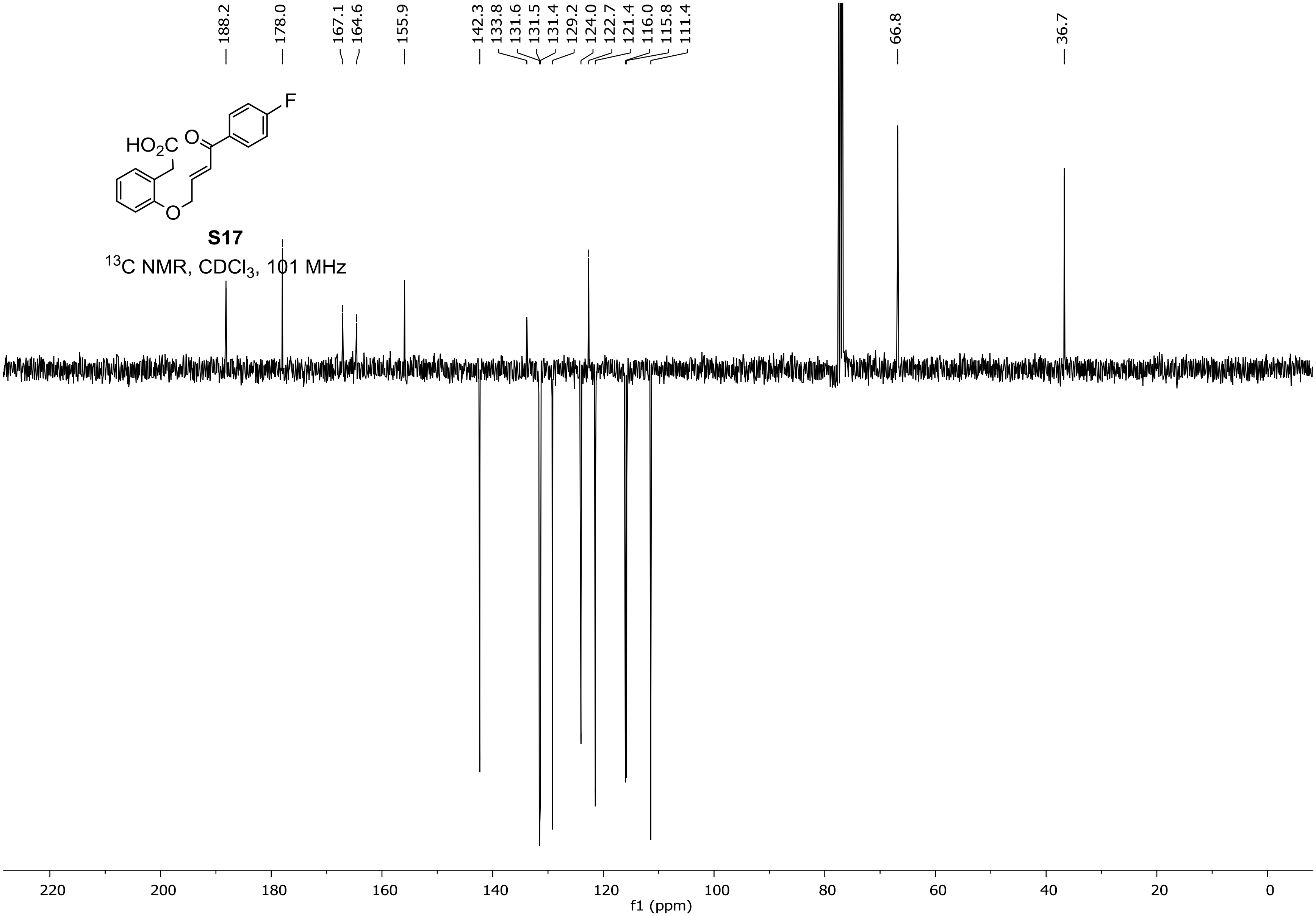

188.2
178.0
167.1
164.6
155.9
142.3
133.8
131.6
131.5
131.4
129.2
124.0
122.7
121.4
116.0
115.8
111.4
66.8
36.7
HO2C
O
F
O
S17
13C NMR, CDCl3, 101 MHz
220
200
180
160
140
120
100
80
60
40
20
0
f1 (ppm)

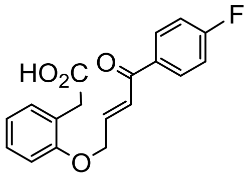

S17

^{19}F NMR, $CDCl_3$, 376 MHz

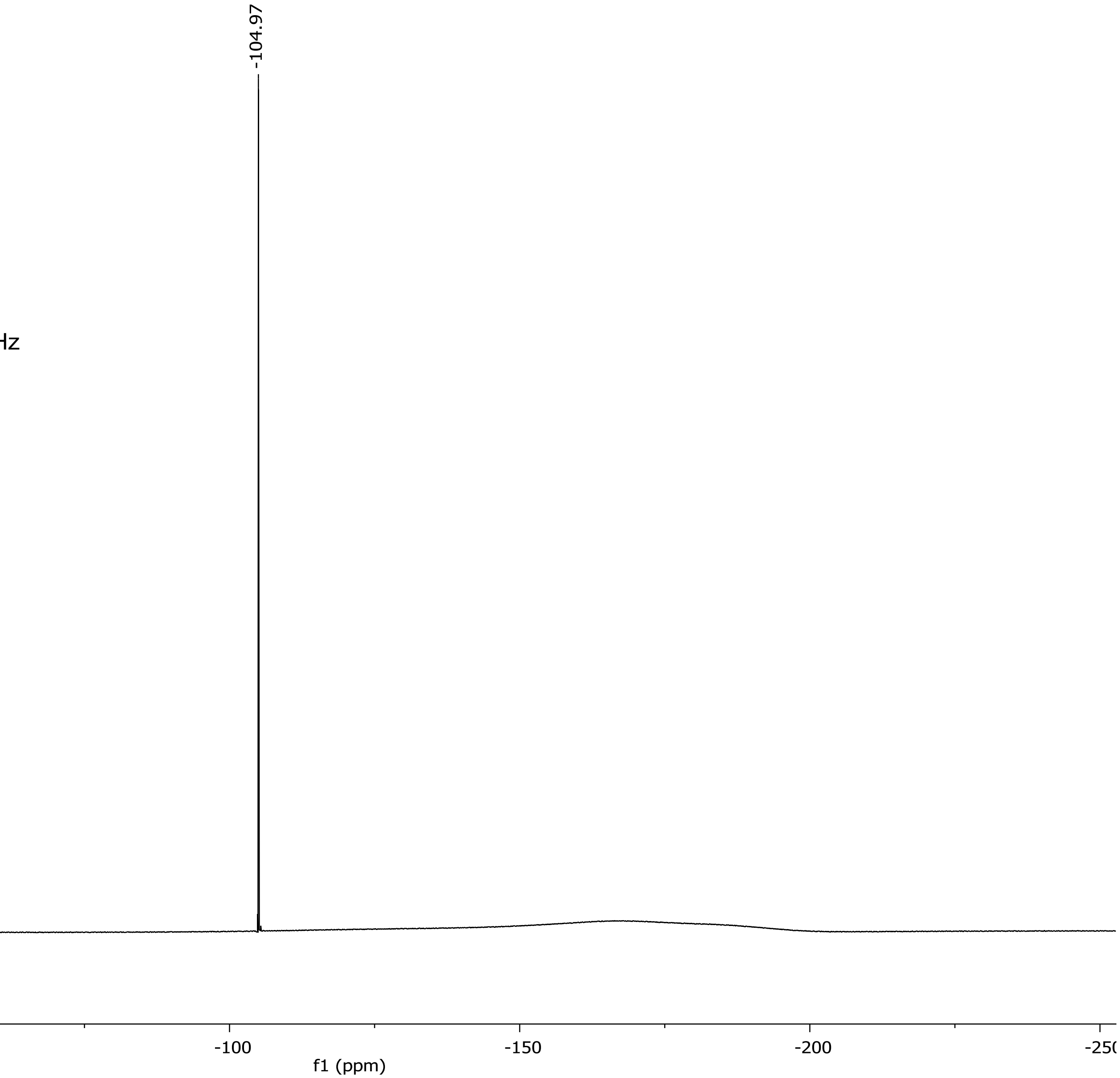

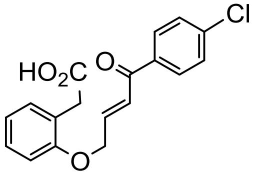

S18

^{1}H NMR, $CDCl_3$, 500 MHz

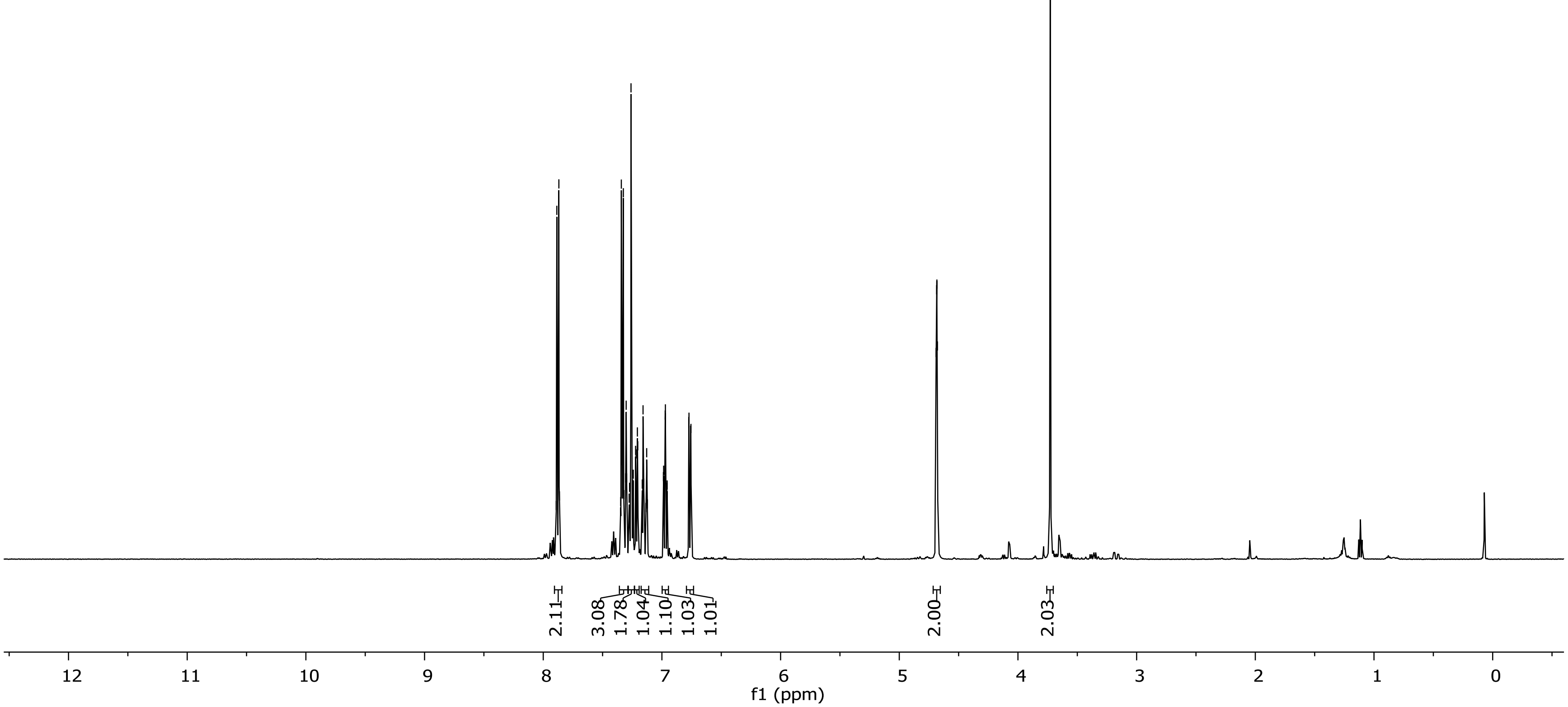

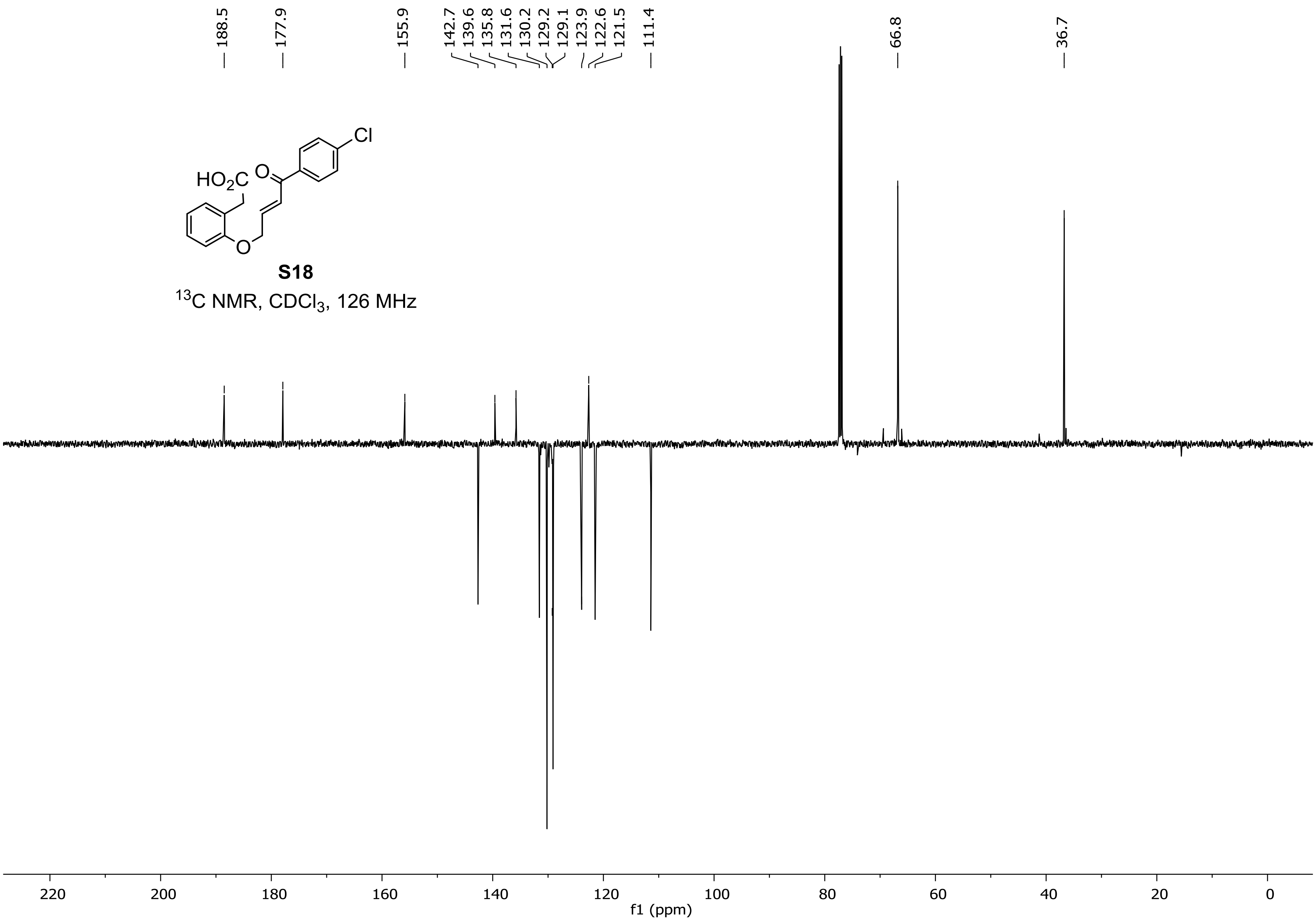
188.5
177.9
155.9
142.7
139.6
135.8
131.6
130.2
129.2
129.1
123.9
122.6
121.5
111.4
66.8
36.7
HO2C
O
Cl
O
S18
13C NMR, CDCl3, 126 MHz
220
200
180
160
140
120
100
80
60
40
20
0
f1 (ppm)

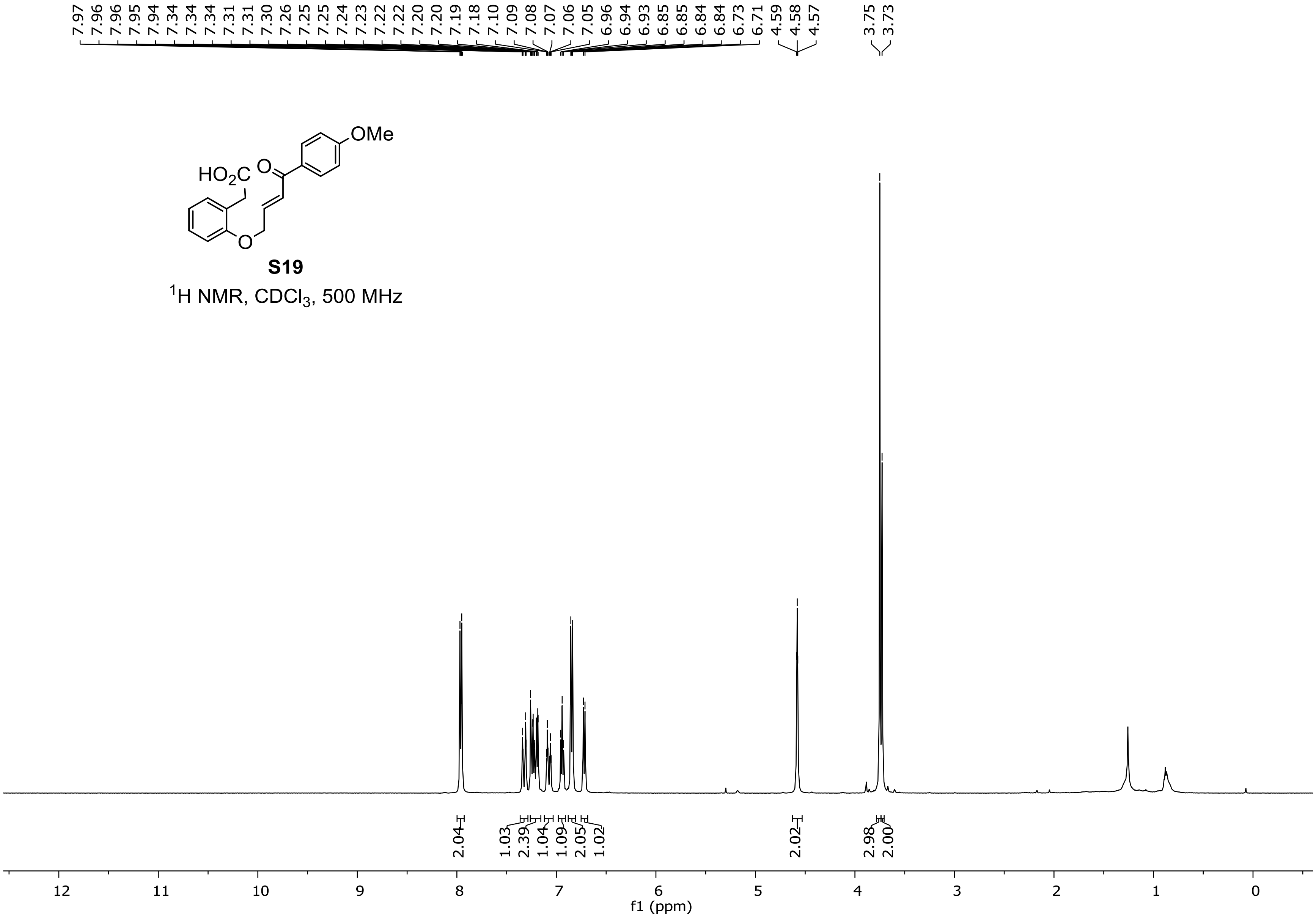
7.97 7.96 7.96 7.95 7.94 7.34 7.34 7.34 7.31 7.31 7.30 7.26 7.25 7.25 7.24 7.23 7.22 7.22 7.20 7.20 7.19 7.18 7.10 7.09 7.08 7.07 7.06 7.05 6.96 6.94 6.93 6.85 6.85 6.84 6.84 6.73 6.71
4.59 4.58 4.57
3.75 3.73
OMe
HO2C
O
O
S19
1H NMR, CDCl3, 500 MHz
2.04 1.03 2.39 1.04 1.09 2.05 1.02 2.02 2.98 2.00
12 11 10 9 8 7 6 5 4 3 2 1 0
f1 (ppm)

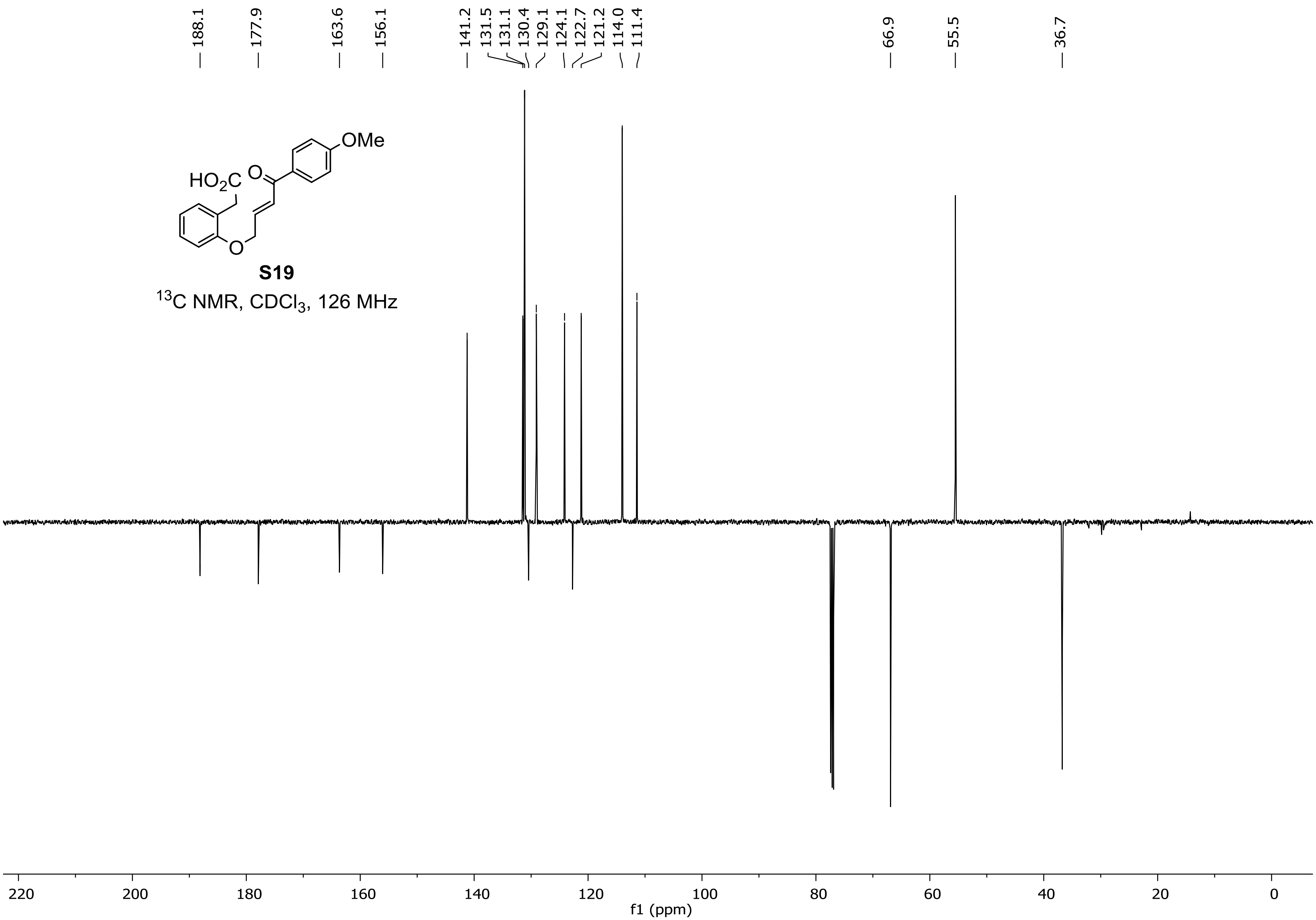
188.1
177.9
163.6
156.1
141.2
131.5
131.1
130.4
129.1
124.1
122.7
121.2
114.0
111.4
66.9
55.5
36.7
HO2C
O
OMe
O
S19
13C NMR, CDCl3, 126 MHz
220
200
180
160
140
120
100
80
60
40
20
0
f1 (ppm)

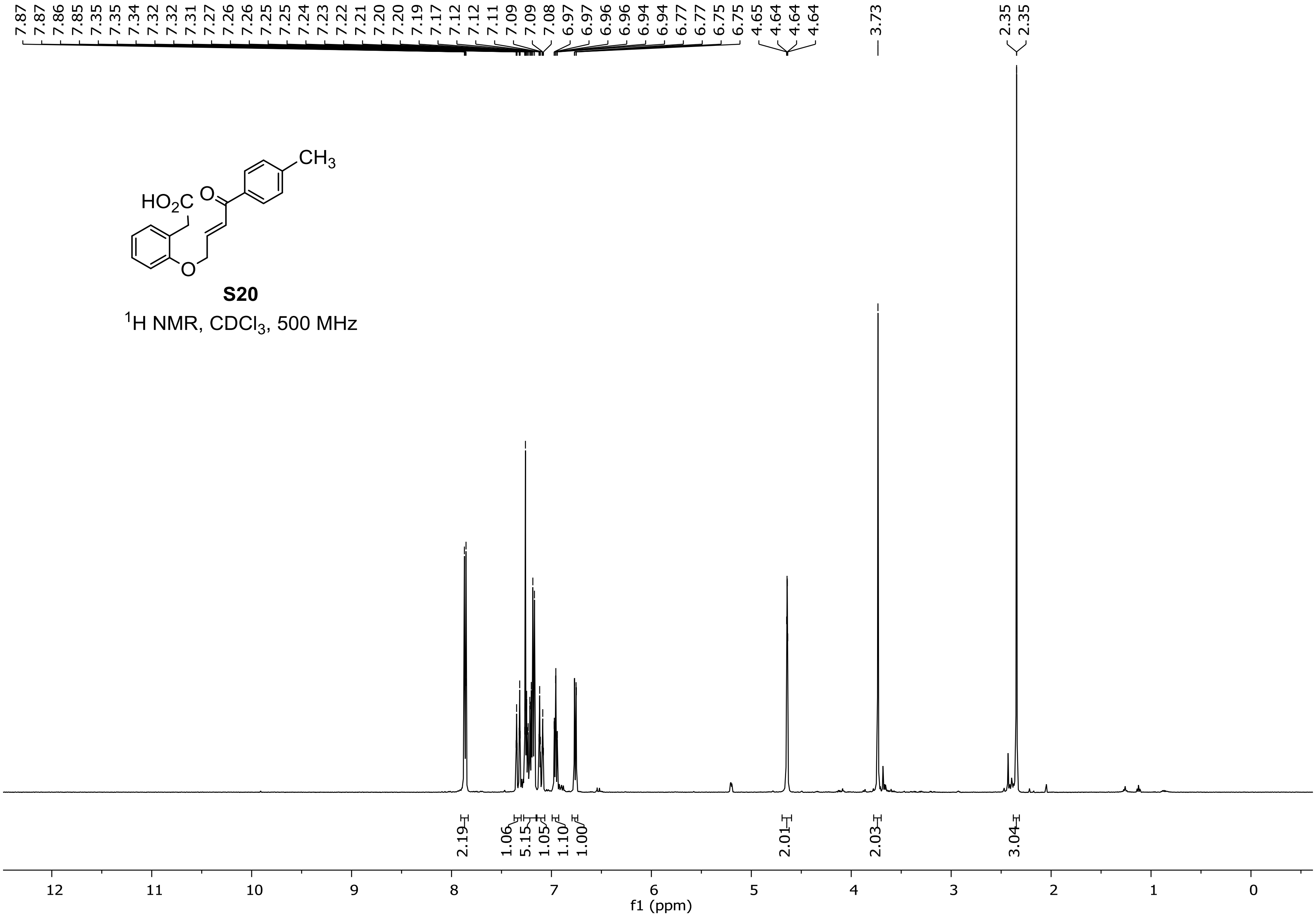
7.87 7.87 7.86 7.85 7.35 7.35 7.34 7.32 7.32 7.31 7.27 7.26 7.26 7.25 7.25 7.24 7.23 7.22 7.21 7.20 7.20 7.19 7.17 7.12 7.12 7.11 7.09 7.09 7.08 6.97 6.97 6.96 6.96 6.94 6.94 6.77 6.77 6.75 6.75 4.65 4.64 4.64 4.64 3.73 2.35 2.35
CH3
HO2C
O
O
S20
1H NMR, CDCl3, 500 MHz
2.19 1.06 5.15 1.05 1.10 1.00 2.01 2.03 3.04
12 11 10 9 8 7 6 5 4 3 2 1 0
f1 (ppm)

S20

^{13}C NMR, $CDCl_3$, 126 MHz

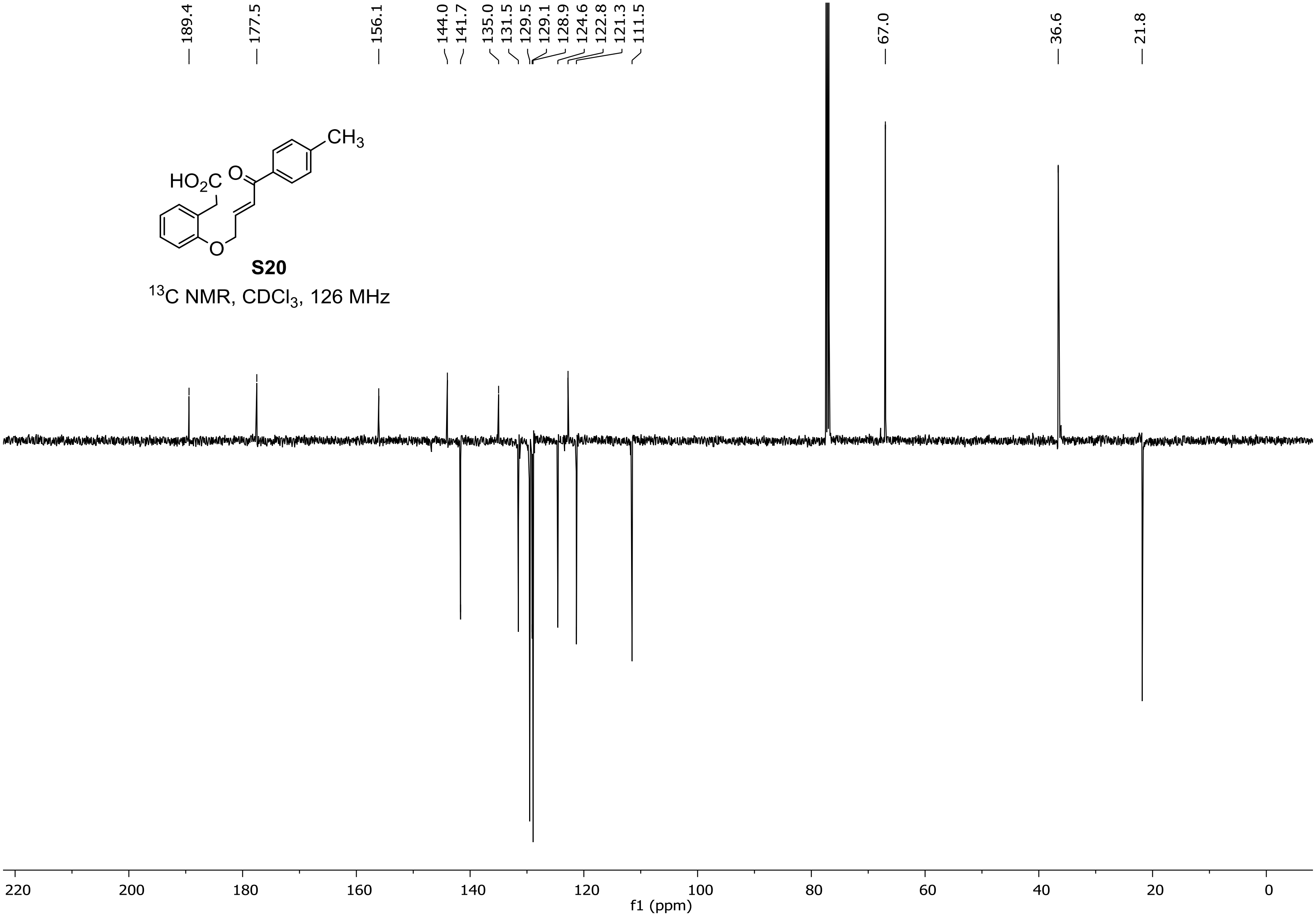

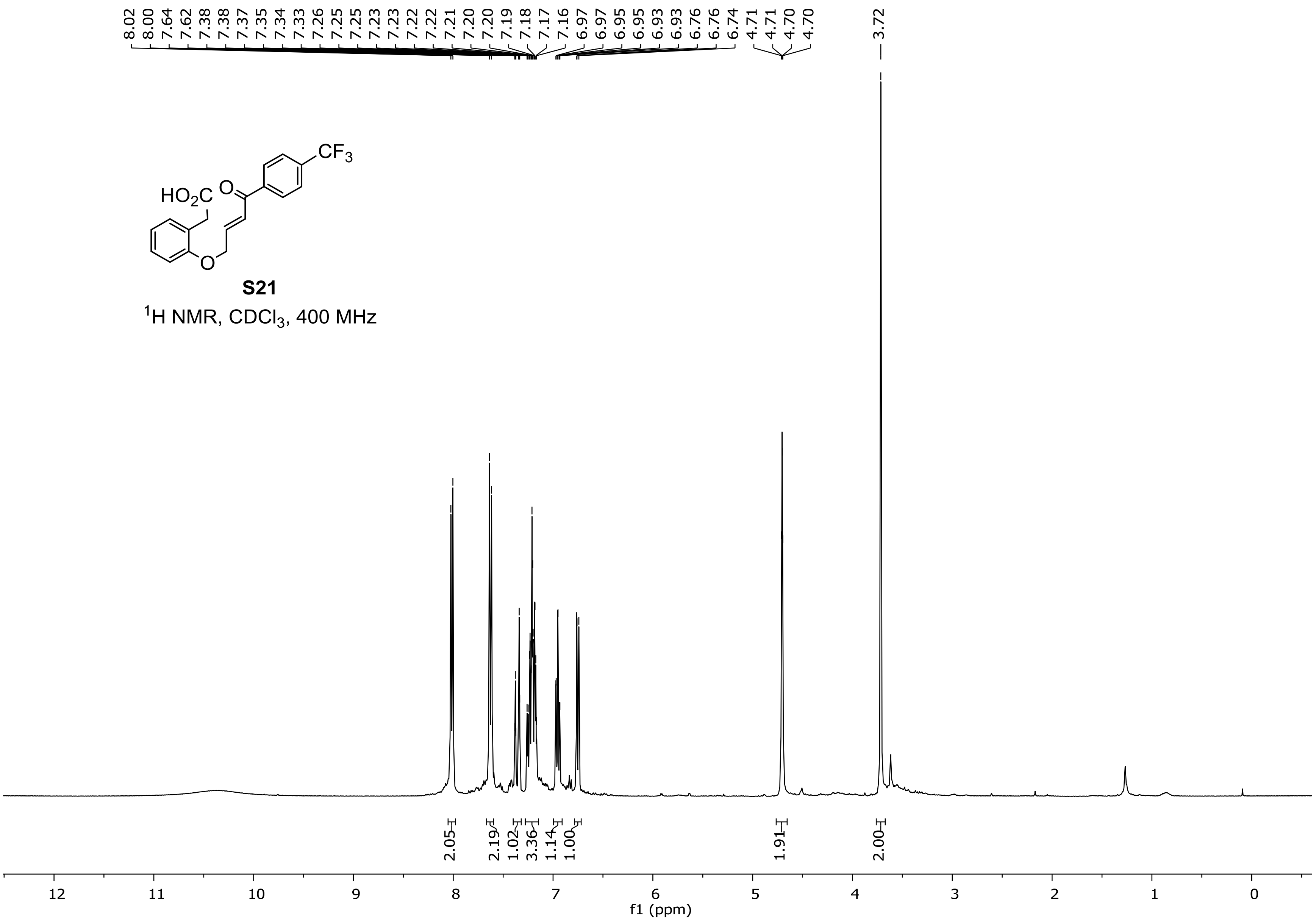
8.02
8.00
7.64
7.62
7.38
7.38
7.37
7.35
7.34
7.33
7.26
7.25
7.25
7.23
7.23
7.22
7.22
7.21
7.20
7.20
7.19
7.18
7.17
7.16
6.97
6.97
6.95
6.95
6.93
6.93
6.76
6.76
6.74
4.71
4.71
4.70
4.70
3.72
CF3
HO2C
O
O
S21
1H NMR, CDCl3, 400 MHz
2.05
2.19
1.02
3.36
1.14
1.00
1.91
2.00
12
11
10
9
8
7
6
5
4
3
2
1
0
f1 (ppm)

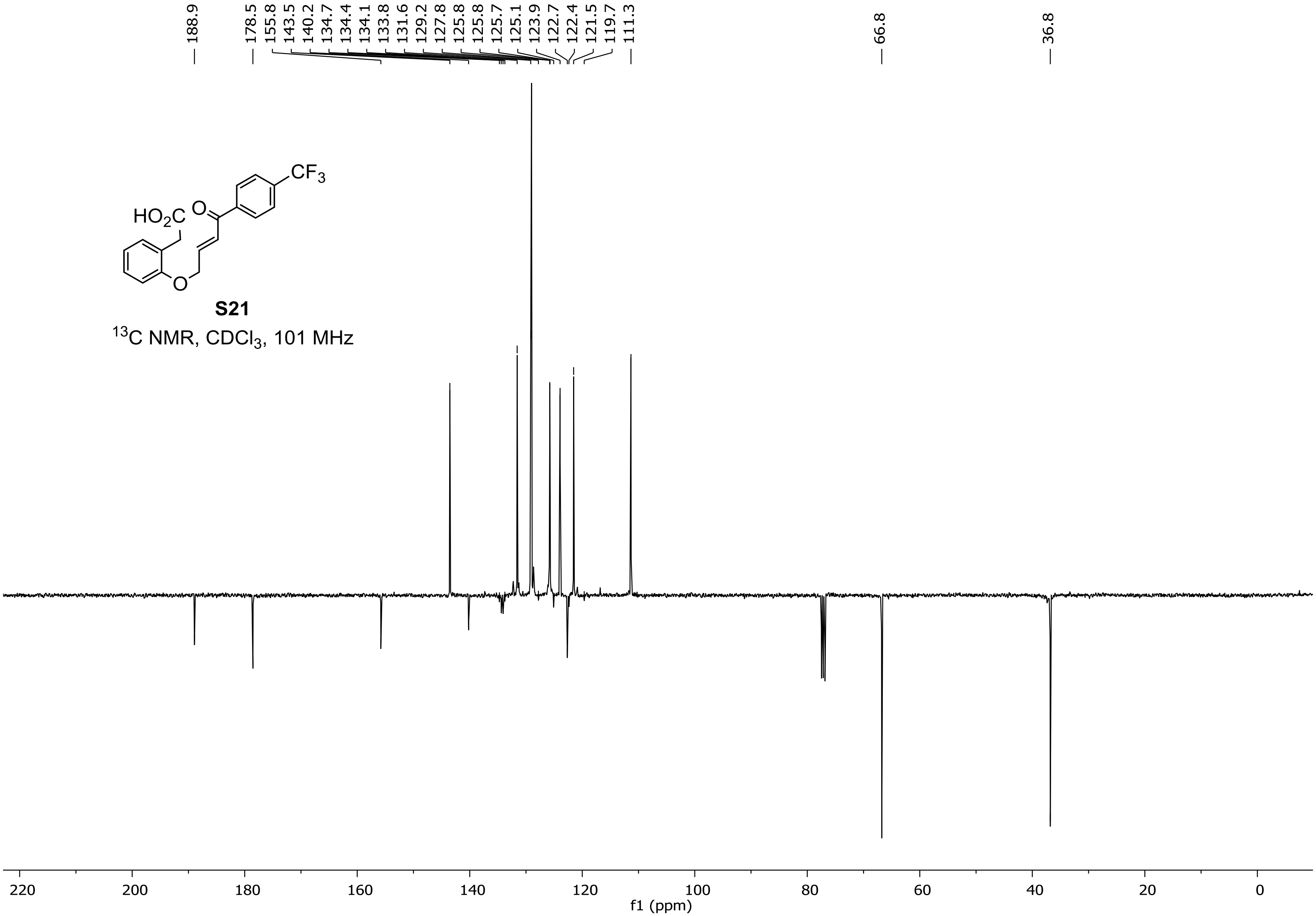

S21

^{13}C NMR, $CDCl_3$, 101 MHz

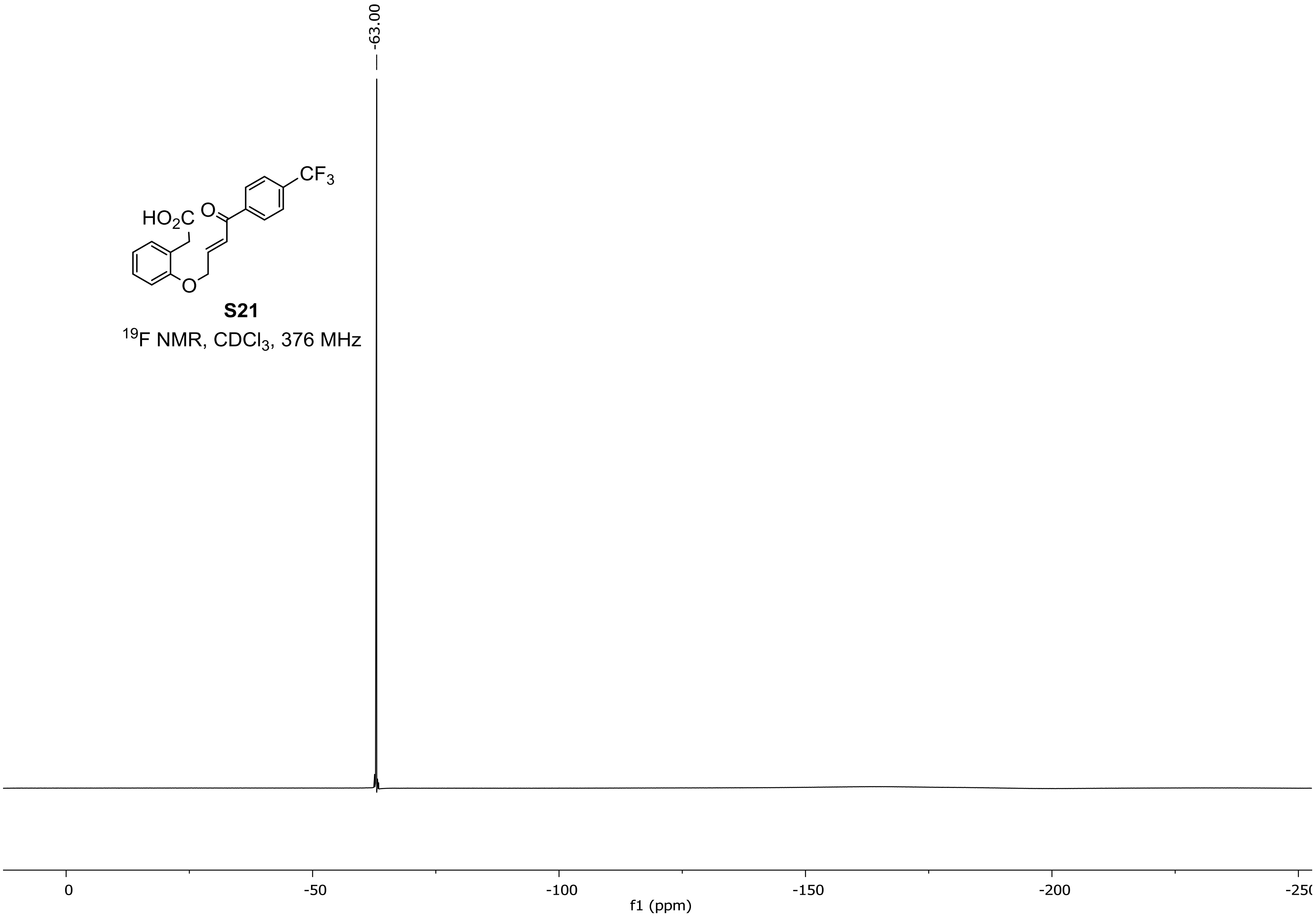
-63.00
CF3
HO2C
O
O
S21
19F NMR, CDCl3, 376 MHz
0
-50
-100
-150
-200
f1 (ppm)

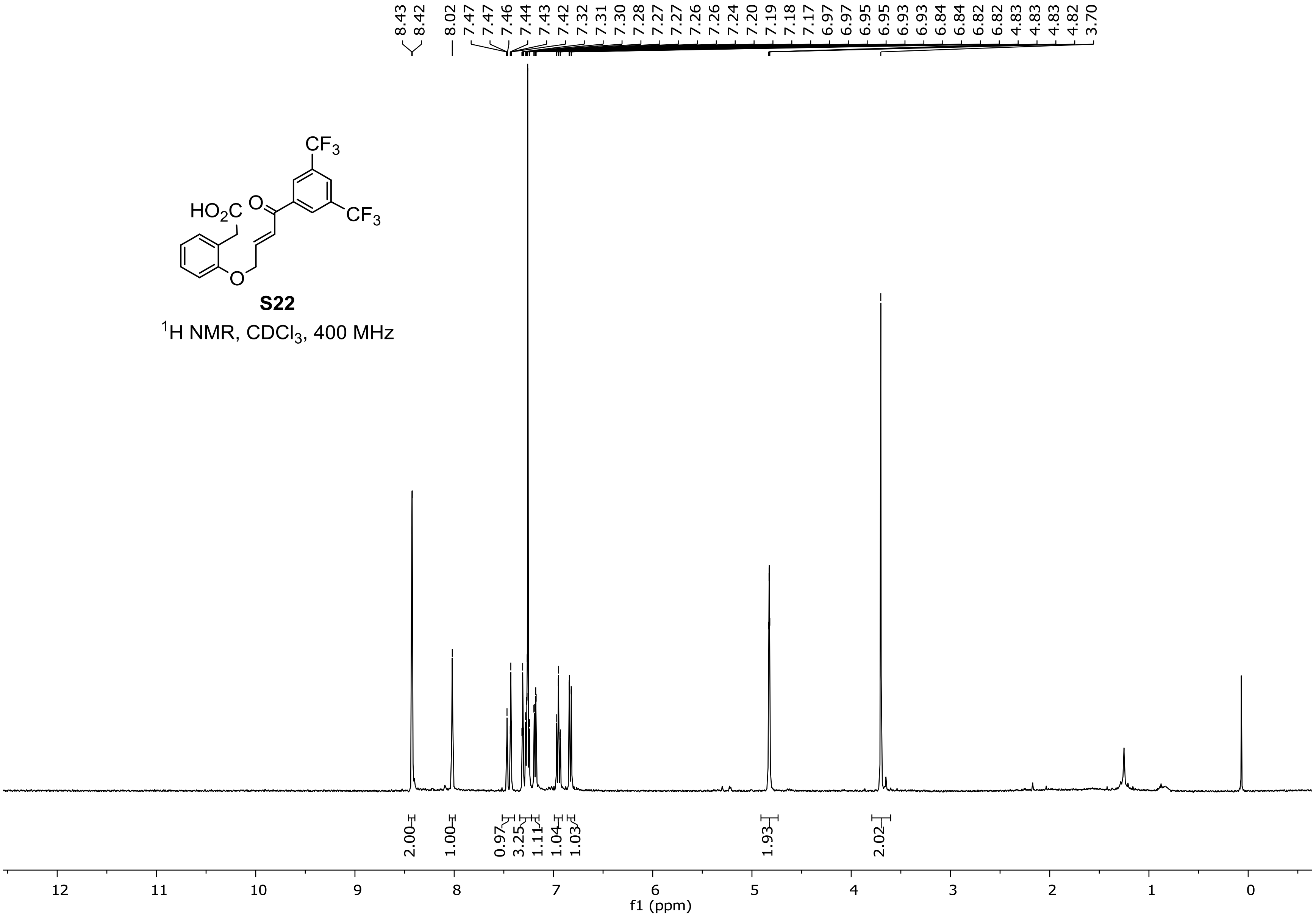

S22

^{1}H NMR, $CDCl_3$, 400 MHz

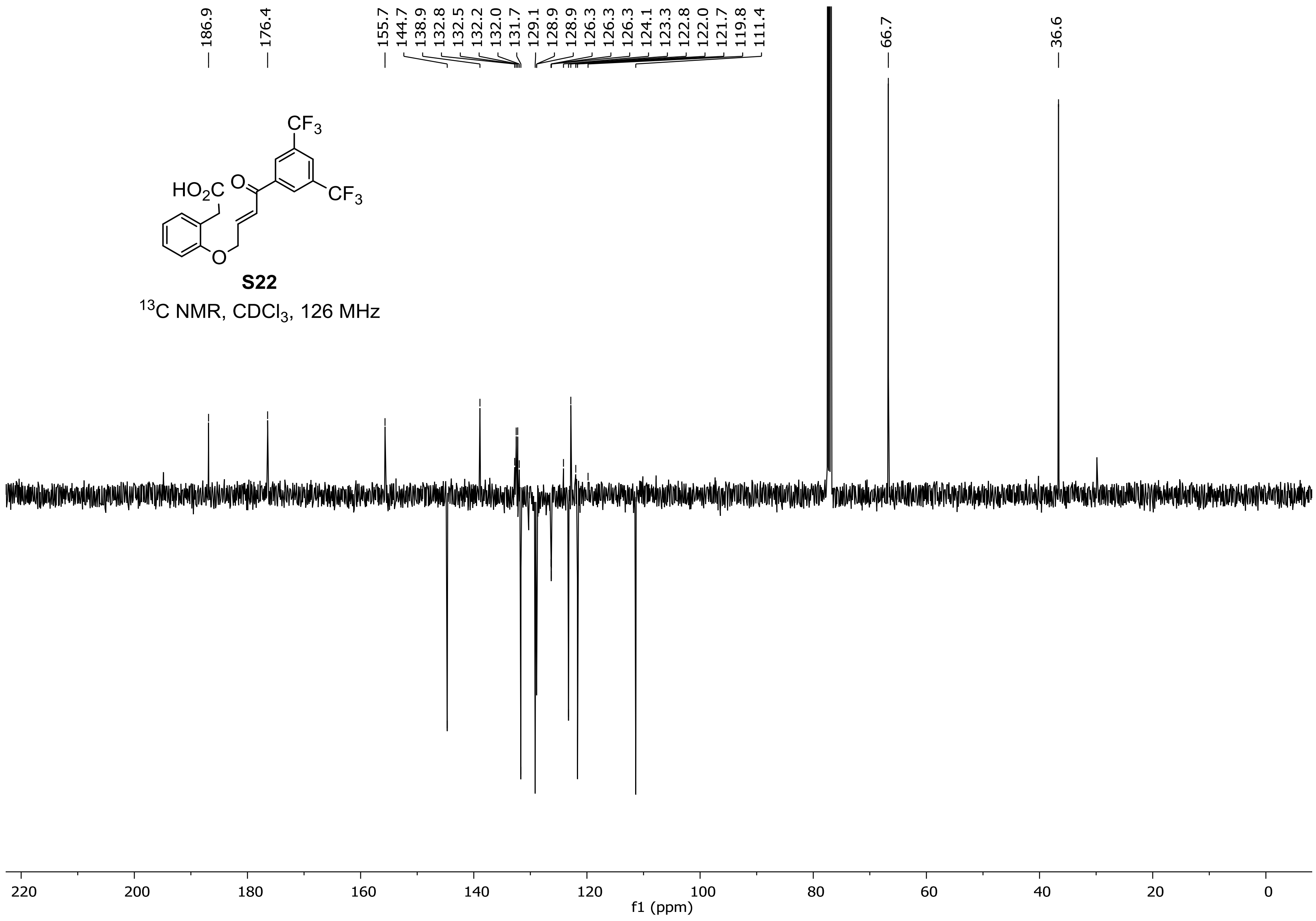

S22

^{13}C NMR, $CDCl_3$, 126 MHz

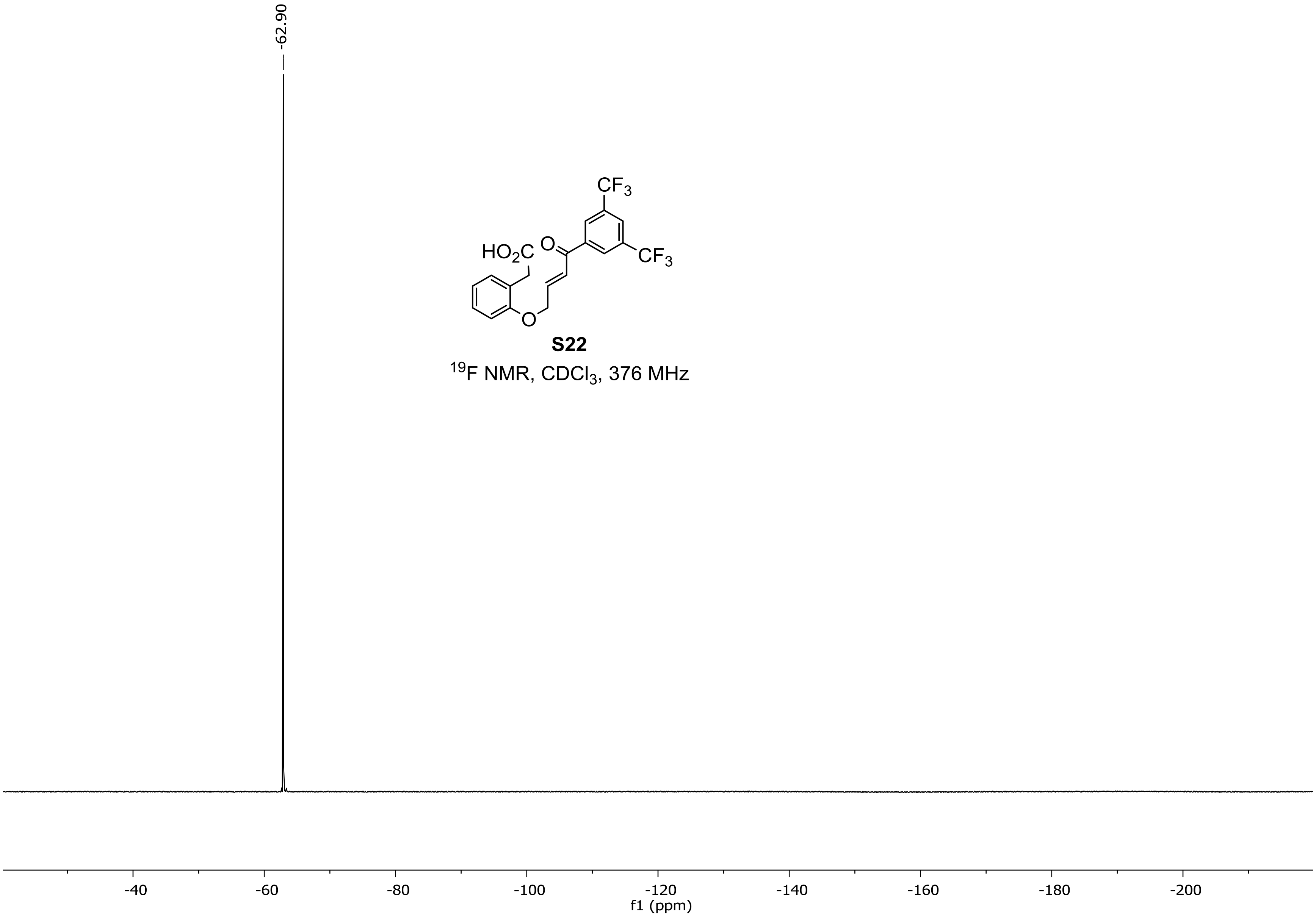

S22

^{19}F NMR, $CDCl_3$, 376 MHz

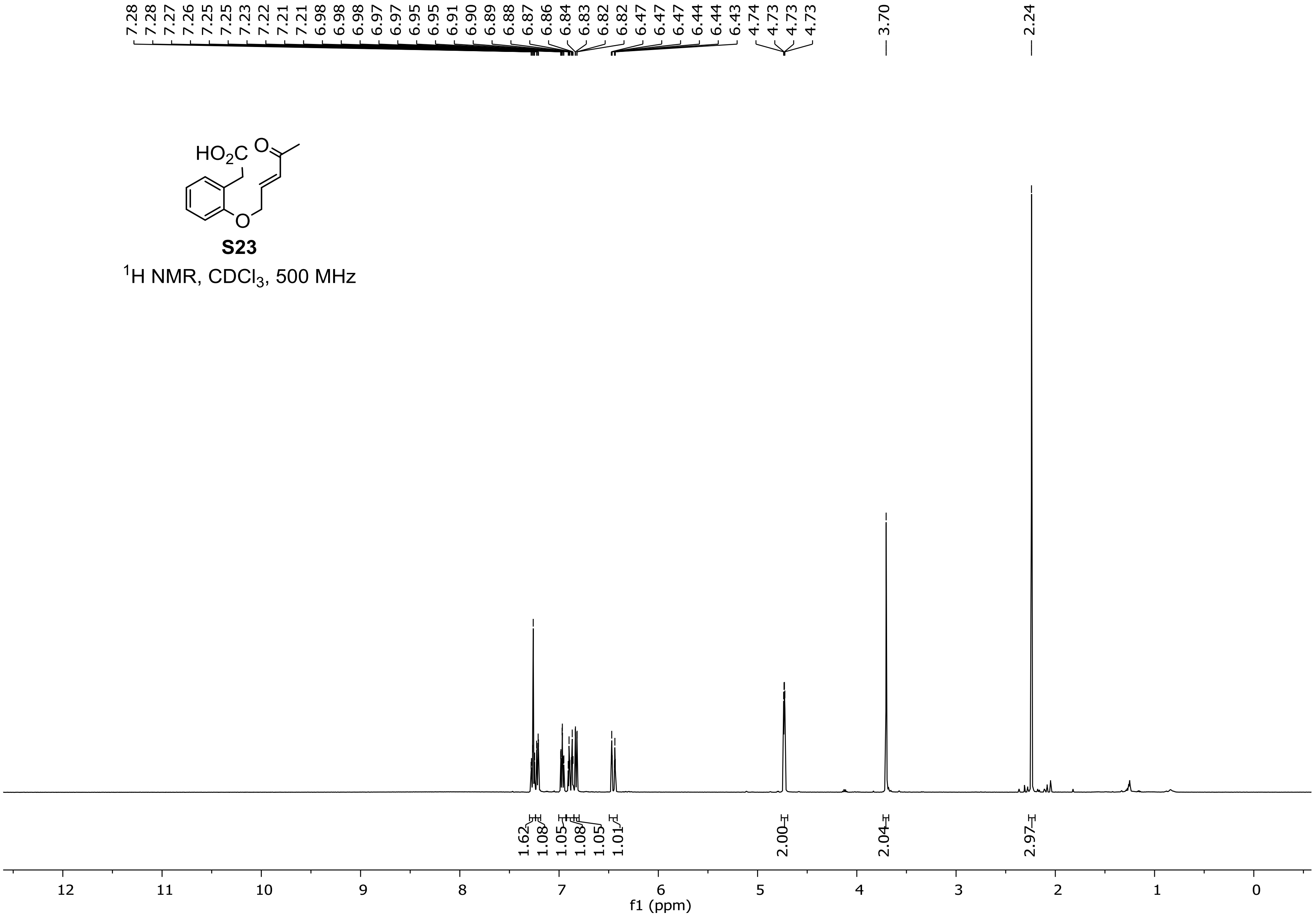
7.28
7.28
7.27
7.26
7.25
7.25
7.23
7.22
7.21
7.21
6.98
6.98
6.98
6.97
6.97
6.95
6.95
6.91
6.90
6.89
6.88
6.87
6.86
6.84
6.83
6.82
6.82
6.47
6.47
6.47
6.44
6.44
6.43
4.74
4.73
4.73
4.73
3.70
2.24
HO2C
O
O
S23
1H NMR, CDCl3, 500 MHz
1.62
1.08
1.05
1.08
1.05
1.01
2.00
2.04
2.97
12
11
10
9
8
7
6
5
4
3
2
1
0
f1 (ppm)

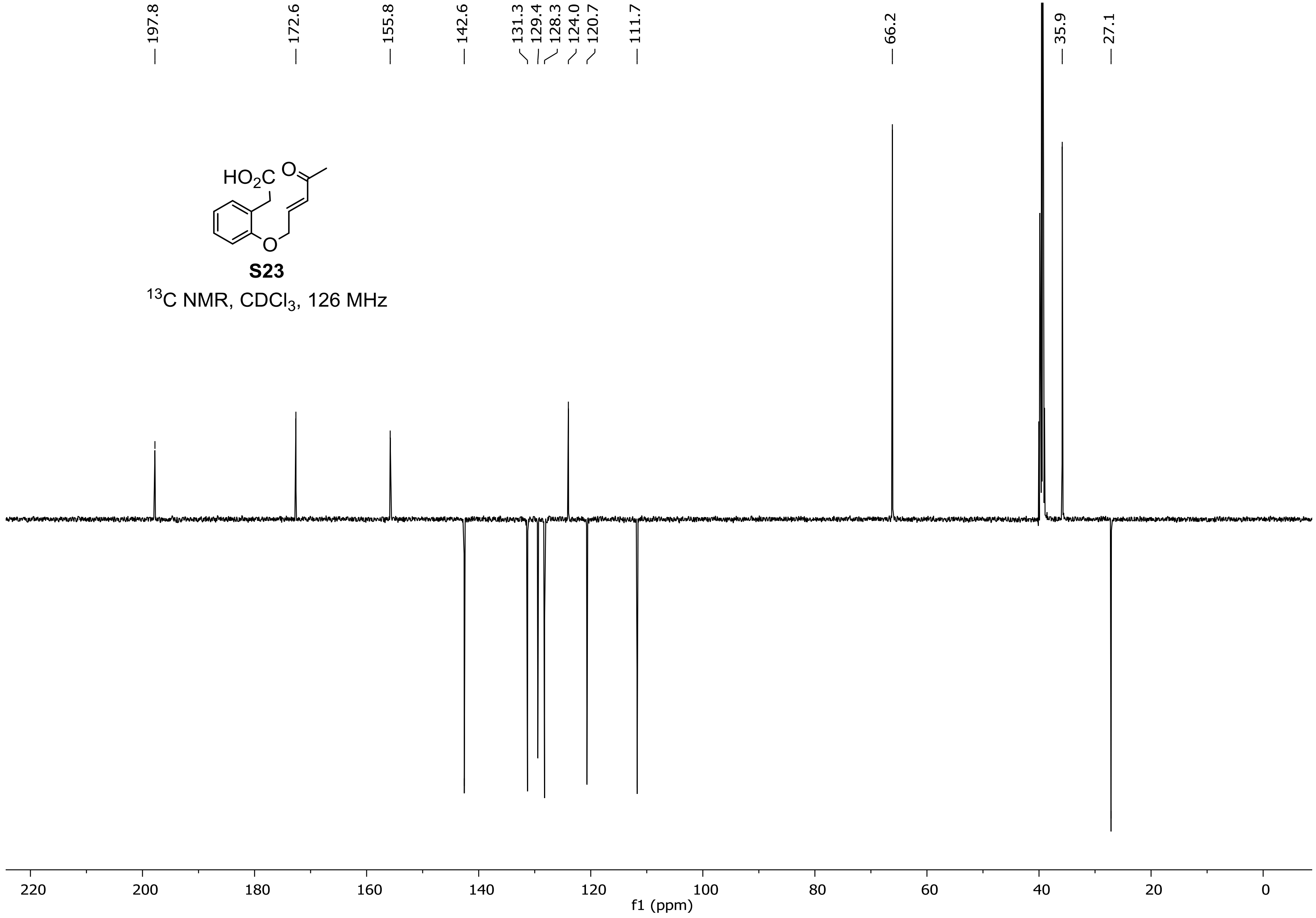
197.8
172.6
155.8
142.6
131.3
129.4
128.3
124.0
120.7
111.7
66.2
35.9
27.1
HO2C
O
O
S23
13C NMR, CDCl3, 126 MHz
220
200
180
160
140
120
100
80
60
40
20
0
f1 (ppm)

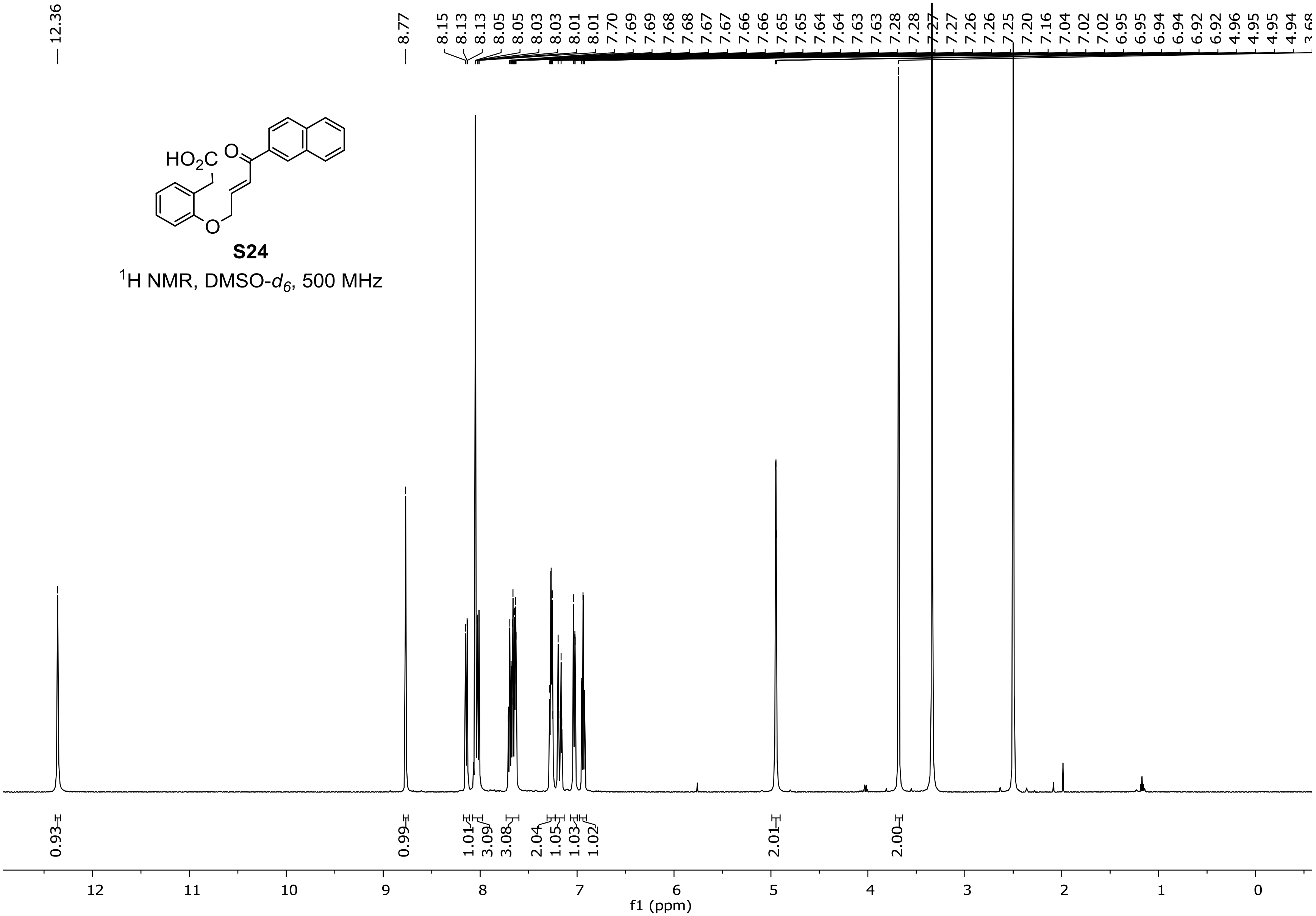
HO2C
O
O
S24
1H NMR, DMSO-d6, 500 MHz
f1 (ppm)

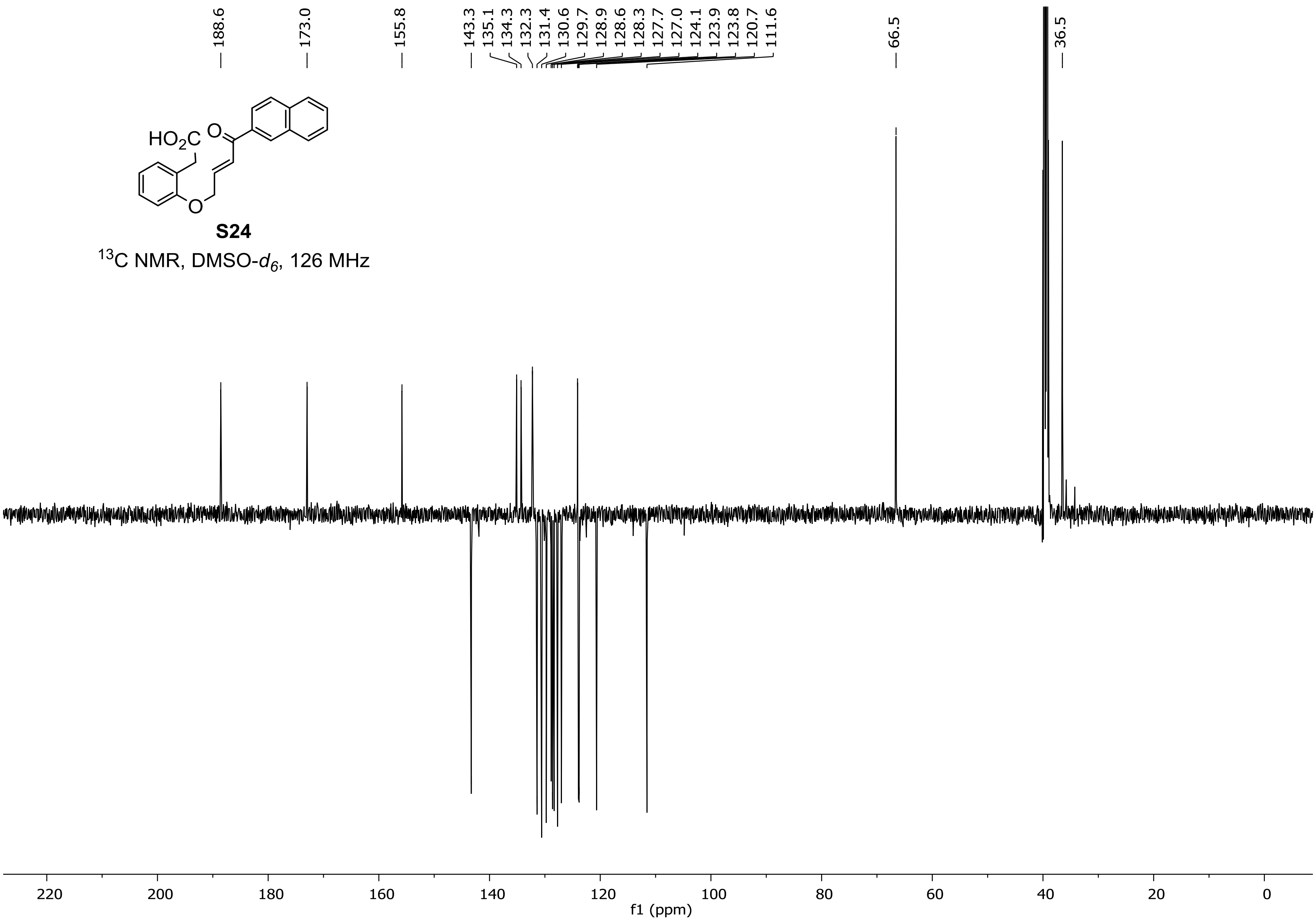
188.6
173.0
155.8
143.3
135.1
134.3
132.3
131.4
130.6
129.7
128.9
128.6
128.3
127.7
127.0
124.1
123.9
123.8
120.7
111.6
66.5
36.5
HO2C
O
O
S24
13C NMR, DMSO-d6, 126 MHz
220
200
180
160
140
120
100
80
60
40
20
0
f1 (ppm)

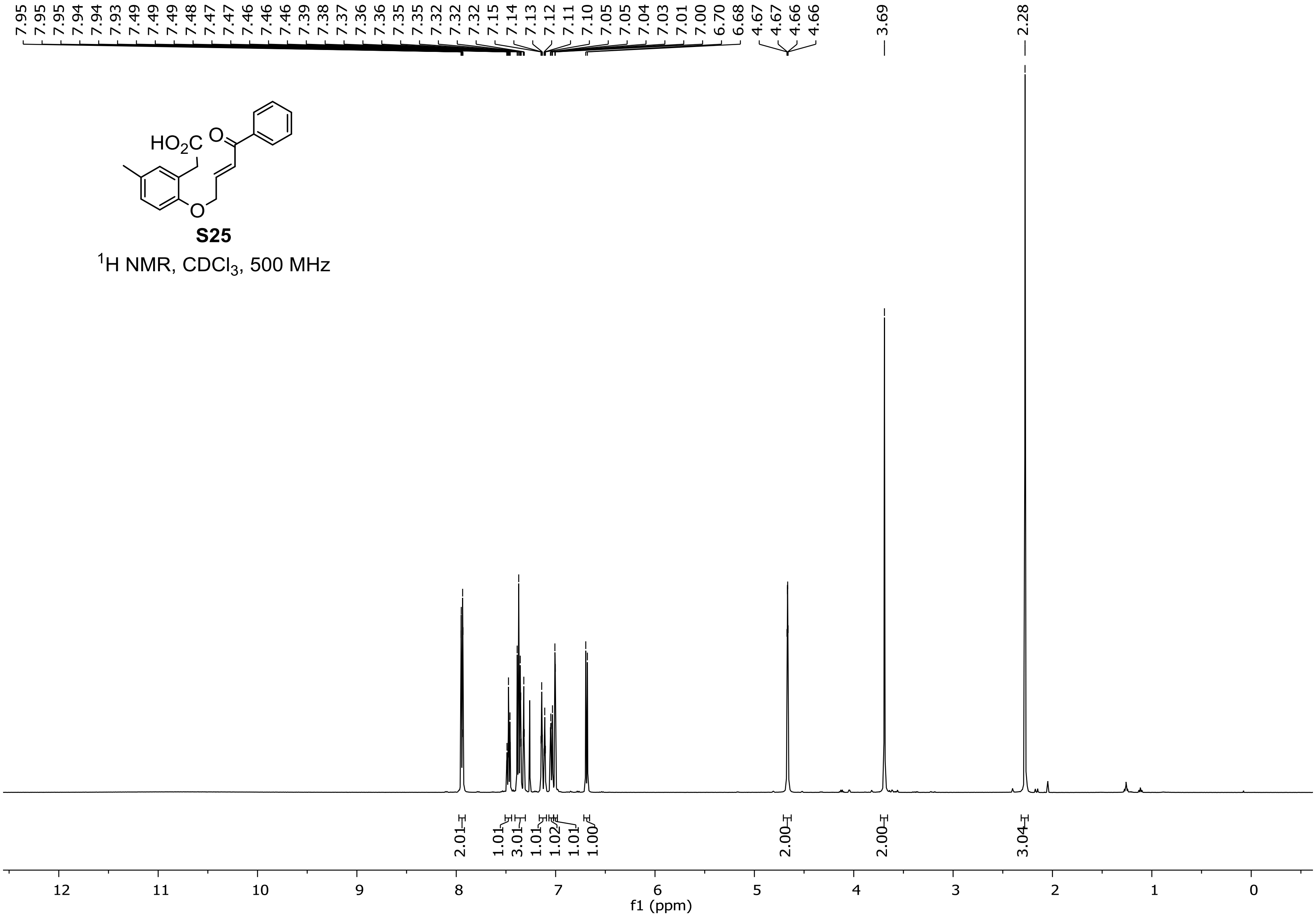

HO2C
O
O
S25
1H NMR, CDCl3, 500 MHz
7.95
7.95
7.95
7.94
7.94
7.93
7.49
7.49
7.49
7.48
7.47
7.47
7.46
7.46
7.46
7.39
7.38
7.37
7.36
7.36
7.35
7.35
7.32
7.32
7.32
7.15
7.14
7.13
7.12
7.11
7.10
7.05
7.05
7.04
7.03
7.01
7.00
6.70
6.68
4.67
4.67
4.66
4.66
3.69
2.28
2.01
1.01
3.01
1.01
1.02
1.01
1.00
2.00
2.00
3.04
f1 (ppm)

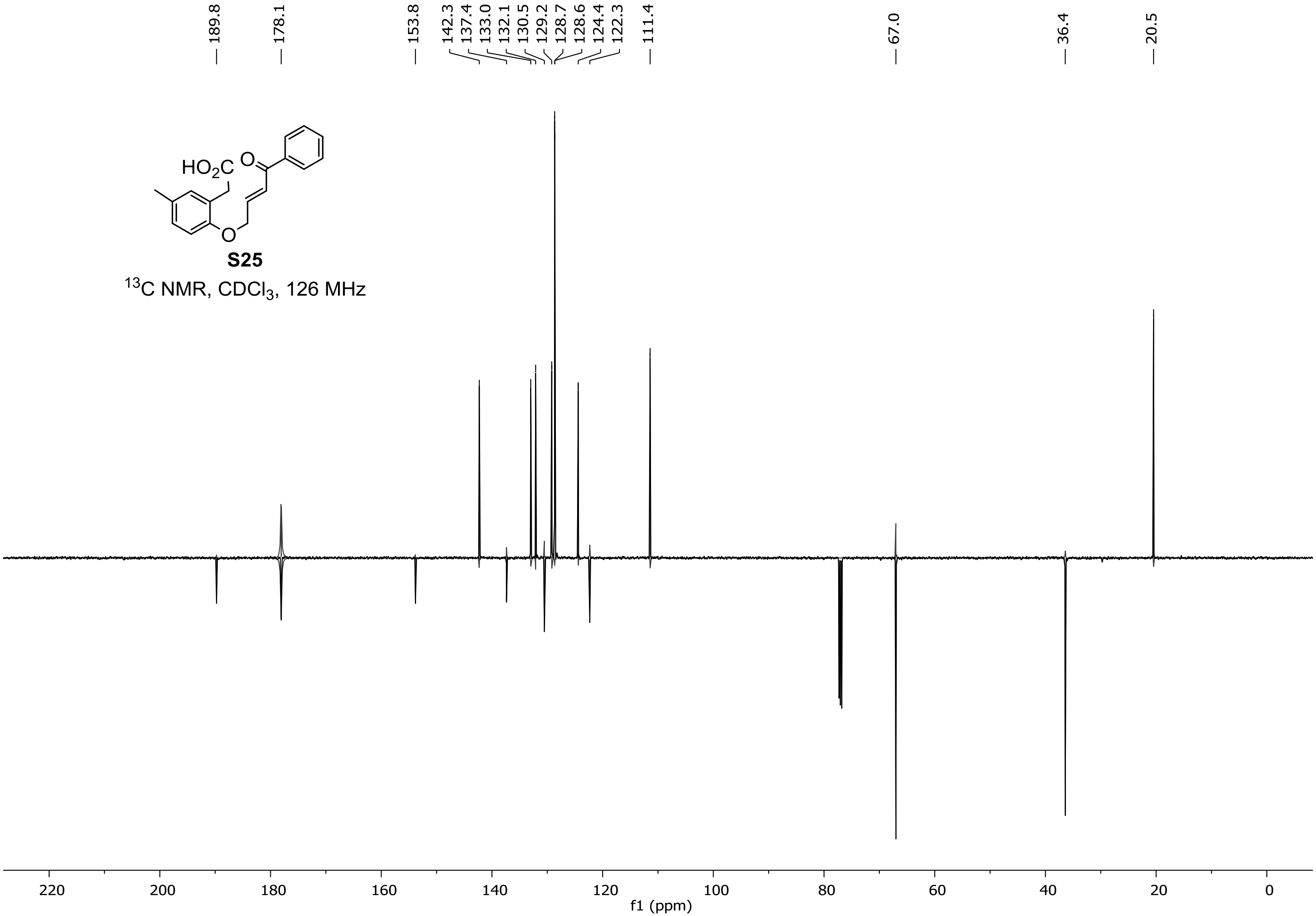
189.8
178.1
153.8
142.3
137.4
133.0
132.1
130.5
129.2
128.7
128.6
124.4
122.3
111.4
67.0
36.4
20.5
HO2C
O
O
S25
13C NMR, CDCl3, 126 MHz
220
200
180
160
140
120
100
80
60
40
20
0
f1 (ppm)

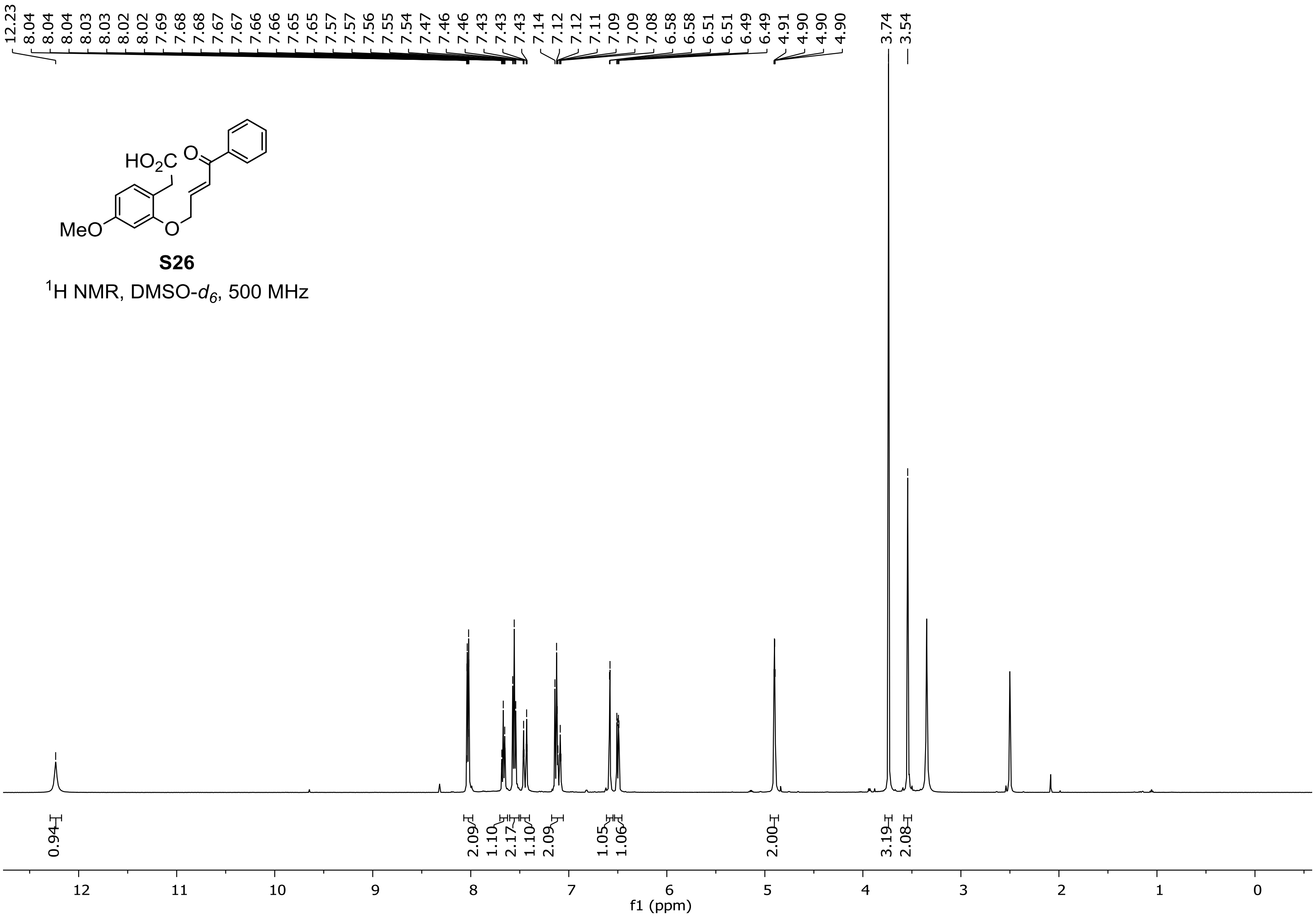
12.23 8.04 8.04 8.04 8.03 8.03 8.02 8.02 7.69 7.68 7.68 7.67 7.67 7.66 7.66 7.65 7.65 7.57 7.57 7.56 7.55 7.54 7.47 7.46 7.46 7.43 7.43 7.43 7.14 7.12 7.12 7.11 7.09 7.09 7.08 6.58 6.58 6.51 6.51 6.49 6.49 4.91 4.90 4.90 4.90 3.74 3.54
HO2C
O
MeO
O
S26
1H NMR, DMSO-d6, 500 MHz
0.94 2.09 1.10 2.17 1.10 2.09 1.05 1.06 2.00 3.19 2.08
12 11 10 9 8 7 6 5 4 3 2 1 0
f1 (ppm)

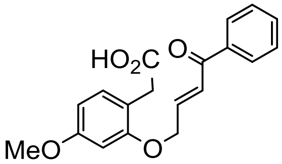

S26

^{13}C NMR, DMSO-d_6, 126 MHz

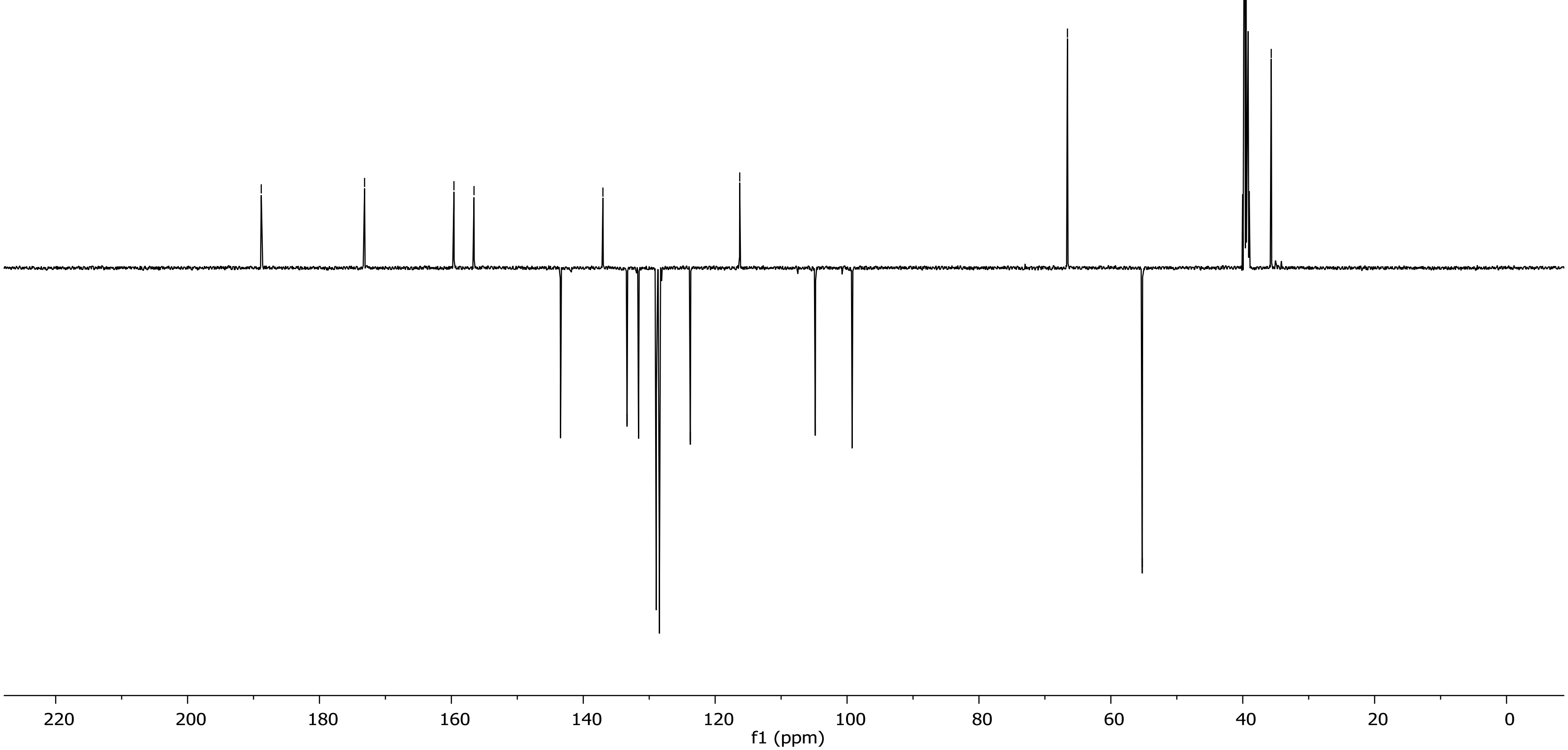

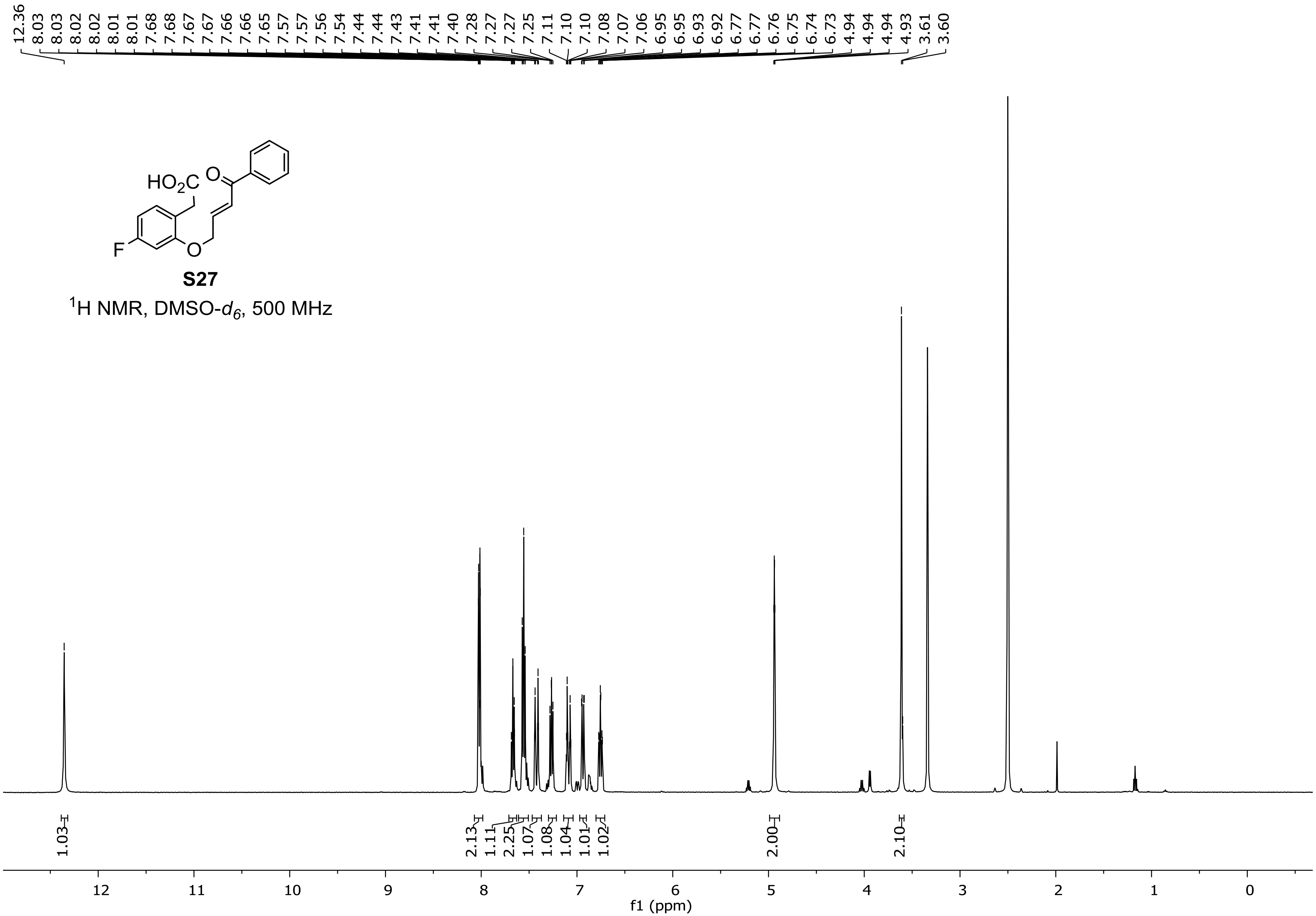
HO2C
O
F
O
S27
1H NMR, DMSO-d6, 500 MHz
12.36
8.03
8.03
8.02
8.02
8.01
8.01
7.68
7.68
7.67
7.67
7.66
7.66
7.65
7.57
7.57
7.56
7.54
7.44
7.44
7.43
7.41
7.41
7.40
7.28
7.27
7.27
7.25
7.11
7.10
7.10
7.08
7.07
7.06
6.95
6.95
6.93
6.92
6.77
6.77
6.76
6.75
6.74
6.73
4.94
4.94
4.94
4.93
3.61
3.60
1.03
2.13
1.11
2.25
1.07
1.08
1.04
1.01
1.02
2.00
2.10
12
11
10
9
8
7
6
5
4
3
2
1
0
f1 (ppm)

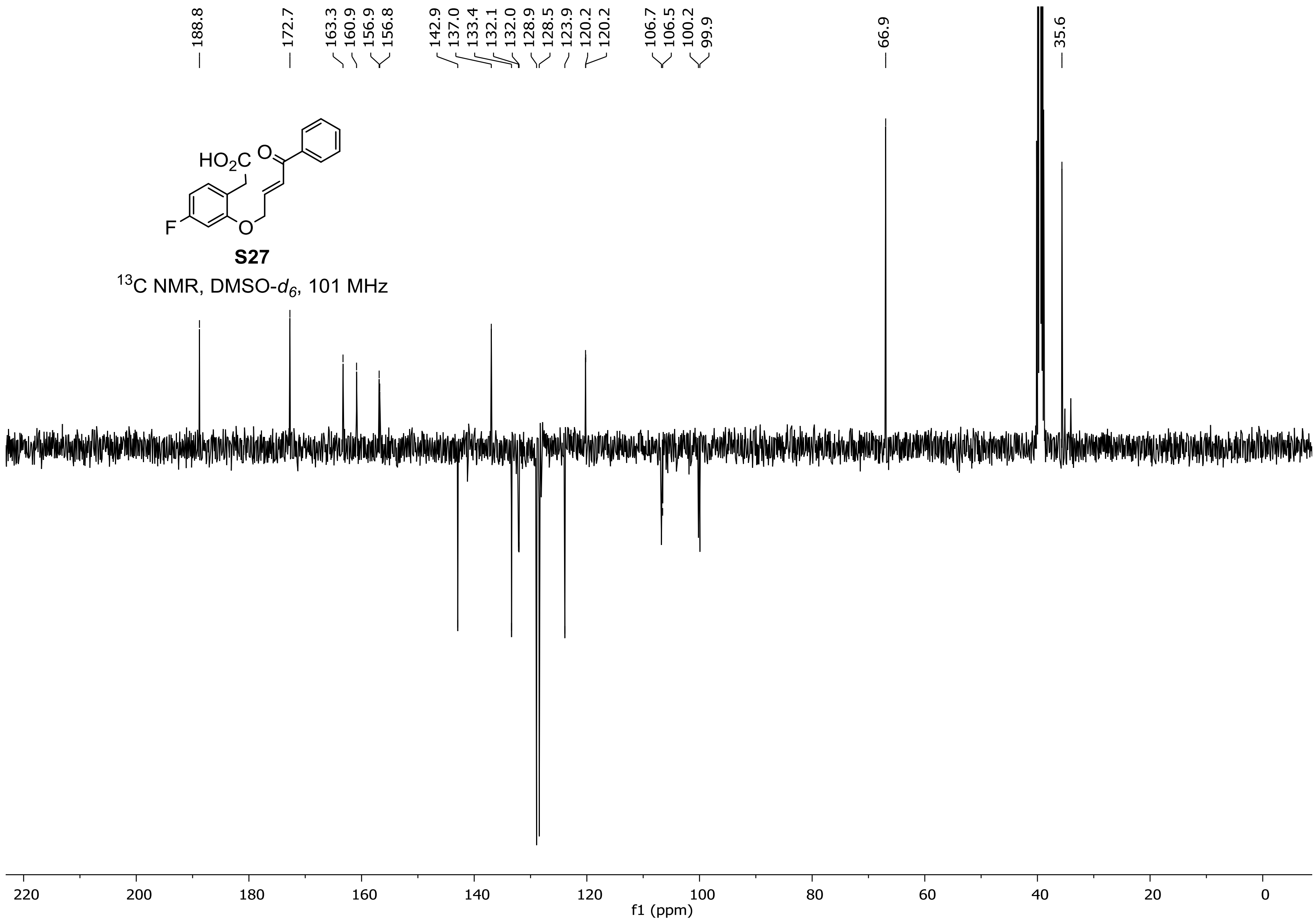
188.8
172.7
163.3
160.9
156.9
156.8
142.9
137.0
133.4
132.1
132.0
128.9
128.5
123.9
120.2
120.2
106.7
106.5
100.2
99.9
66.9
35.6
HO2C
O
F
O
S27
13C NMR, DMSO-d6, 101 MHz
220
200
180
160
140
120
100
80
60
40
20
0
f1 (ppm)

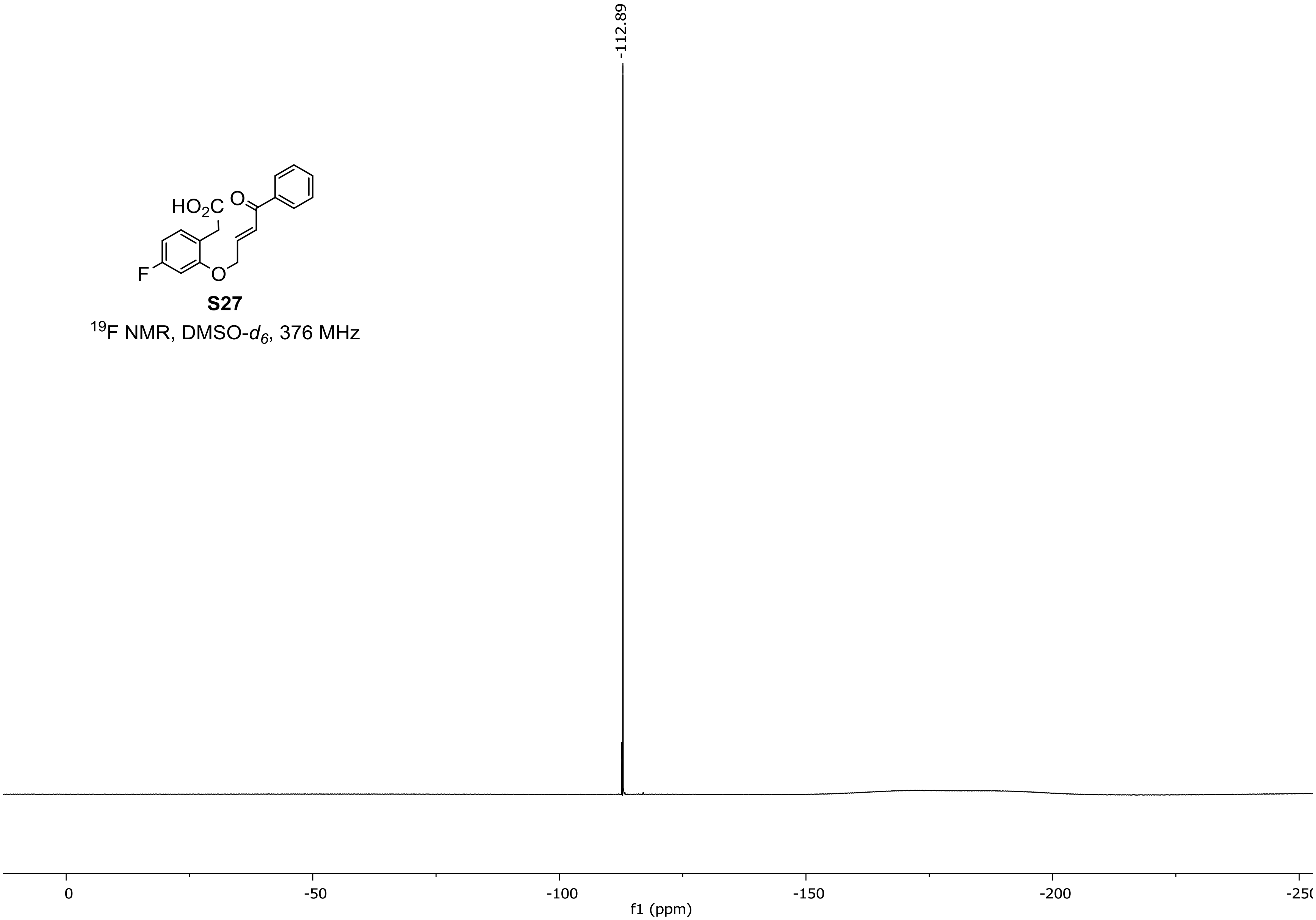

S27

^{19}F NMR, DMSO-d_6, 376 MHz

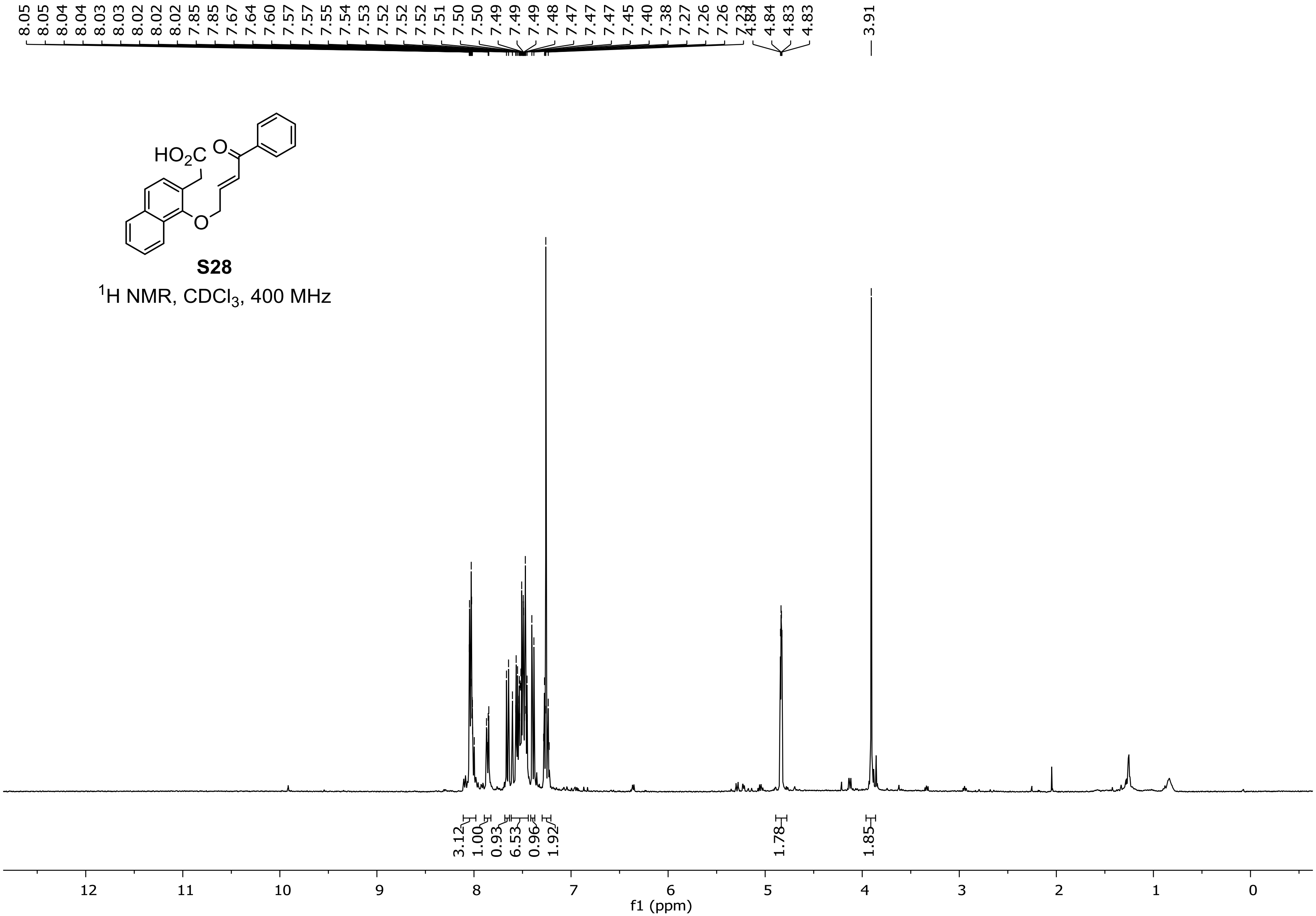

S28

^{1}H NMR, $CDCl_3$, 400 MHz

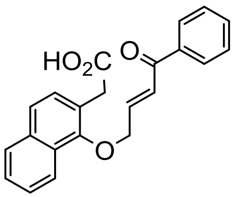

S28

^{13}C NMR, $CDCl_3$, 101 MHz

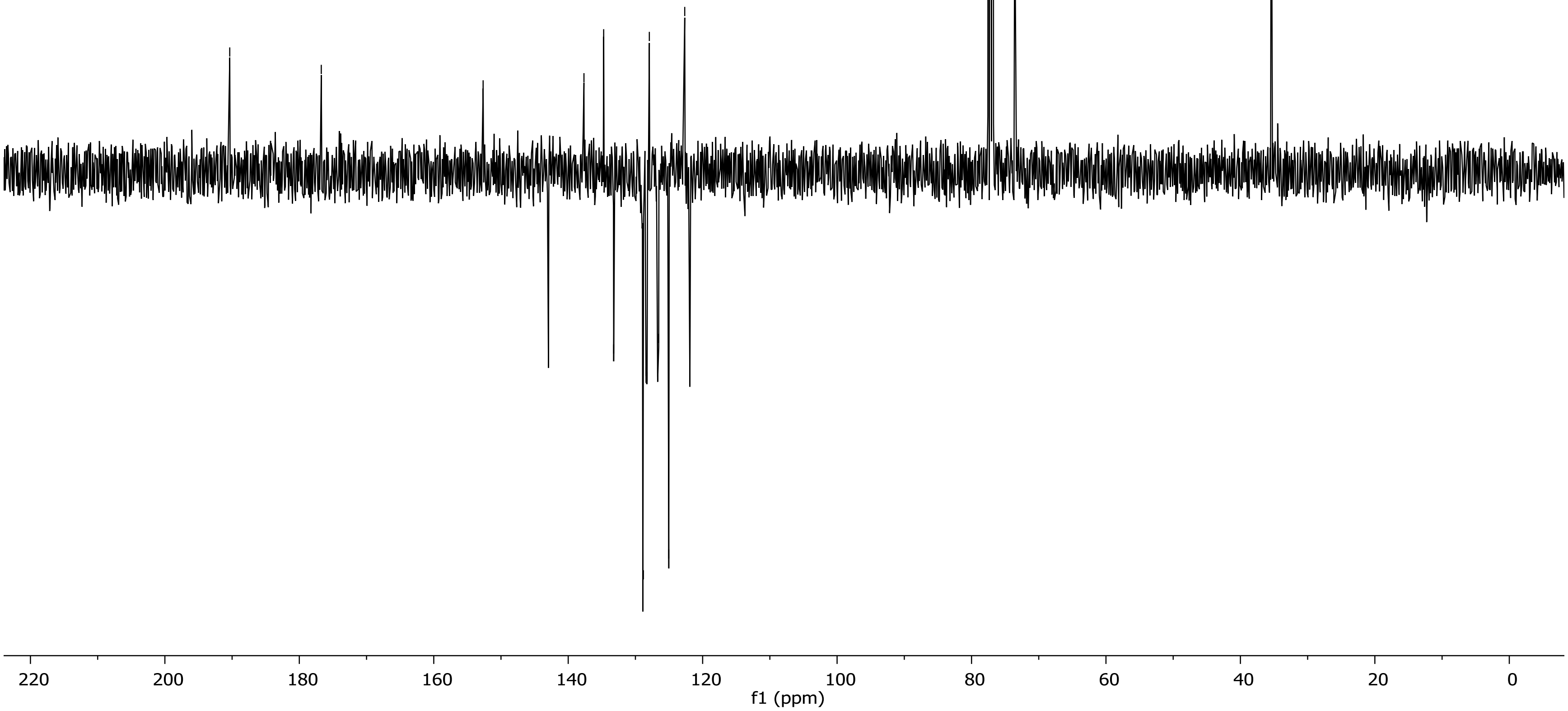

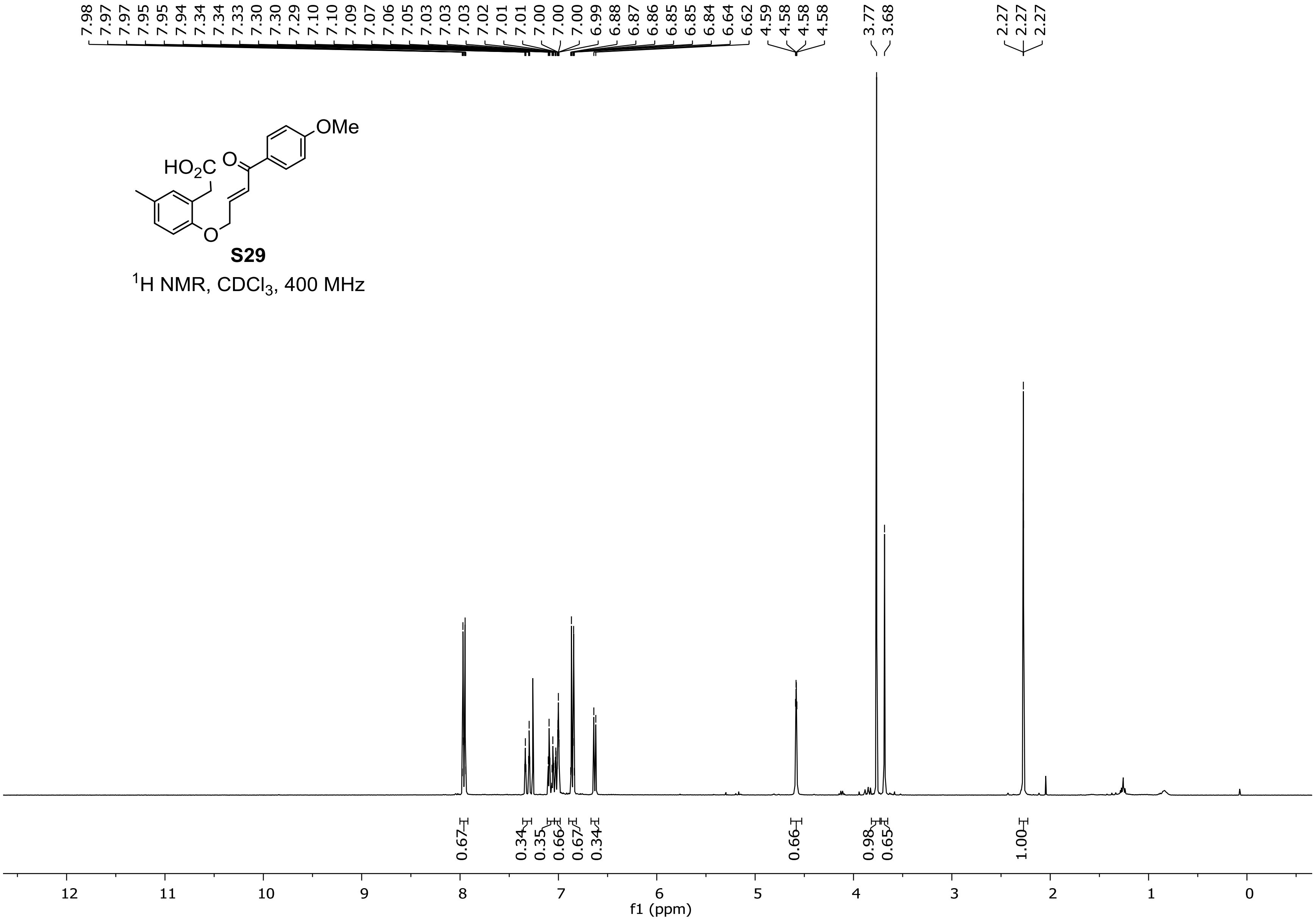
7.98
7.97
7.97
7.95
7.95
7.94
7.34
7.34
7.33
7.30
7.30
7.29
7.10
7.10
7.09
7.07
7.06
7.05
7.03
7.03
7.03
7.02
7.01
7.01
7.00
7.00
7.00
6.99
6.88
6.87
6.86
6.85
6.85
6.84
6.64
6.62
4.59
4.58
4.58
4.58
3.77
3.68
2.27
2.27
2.27
OMe
HO2C
O
O
S29
1H NMR, CDCl3, 400 MHz
0.67
0.34
0.35
0.66
0.67
0.34
0.66
0.98
0.65
1.00
12
11
10
9
8
7
6
5
4
3
2
1
0
f1 (ppm)

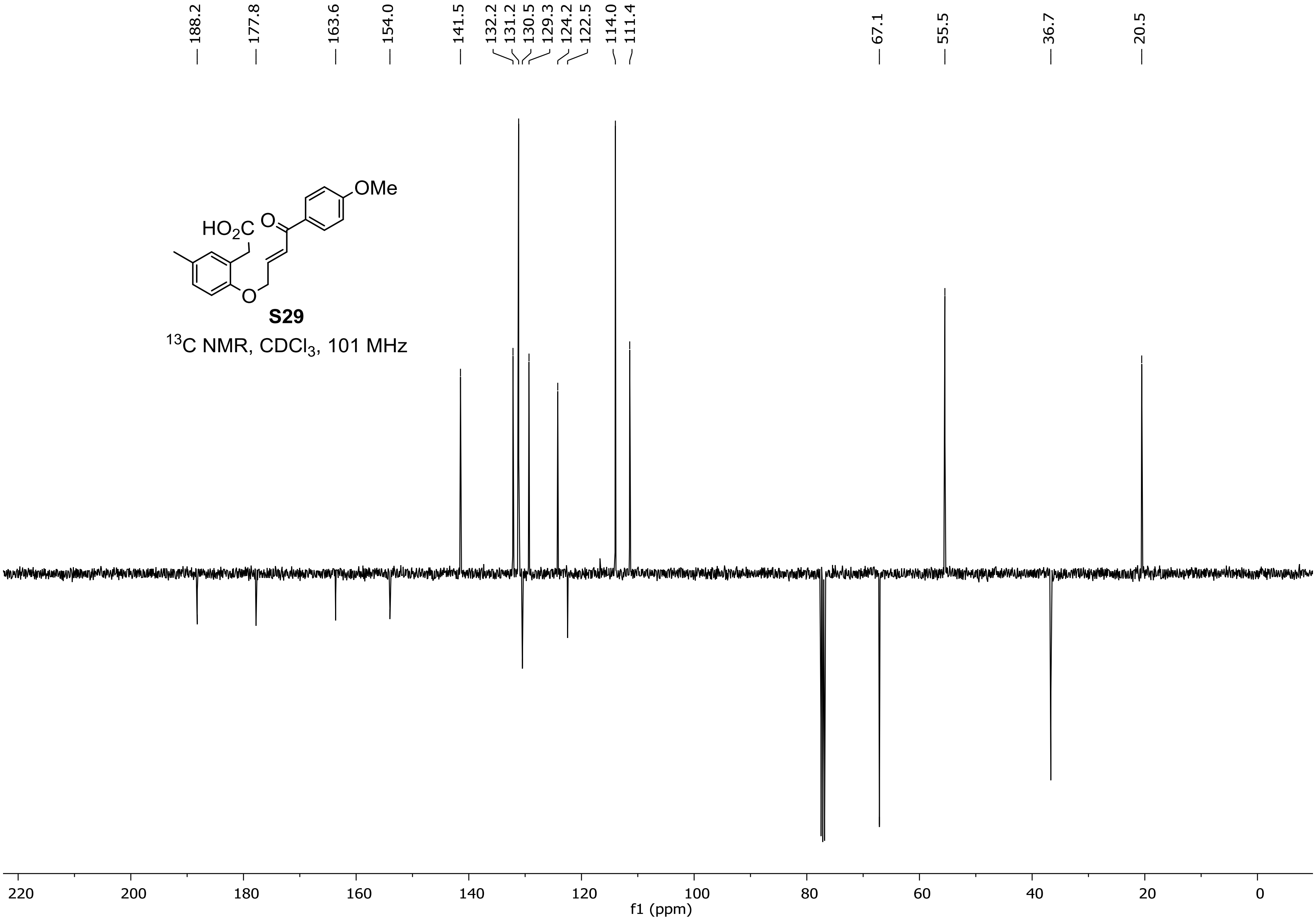

S29

^{13}C NMR, $CDCl_3$, 101 MHz

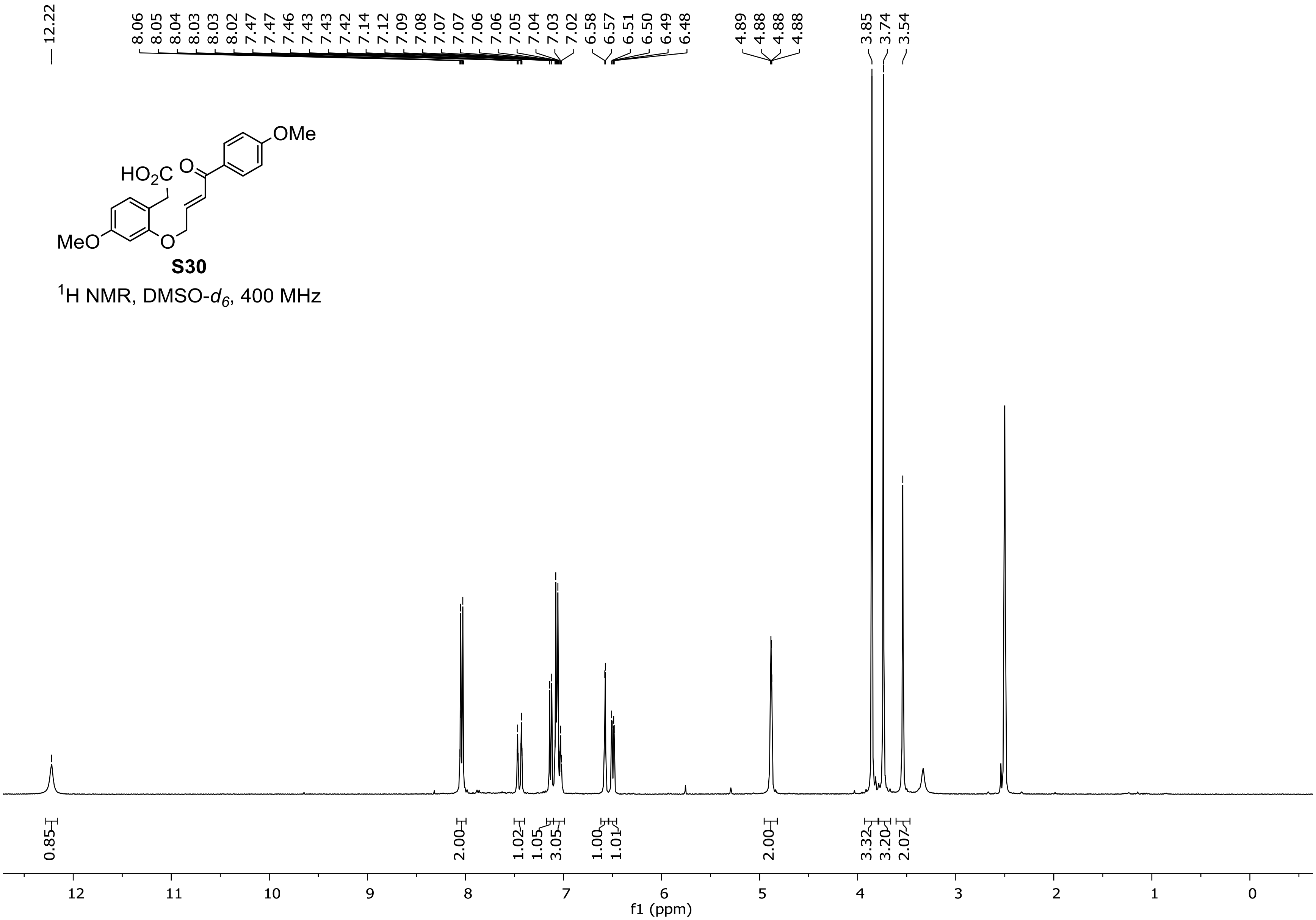

12.22
8.06 8.05 8.04 8.03 8.03 8.02 7.47 7.47 7.46 7.43 7.43 7.42 7.14 7.12 7.09 7.08 7.07 7.07 7.06 7.06 7.05 7.04 7.03 7.02 6.58 6.57 6.51 6.50 6.49 6.48
4.89 4.88 4.88 4.88
3.85 3.74 3.54
HO2C
O
OMe
MeO
O
S30
1H NMR, DMSO-d6, 400 MHz
0.85
2.00
1.02
1.05
3.05
1.00
1.01
2.00
3.32
3.20
2.07
12 11 10 9 8 7 6 5 4 3 2 1 0
f1 (ppm)

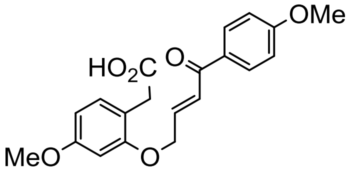

S30

^{13}C NMR, DMSO-d_6, 101 MHz

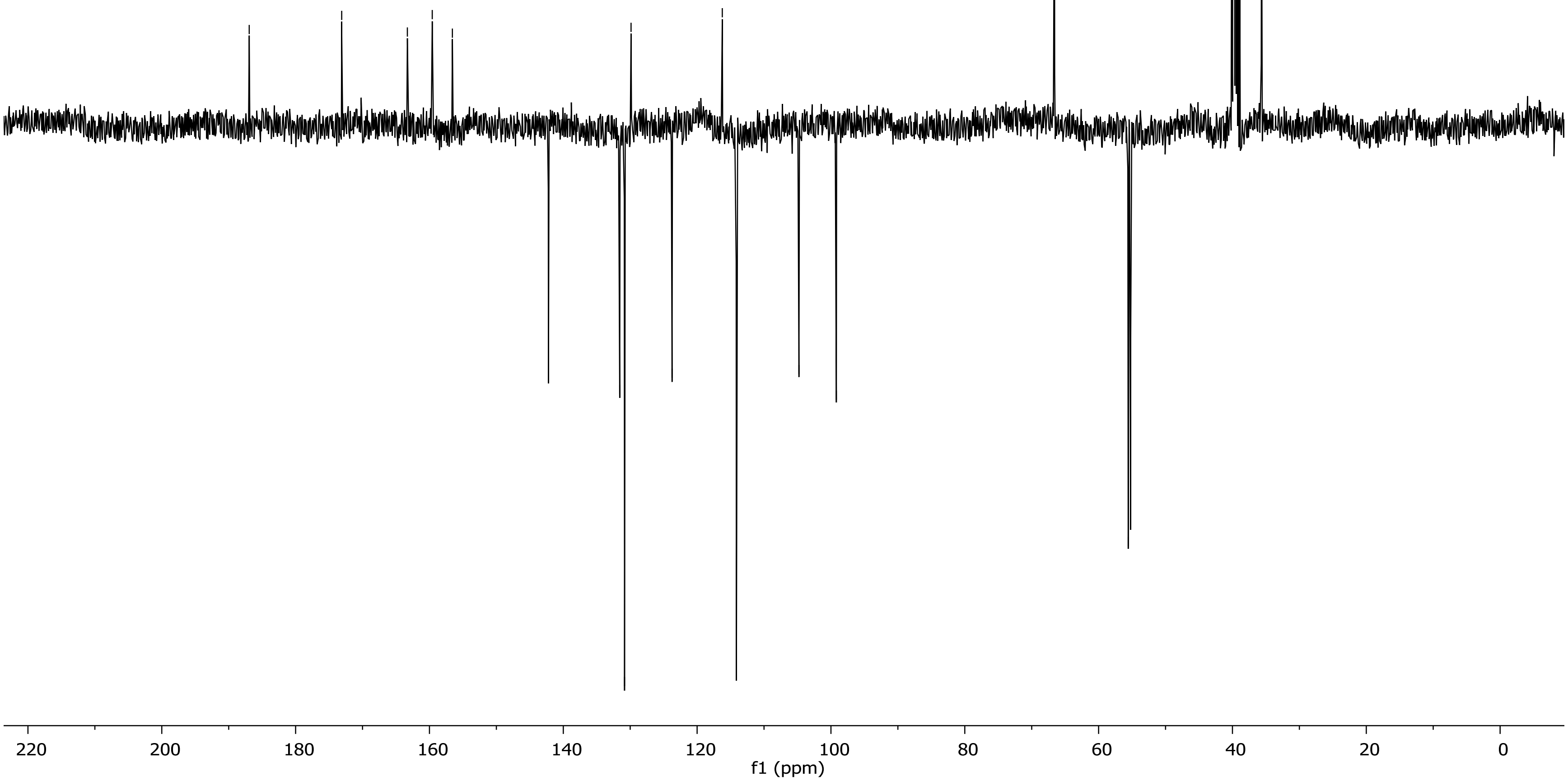

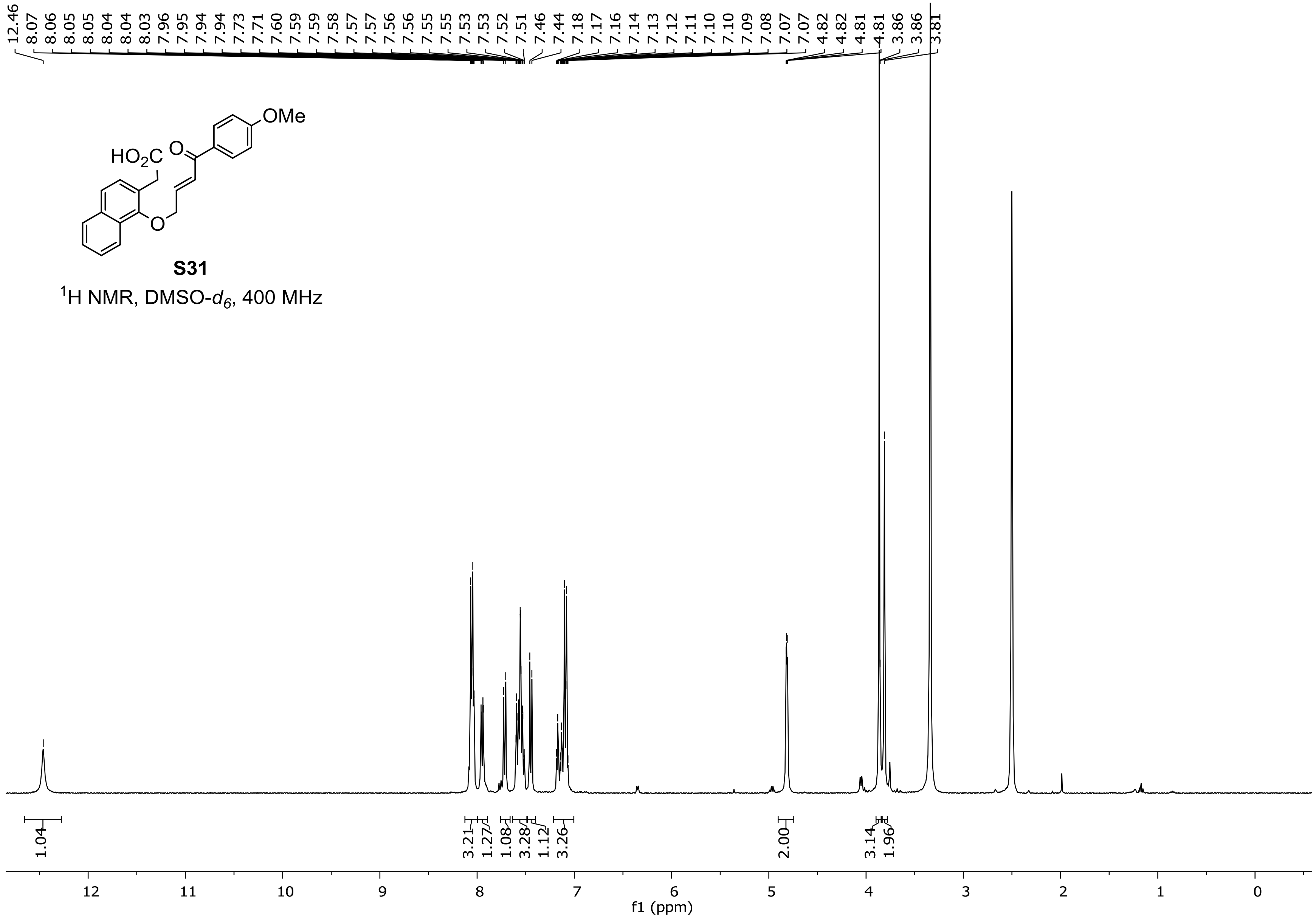
S31
^{1}H NMR, DMSO-d_6, 400 MHz
f1 (ppm)

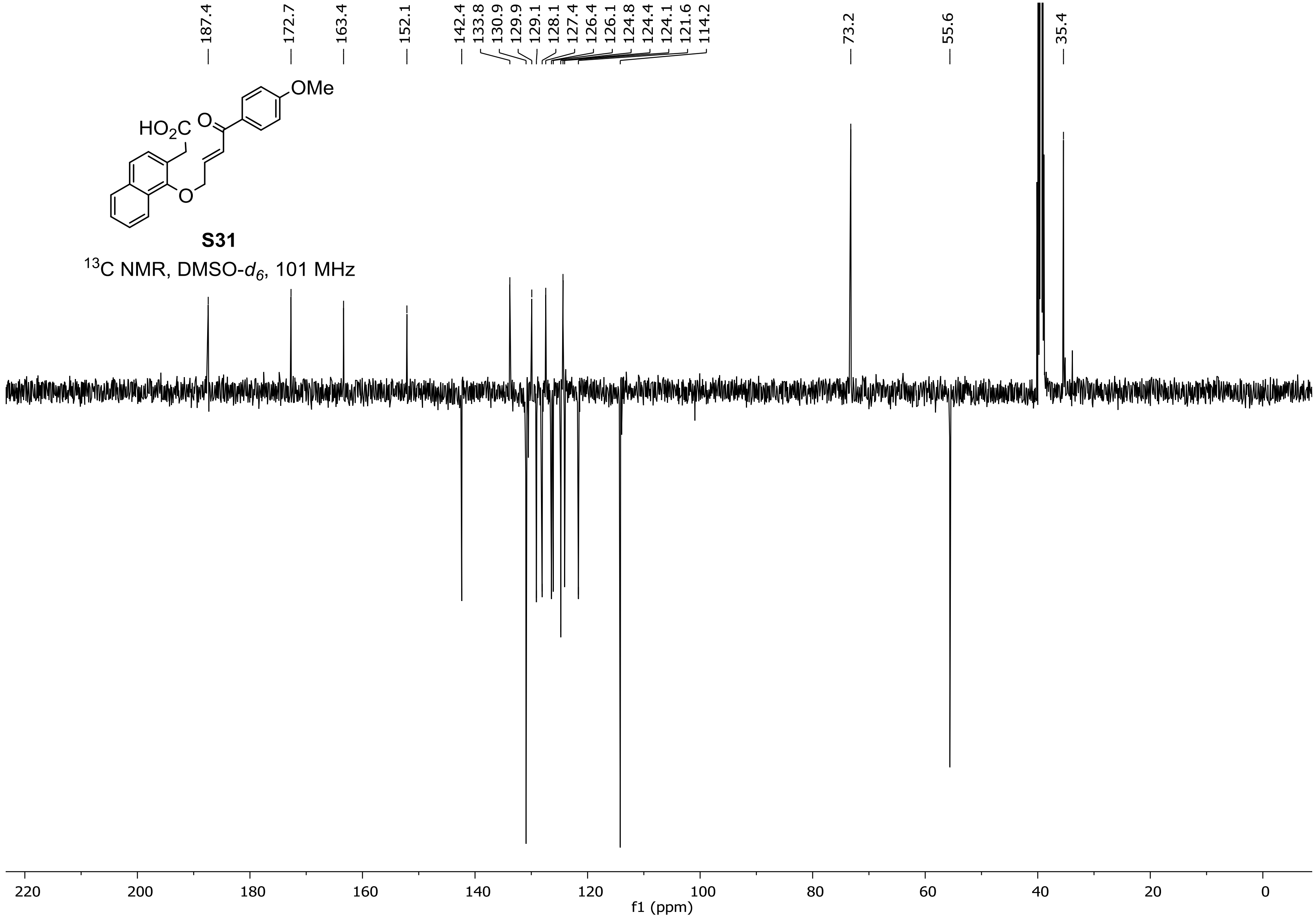

187.4
172.7
163.4
152.1
142.4
133.8
130.9
129.9
129.1
128.1
127.4
126.4
126.1
124.8
124.4
124.1
121.6
114.2
73.2
55.6
35.4
OMe
O
HO_2C
O
S31
^{13}C NMR, DMSO-d_6, 101 MHz
220 200 180 160 140 120 100 80 60 40 20 0
f1 (ppm)

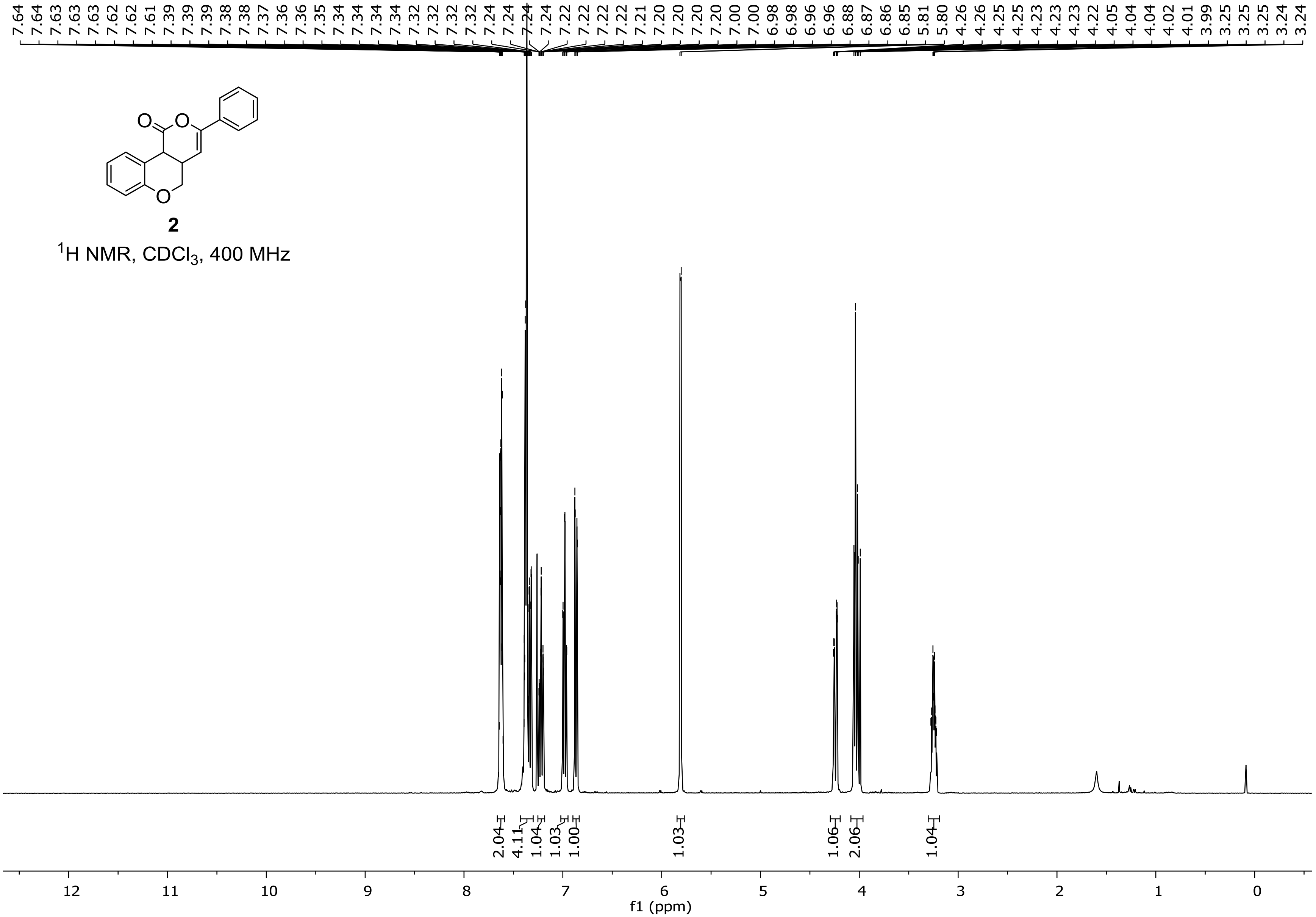

2

^{1}H NMR, $CDCl_3$, 400 MHz

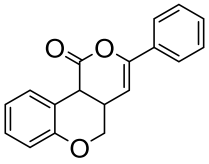

2

^{13}C NMR, $CDCl_3$, 101 MHz

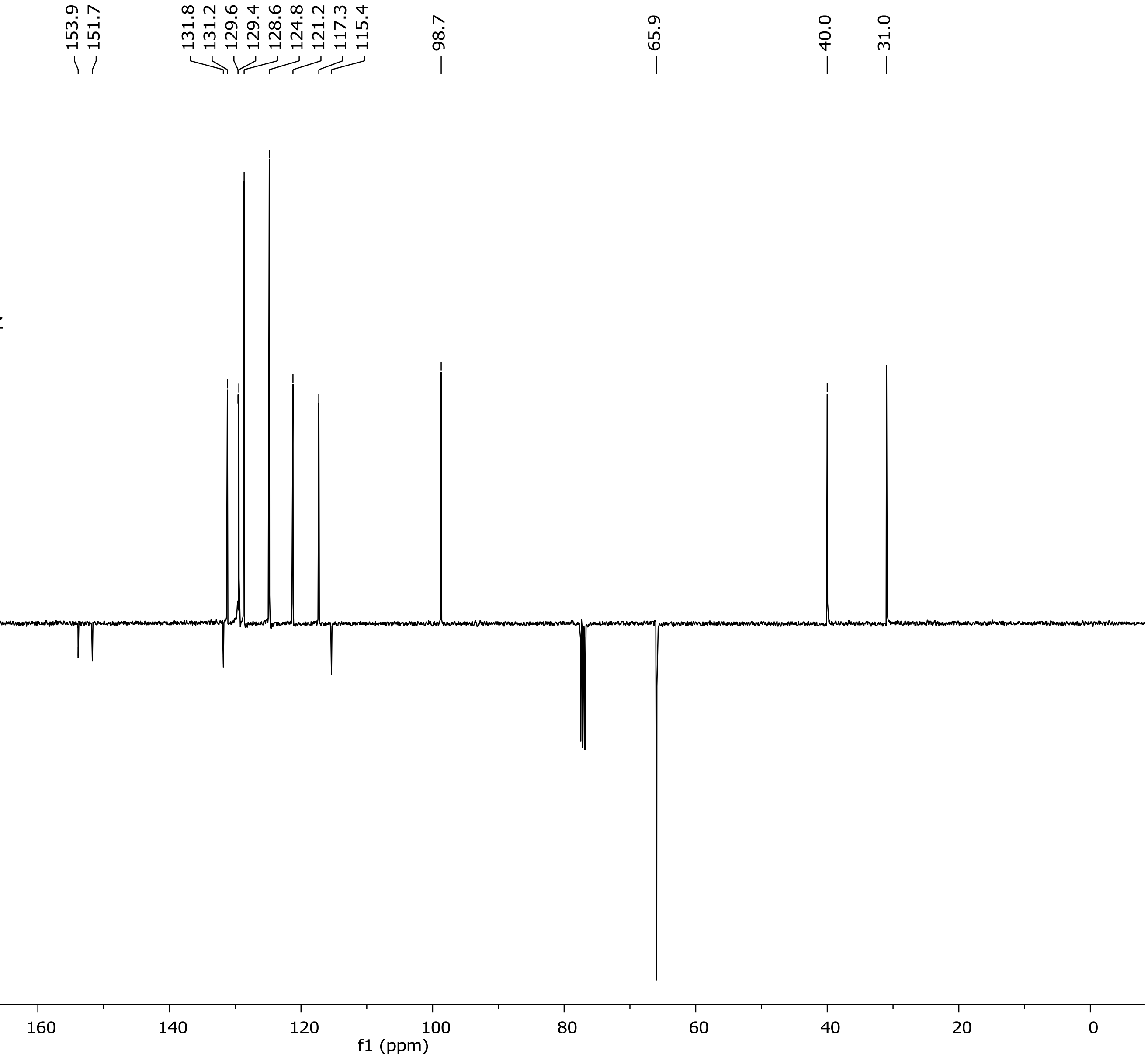

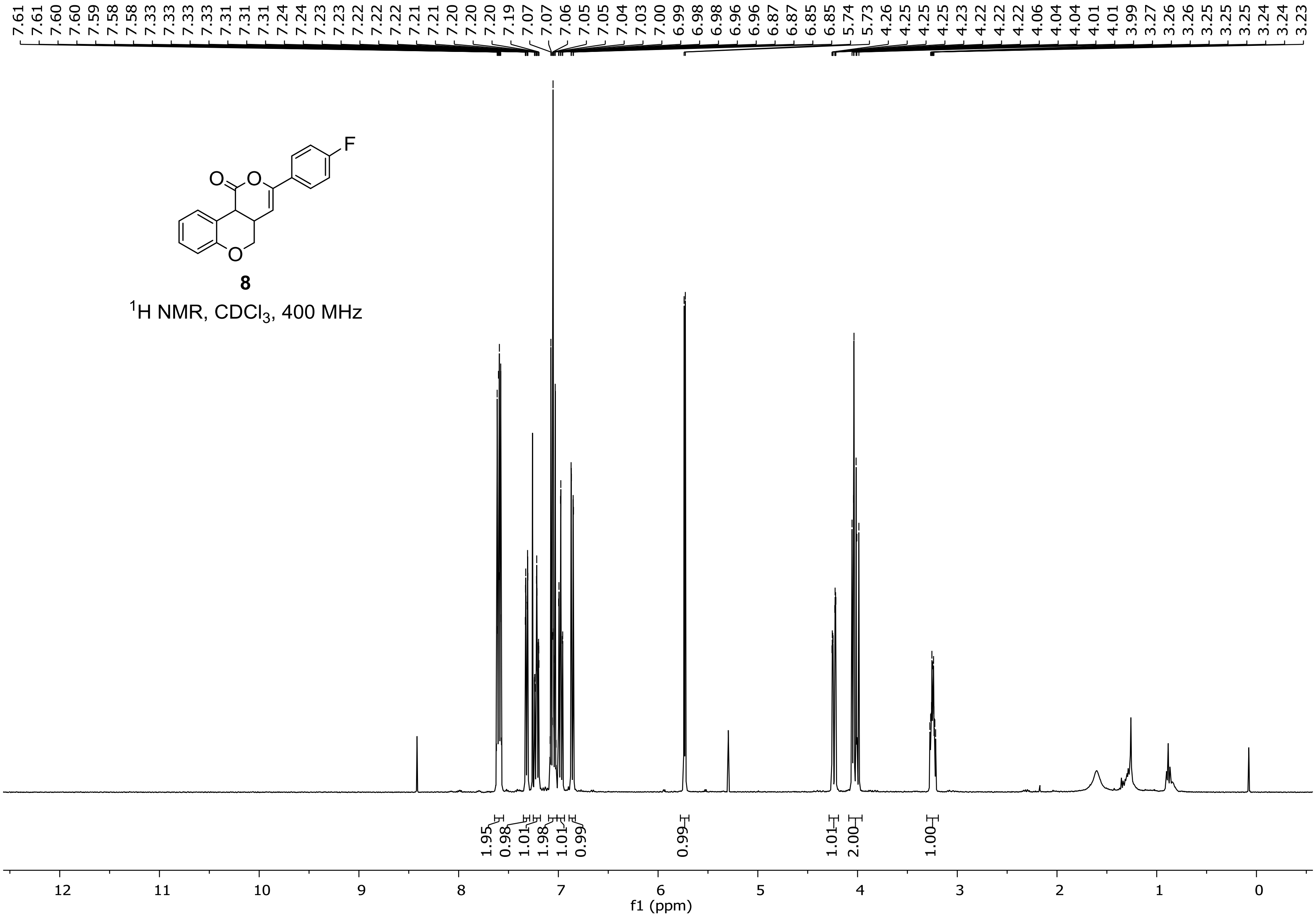

7.61 7.61 7.60 7.60 7.59 7.58 7.58 7.33 7.33 7.33 7.33 7.31 7.31 7.31 7.24 7.24 7.23 7.23 7.22 7.22 7.22 7.21 7.21 7.20 7.20 7.20 7.19 7.07 7.07 7.06 7.05 7.05 7.04 7.03 7.00 6.99 6.98 6.98 6.96 6.96 6.87 6.87 6.85 6.85 5.74 5.73 4.26 4.25 4.25 4.25 4.23 4.22 4.22 4.22 4.06 4.04 4.04 4.01 4.01 3.99 3.27 3.26 3.26 3.25 3.25 3.25 3.24 3.24 3.23
F
O
O
O
8
1H NMR, CDCl3, 400 MHz
1.95 0.98 1.01 1.98 1.01 0.99
0.99
1.01 2.00
1.00
12 11 10 9 8 7 6 5 4 3 2 1 0
f1 (ppm)

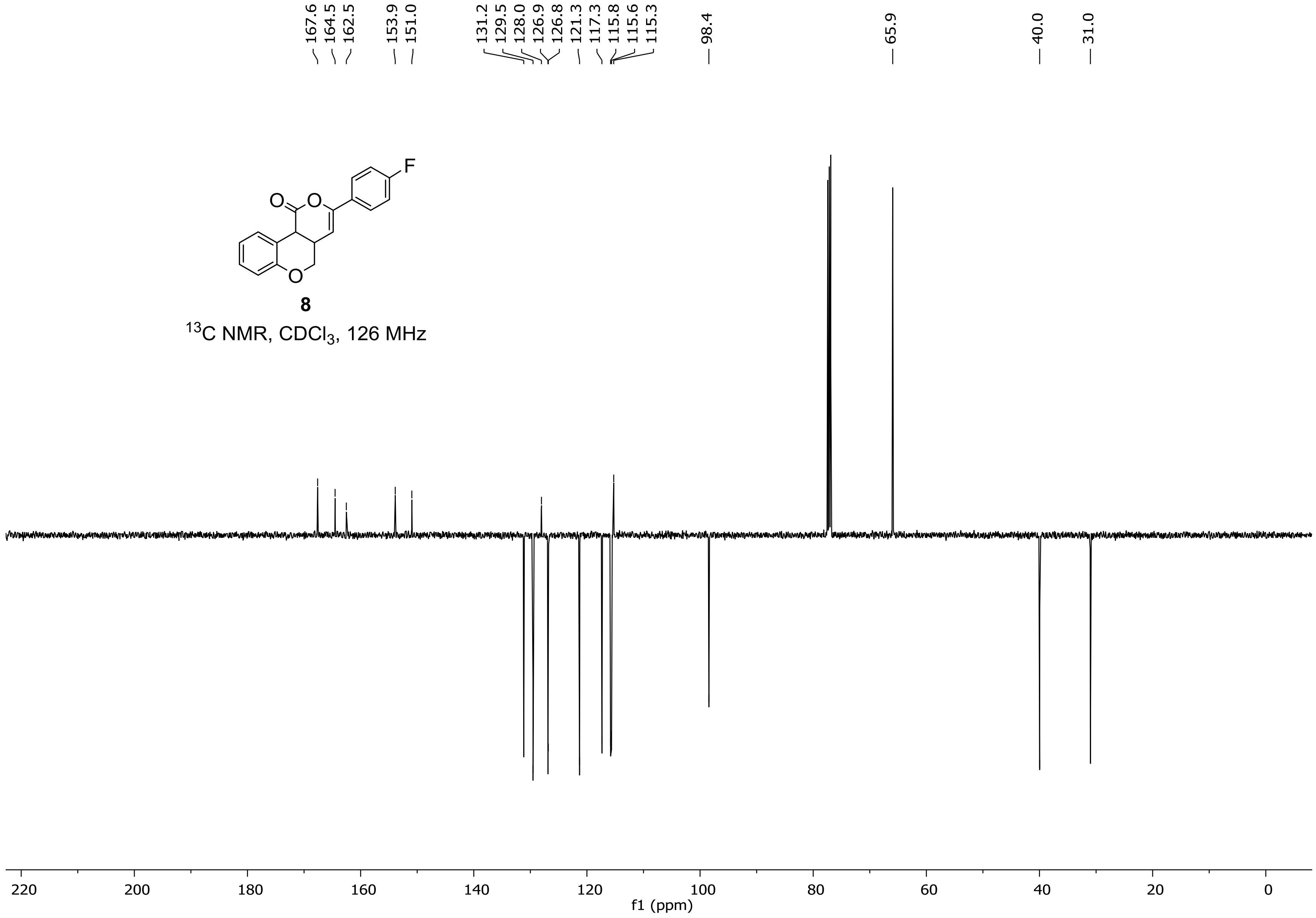
167.6
164.5
162.5
153.9
151.0
131.2
129.5
128.0
126.9
126.8
121.3
117.3
115.8
115.6
115.3
98.4
65.9
40.0
31.0
O
O
F
O
8
^{13}C NMR, $CDCl_3$, 126 MHz
220
200
180
160
140
120
100
80
60
40
20
0
f1 (ppm)

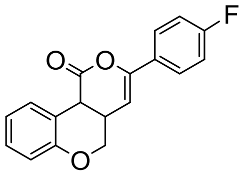

8

^{19}F NMR, $CDCl_3$, 480 MHz

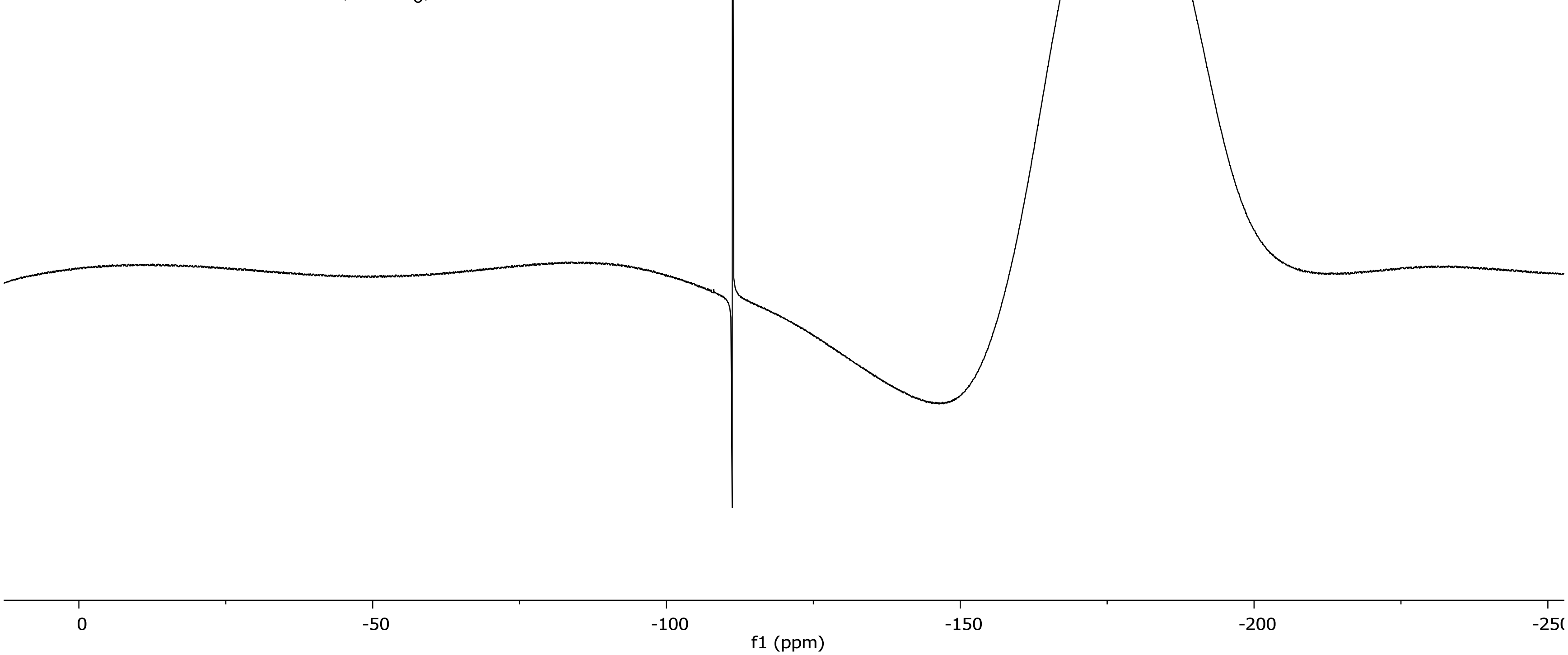

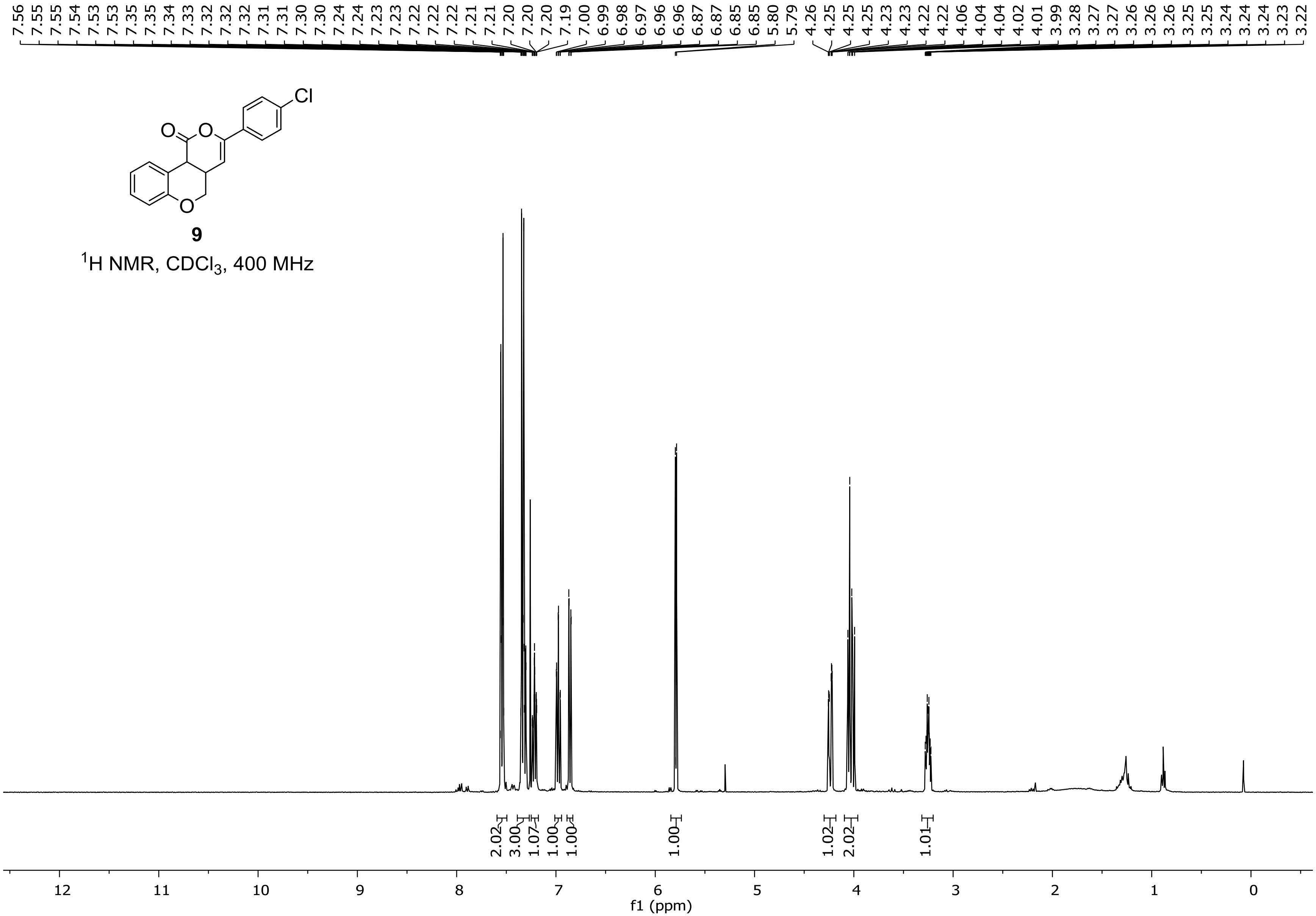

7.56 7.55 7.55 7.54 7.53 7.53 7.35 7.35 7.34 7.33 7.32 7.32 7.32 7.31 7.31 7.30 7.30 7.24 7.24 7.23 7.23 7.22 7.22 7.22 7.21 7.21 7.20 7.20 7.20 7.19 7.00 6.99 6.98 6.97 6.96 6.96 6.87 6.87 6.85 6.85 5.80 5.79 4.26 4.25 4.25 4.25 4.23 4.23 4.22 4.22 4.06 4.04 4.04 4.02 4.01 3.99 3.28 3.27 3.27 3.26 3.26 3.26 3.25 3.25 3.24 3.24 3.24 3.23 3.22
Cl
O
O
O
9
^{1}H NMR, CDCl$_3$, 400 MHz
2.02 3.00 1.07 1.00 1.00 1.00 1.02 2.02 1.01
12 11 10 9 8 7 6 5 4 3 2 1 0
f1 (ppm)

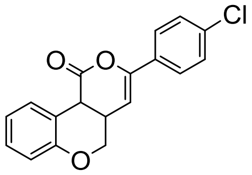

9

^{13}C NMR, $CDCl_3$, 126 MHz

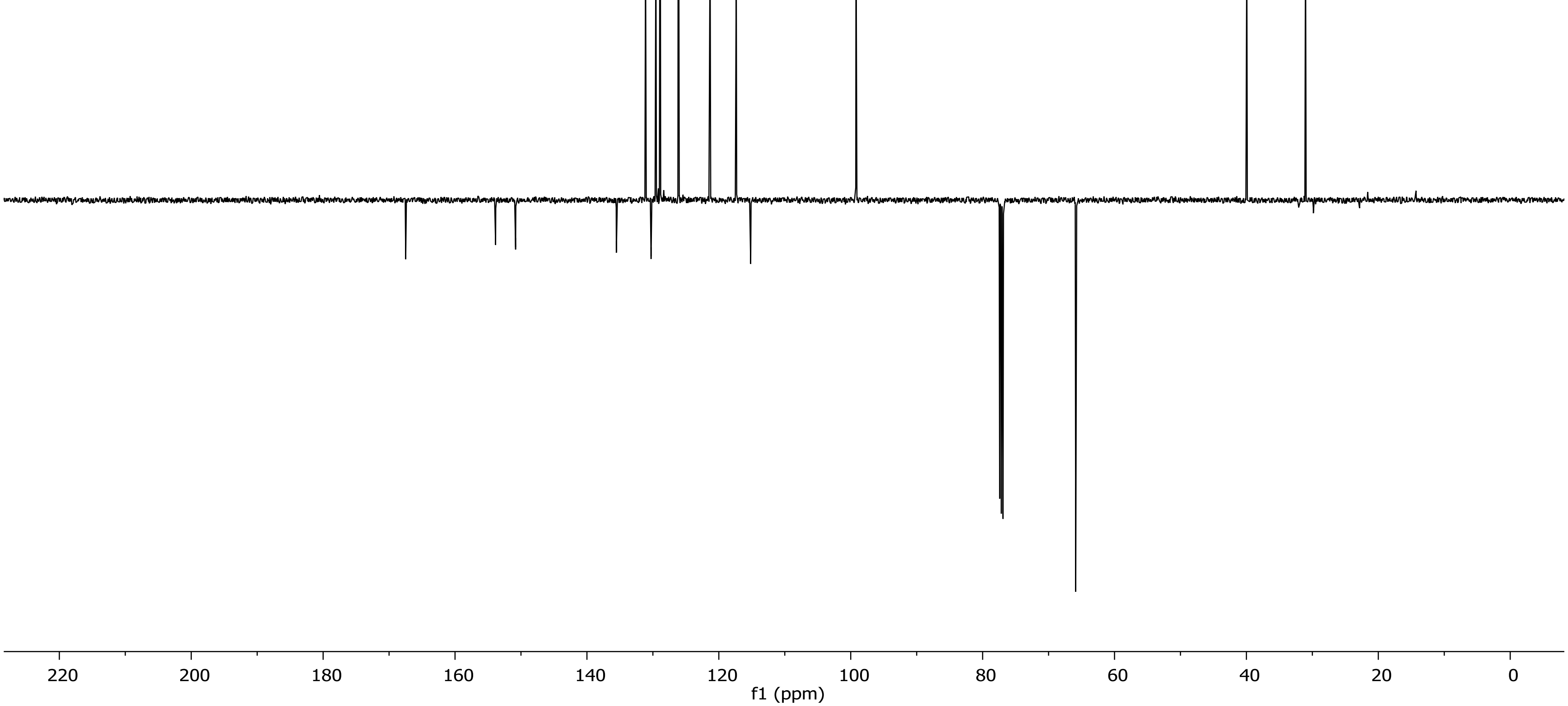

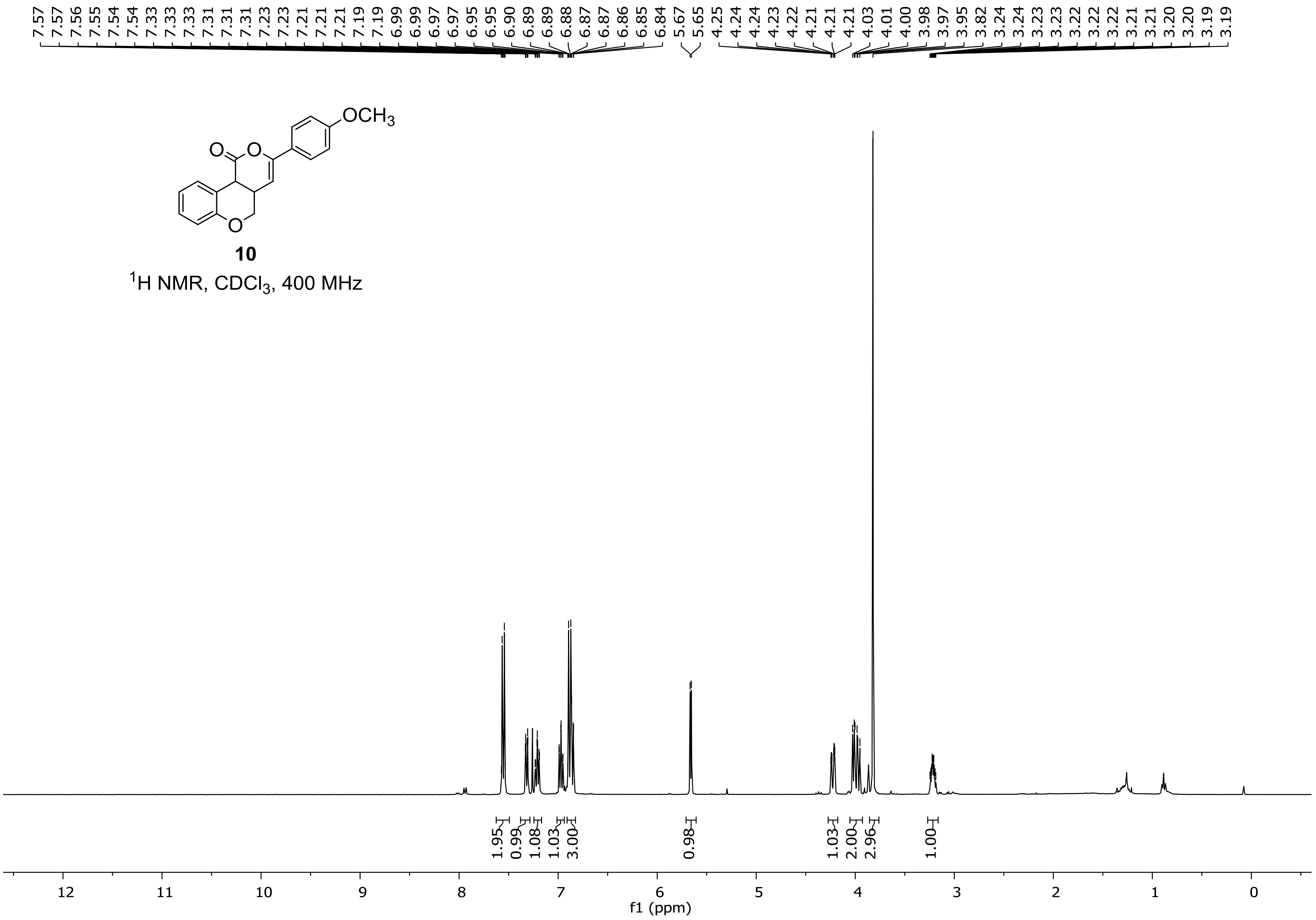
7.57 7.57 7.56 7.55 7.54 7.54 7.33 7.33 7.33 7.31 7.31 7.31 7.23 7.23 7.21 7.21 7.21 7.19 7.19 6.99 6.99 6.97 6.97 6.95 6.95 6.90 6.89 6.89 6.88 6.87 6.87 6.86 6.85 6.84 5.67 5.65 4.25 4.24 4.24 4.23 4.22 4.21 4.21 4.21 4.03 4.01 4.00 3.98 3.97 3.95 3.82 3.24 3.24 3.23 3.23 3.22 3.22 3.22 3.21 3.21 3.20 3.20 3.19 3.19
OCH3
O
O
O
10
1H NMR, CDCl3, 400 MHz
1.95 0.99 1.08 1.03 3.00 0.98 1.03 2.00 2.96 1.00
12 11 10 9 8 7 6 5 4 3 2 1 0
f1 (ppm)

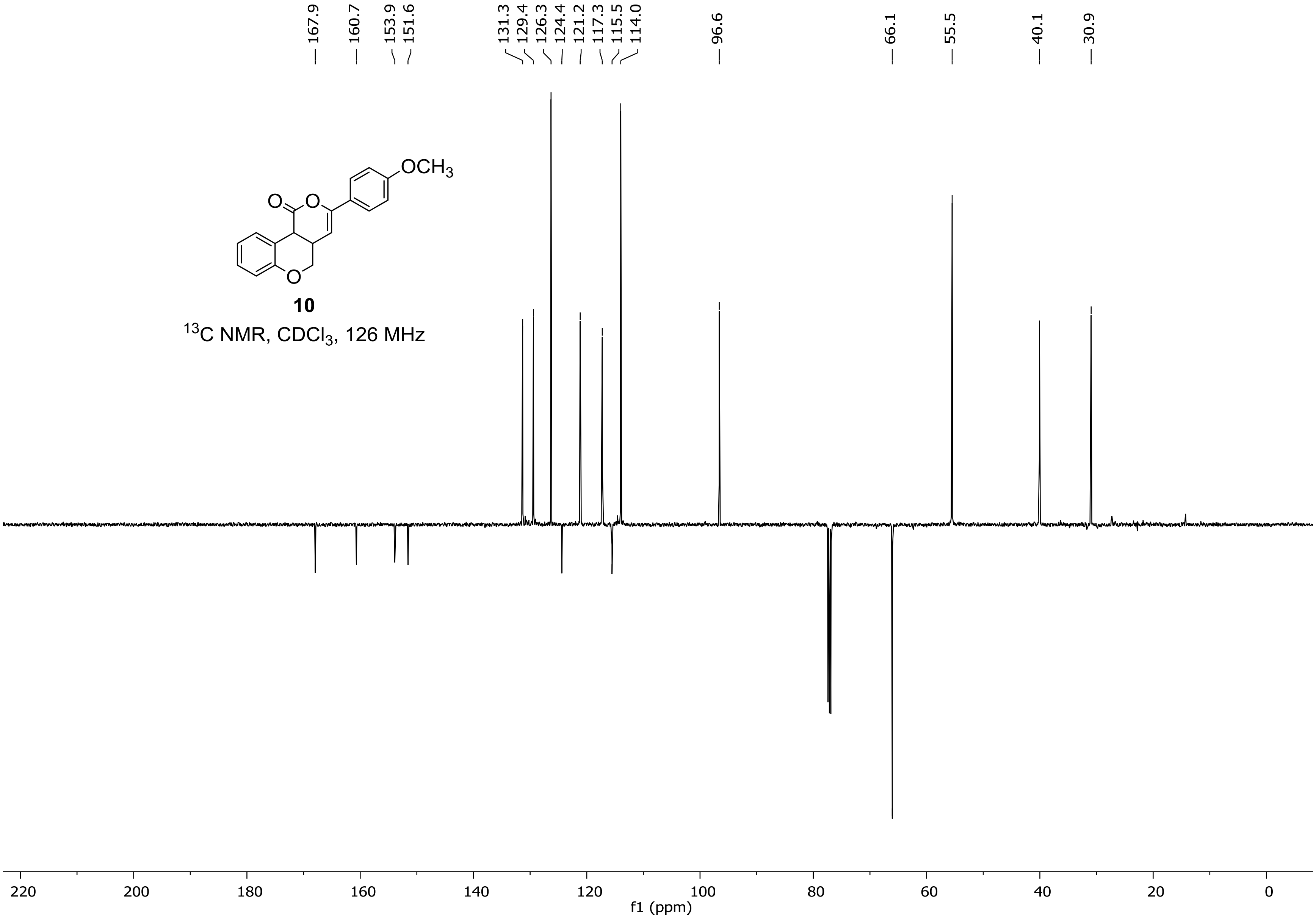

OCH3
O
O
O
10
^{13}C NMR, $CDCl_3$, 126 MHz
167.9
160.7
153.9
151.6
131.3
129.4
126.3
124.4
121.2
117.3
115.5
114.0
96.6
66.1
55.5
40.1
30.9
220
200
180
160
140
120
100
80
60
40
20
0
f1 (ppm)

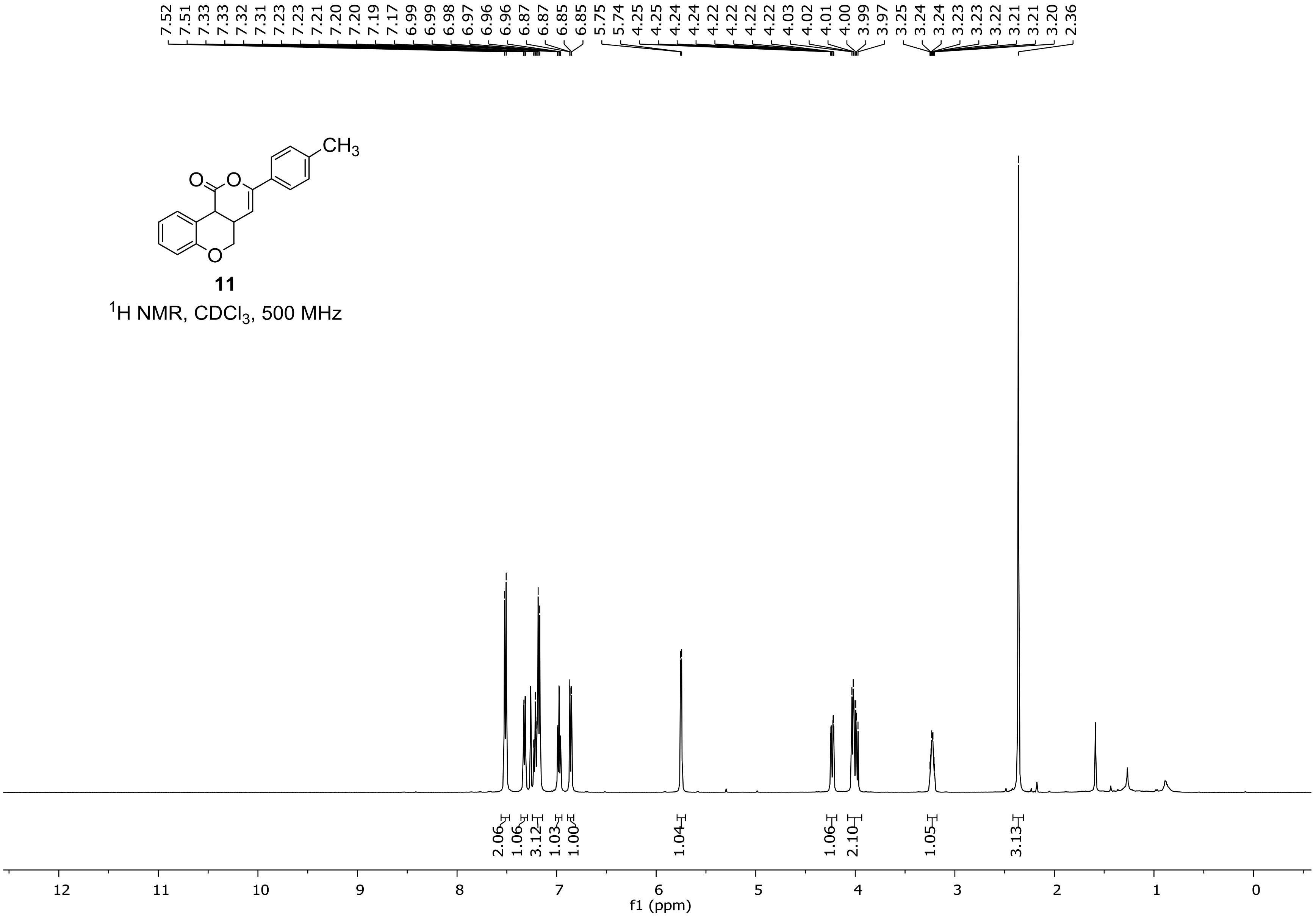

11

^{1}H NMR, $CDCl_3$, 500 MHz

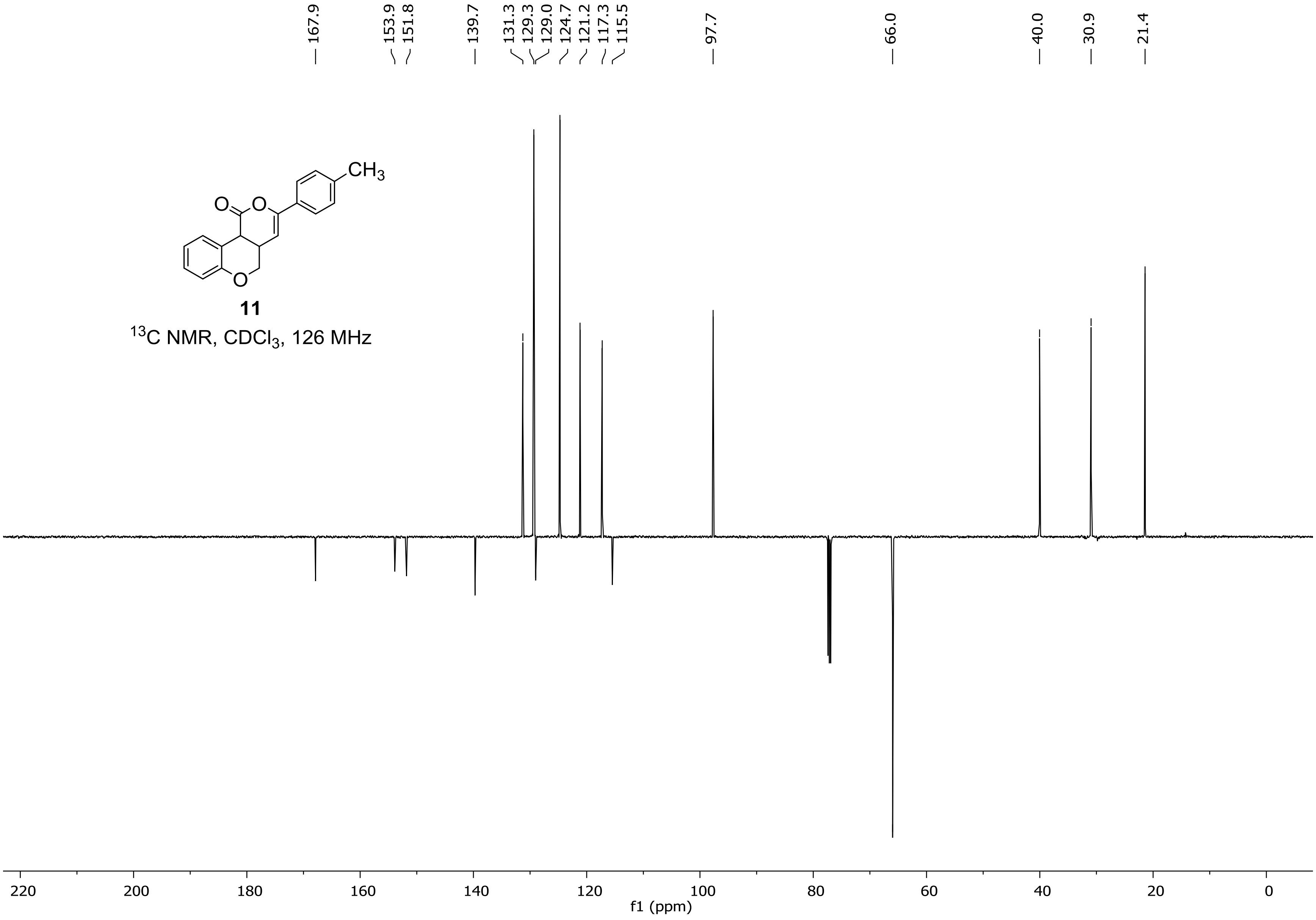

167.9
153.9
151.8
139.7
131.3
129.3
129.0
124.7
121.2
117.3
115.5
97.7
66.0
40.0
30.9
21.4
CH3
O
O
O
11
13C NMR, CDCl3, 126 MHz
220
200
180
160
140
120
100
80
60
40
20
0
f1 (ppm)

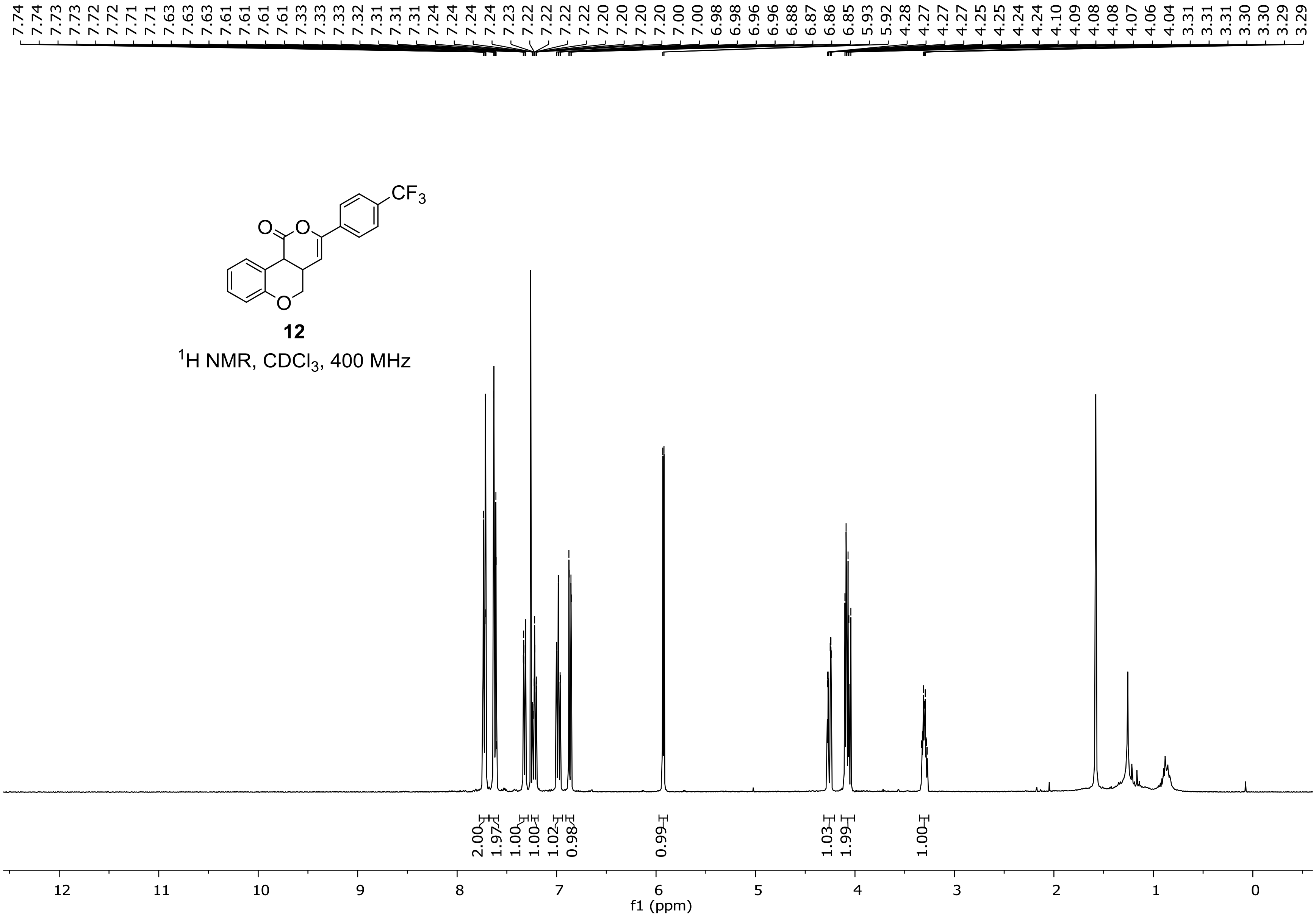

12

^1H NMR, $CDCl_3$, 400 MHz

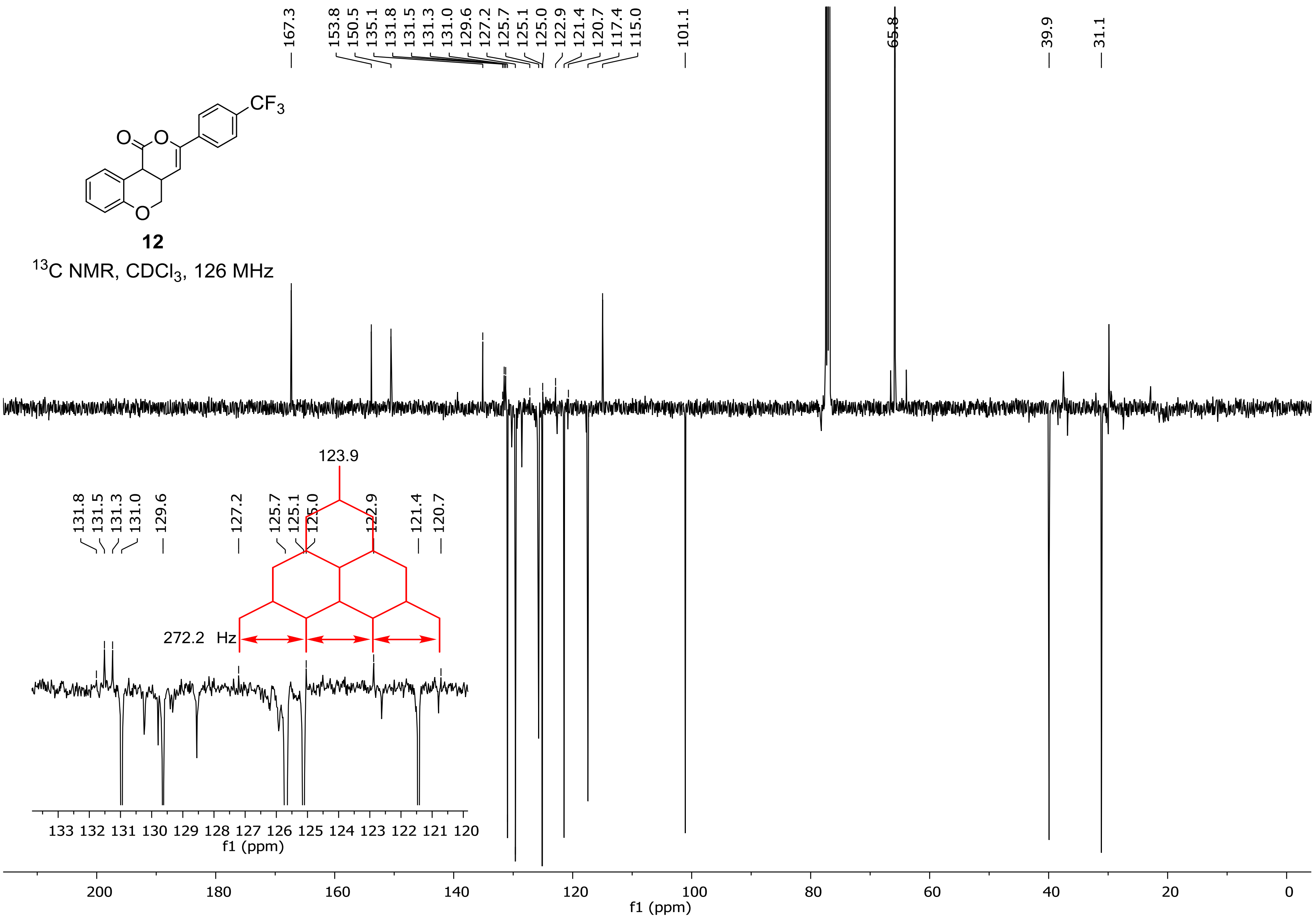
167.3
153.8
150.5
135.1
131.8
131.5
131.3
131.0
129.6
127.2
125.7
125.1
125.0
122.9
121.4
120.7
117.4
115.0
101.1
65.8
39.9
31.1
CF3
O
O
O
O
12
13C NMR, CDCl3, 126 MHz
123.9
272.2 Hz
133 132 131 130 129 128 127 126 125 124 123 122 121 120
f1 (ppm)
200 180 160 140 120 100 80 60 40 20 0
f1 (ppm)

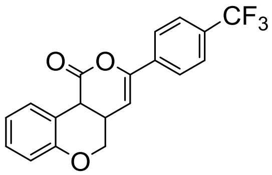

12

^{19}F NMR, $CDCl_3$, 376 MHz

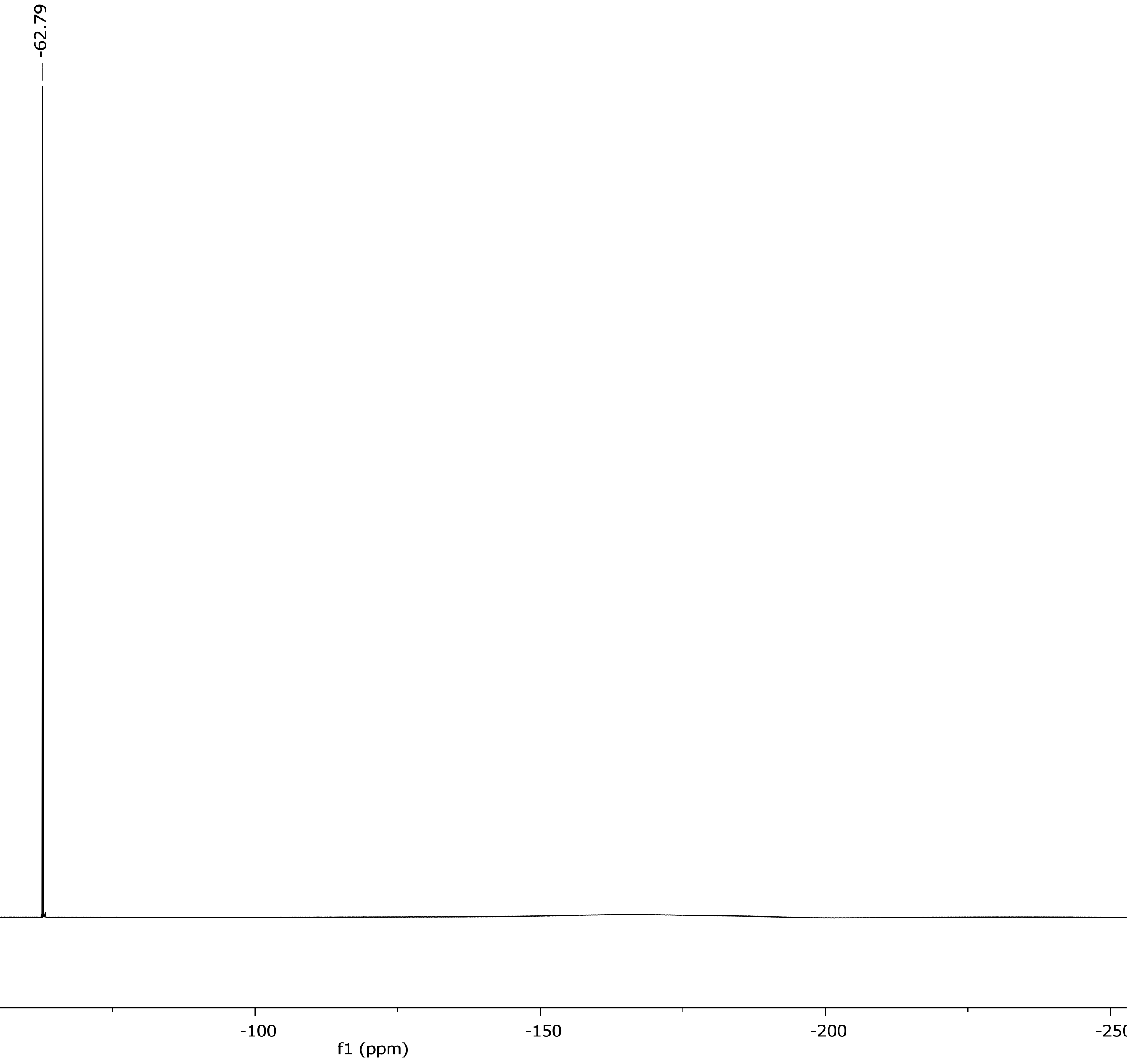

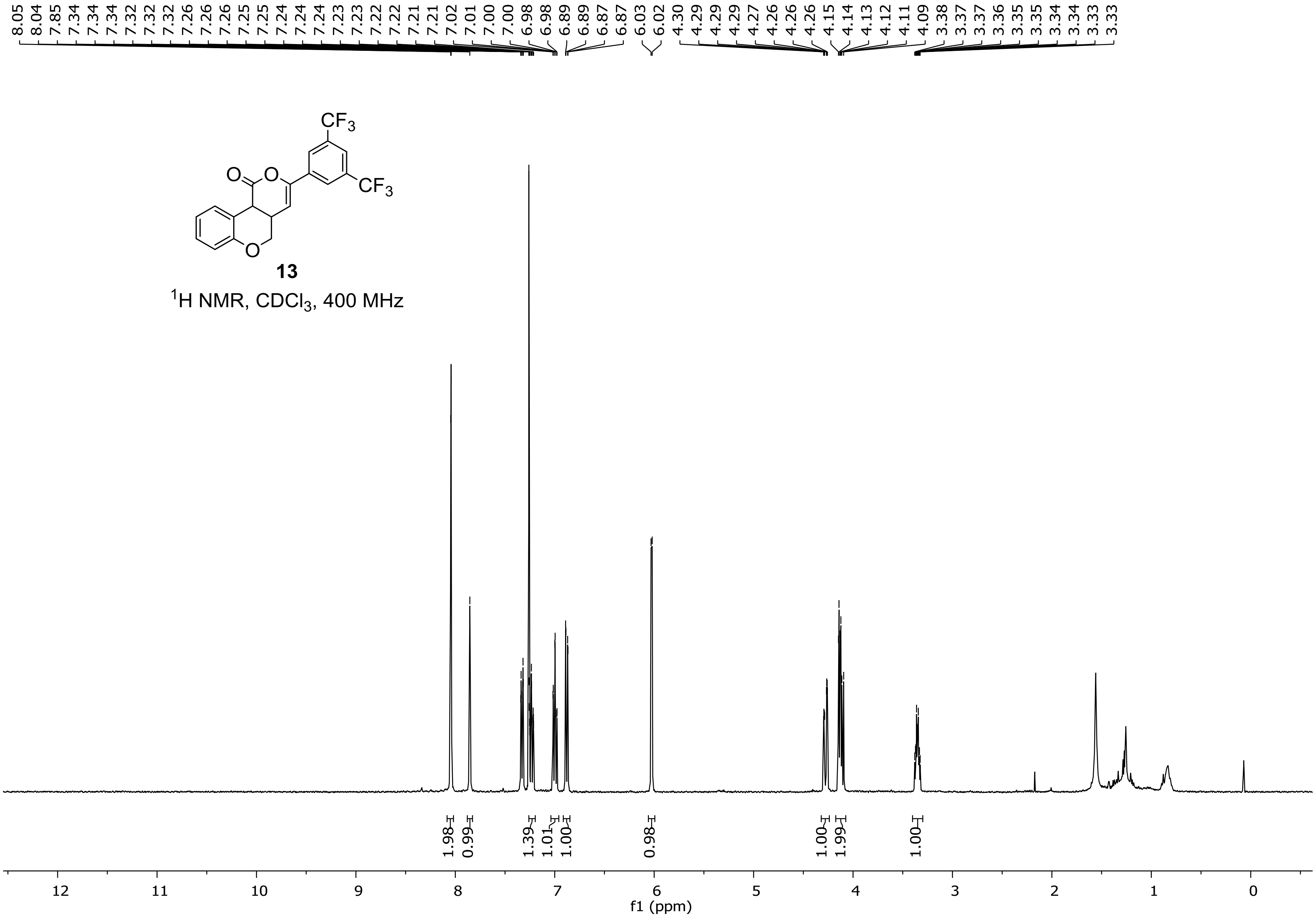
8.05 8.04 7.85 7.34 7.34 7.34 7.32 7.32 7.32 7.26 7.26 7.26 7.25 7.25 7.24 7.24 7.24 7.23 7.23 7.22 7.22 7.21 7.21 7.02 7.01 7.00 7.00 6.98 6.98 6.89 6.89 6.87 6.87 6.03 6.02 4.30 4.29 4.29 4.29 4.27 4.26 4.26 4.26 4.15 4.14 4.13 4.12 4.11 4.09 3.38 3.37 3.37 3.36 3.35 3.35 3.34 3.34 3.33 3.33
CF_3
O
O
CF_3
O
13
^1H NMR, $CDCl_3$, 400 MHz
1.98
0.99
1.39
1.01
1.00
0.98
1.00
1.99
1.00
12 11 10 9 8 7 6 5 4 3 2 1 0
f1 (ppm)

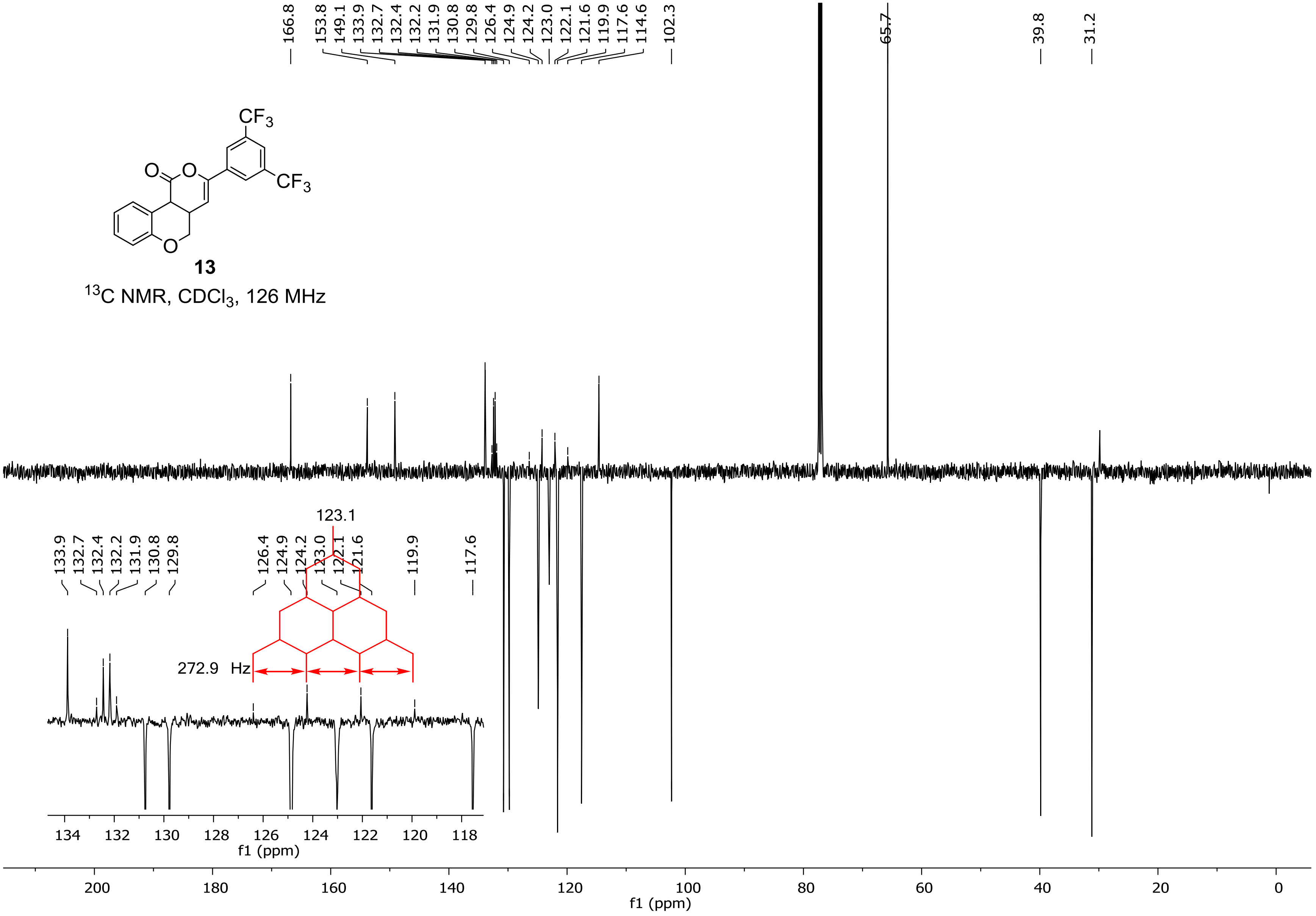

166.8
153.8
149.1
133.9
132.7
132.4
132.2
131.9
130.8
129.8
126.4
124.9
124.2
123.0
122.1
121.6
119.9
117.6
114.6
102.3
65.7
39.8
31.2
CF3
CF3
O
O
O
13
13C NMR, CDCl3, 126 MHz
123.1
272.9 Hz
f1 (ppm)
f1 (ppm)

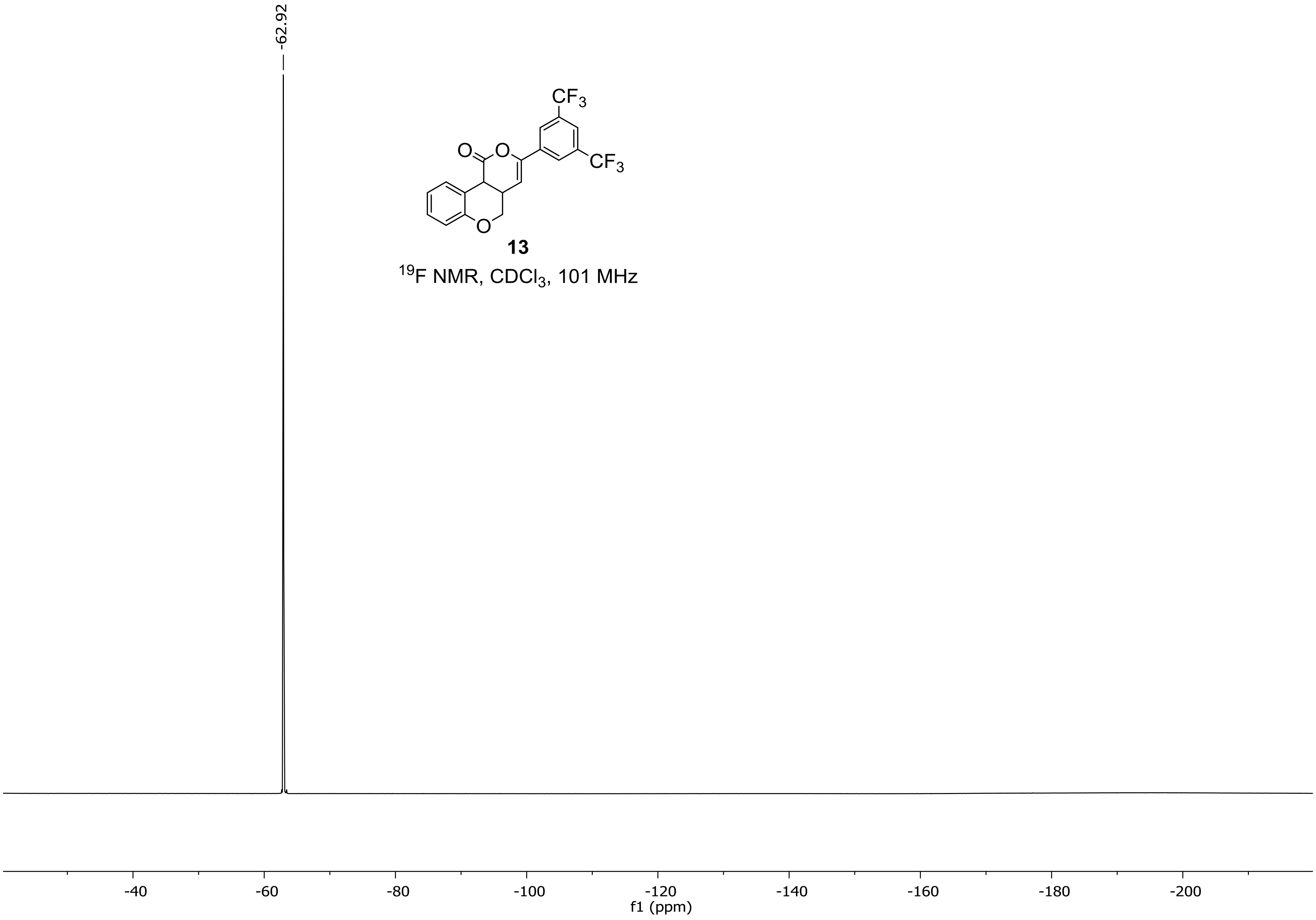

13

^{19}F NMR, $CDCl_3$, 101 MHz

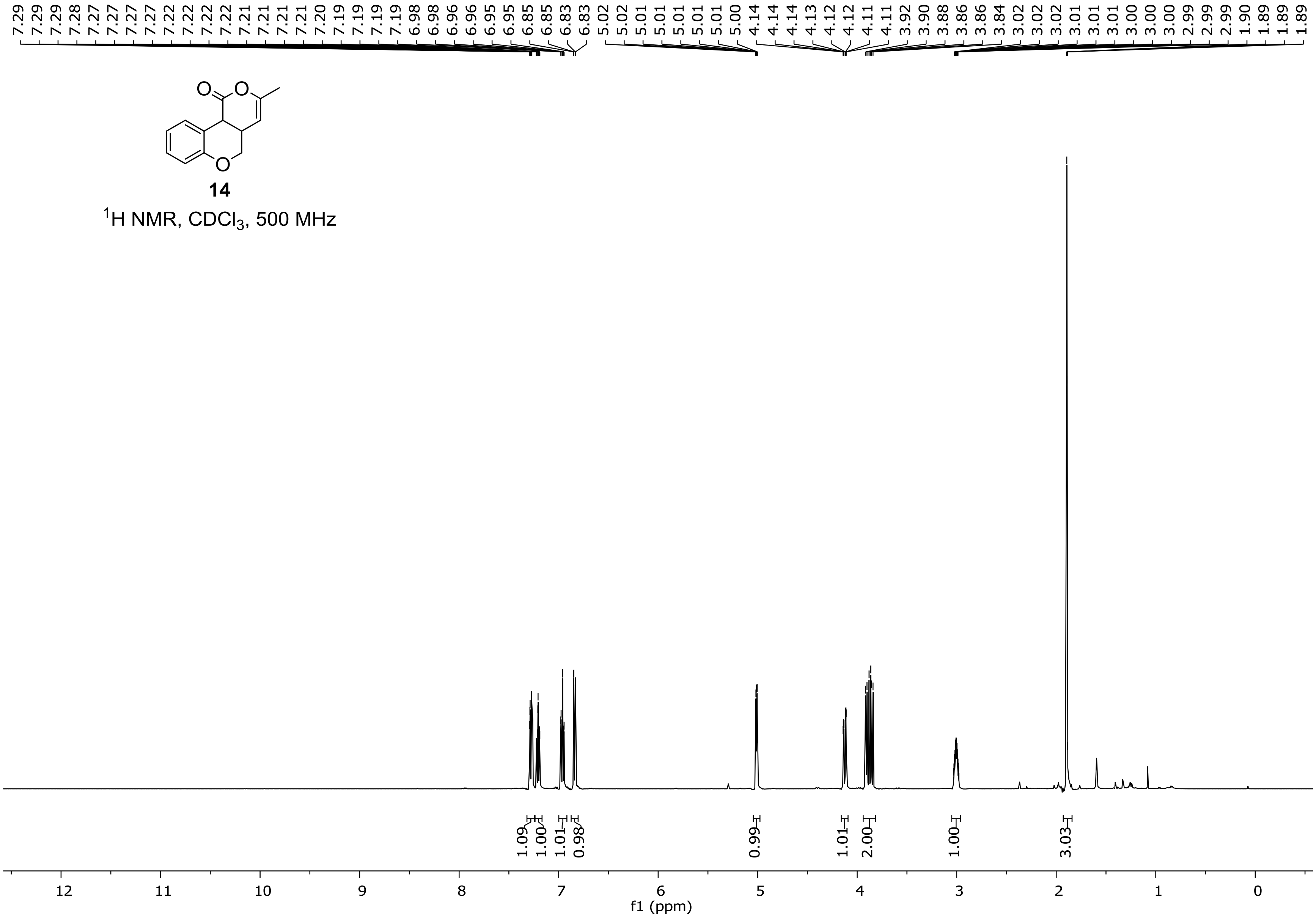
7.29 7.29 7.29 7.28 7.27 7.27 7.27 7.27 7.22 7.22 7.22 7.22 7.21 7.21 7.21 7.21 7.20 7.19 7.19 7.19 7.19 6.98 6.98 6.96 6.96 6.95 6.95 6.85 6.85 6.83 6.83 5.02 5.02 5.01 5.01 5.01 5.01 5.01 5.00 4.14 4.14 4.14 4.13 4.12 4.12 4.11 4.11 3.92 3.90 3.88 3.86 3.86 3.84 3.02 3.02 3.02 3.01 3.01 3.01 3.00 3.00 3.00 2.99 2.99 2.99 1.90 1.89 1.89 1.89
14
1H NMR, CDCl3, 500 MHz
1.09 1.00 1.01 0.98 0.99 1.01 2.00 1.00 3.03
12 11 10 9 8 7 6 5 4 3 2 1 0
f1 (ppm)

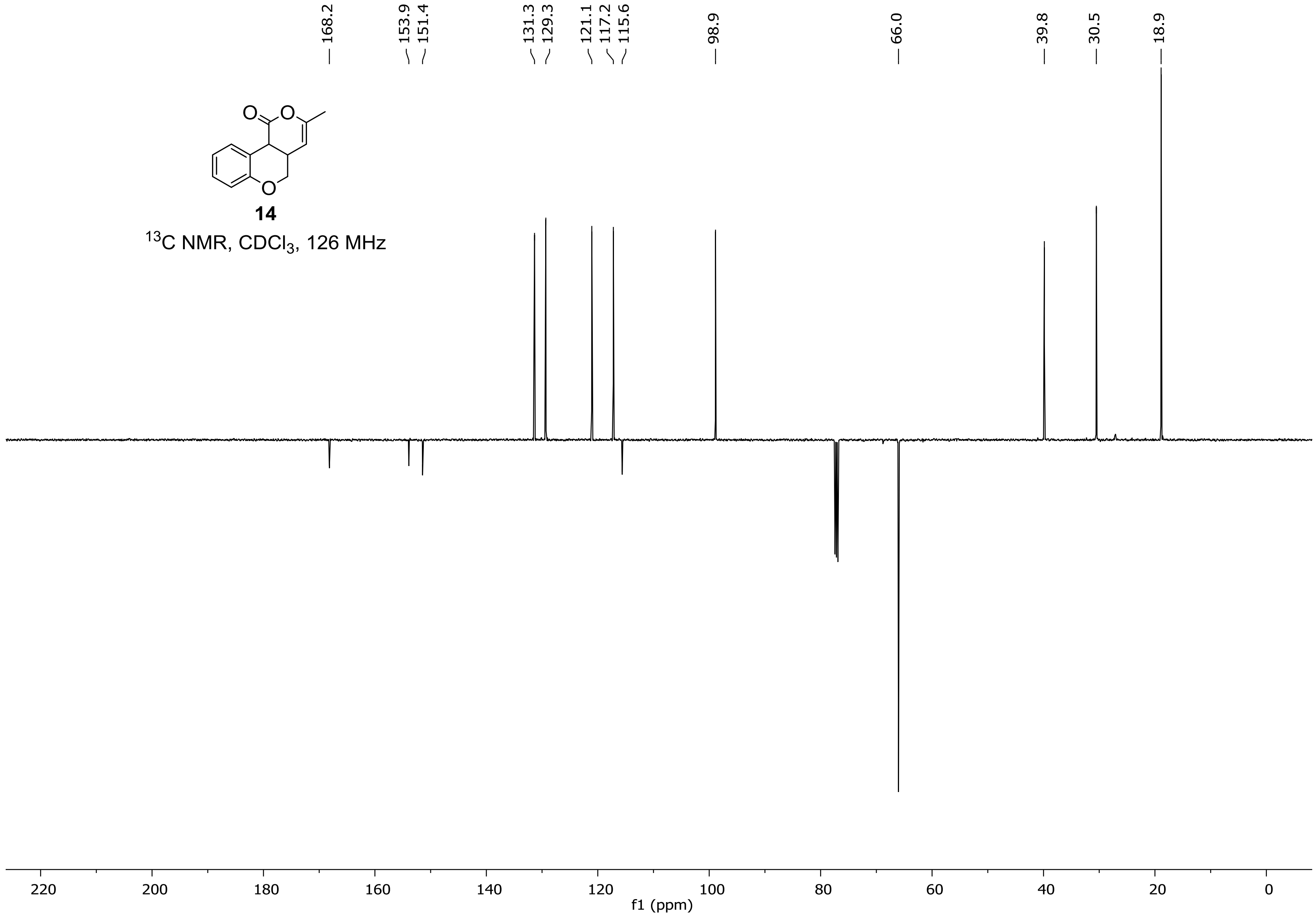
168.2
153.9
151.4
131.3
129.3
121.1
117.2
115.6
98.9
66.0
39.8
30.5
18.9
O
O
O
14
13C NMR, CDCl3, 126 MHz
220
200
180
160
140
120
100
80
60
40
20
0
f1 (ppm)

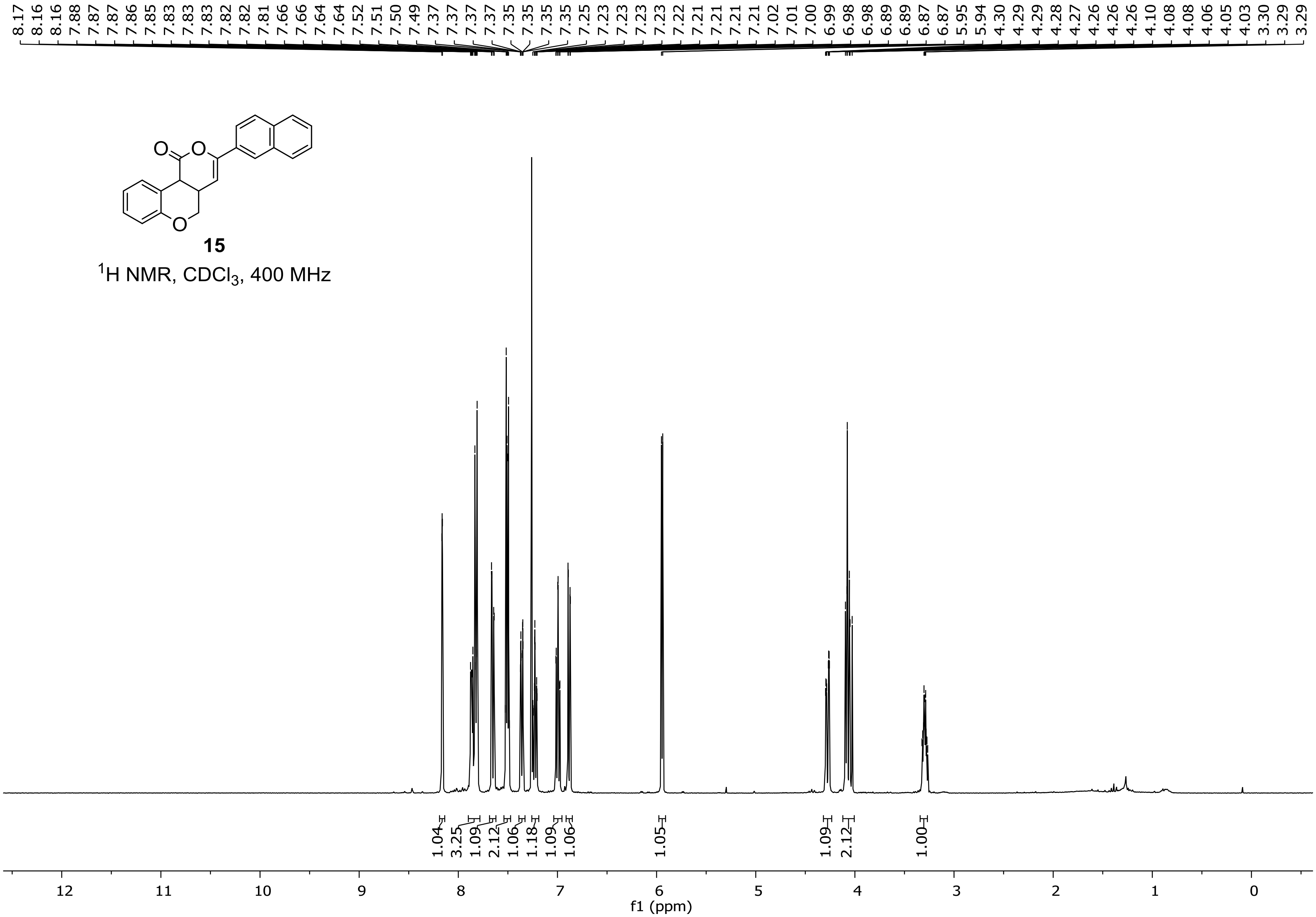
8.17 8.16 8.16 7.88 7.87 7.87 7.86 7.85 7.83 7.83 7.83 7.82 7.82 7.81 7.66 7.66 7.64 7.64 7.52 7.51 7.50 7.49 7.37 7.37 7.37 7.37 7.35 7.35 7.35 7.35 7.25 7.23 7.23 7.23 7.23 7.22 7.21 7.21 7.21 7.21 7.02 7.01 7.00 6.99 6.98 6.98 6.89 6.89 6.87 6.87 5.95 5.94 4.30 4.29 4.29 4.28 4.27 4.26 4.26 4.26 4.10 4.08 4.08 4.06 4.05 4.03 3.30 3.29 3.29
O
O
O
15
^{1}H NMR, $CDCl_3$, 400 MHz
1.04 3.25 1.09 2.12 1.06 1.18 1.09 1.06 1.05 1.09 2.12 1.00
12 11 10 9 8 7 6 5 4 3 2 1 0
f1 (ppm)

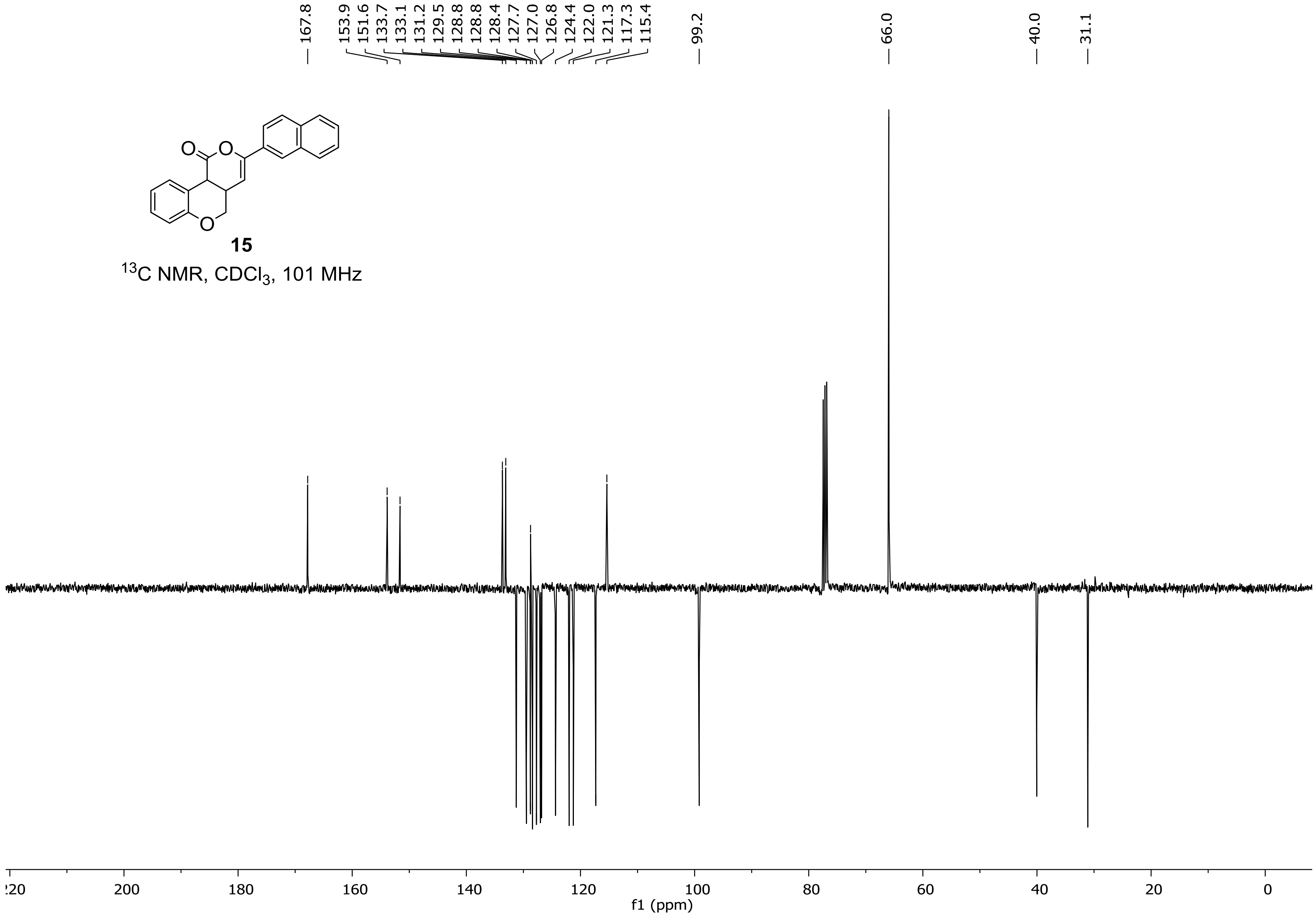
167.8
153.9
151.6
133.7
133.1
131.2
129.5
128.8
128.8
128.4
127.7
127.0
126.8
124.4
122.0
121.3
117.3
115.4
99.2
66.0
40.0
31.1
15
^{13}C NMR, $CDCl_3$, 101 MHz
f1 (ppm)

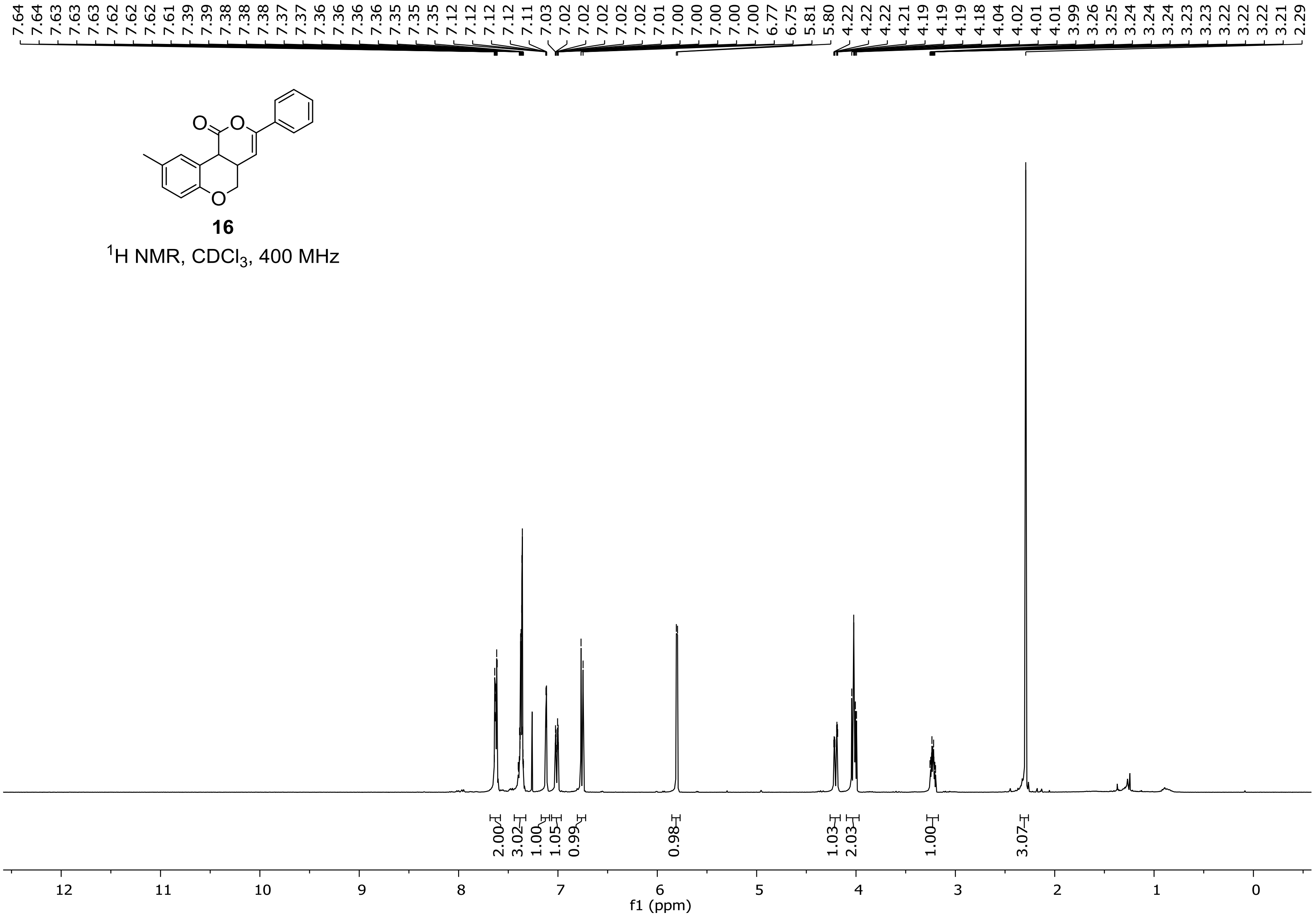
7.64
7.64
7.63
7.63
7.63
7.62
7.62
7.62
7.61
7.39
7.39
7.38
7.38
7.38
7.37
7.37
7.36
7.36
7.36
7.36
7.35
7.35
7.35
7.12
7.12
7.12
7.12
7.11
7.03
7.02
7.02
7.02
7.02
7.01
7.00
7.00
7.00
7.00
6.77
6.75
5.81
5.80
4.22
4.22
4.22
4.21
4.19
4.19
4.18
4.04
4.02
4.01
4.01
3.99
3.26
3.25
3.24
3.24
3.24
3.23
3.23
3.22
3.22
3.22
3.21
2.29
O
O
O
16
^{1}H NMR, $CDCl_3$, 400 MHz
2.00
3.02
1.00
1.05
0.99
0.98
1.03
2.03
1.00
3.07
12
11
10
9
8
7
6
5
4
3
2
1
0
f1 (ppm)

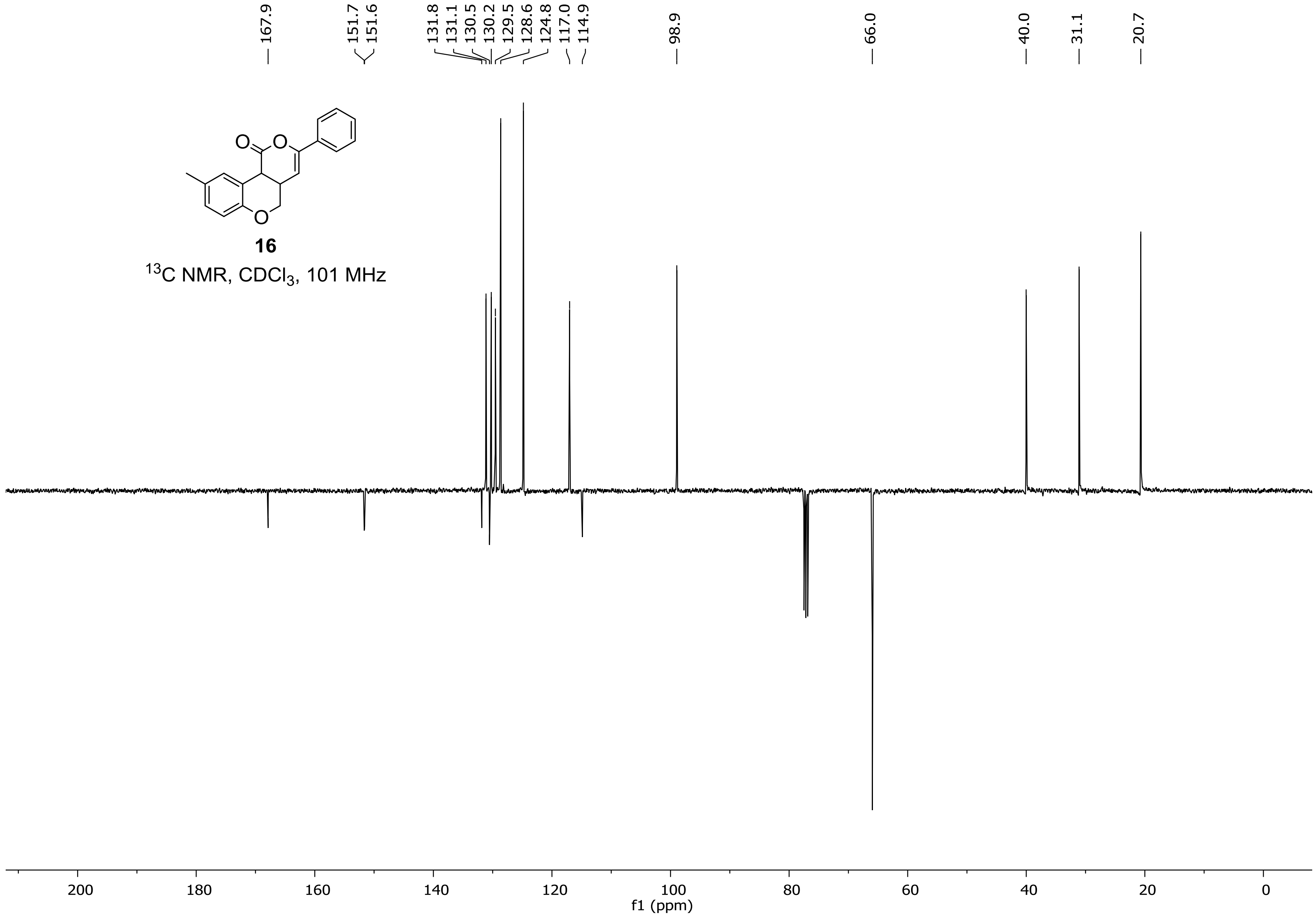

167.9
151.7
151.6
131.8
131.1
130.5
130.2
129.5
128.6
124.8
117.0
114.9
98.9
66.0
40.0
31.1
20.7
16
^{13}C NMR, $CDCl_3$, 101 MHz
200
180
160
140
120
100
80
60
40
20
0
f1 (ppm)

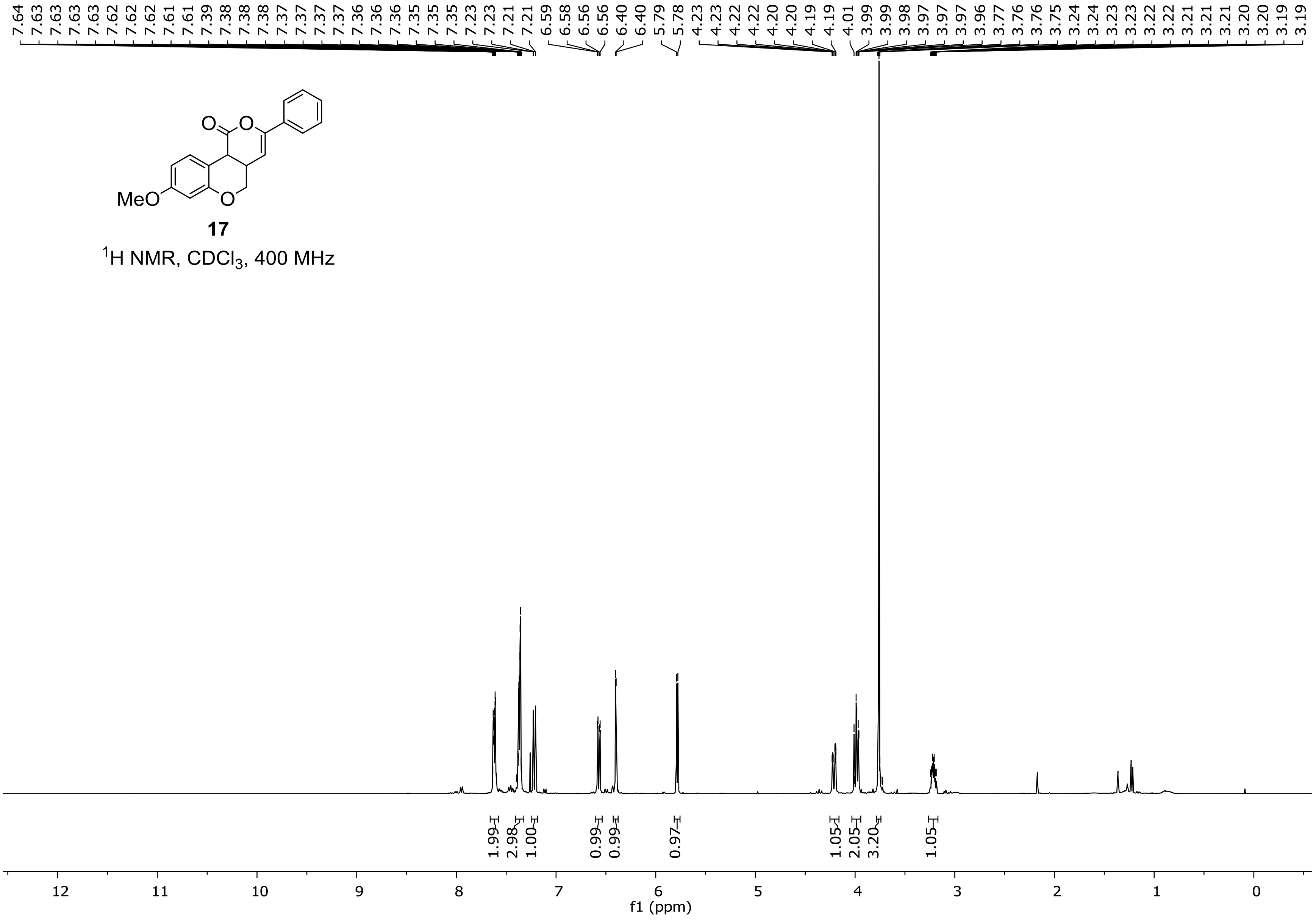

17

^{1}H NMR, $CDCl_3$, 400 MHz

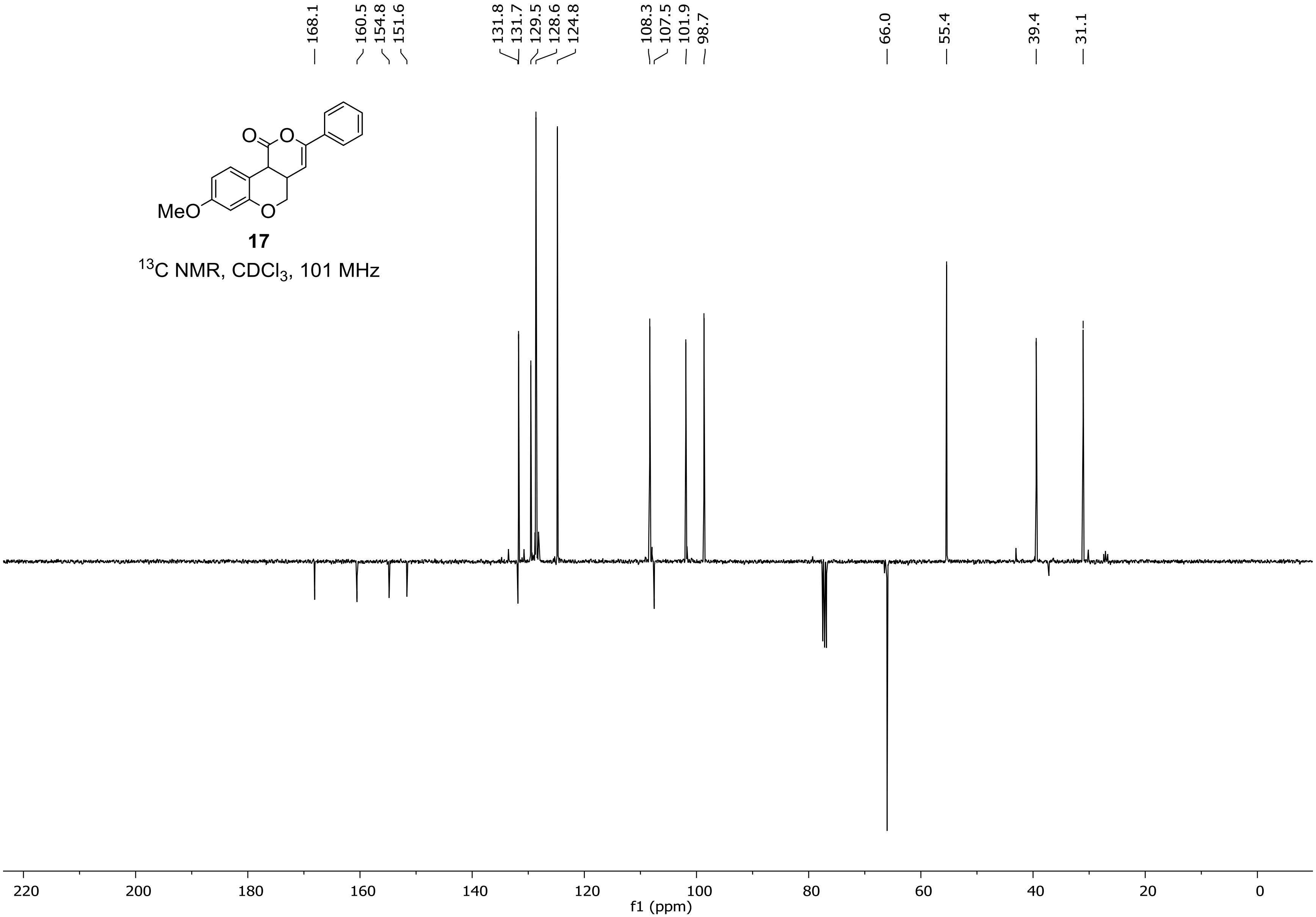

168.1
160.5
154.8
151.6
131.8
131.7
129.5
128.6
124.8
108.3
107.5
101.9
98.7
66.0
55.4
39.4
31.1
O
O
MeO
O
17
^{13}C NMR, $CDCl_3$, 101 MHz
220
200
180
160
140
120
100
80
60
40
20
0
f1 (ppm)

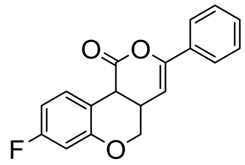

18

^{1}H NMR, $CDCl_3$, 400 MHz

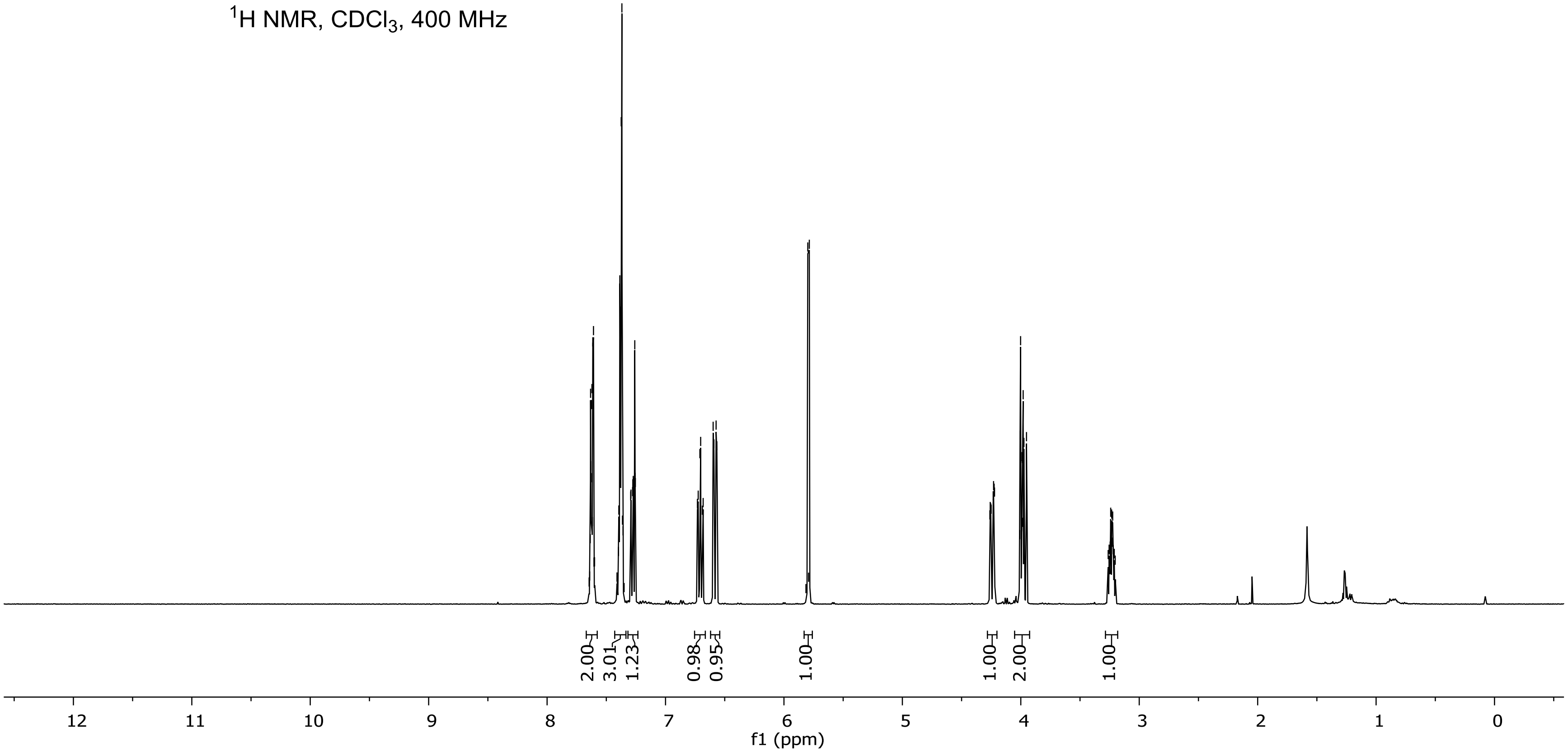

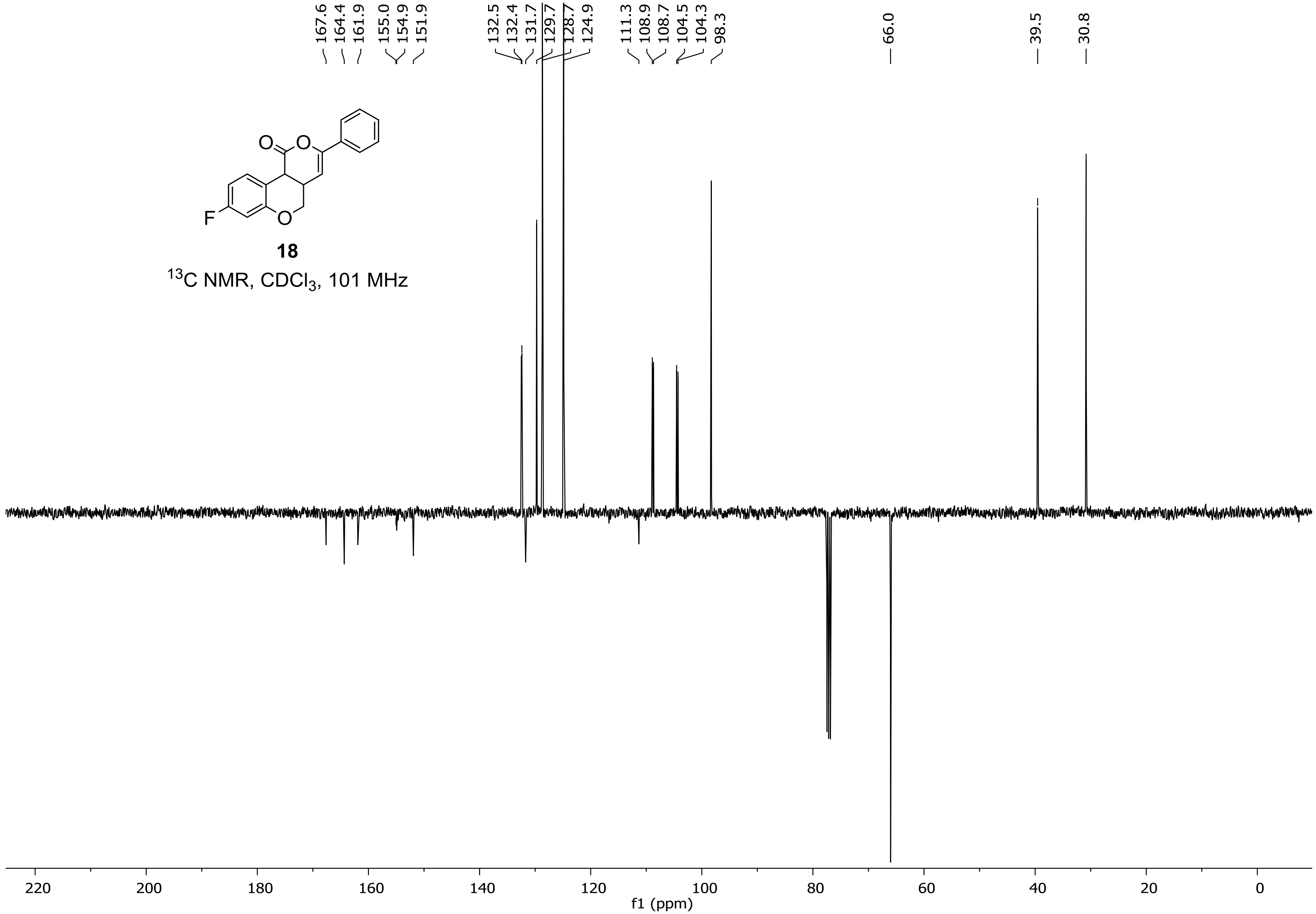
167.6
164.4
161.9
155.0
154.9
151.9
132.5
132.4
131.7
129.7
128.7
124.9
111.3
108.9
108.7
104.5
104.3
98.3
66.0
39.5
30.8
O
O
F
O
18
13C NMR, CDCl3, 101 MHz
220
200
180
160
140
120
100
80
60
40
20
0
f1 (ppm)

19

^{1}H NMR, $CDCl_3$, 400 MHz

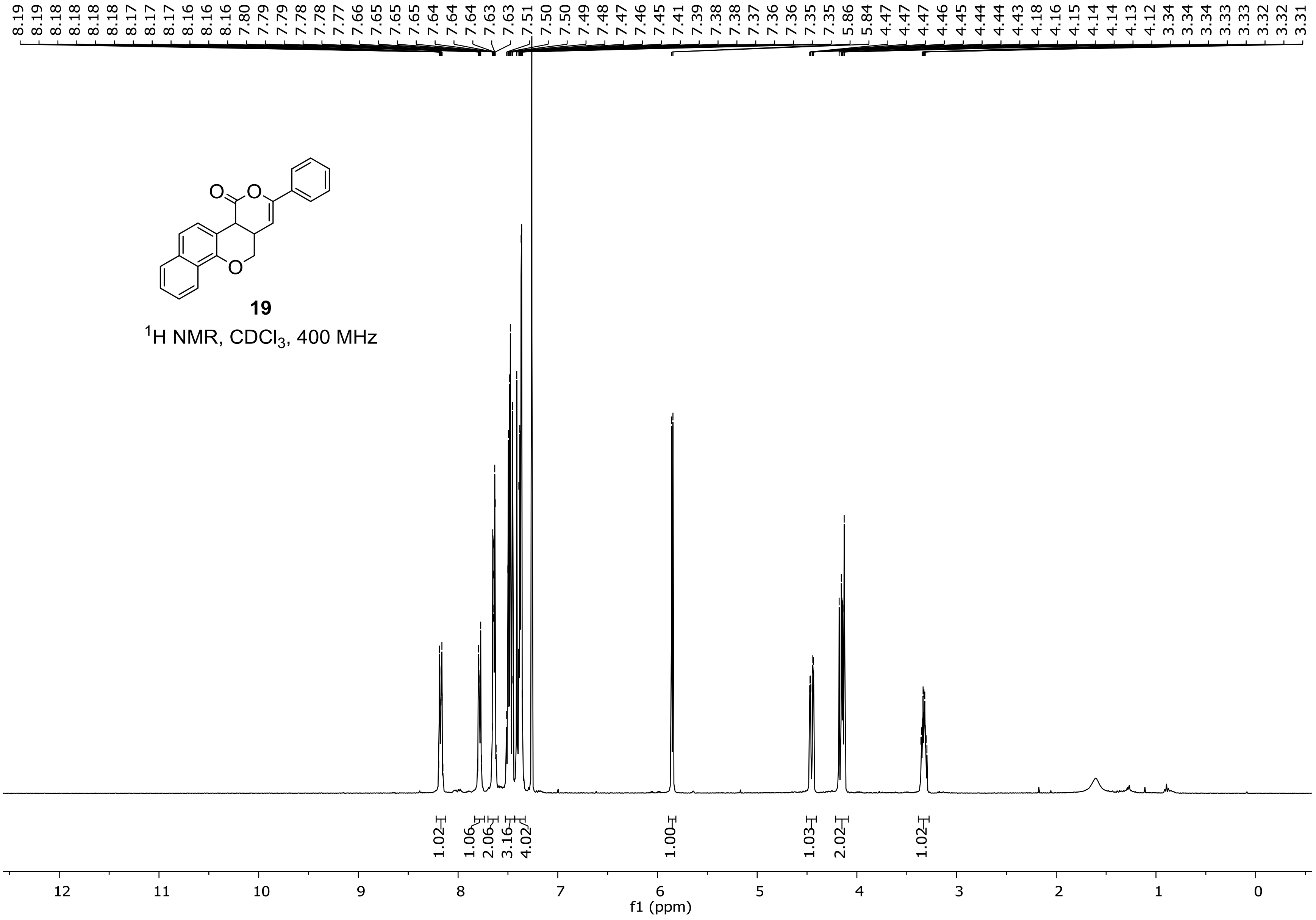

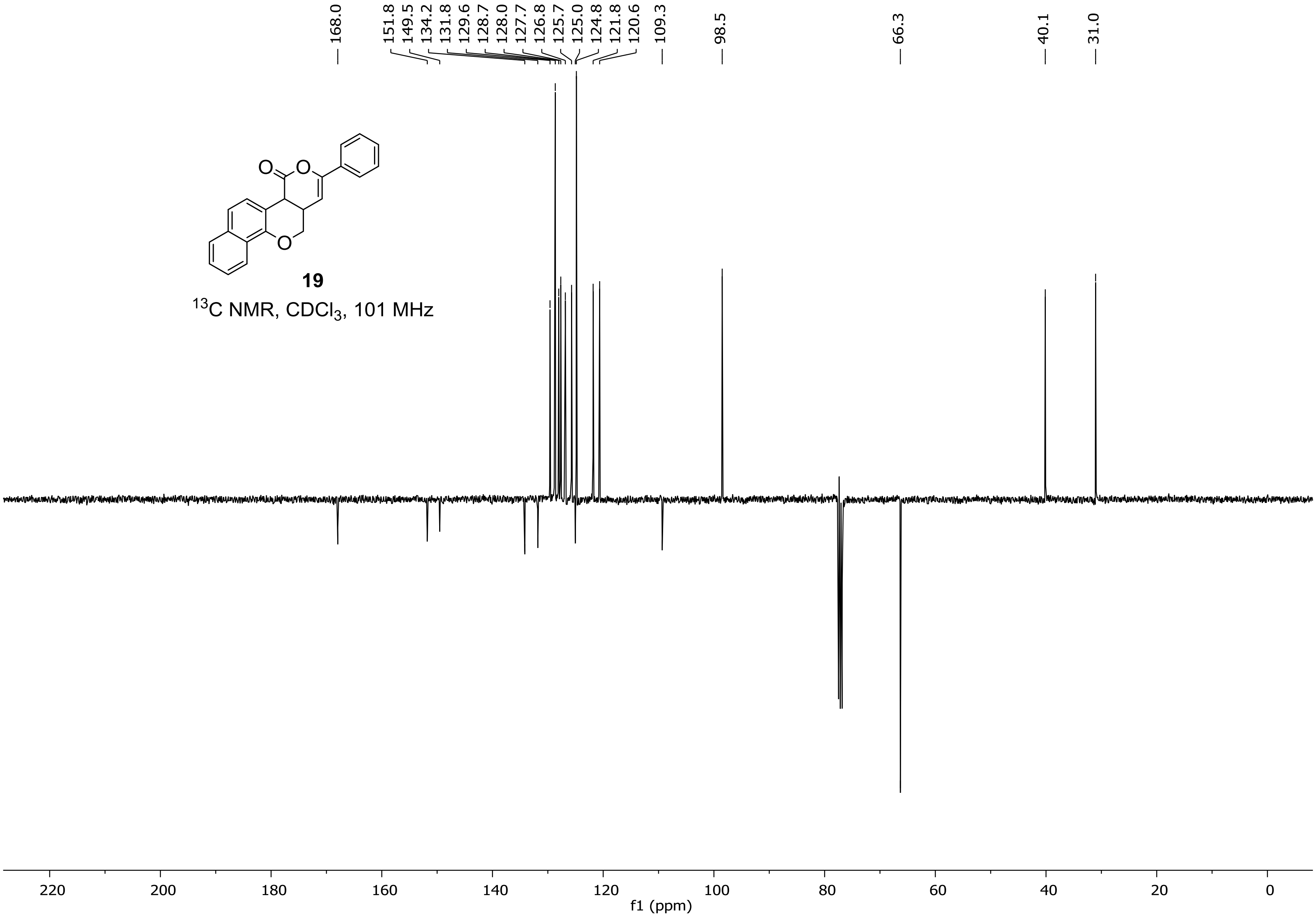
168.0
151.8
149.5
134.2
131.8
129.6
128.7
128.0
127.7
126.8
125.7
125.0
124.8
121.8
120.6
109.3
98.5
66.3
40.1
31.0
O
O
O
19
13C NMR, CDCl3, 101 MHz
220
200
180
160
140
120
100
80
60
40
20
0
f1 (ppm)

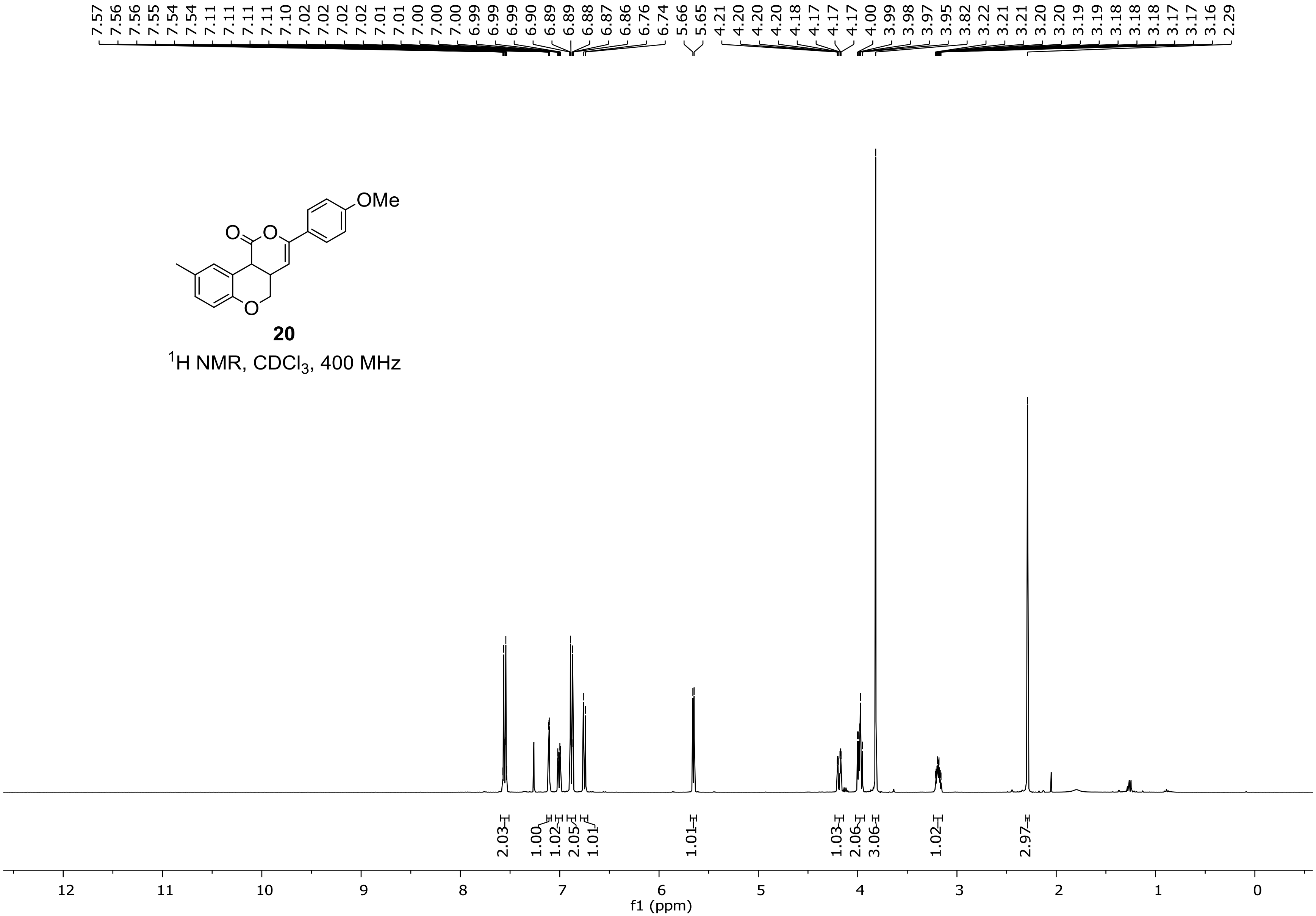
7.57 7.56 7.56 7.55 7.54 7.54 7.11 7.11 7.11 7.11 7.10 7.02 7.02 7.02 7.01 7.01 7.00 7.00 6.99 6.99 6.90 6.89 6.89 6.88 6.87 6.86 6.76 6.74 5.66 5.65 4.21 4.20 4.20 4.20 4.18 4.17 4.17 4.17 4.17 4.00 3.99 3.98 3.97 3.95 3.82 3.22 3.21 3.21 3.20 3.20 3.19 3.19 3.18 3.18 3.18 3.17 3.17 3.16 2.29
OMe
O
O
O
20
^{1}H NMR, $CDCl_3$, 400 MHz
2.03 1.00 1.02 2.05 1.01 1.01 1.03 2.06 3.06 1.02 2.97
12 11 10 9 8 7 6 5 4 3 2 1 0
f1 (ppm)

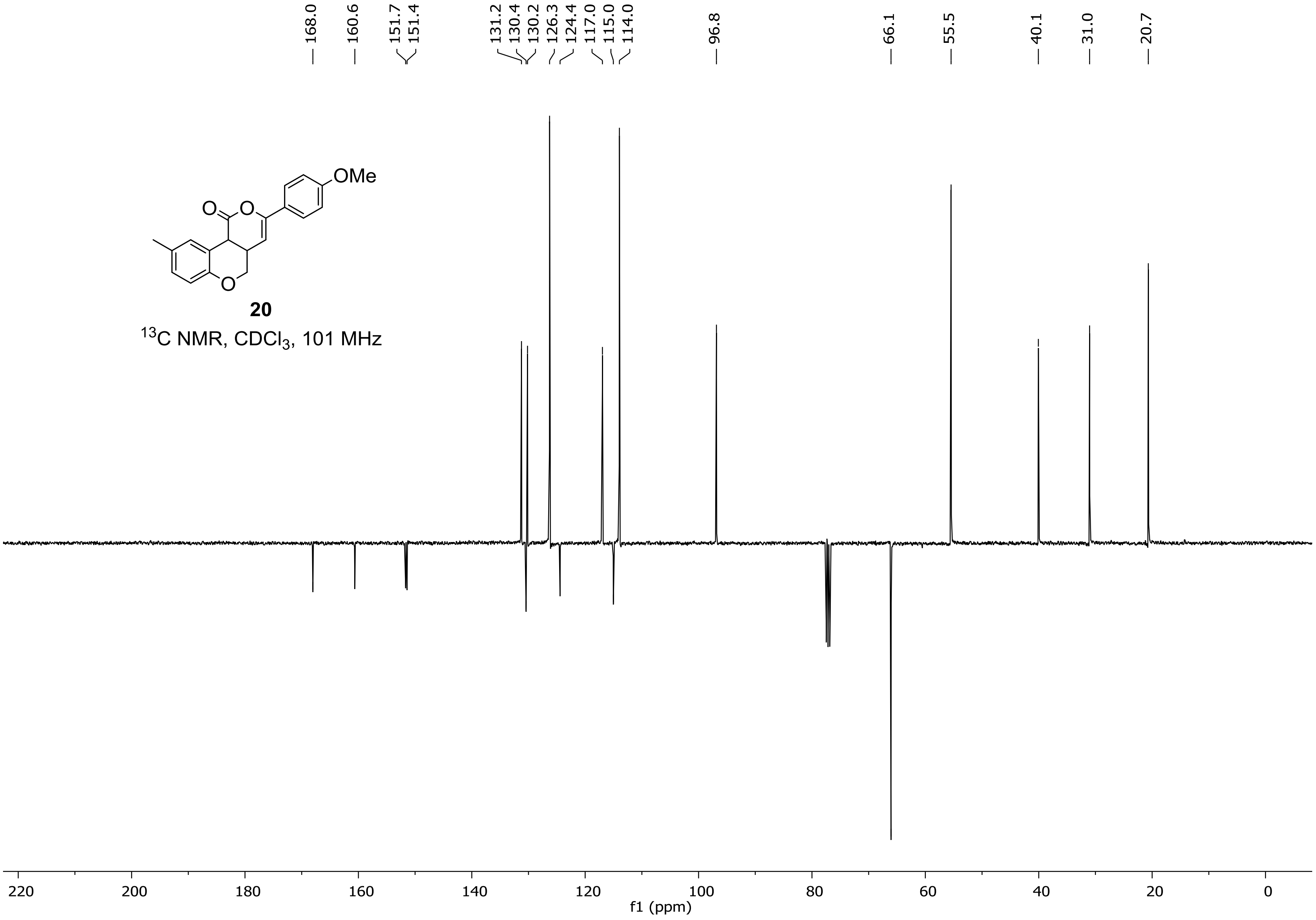

OMe
O
O
O
20
^{13}C NMR, $CDCl_3$, 101 MHz
168.0
160.6
151.7
151.4
131.2
130.4
130.2
126.3
124.4
117.0
115.0
114.0
96.8
66.1
55.5
40.1
31.0
20.7
220
200
180
160
140
120
100
80
60
40
20
0
f1 (ppm)

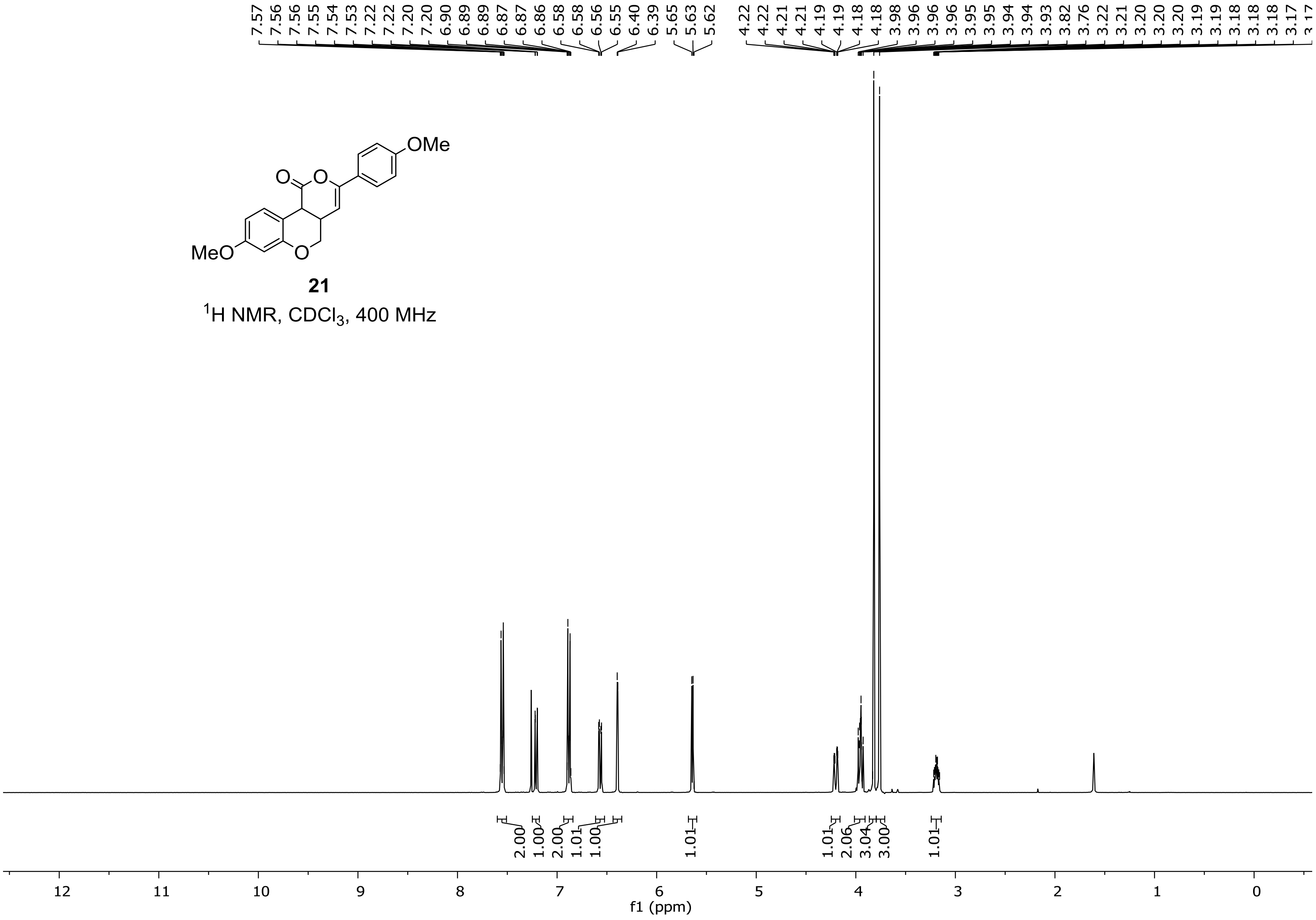
7.57 7.56 7.56 7.55 7.54 7.53 7.22 7.22 7.20 7.20 6.90 6.89 6.89 6.87 6.87 6.86 6.58 6.58 6.56 6.55 6.40 6.39 5.65 5.63 5.62
4.22 4.22 4.21 4.21 4.19 4.19 4.18 4.18 3.98 3.96 3.96 3.96 3.95 3.95 3.94 3.94 3.93 3.82 3.76 3.22 3.21 3.20 3.20 3.20 3.19 3.19 3.18 3.18 3.18 3.17
OMe
O
O
MeO
O
21
^{1}H NMR, CDCl$_3$, 400 MHz
2.00 1.00 2.00 1.01 1.00 1.01 1.01 2.06 3.04 3.00 1.01
12 11 10 9 8 7 6 5 4 3 2 1 0
f1 (ppm)

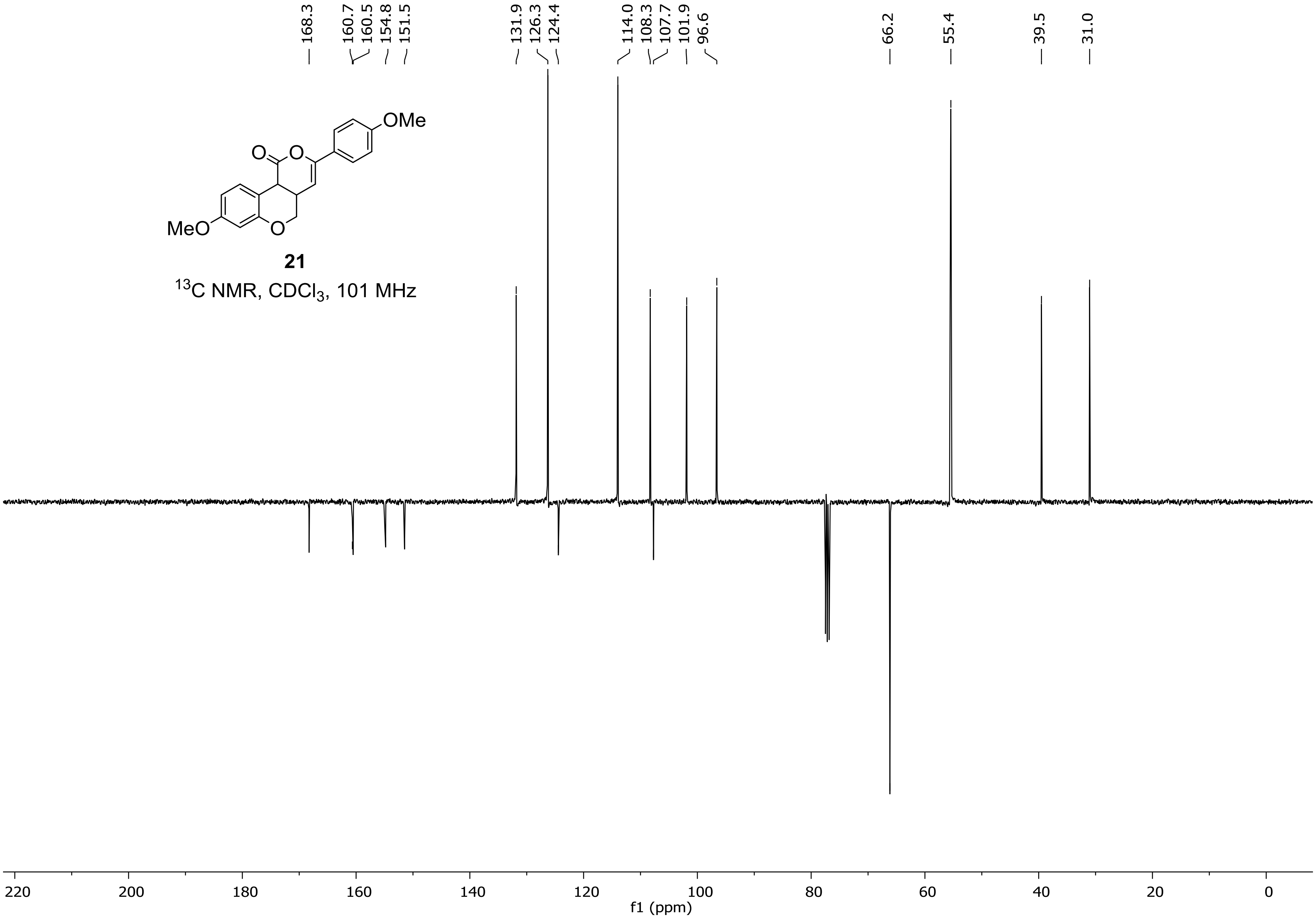

168.3
160.7
160.5
154.8
151.5
131.9
126.3
124.4
114.0
108.3
107.7
101.9
96.6
66.2
55.4
39.5
31.0
OMe
O
O
MeO
O
21
^{13}C NMR, CDCl$_3$, 101 MHz
220
200
180
160
140
120
100
80
60
40
20
0
f1 (ppm)

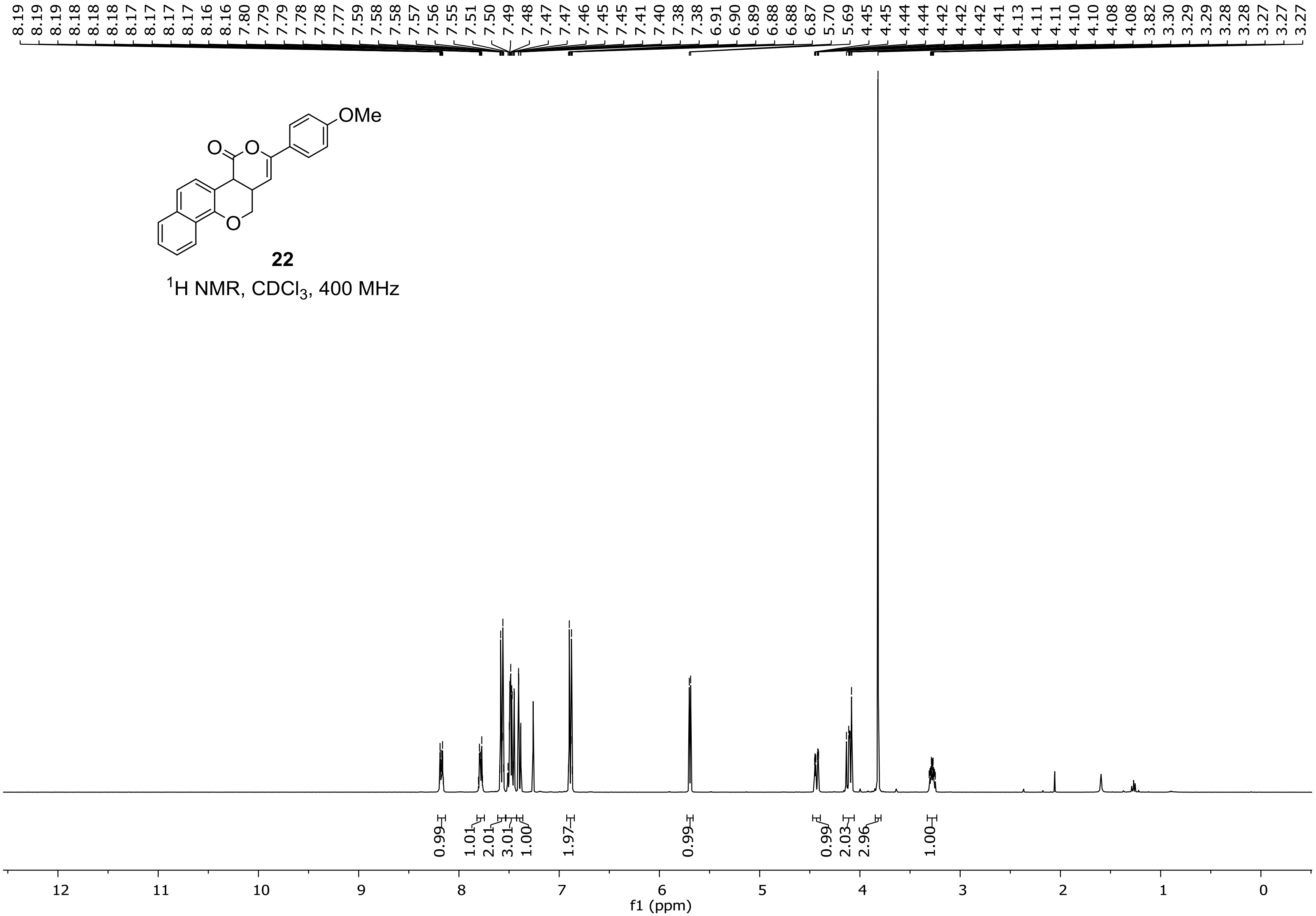
OMe
O
O
O
22
^{1}H NMR, $CDCl_3$, 400 MHz
0.99 1.01 2.01 3.01 1.00 1.97 0.99 0.99 2.03 2.96 1.00
f1 (ppm)

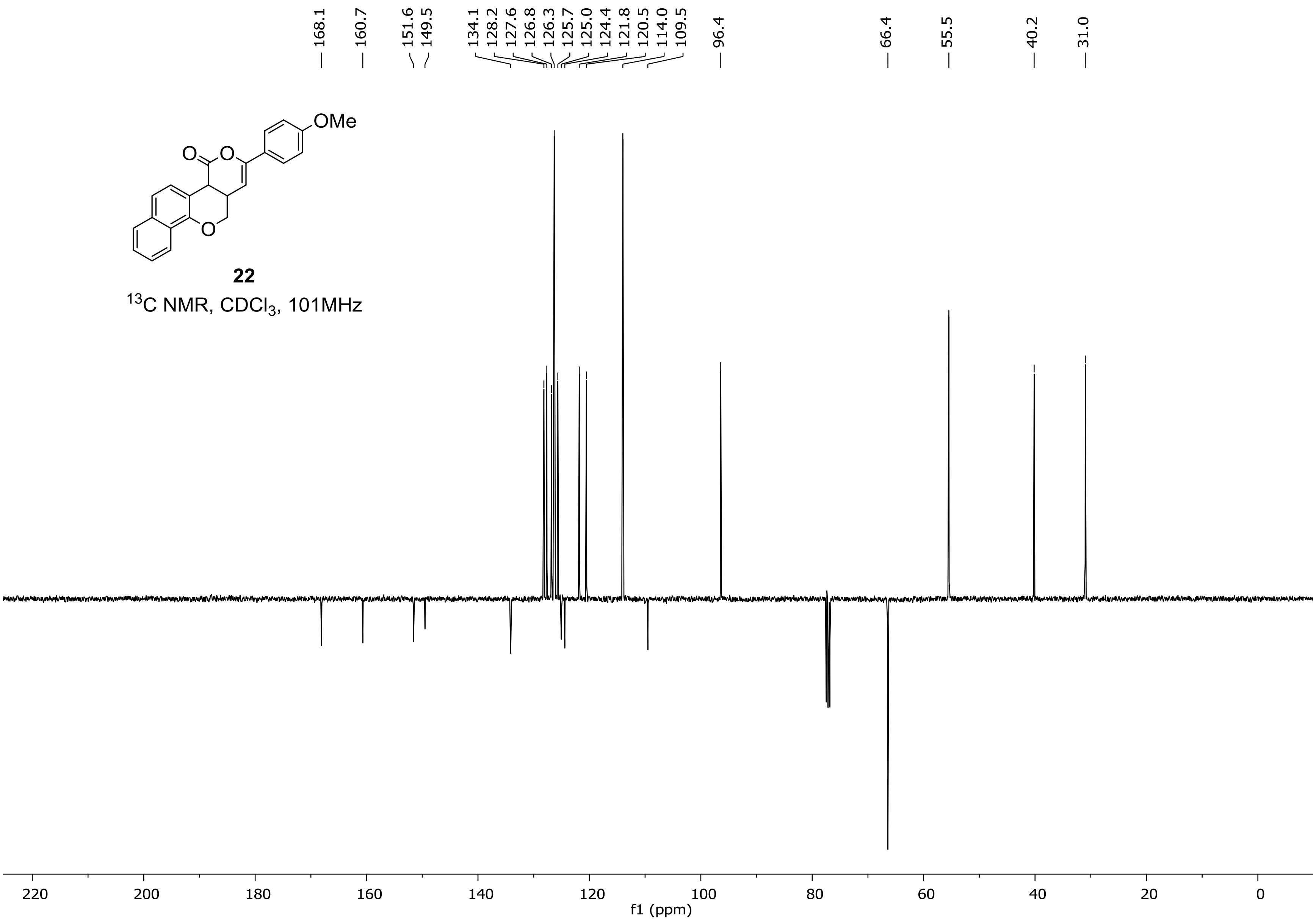
168.1
160.7
151.6
149.5
134.1
128.2
127.6
126.8
126.3
125.7
125.0
124.4
121.8
120.5
114.0
109.5
96.4
66.4
55.5
40.2
31.0
OMe
O
O
O
22
^{13}C NMR, $CDCl_3$, 101MHz
220
200
180
160
140
120
100
80
60
40
20
0
f1 (ppm)

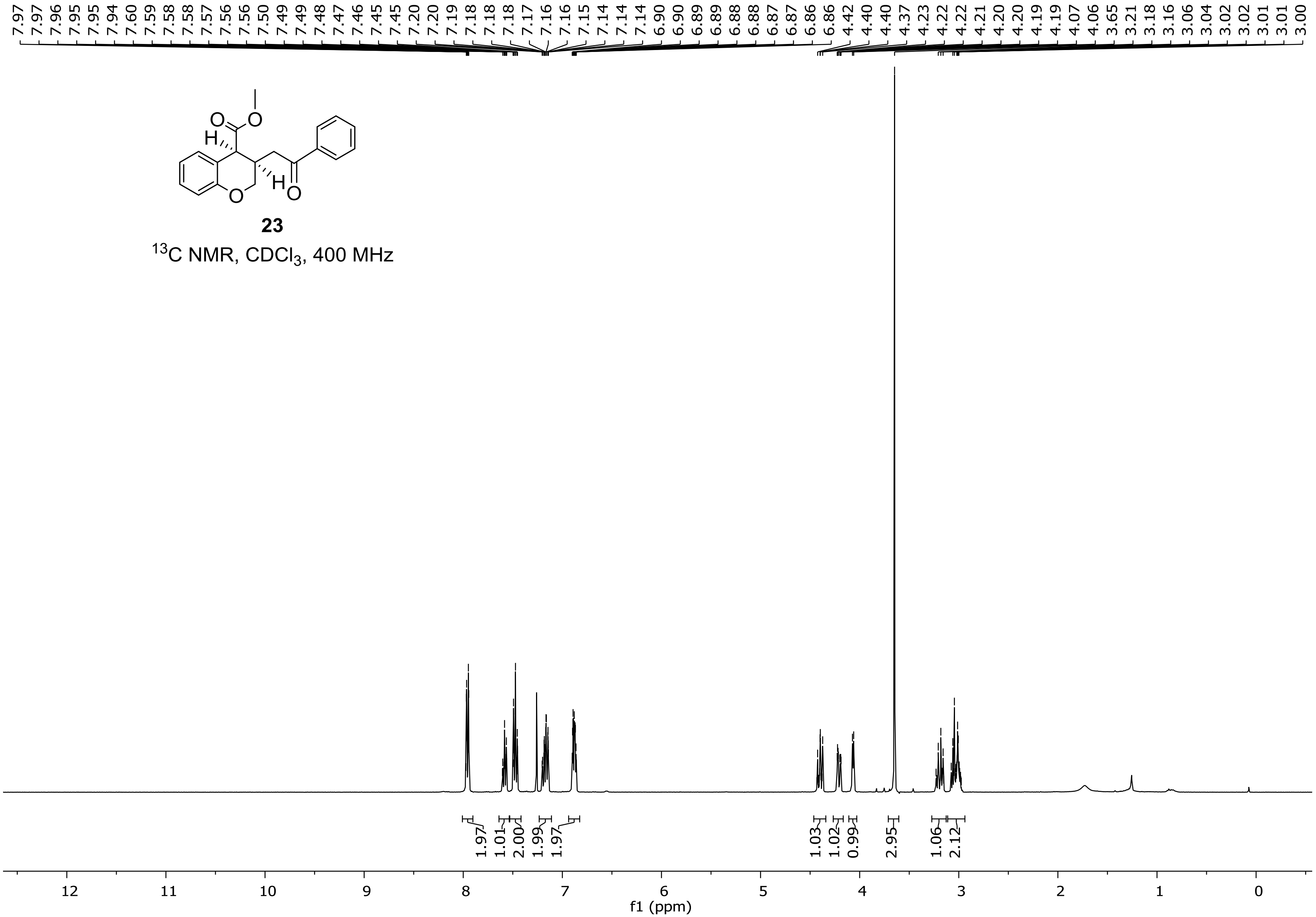
7.97 7.97 7.96 7.95 7.95 7.94 7.60 7.59 7.58 7.58 7.57 7.56 7.56 7.50 7.49 7.49 7.48 7.47 7.46 7.45 7.45 7.20 7.20 7.19 7.18 7.18 7.18 7.17 7.16 7.16 7.15 7.14 7.14 7.14 6.90 6.90 6.89 6.89 6.88 6.88 6.87 6.87 6.86 6.86 4.42 4.40 4.40 4.37 4.23 4.22 4.22 4.21 4.20 4.20 4.19 4.19 4.07 4.06 3.65 3.21 3.18 3.16 3.06 3.04 3.02 3.02 3.01 3.01 3.00
O
O
H
H
O
O
23
^{13}C NMR, $CDCl_3$, 400 MHz
1.97 1.01 2.00 1.99 1.97 1.03 1.02 0.99 2.95 1.06 2.12
12 11 10 9 8 7 6 5 4 3 2 1 0
f1 (ppm)

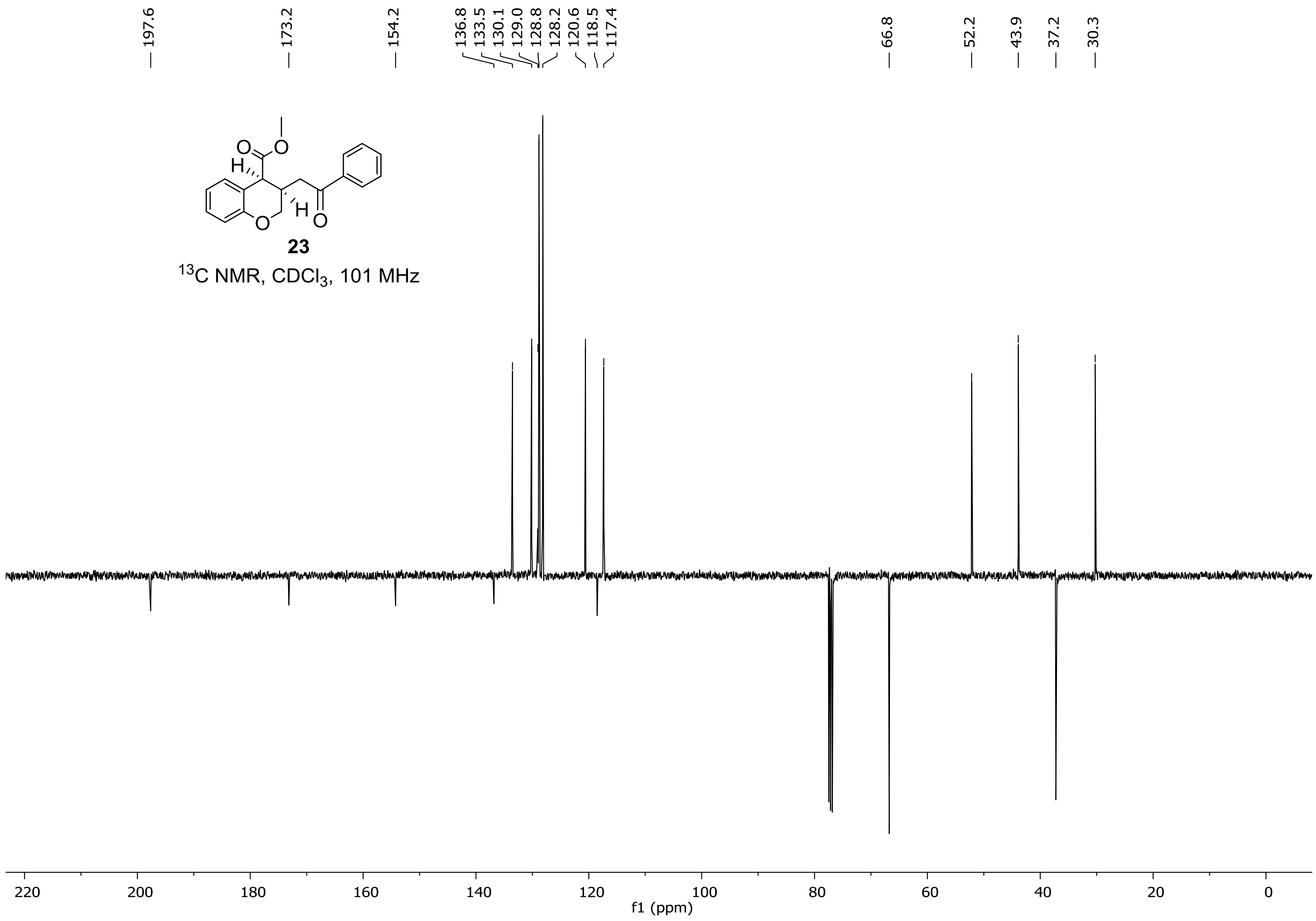
197.6
173.2
154.2
136.8
133.5
130.1
129.0
128.8
128.2
120.6
118.5
117.4
66.8
52.2
43.9
37.2
30.3
23
13C NMR, CDCl3, 101 MHz
220
200
180
160
140
120
100
80
60
40
20
0
f1 (ppm)

24

^{1}H NMR, $CDCl_3$, 400 MHz

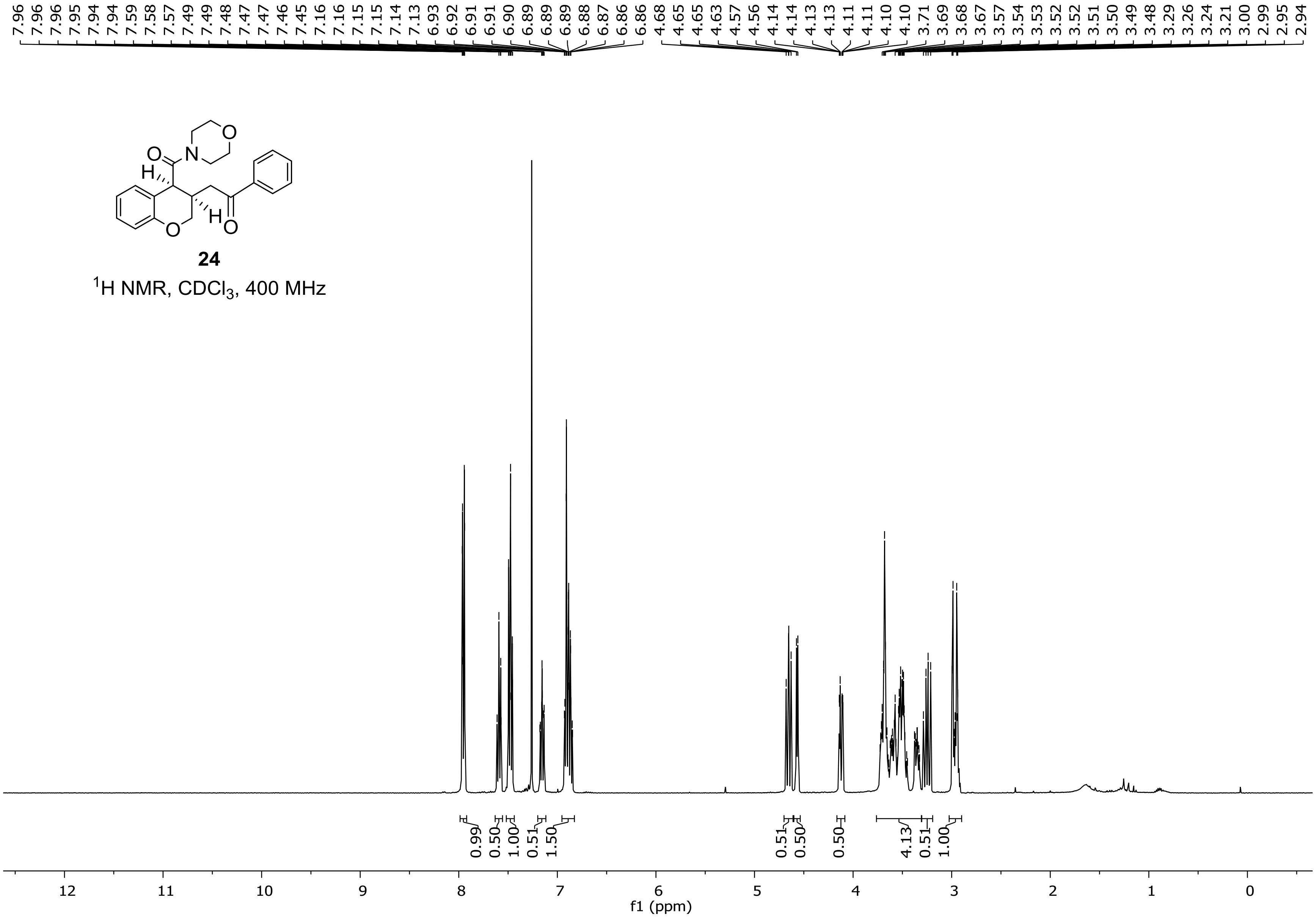

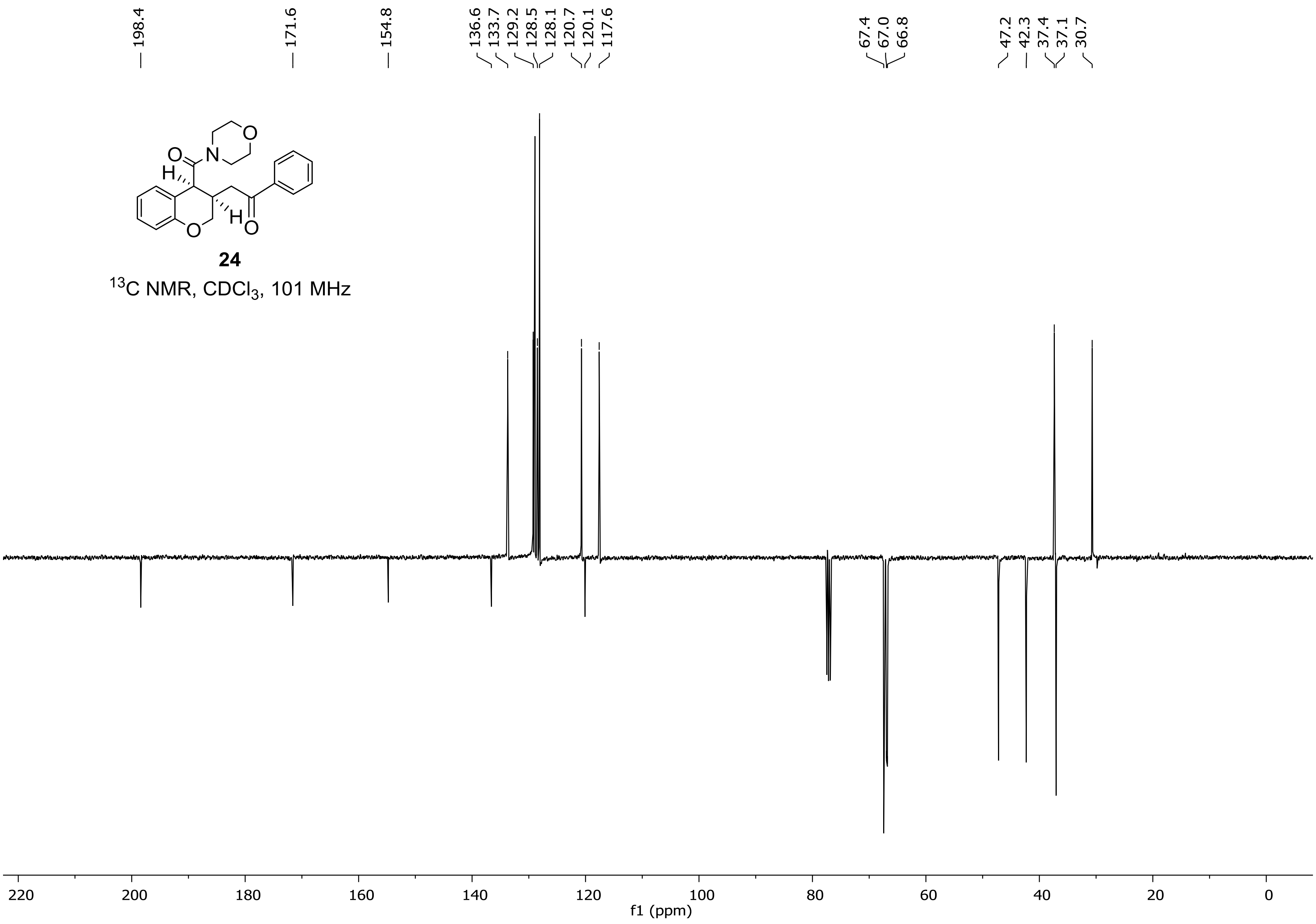

198.4
171.6
154.8
136.6
133.7
129.2
128.5
128.1
120.7
120.1
117.6
67.4
67.0
66.8
47.2
42.3
37.4
37.1
30.7
24
^{13}C NMR, $CDCl_3$, 101 MHz
220
200
180
160
140
120
100
80
60
40
20
0
f1 (ppm)

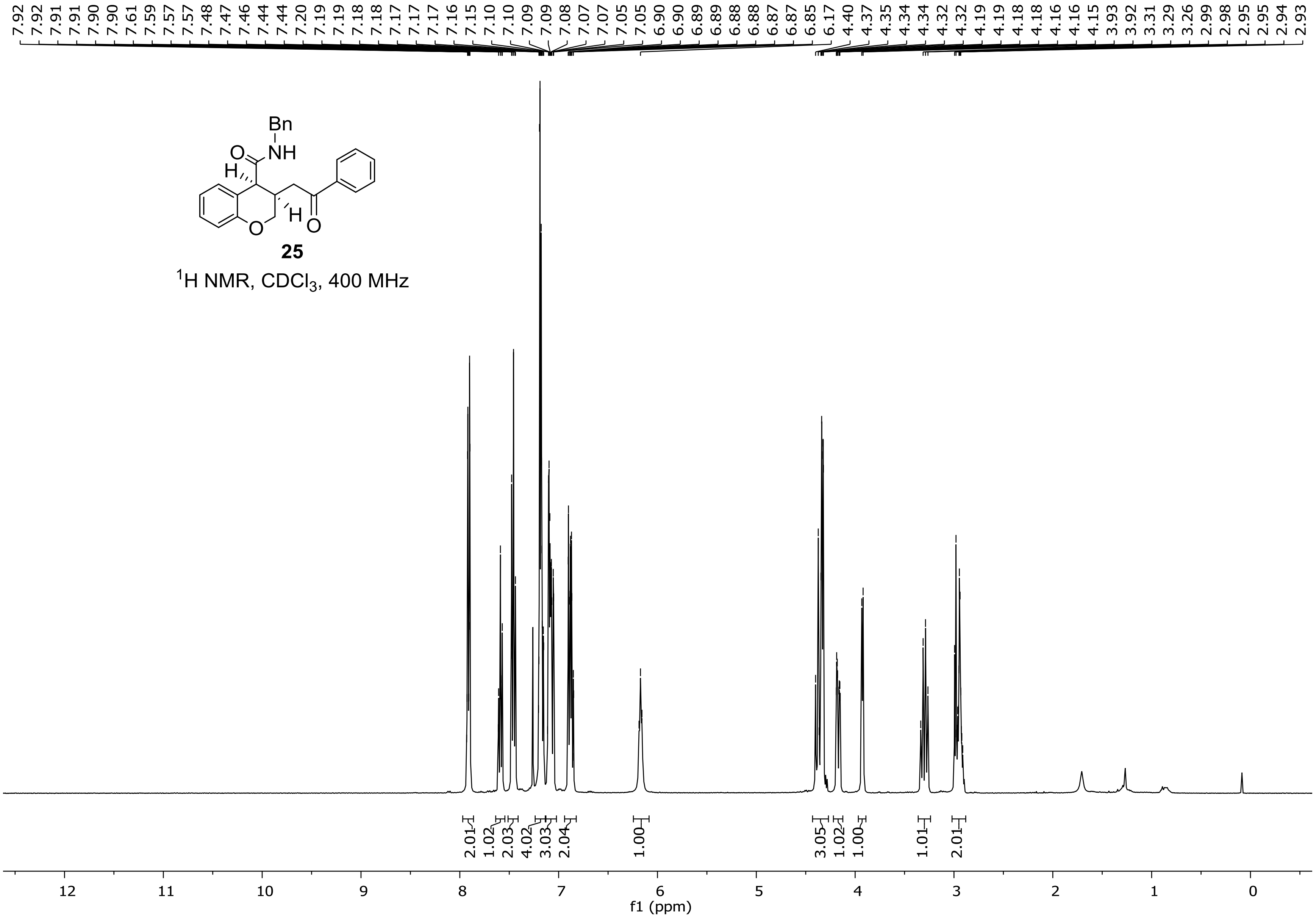
7.92 7.92 7.91 7.91 7.90 7.90 7.61 7.59 7.57 7.57 7.48 7.47 7.46 7.44 7.44 7.20 7.19 7.19 7.18 7.18 7.17 7.17 7.17 7.16 7.15 7.10 7.10 7.09 7.09 7.08 7.07 7.07 7.05 7.05 6.90 6.90 6.89 6.89 6.88 6.88 6.87 6.87 6.85 6.17 4.40 4.37 4.35 4.34 4.34 4.32 4.32 4.19 4.19 4.18 4.18 4.16 4.16 4.15 3.93 3.92 3.31 3.29 3.26 2.99 2.98 2.95 2.95 2.94 2.93
Bn
NH
O
H
H
O
O
25
^{1}H NMR, CDCl$_3$, 400 MHz
2.01 1.02 2.03 4.02 3.03 2.04 1.00 3.05 1.02 1.00 1.01 2.01
12 11 10 9 8 7 6 5 4 3 2 1 0
f1 (ppm)

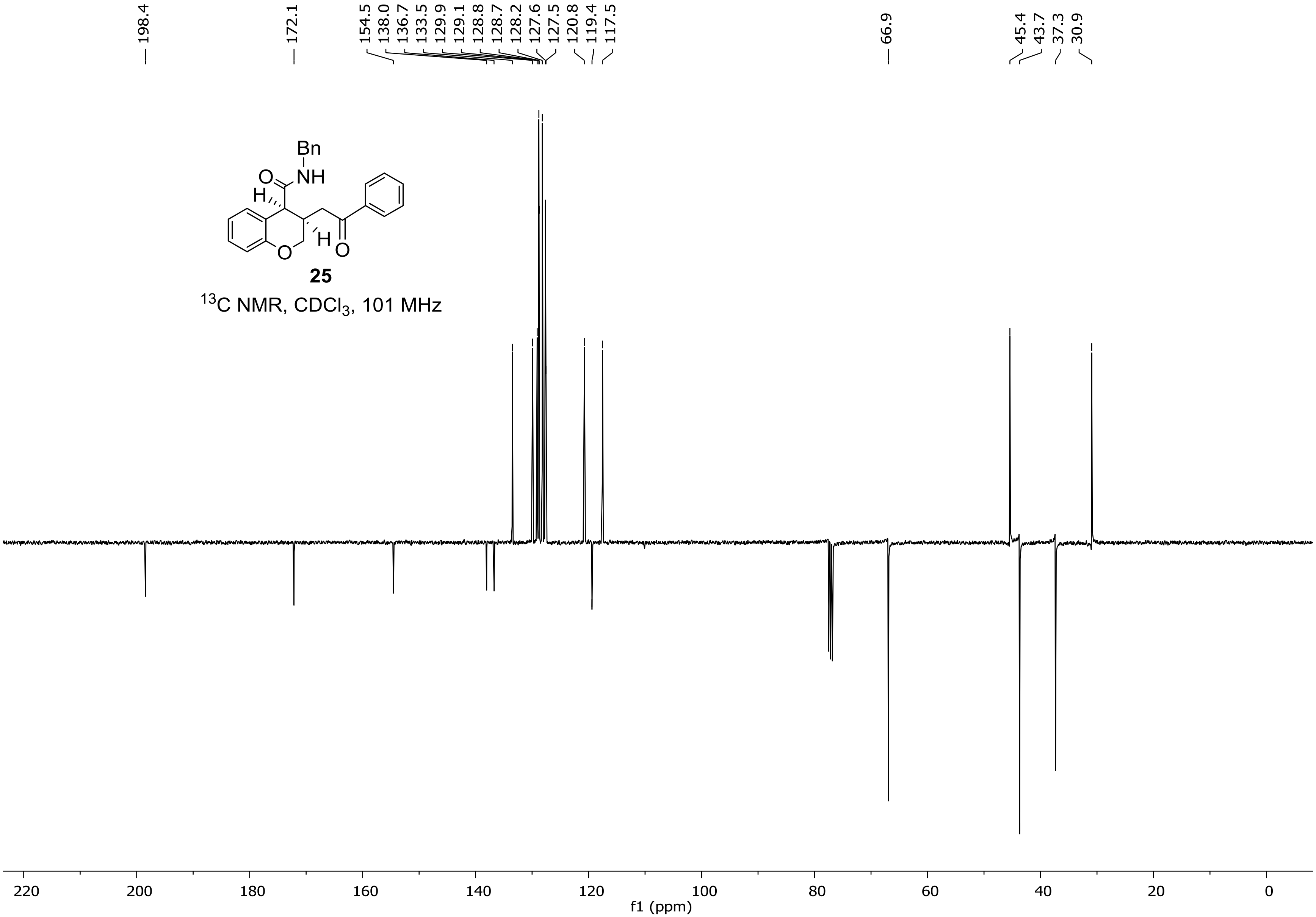
198.4
172.1
154.5
138.0
136.7
133.5
129.9
129.1
128.8
128.7
128.2
127.6
127.5
120.8
119.4
117.5
66.9
45.4
43.7
37.3
30.9
Bn
NH
O
H
H
O
O
25
^{13}C NMR, $CDCl_3$, 101 MHz
220
200
180
160
140
120
100
80
60
40
20
0
f1 (ppm)

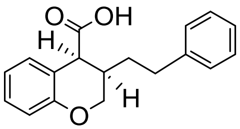

26

^{1}H NMR, $CDCl_3$, 400 MHz

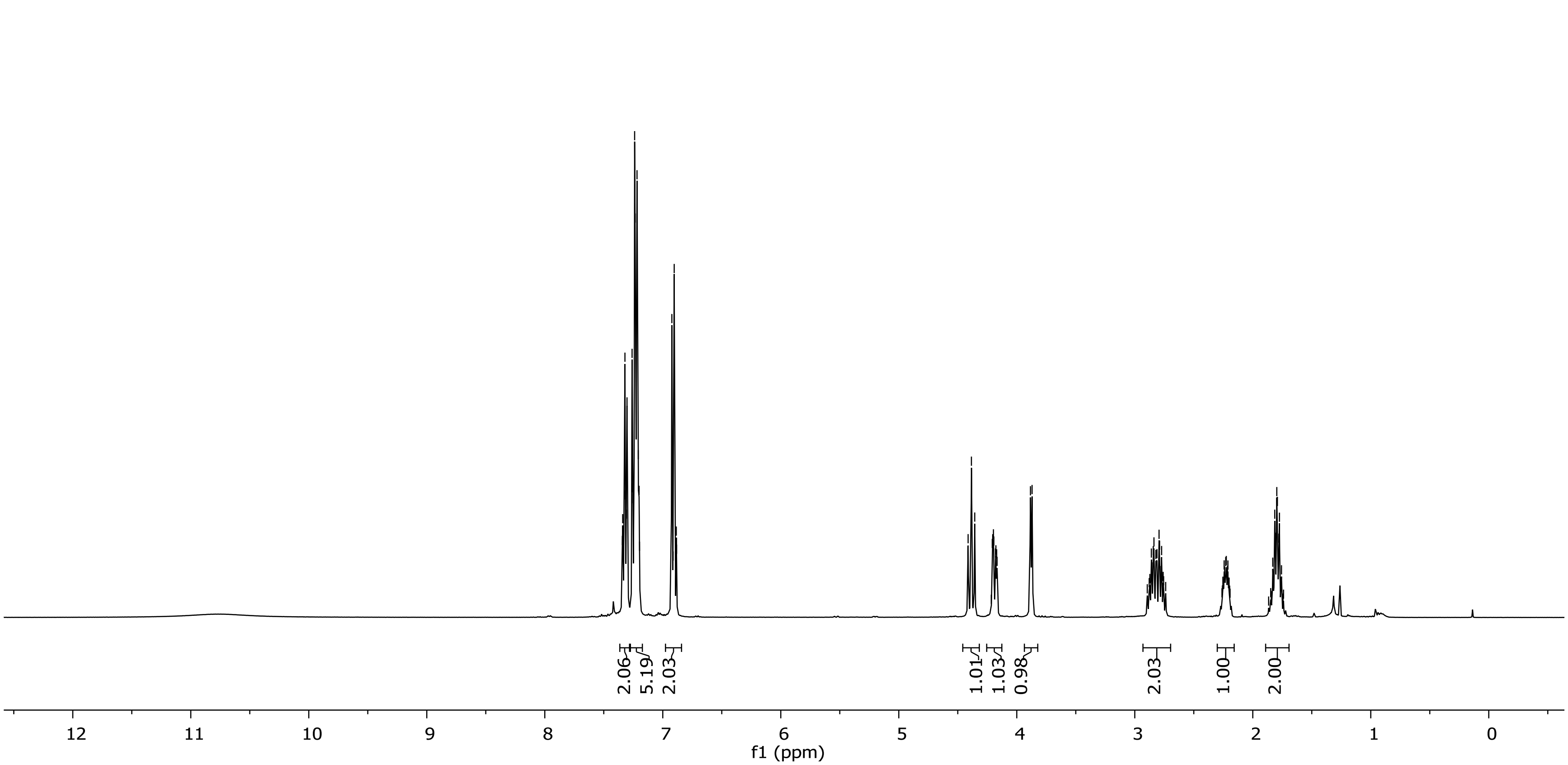

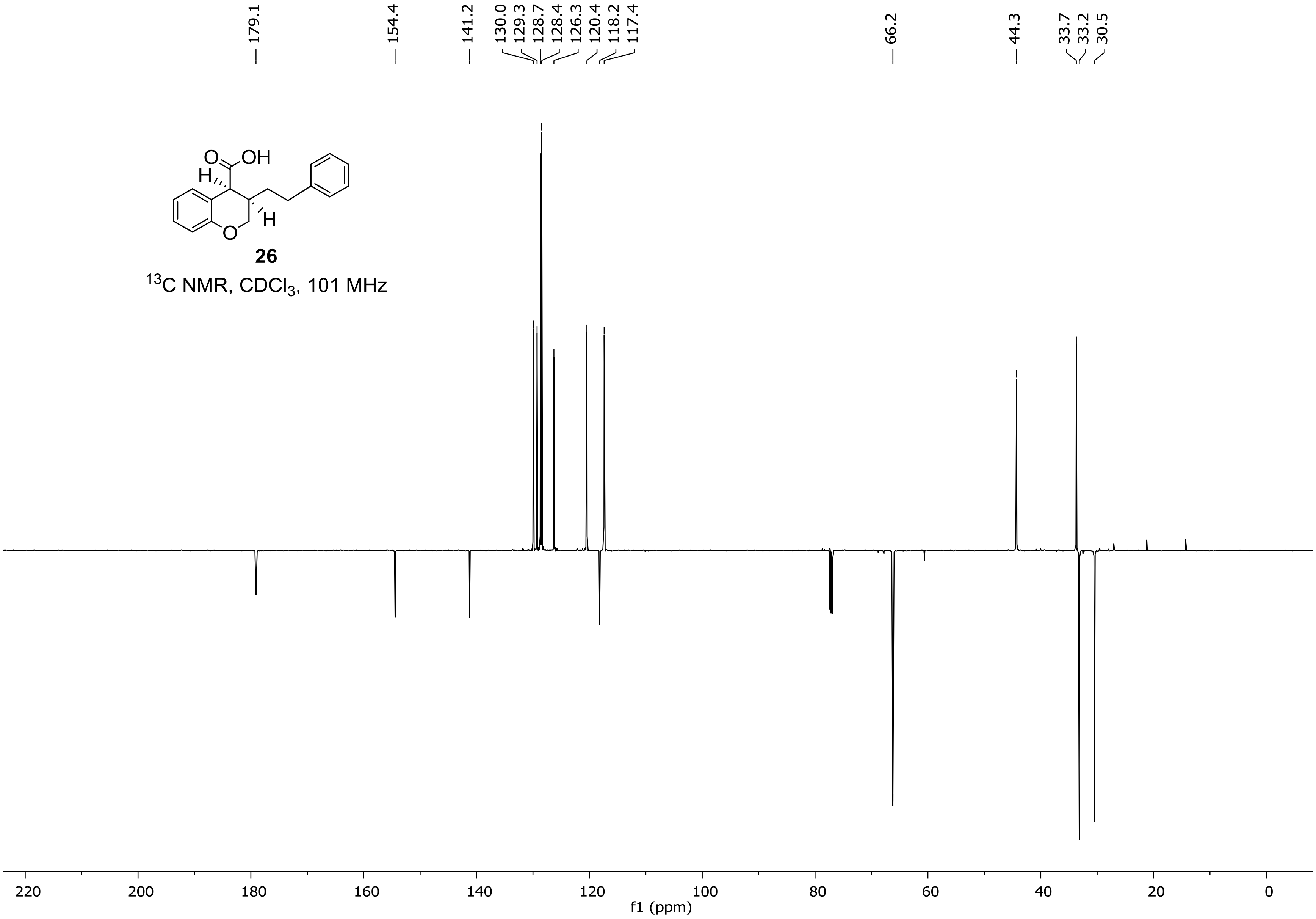

179.1
154.4
141.2
130.0
129.3
128.7
128.4
126.3
120.4
118.2
117.4
66.2
44.3
33.7
33.2
30.5
26
^{13}C NMR, $CDCl_3$, 101 MHz
220
200
180
160
140
120
100
80
60
40
20
0
f1 (ppm)

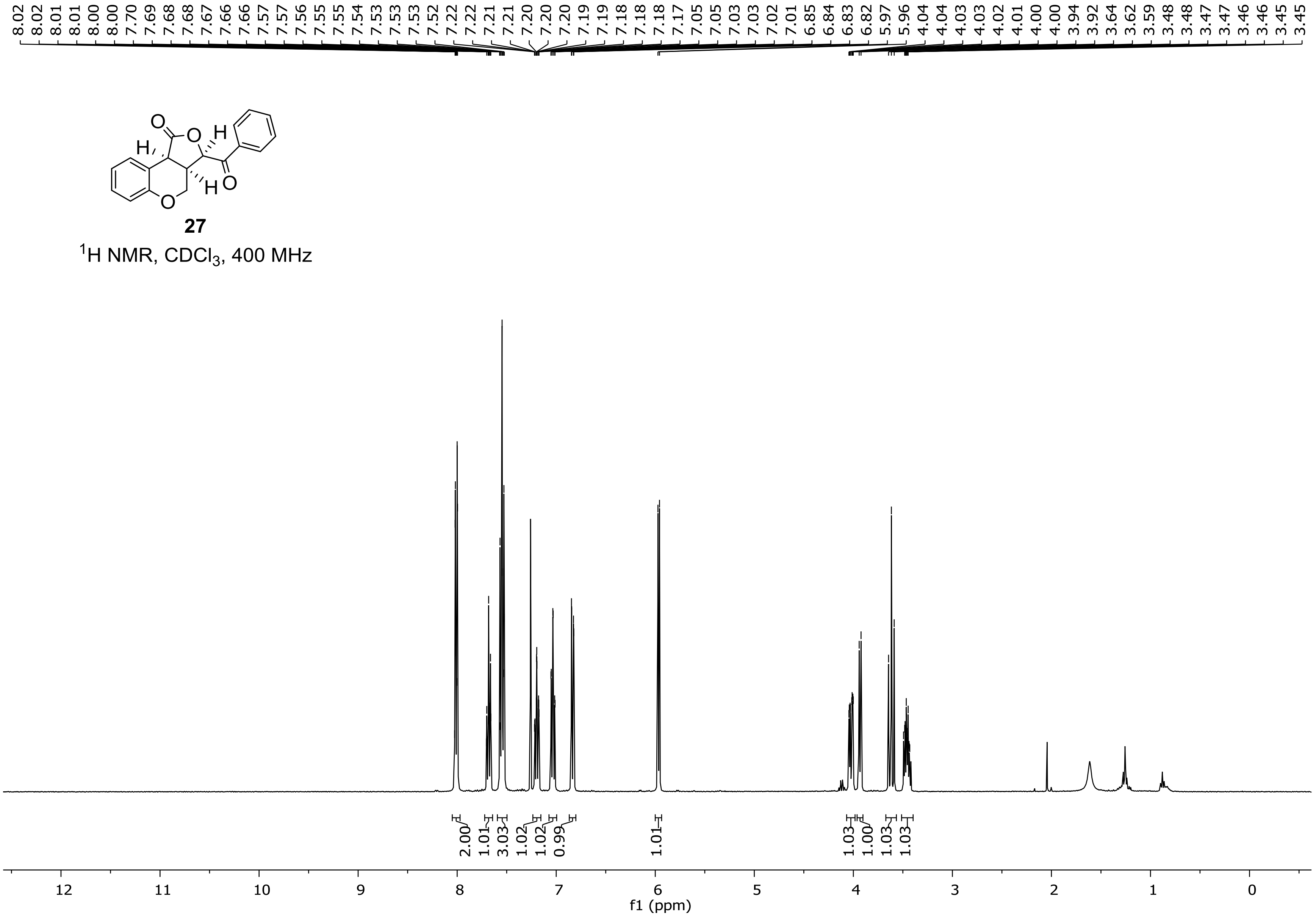

27
^{1}H NMR, $CDCl_3$, 400 MHz
f1 (ppm)

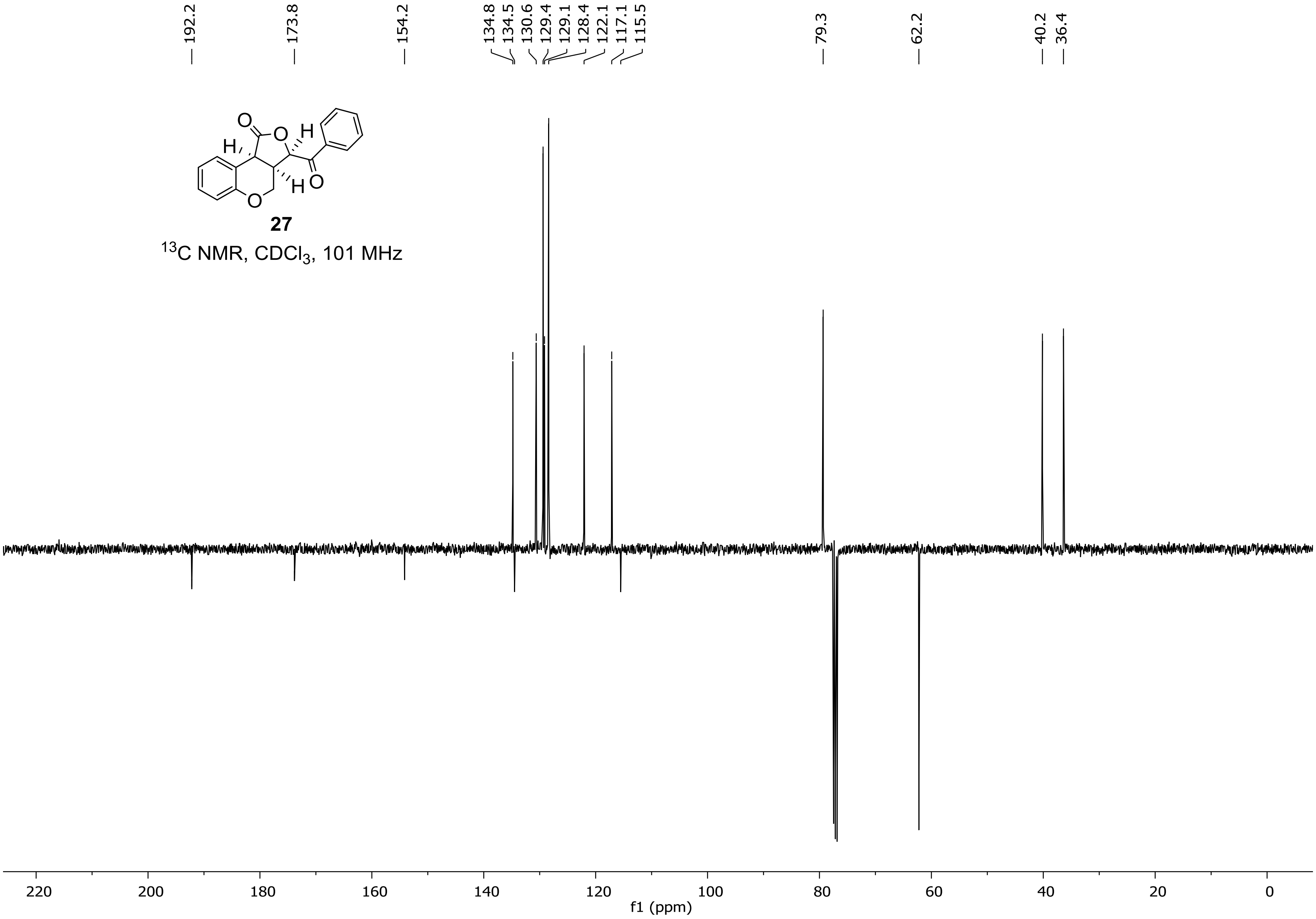

27

^{13}C NMR, $CDCl_3$, 101 MHz

HPLC Data for **1**: Chiralpak AD-H (95:5 hexane : IPA, flow rate 1.00 mLmin^{-1}, 254 nm, 40 °C) t_R (4a*R*,10b*S*): 20.0 min, t_R (4a*S*,10b*R*) : 14.3 min, 98:2 er.

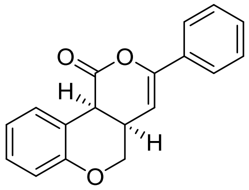

PDA Ch1 254nm

Peak#	Ret. Time	Area%
1	14.484	49.392
2	20.243	50.608
Total		100.000

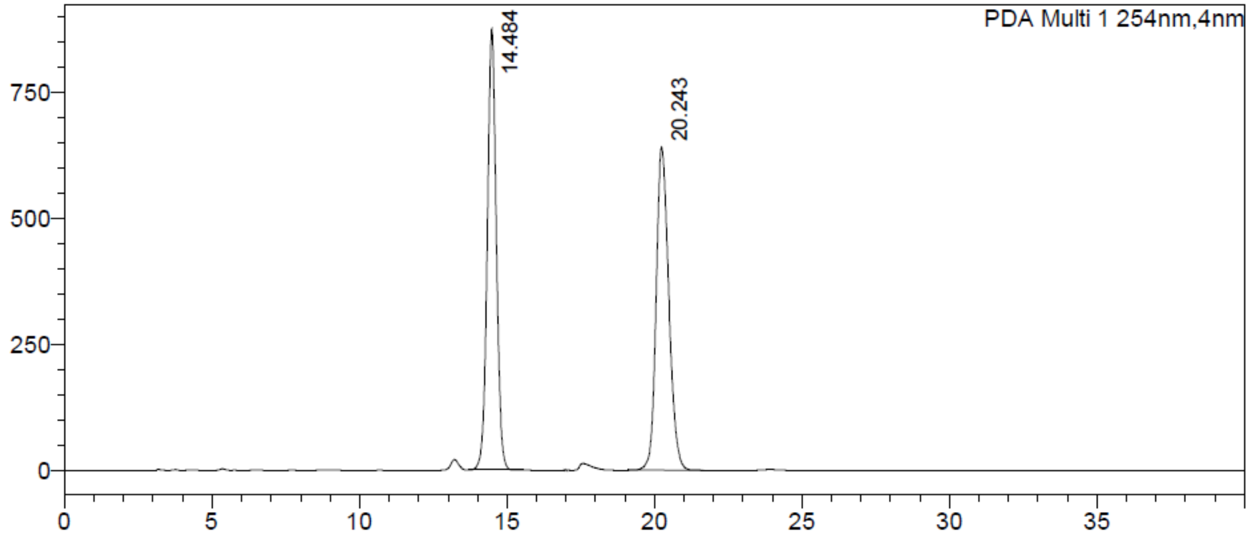

PDA Ch3 254nm

Peak#	Ret. Time	Area%
1	14.333	1.787
2	20.047	98.213
Total		100.000

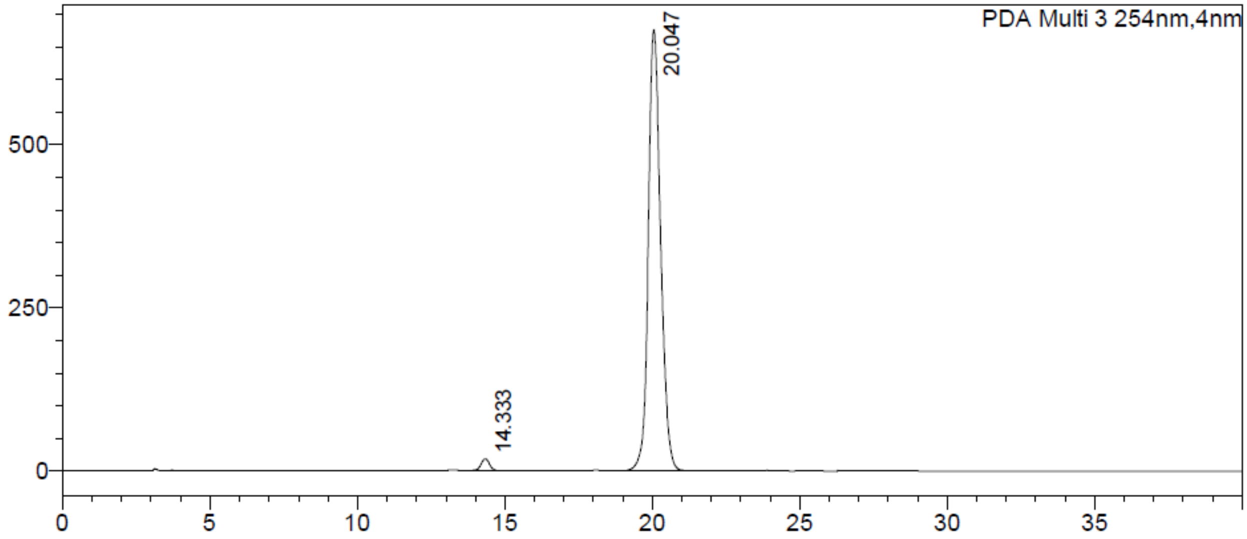

HPLC Data for **8**: Chiralpak AD-H (95:5 hexane : IPA, flow rate 1.00 mLmin^{-1}, 254 nm, 40 °C) t_R (4a*R*,10b*S*): 20.2 min, t_R (4a*S*,10b*R*) : 16.0 min, 96:4 er.

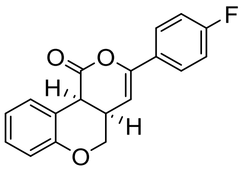

PDA Ch1 254nm

Peak#	Ret. Time	Area%
1	15.053	0.920
2	16.407	49.079
3	19.440	0.755
4	20.780	49.247
Total		100.000

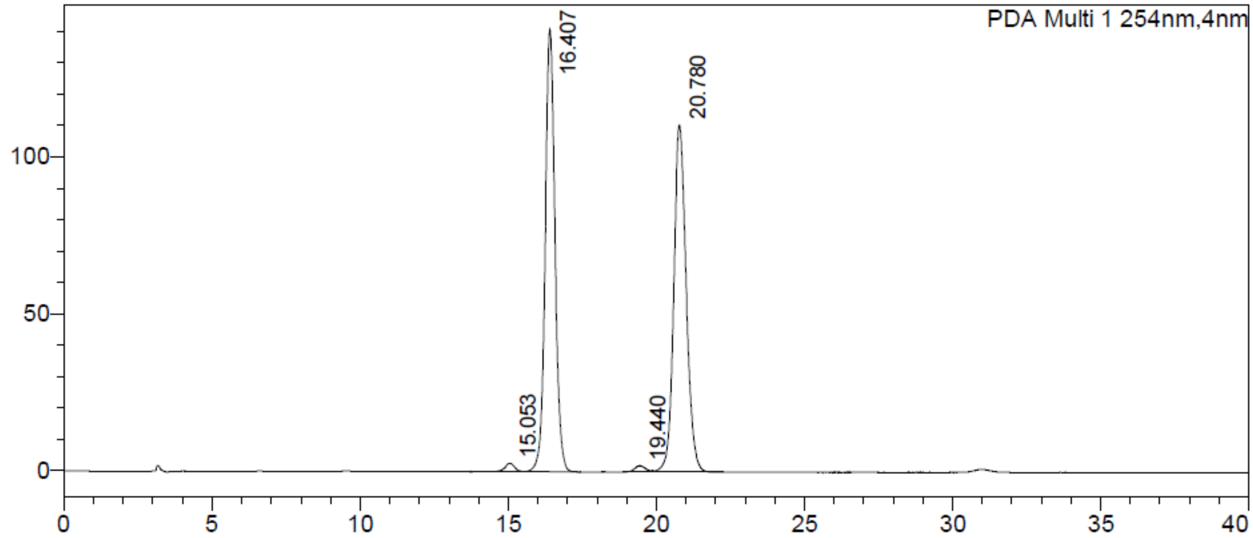

PDA Ch3 254nm

Peak#	Ret. Time	Area%
1	16.040	3.649
2	20.173	96.351
Total		100.000

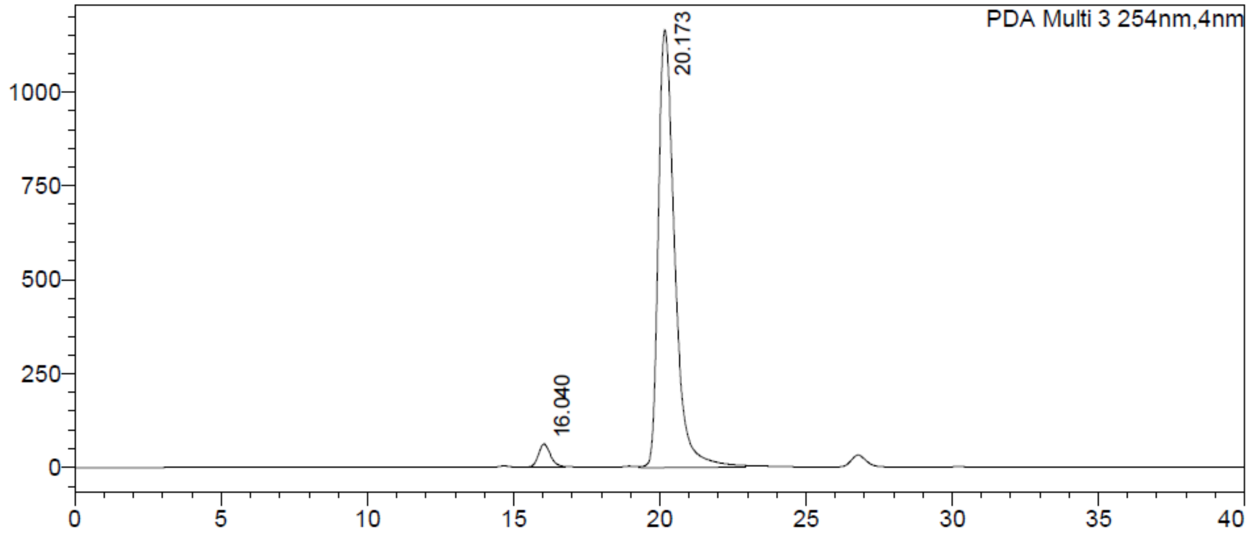

HPLC Data for **9**: Chiralpak AD-H (95:5 hexane : IPA, flow rate 1.00 mLmin^{-1}, 254 nm, 40 °C) t_R (4a*R*,10b*S*) : 17.8 min, t_R (4a*S*,10b*R*) : 14.2 min, 98:2 er.

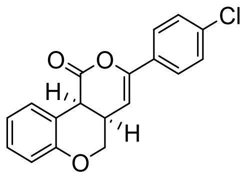

PDA Ch1 254nm

Peak#	Ret. Time	Area%
1	18.217	50.007
2	21.834	49.993
Total		100.000

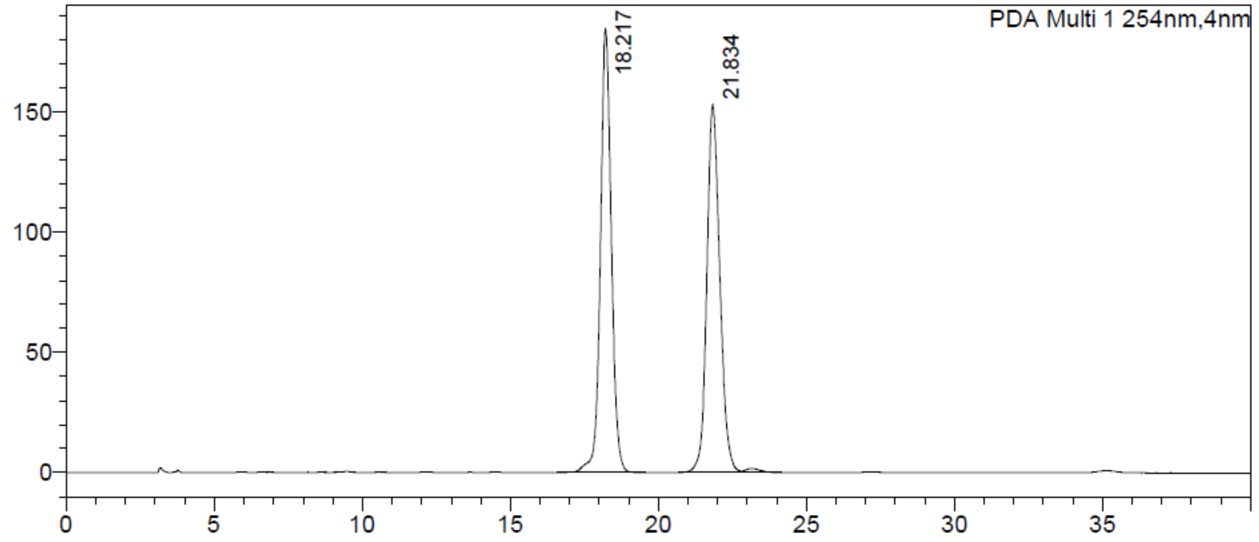

PDA Ch3 254nm

Peak#	Ret. Time	Area%
1	17.941	2.450
2	21.438	97.550
Total		100.000

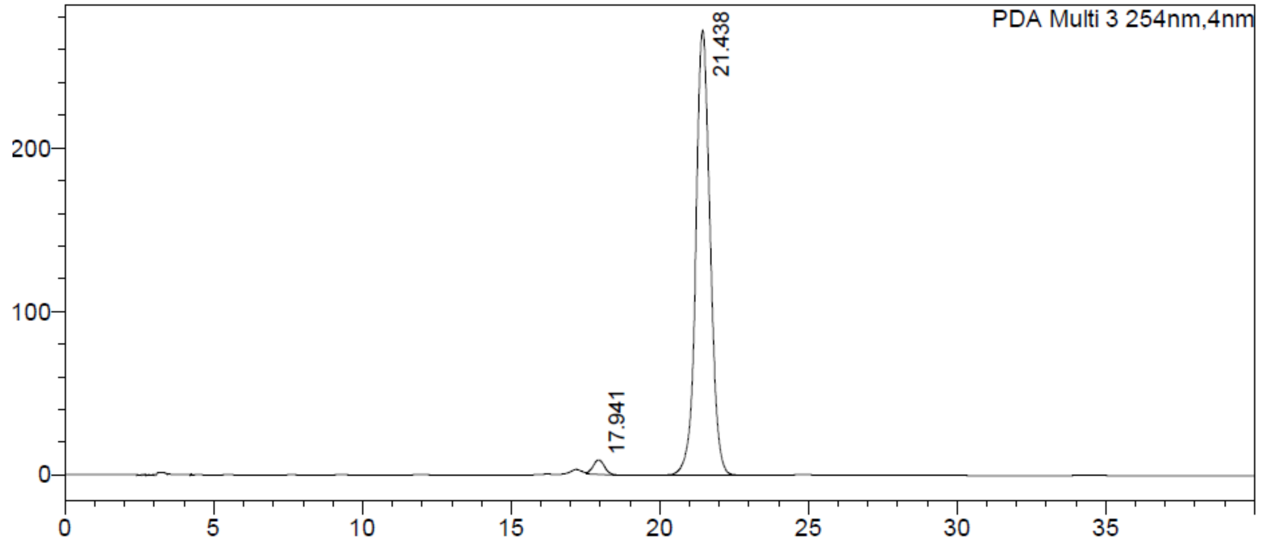

HPLC Data for **10**: Chiralpak AD-H (95:5 hexane : IPA, flow rate 1.00 mLmin^{-1}, 254 nm, 30 °C) t_R (4a*R*,10b*S*): 27.6 min, t_R (4a*S*,10b*R*) : 23.0 min, 98:2 er.

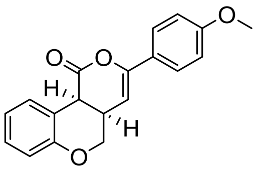

PDA Ch1 254nm

Peak#	Ret. Time	Area%
1	23.454	49.760
2	28.103	50.240
Total		100.000

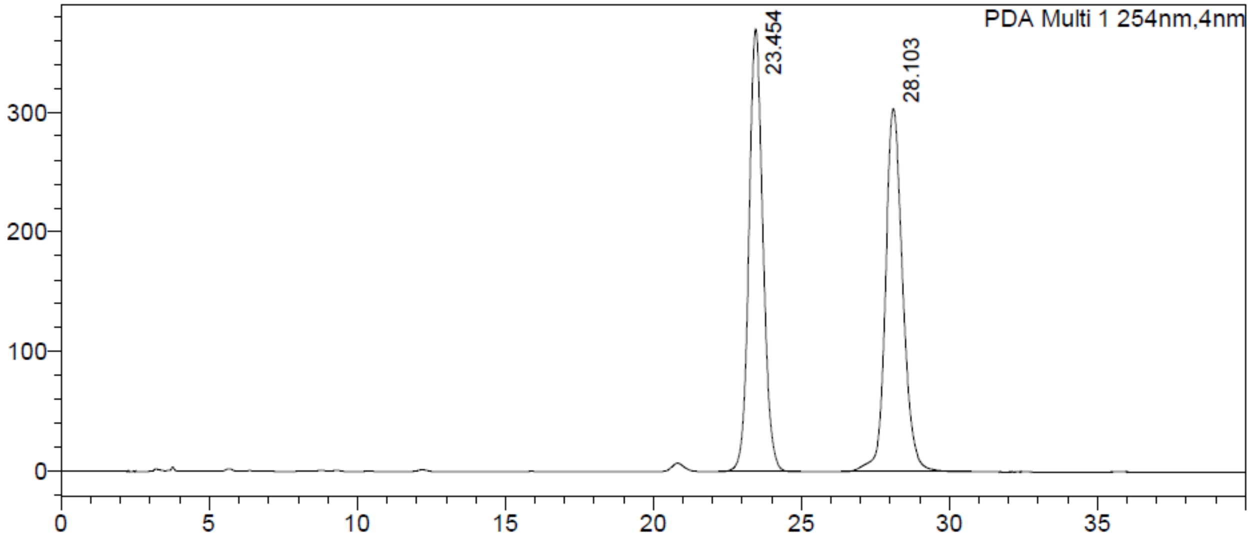

PDA Ch3 254nm

Peak#	Ret. Time	Area%
1	23.013	1.907
2	27.575	98.093
Total		100.000

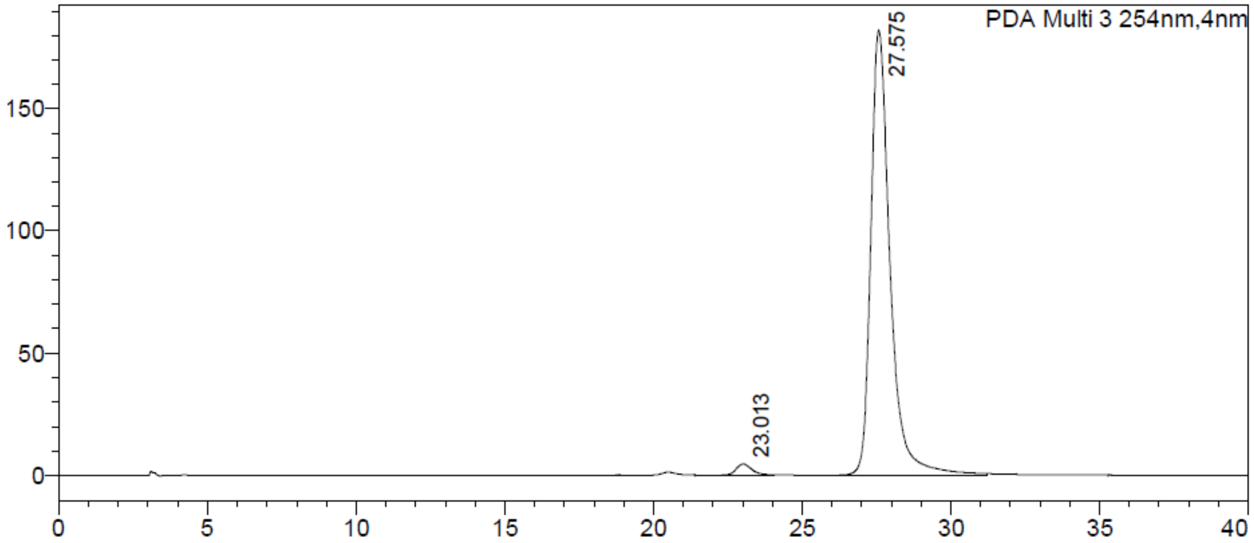

HPLC Data for **11**: Chiralpak AD-H (95:5 hexane : IPA, flow rate 1.00 mLmin^{-1}, 254 nm, 40 °C) t_R (4a*R*,10b*S*) : 17.8 min, t_R (4a*S*,10b*R*) : 14.2 min, 98:2 er.

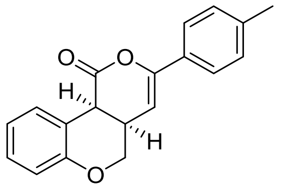

PDA Ch1 254nm

Peak#	Ret. Time	Area%
1	14.450	49.939
2	18.178	50.061
Total		100.000

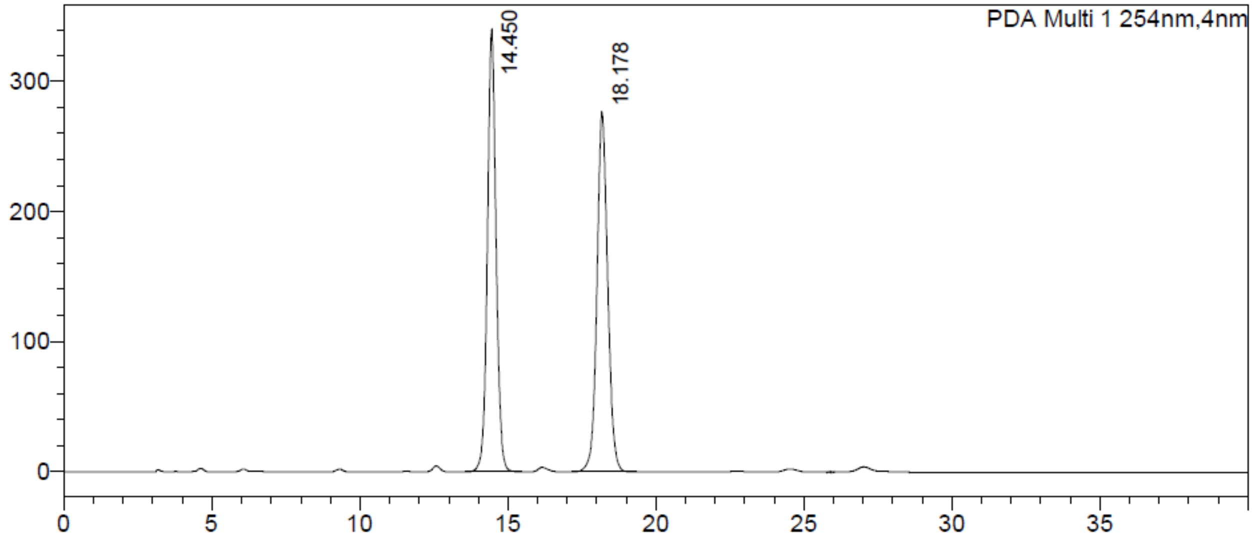

PDA Ch3 254nm

Peak#	Ret. Time	Area%
1	14.205	2.265
2	17.807	97.735
Total		100.000

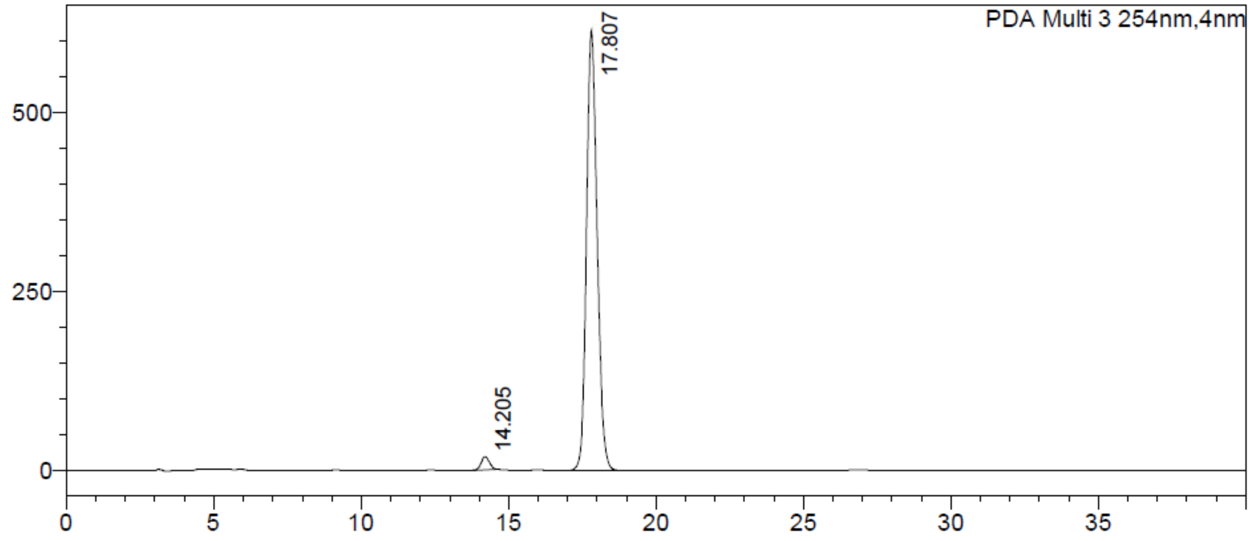

HPLC Data for **12**: Chiralpak AD-H (95:5 hexane : IPA, flow rate 1.00 mLmin^{-1}, 254 nm, 40 °C) t_R (4a*R*,10b*S*) : 17.8 min, t_R (4a*S*,10b*R*) : 15.6 min, 96:4 er.

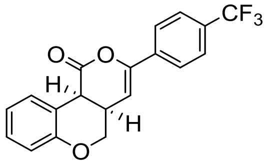

PDA Ch1 254nm

Peak#	Ret. Time	Area%
1	15.972	50.479
2	18.257	49.521
Total		100.000

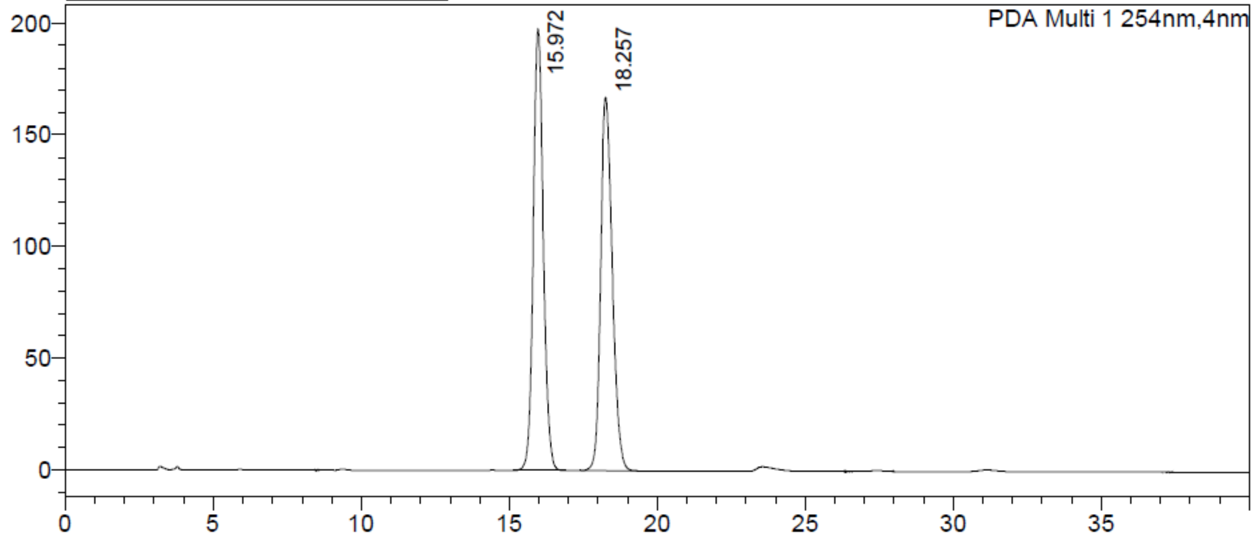

PDA Ch3 254nm

Peak#	Ret. Time	Area%
1	15.597	4.136
2	17.762	95.864
Total		100.000

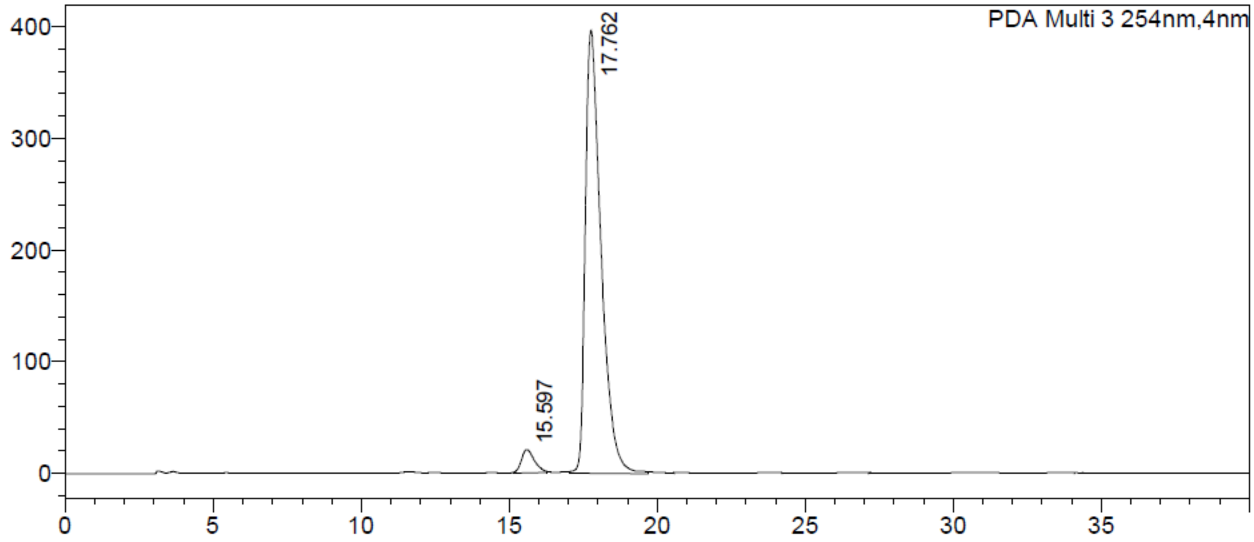

HPLC Data for **13**: Chiralpak OD-H (97:3 hexane : IPA, flow rate 1.00 mLmin^{-1}, 220 nm, 40 °C) t_R (4a*R*,10b*S*) : 19.9 min, t_R (4a*S*,10b*R*) : 17.4 min, 91:9 er.

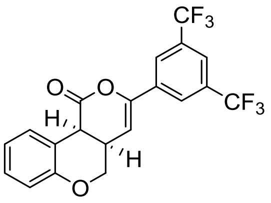

PDA Ch2 220nm

Peak#	Ret. Time	Area%
1	17.409	52.313
2	20.001	47.687
Total		100.000

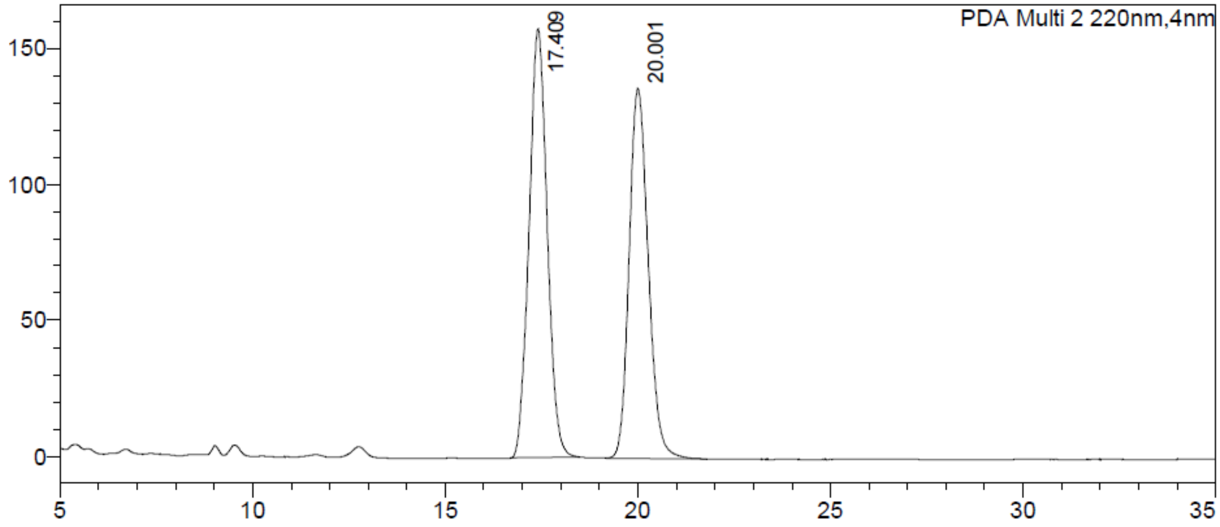

PDA Ch2 220nm

Peak#	Ret. Time	Area%
1	17.417	9.204
2	19.912	90.796
Total		100.000

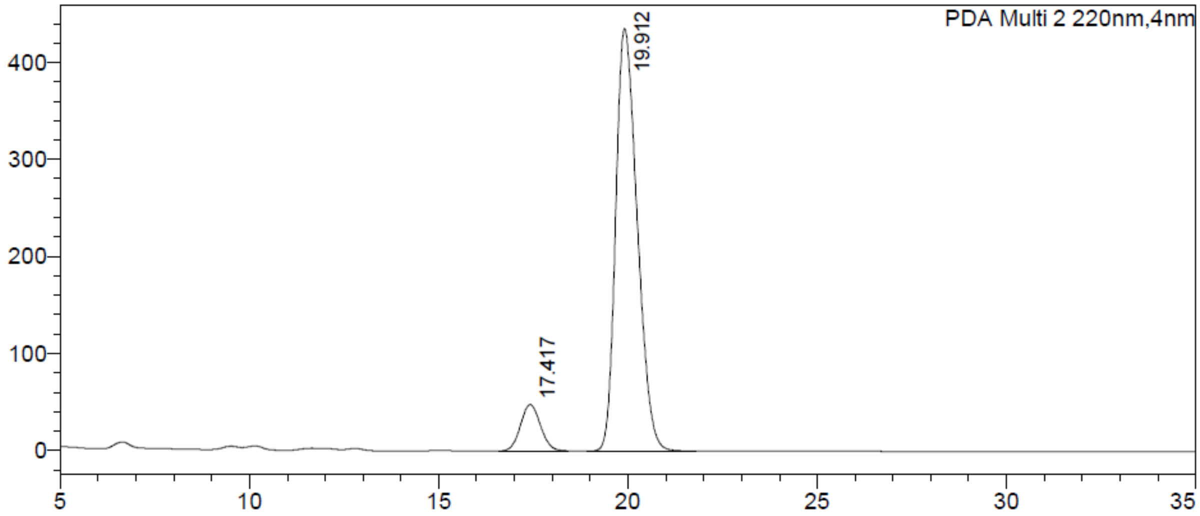

HPLC Data for **14**: Chiralpak AD-H (95:5 hexane : IPA, flow rate 1.00 mLmin^{-1}, 254 nm, 40 °C) t_R (4a*R*,10b*S*) : 9.8 min, t_R (4a*S*,10b*R*) : 8.7 min, 71:29 er.

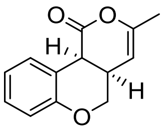

PDA Ch1 254nm

Peak#	Ret. Time	Area%
1	8.792	50.389
2	9.871	49.611
Total		100.000

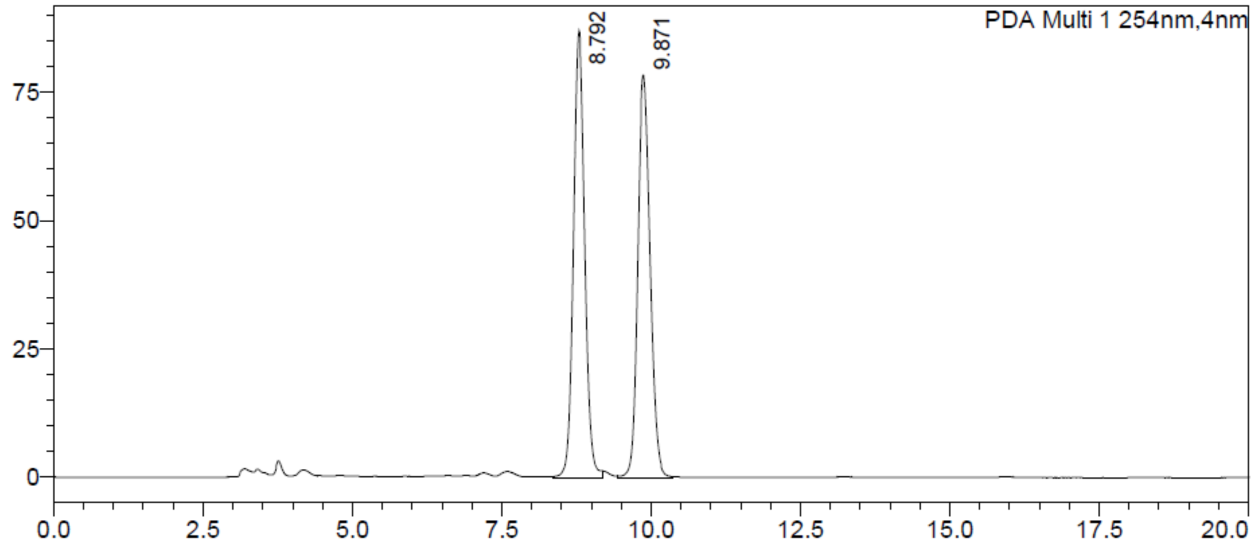

PDA Ch3 254nm

Peak#	Ret. Time	Area%
1	8.694	29.046
2	9.767	70.954
Total		100.000

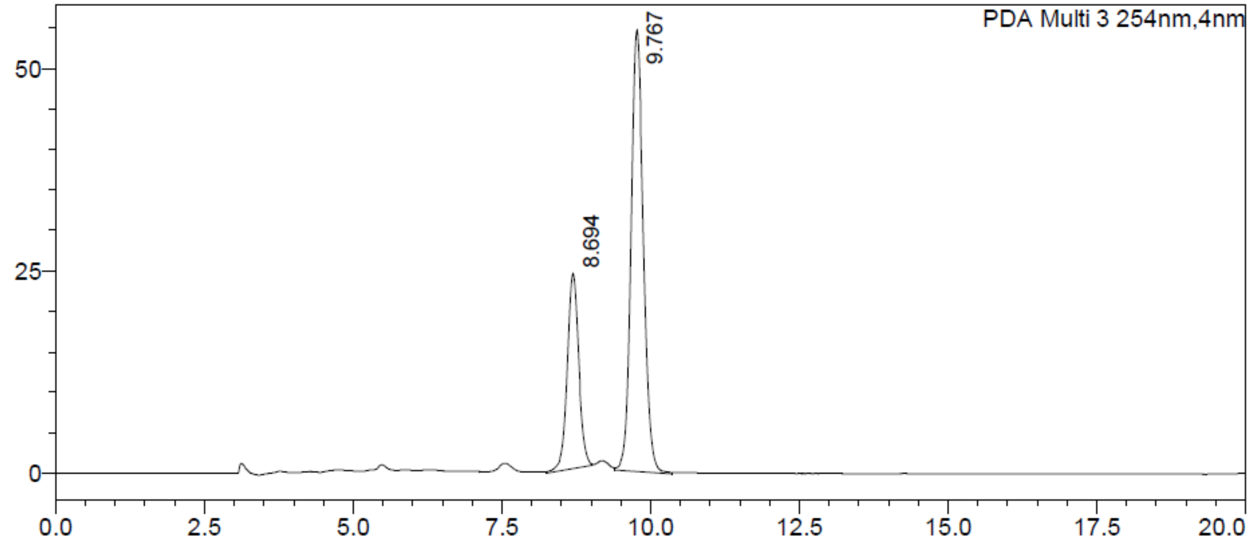

HPLC Data for **15**: Chiralpak AD-H (95:5 hexane : IPA, flow rate 1.00 mLmin^{-1}, 254 nm, 40 °C) t_R (4a*R*,10b*S*) : 26.8 min, t_R (4a*S*,10b*R*) : 22.9 min, 97:3 er.

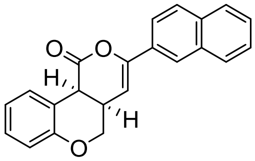

PDA Ch1 254nm

Peak#	Ret. Time	Area%
1	23.246	49.489
2	27.127	50.511
Total		100.000

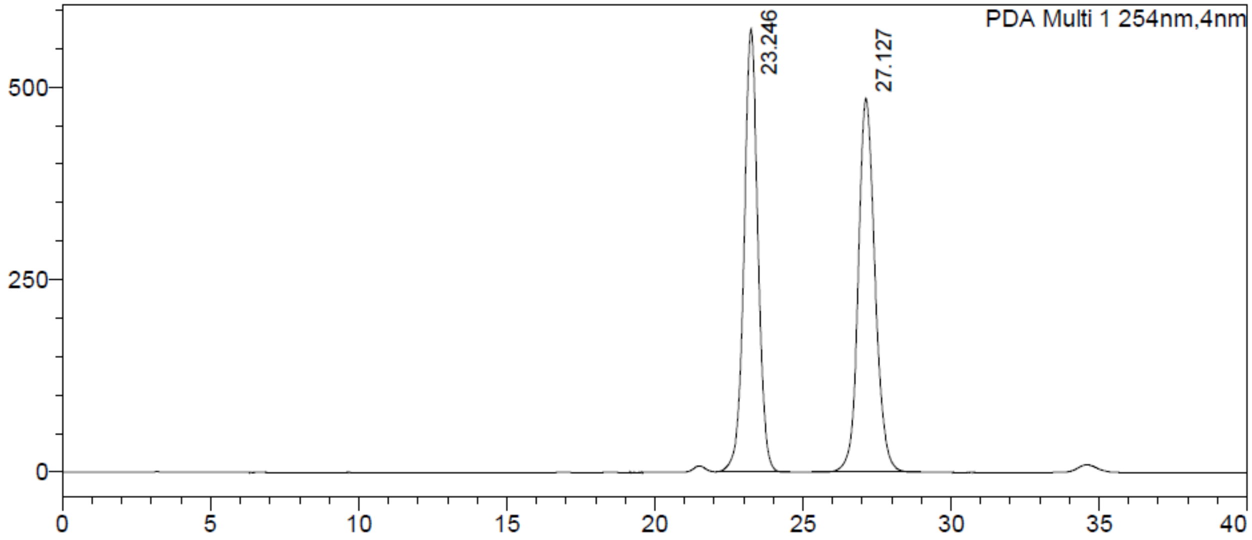

PDA Ch3 254nm

Peak#	Ret. Time	Area%
1	22.866	2.849
2	26.755	97.151
Total		100.000

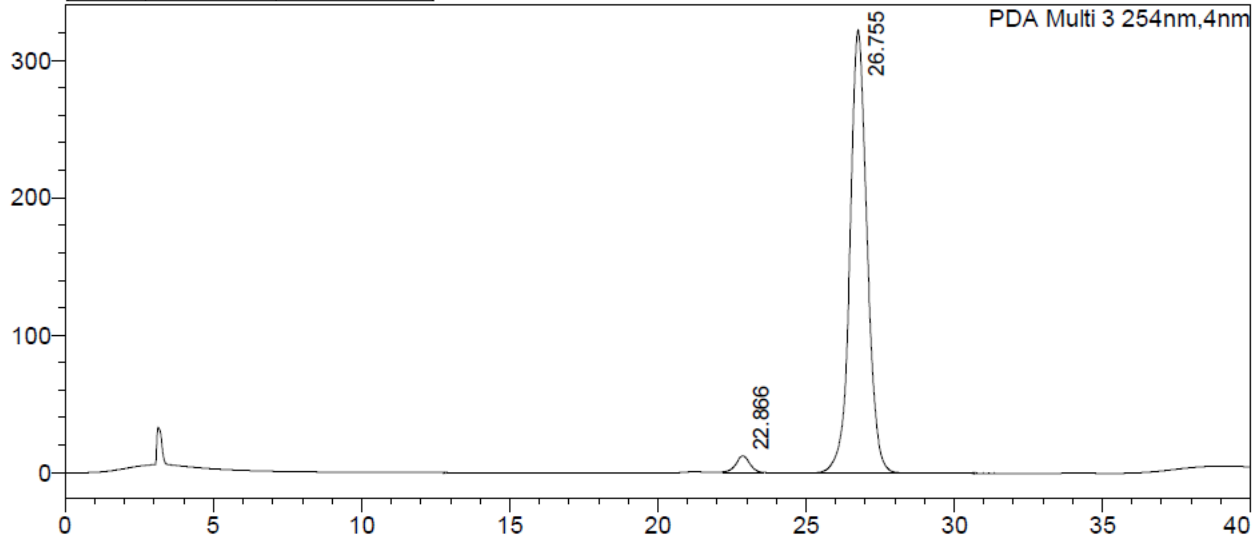

HPLC Data for **16**: Chiralpak AD-H (95:5 hexane : IPA, flow rate 1.00 mLmin^{-1}, 254 nm, 40 °C) t_R (4a*S*,10b*R*) : 12.9 min, t_R (4a*R*,10b*S*) : 15.2 min, 98:2 er.

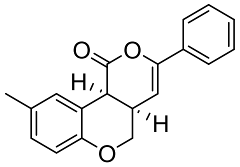

PDA Ch3 254nm

Peak#	Ret. Time	Area%
1	12.851	50.166
2	15.220	49.834
Total		100.000

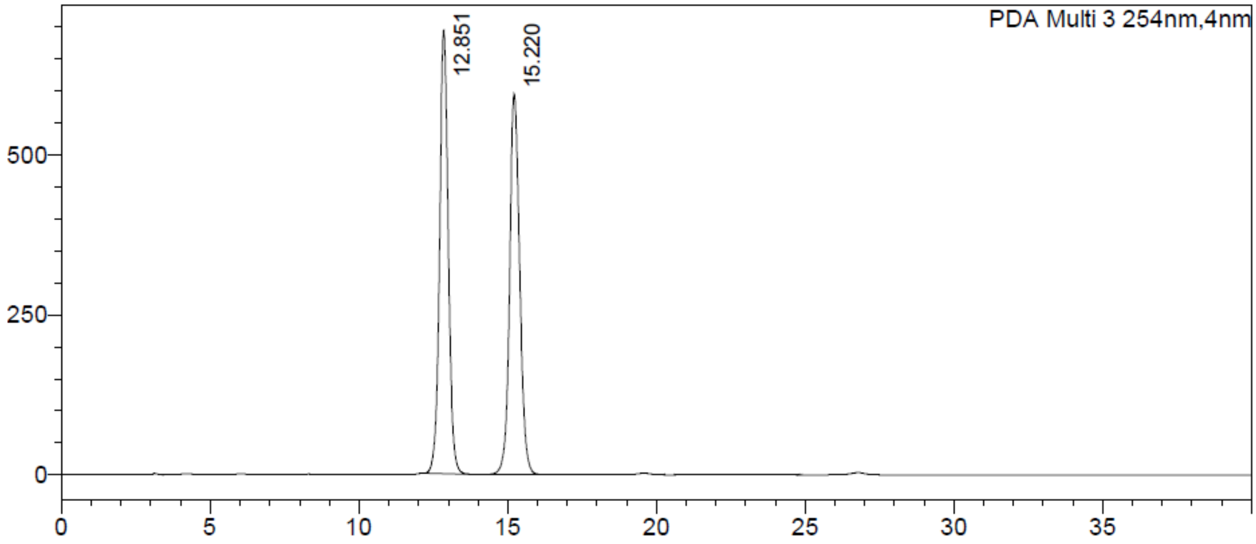

PDA Ch3 254nm

Peak#	Ret. Time	Area%
1	12.864	1.522
2	15.214	98.478
Total		100.000

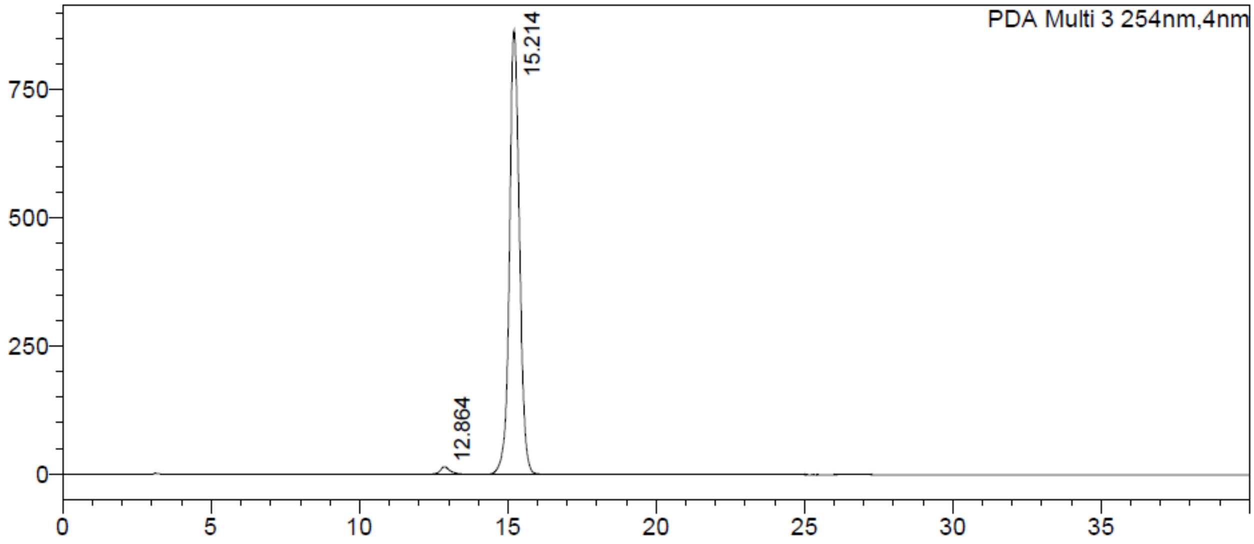

HPLC Data for **17**: Chiralpak AD-H (95:5 hexane : IPA, flow rate 1.00 mLmin^{-1}, 254 nm, 40 °C) t_R (4a*S*,10b*R*) : 19.7 min, t_R (4a*R*,10b*S*) : 29.7 min, 98:2 er.

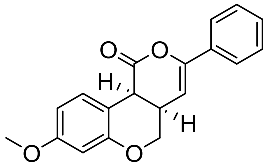

PDA Ch3 254nm

Peak#	Ret. Time	Area%
1	19.684	49.952
2	29.682	50.048
Total		100.000

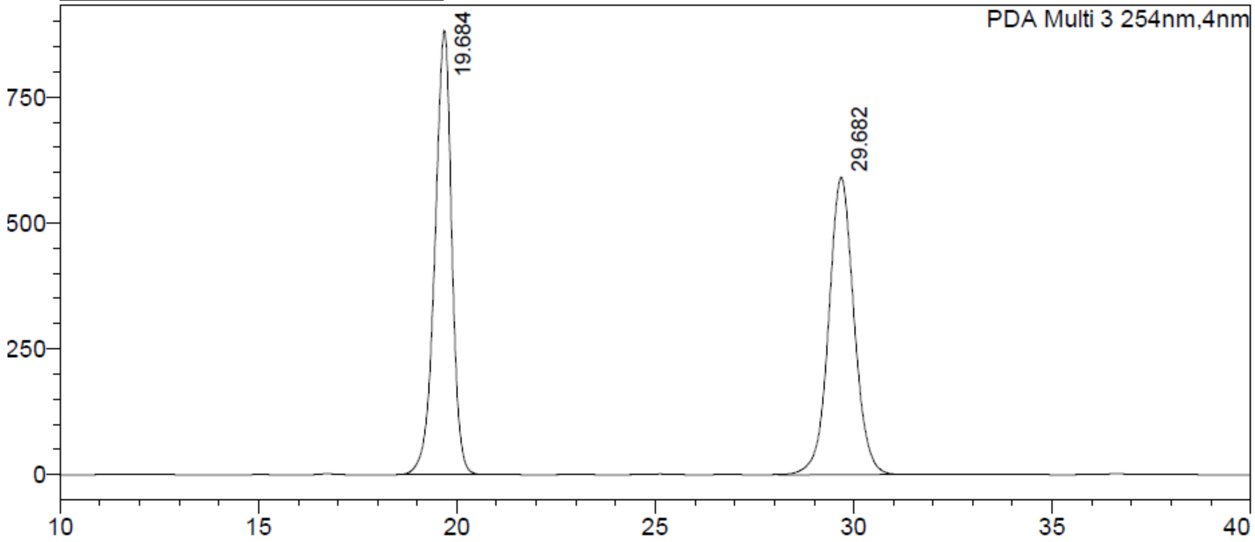

PDA Ch3 254nm

Peak#	Ret. Time	Area%
1	19.651	2.026
2	29.709	97.974
Total		100.000

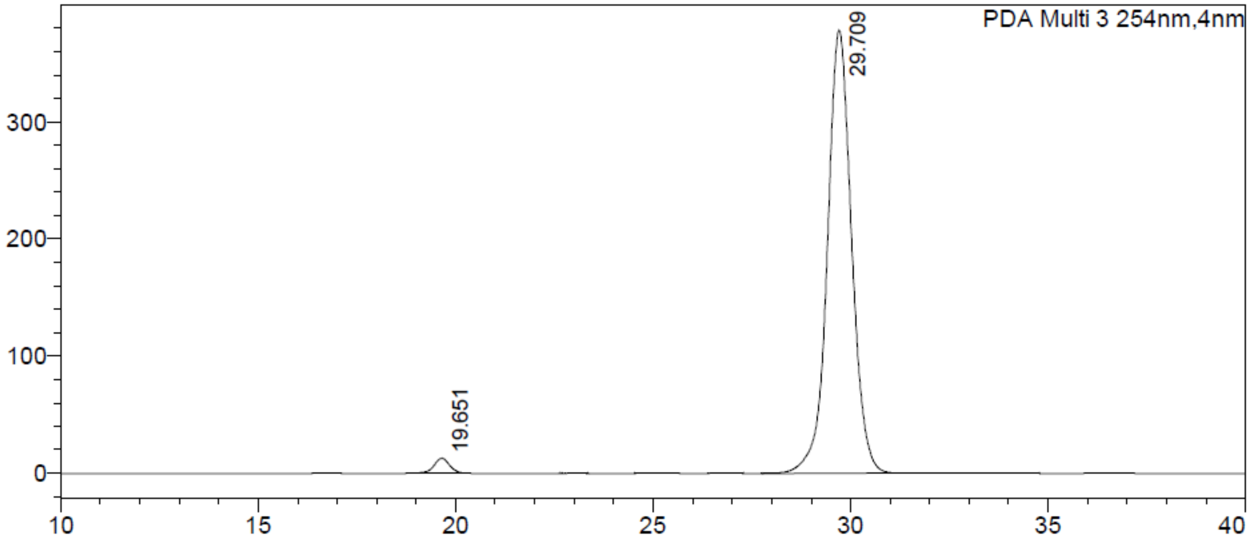

HPLC Data for **18**: Chiralpak AD-H (95:5 hexane : IPA, flow rate 1.00 mLmin^{-1}, 254 nm, 40 °C) t_R (4a*R*,10b*S*) : 21.1 min, t_R (4a*S*,10b*R*) : 13.0 min, 96:4 er.

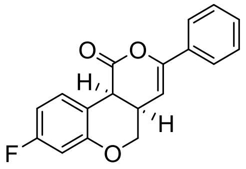

PDA Ch3 254nm

Peak#	Ret. Time	Area%
1	13.049	49.270
2	21.231	50.730
Total		100.000

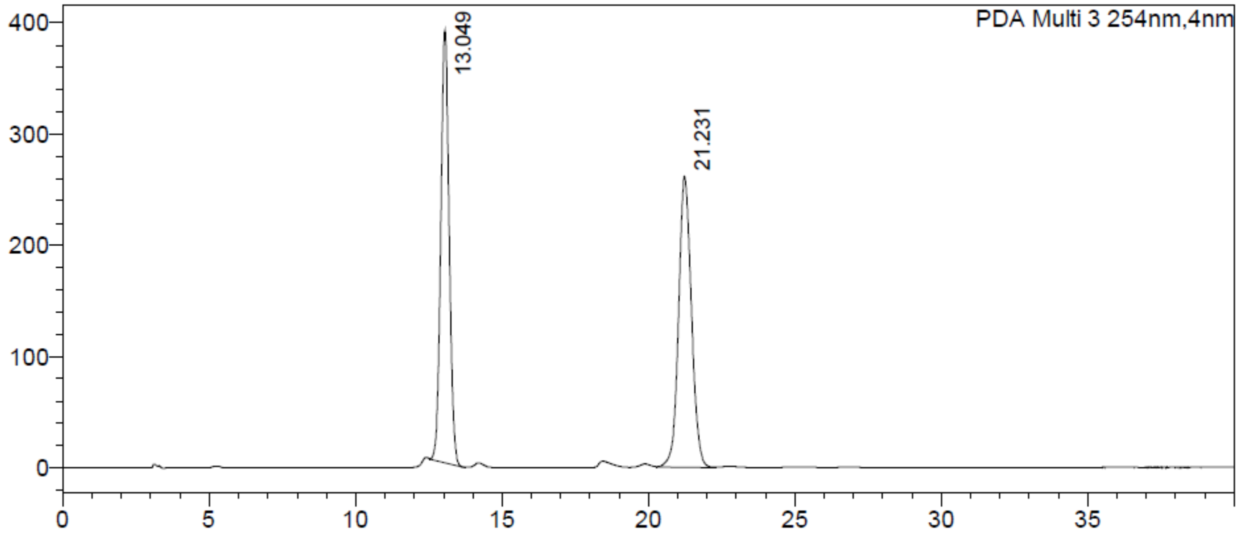

PDA Ch3 254nm

Peak#	Ret. Time	Area%
1	13.014	4.369
2	21.129	95.631
Total		100.000

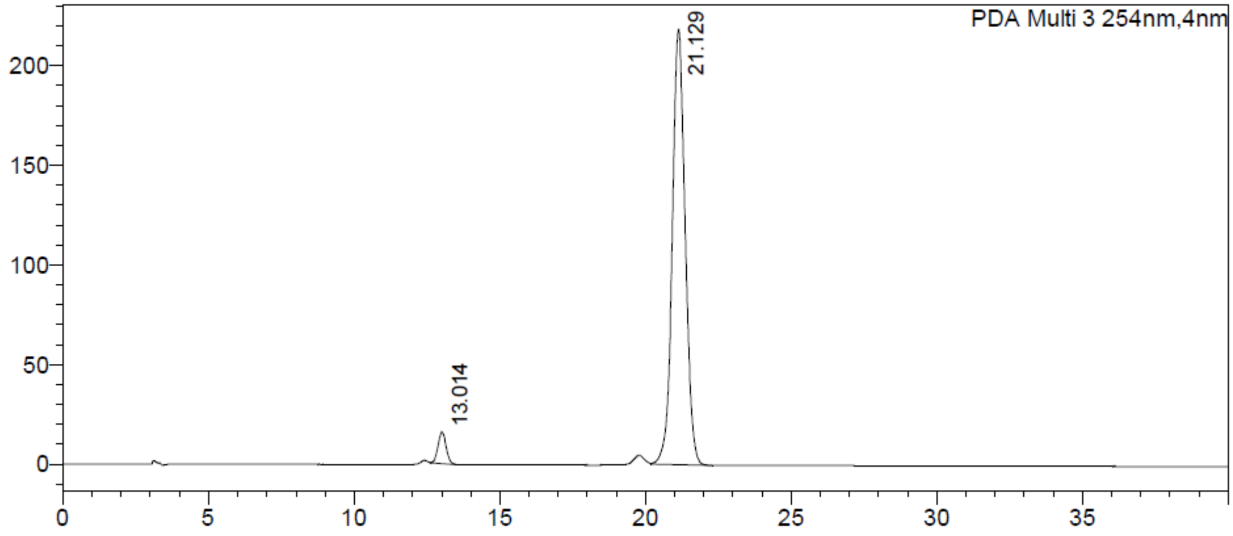

HPLC Data for **19**: Chiralpak AD-H (95:5 hexane : IPA, flow rate 1.00 mLmin^{-1}, 254 nm, 40 °C) t_R (4a*S*,12a*R*) : 28.8 min, t_R (4a*R*,12a*S*) : 15.0 min, 97:3 er.

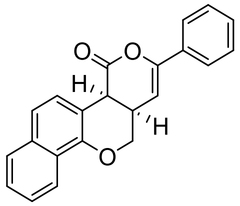

PDA Ch3 254nm

Peak#	Ret. Time	Area%
1	15.057	50.276
2	29.168	49.724
Total		100.000

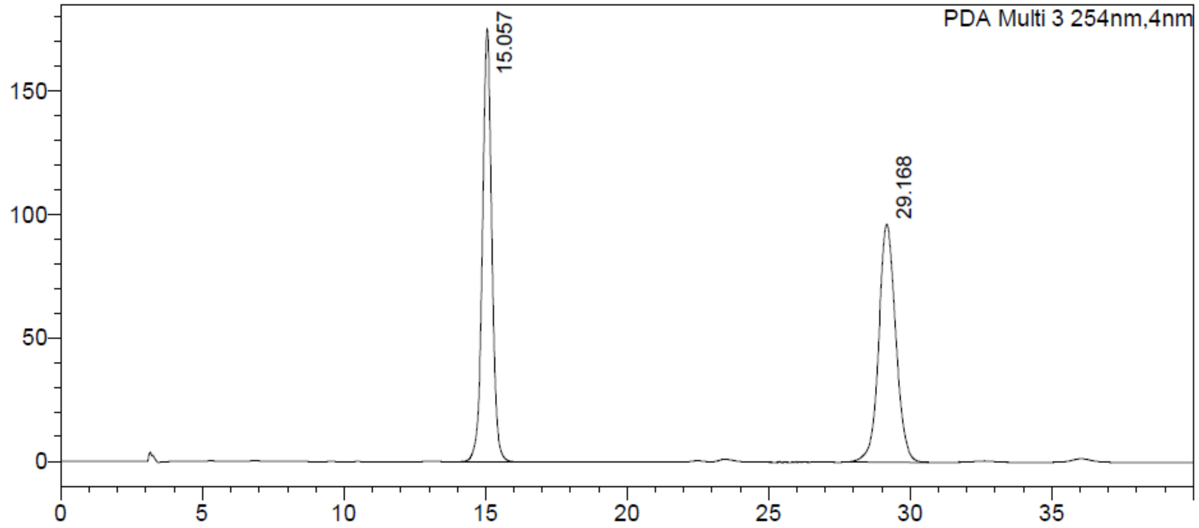

PDA Ch3 254nm

Peak#	Ret. Time	Area%
1	14.969	3.058
2	28.788	96.942
Total		100.000

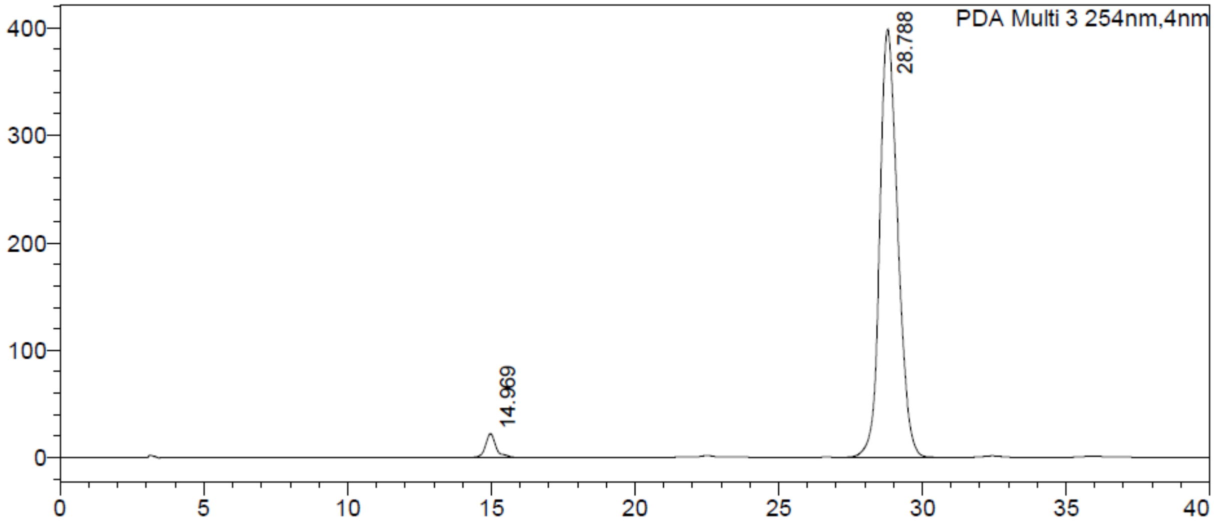

HPLC Data for **20**: Chiralpak OD-H (95:5 hexane : IPA, flow rate 1.00 mLmin^{-1}, 254 nm, 40 °C) t_R (4a*R*,10b*S*): 28.2 min, t_R (4a*S*,10b*R*) : 22.6 min, 97:3 er.

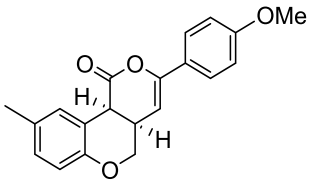

PDA Ch3 254nm

Peak#	Ret. Time	Area%
1	22.321	50.250
2	27.512	49.750
Total		100.000

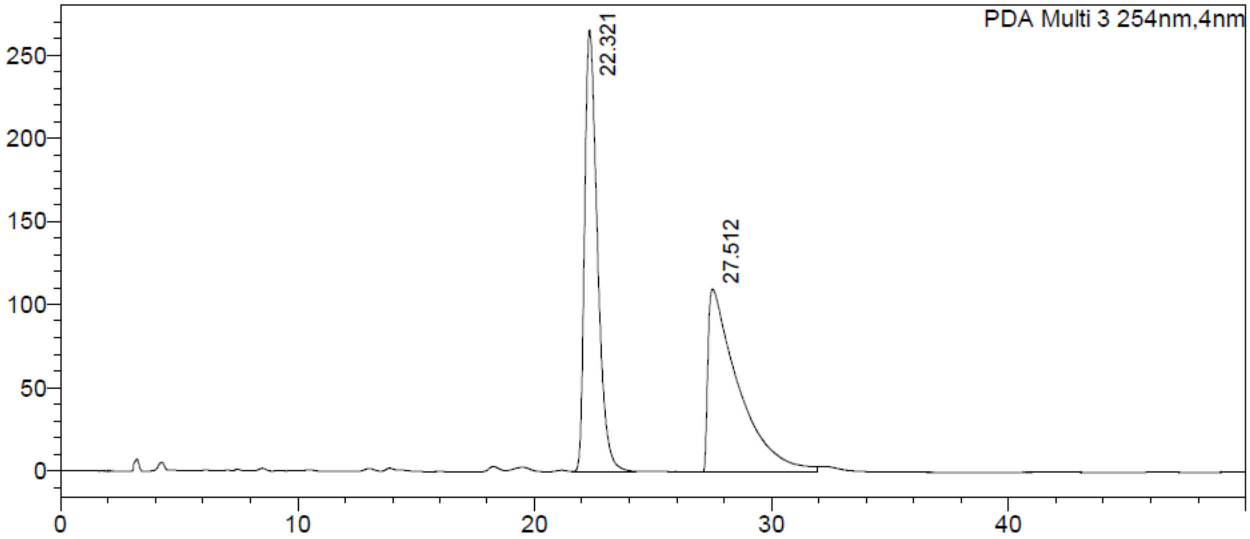

PDA Ch3 254nm

Peak#	Ret. Time	Area%
1	22.551	2.837
2	28.201	97.163
Total		100.000

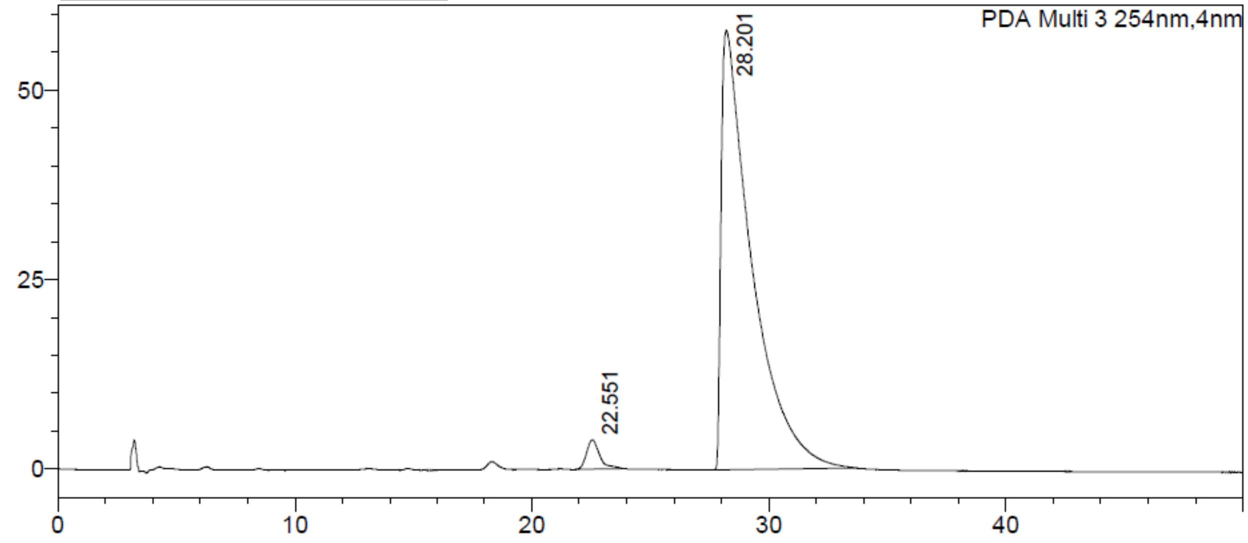

HPLC Data for **21**: Chiralpak OD-H (95:5 hexane : IPA, flow rate 1.00 mLmin^{-1}, 220 nm, 40 °C) t_R (4a*R*,10b*S*) : 45.5 min, t_R (4a*S*,10b*R*) : 39.8 min, 97:3 er.

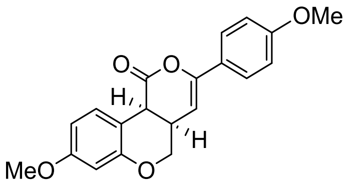

PDA Ch2 220nm

Peak#	Ret. Time	Area%
1	38.724	50.173
2	45.246	49.827
Total		100.000

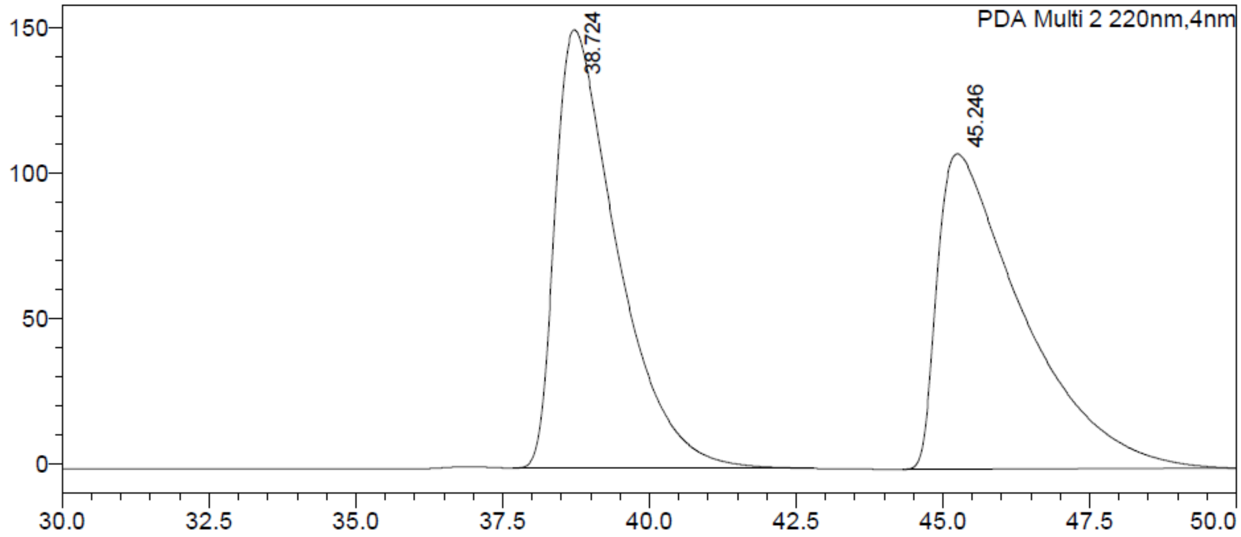

PDA Ch2 220nm

Peak#	Ret. Time	Area%
1	39.809	2.873
2	45.513	97.127
Total		100.000

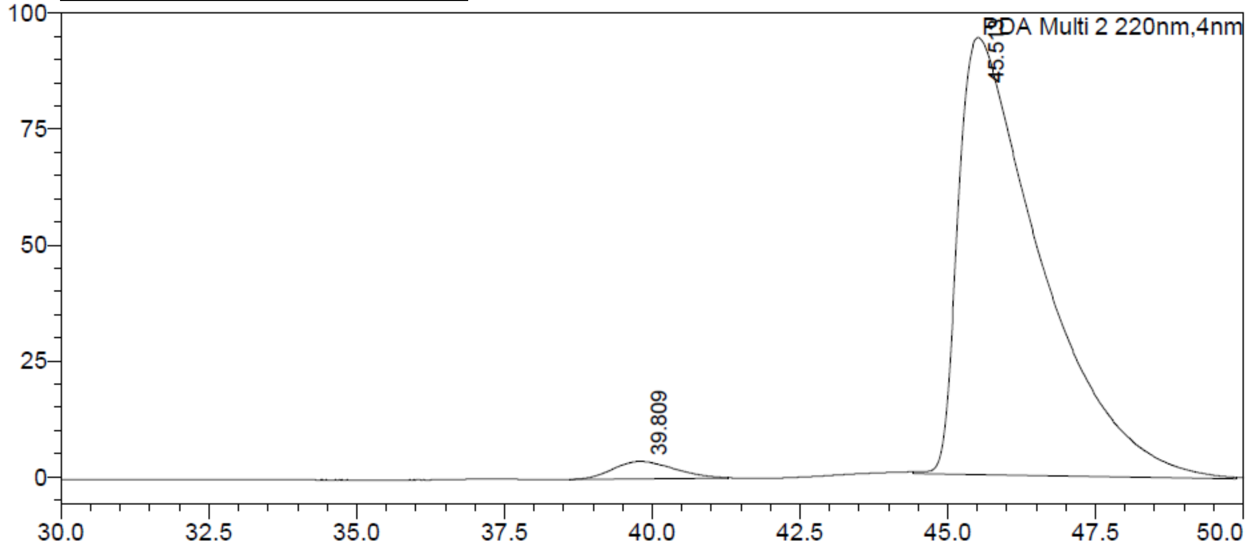

HPLC Data for **22**: Chiralpak AD-H (95:5 hexane : IPA, flow rate 1.50 mLmin^{-1}, 270 nm, 40 °C) t_R (4a*S*,12a*R*) : 38.4 min, t_R (4a*R*,12a*S*) : 23.7 min, 97:3 er.

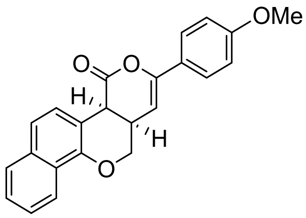

PDA Ch4 270nm

Peak#	Ret. Time	Area%
1	23.775	50.063
2	38.658	49.937
Total		100.000

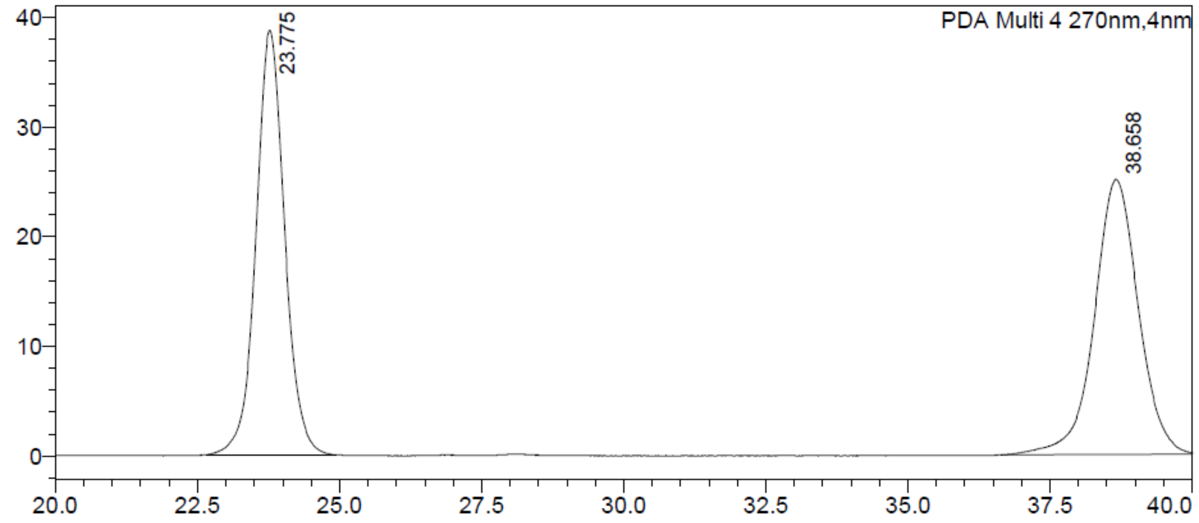

PDA Ch4 270nm

Peak#	Ret. Time	Area%
1	23.705	3.183
2	38.404	96.817
Total		100.000

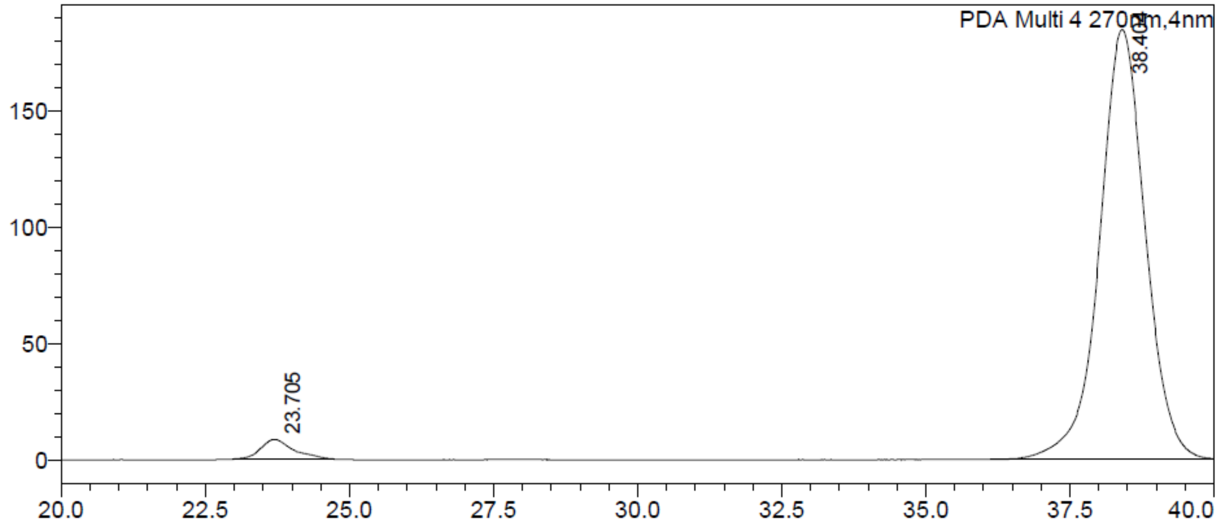

HPLC Data for **23**: Chiralpak AD-H (95:5 hexane : IPA, flow rate 1.00 mLmin^{-1}, 254 nm, 40 °C) t_R (3*R*,4*S*): 13.5 min, t_R (3*S*,4*R*): 14.9 min, 98:2 er.

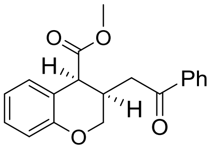

PDA Ch3 254nm

Peak#	Ret. Time	Area%
1	13.481	49.859
2	14.894	50.141
Total		100.000

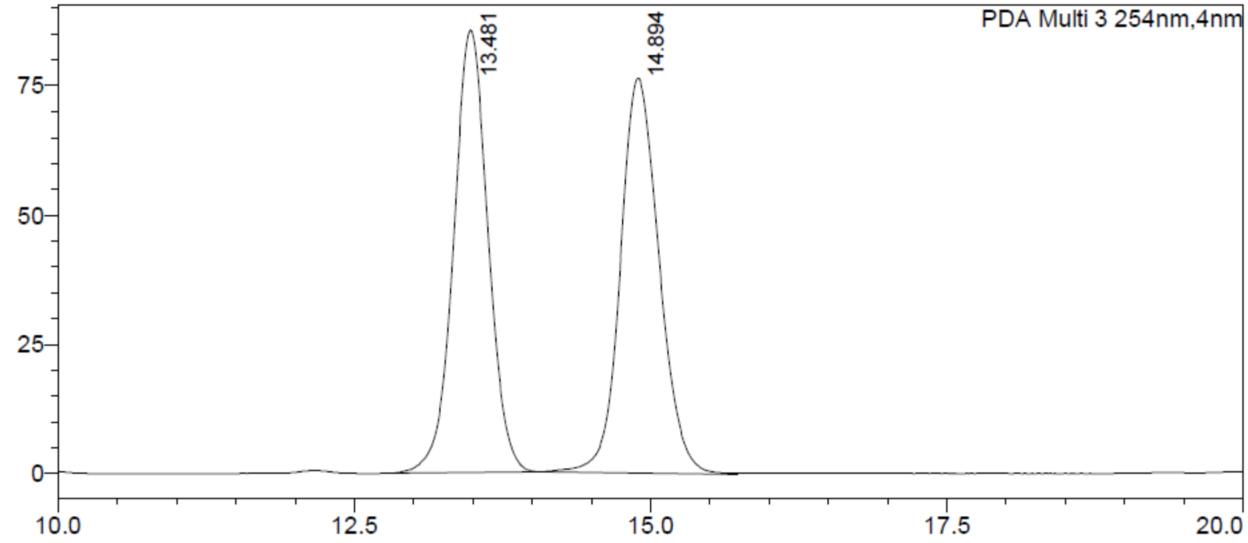

PDA Ch3 254nm

Peak#	Ret. Time	Area%
1	13.482	97.933
2	14.947	2.067
Total		100.000

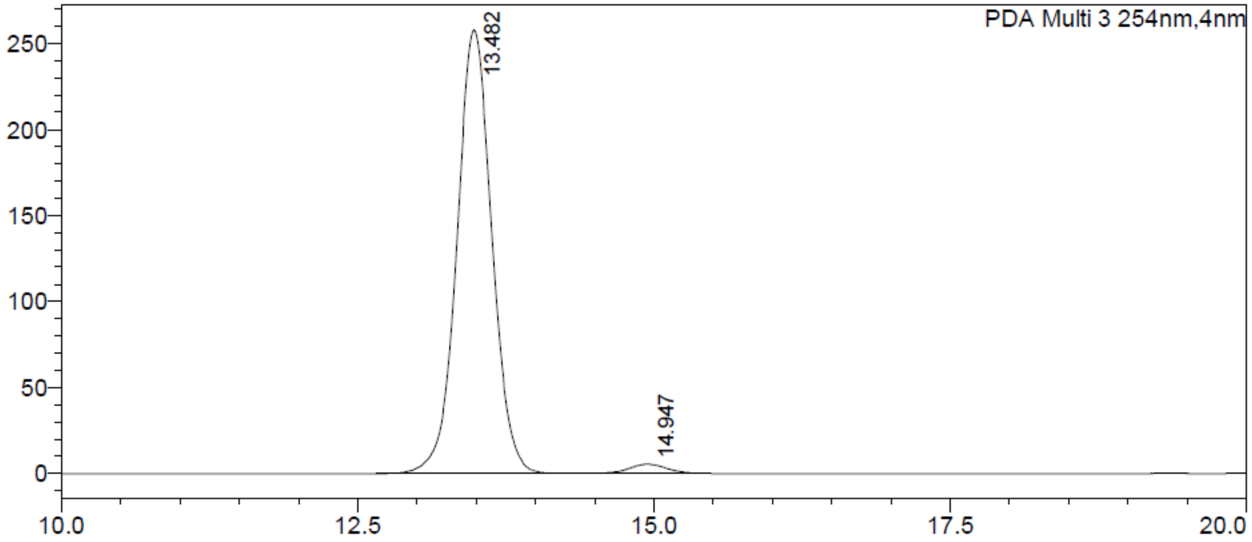

HPLC Data for **24**: Chiralpak IB (93:7 hexane : IPA, flow rate 1.00 mLmin^{-1}, 220 nm, 40 °C) t_R (3*R*,4*S*) : 35.8 min,t_R (3*S*,4*R*) : 22.6 min, 98:2 er.

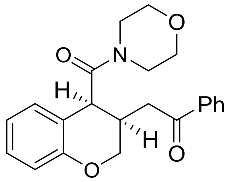

PDA Ch3 254nm

Peak#	Ret. Time	Area%
1	21.833	46.344
2	35.602	53.656
Total		100.000

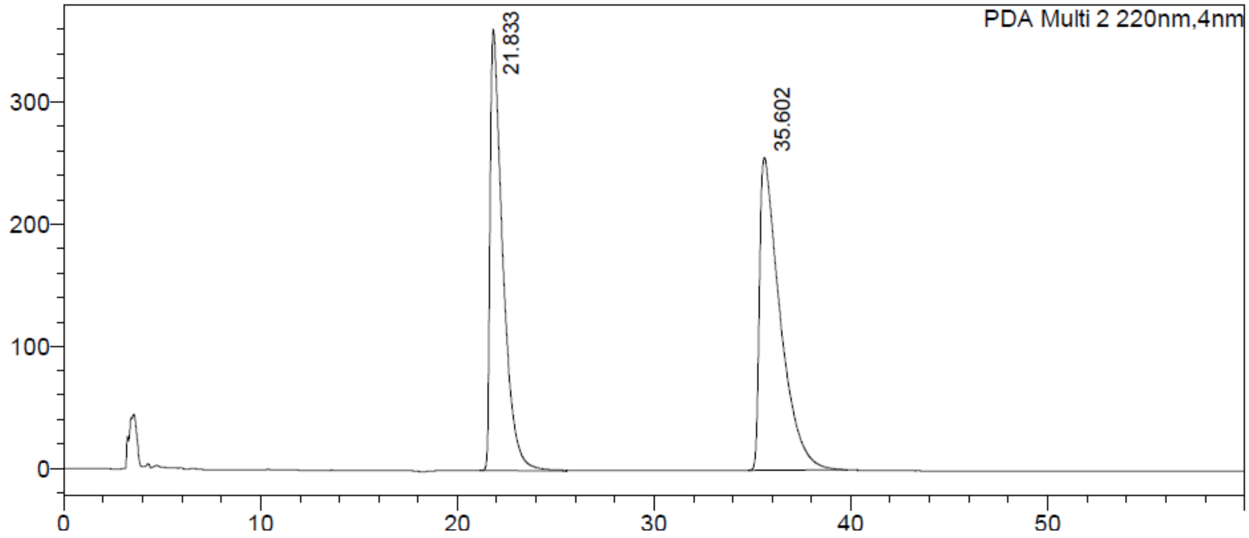

PDA Ch2 220nm

Peak#	Ret. Time	Area%
1	22.571	1.923
2	35.822	98.077
Total		100.000

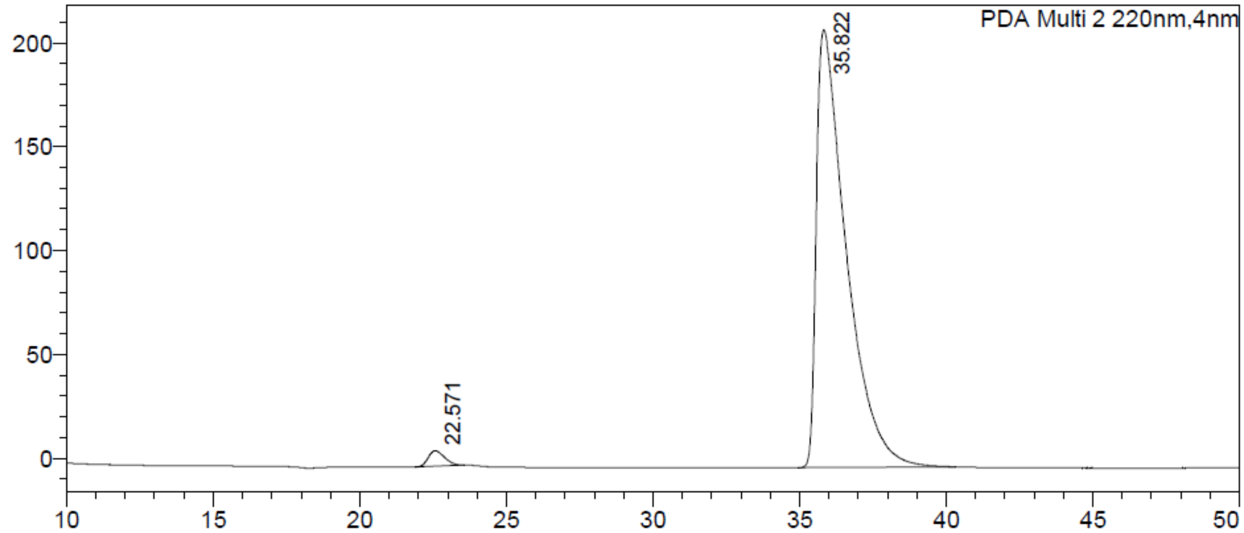

HPLC Data for **25**: Chiralpak AD-H (90:10 hexane : IPA, flow rate 1.00 mLmin^{-1}, 254 nm, 40 °C) t_R (3*R*,4*S*) : 28.3 min, t_R (3*S*,4*R*) : 32.5 min, 98:2 er.

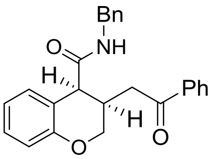

PDA Ch3 254nm

Peak#	Ret. Time	Area%
1	28.297	49.580
2	31.301	50.420
Total		100.000

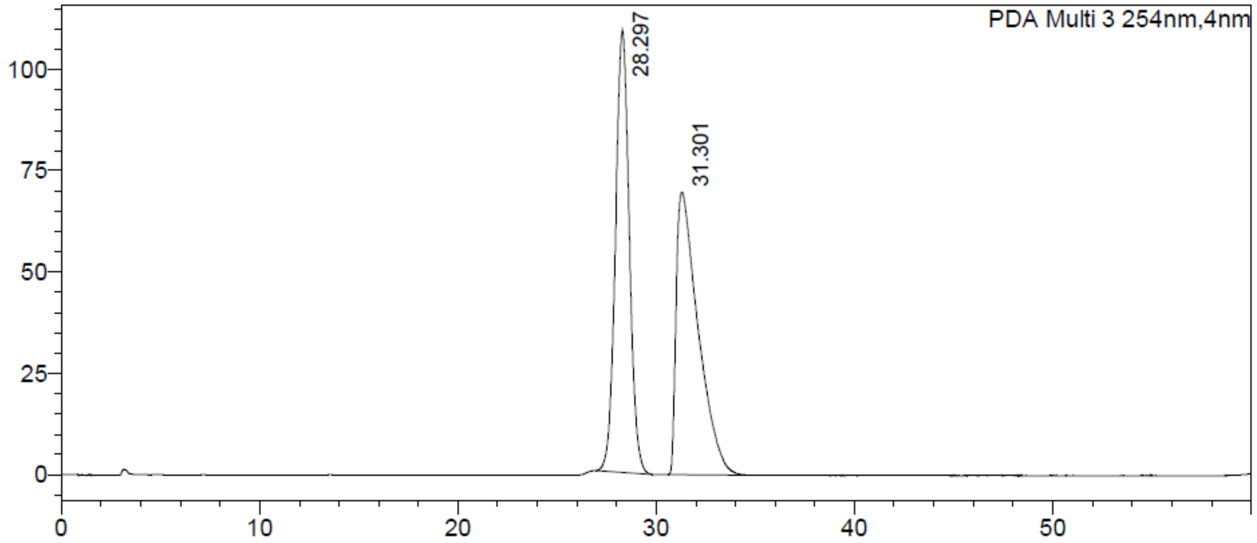

PDA Ch3 254nm

Peak#	Ret. Time	Area%
1	28.343	97.761
2	32.471	2.239
Total		100.000

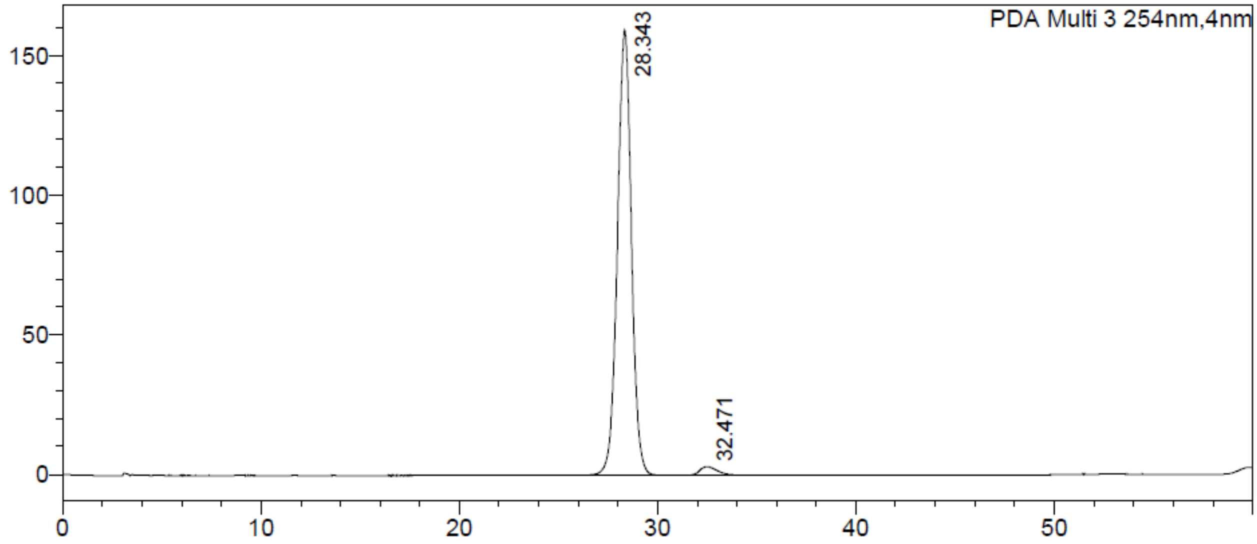

HPLC Data for **26**: Chiralpak AD-H (97:3 hexane : IPA, flow rate 1.00 mLmin^{-1}, 211 nm, 30 °C) t_R (3*R*,4*S*) : 25.3 min, t_R (3*S*,4*R*) : 20.6 min, 98:2 er.

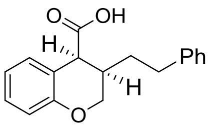

Detector A Channel 1 211nm

Peak#	Ret. Time	Area%
1	20.405	50.023
2	25.035	49.977
Total		100.000

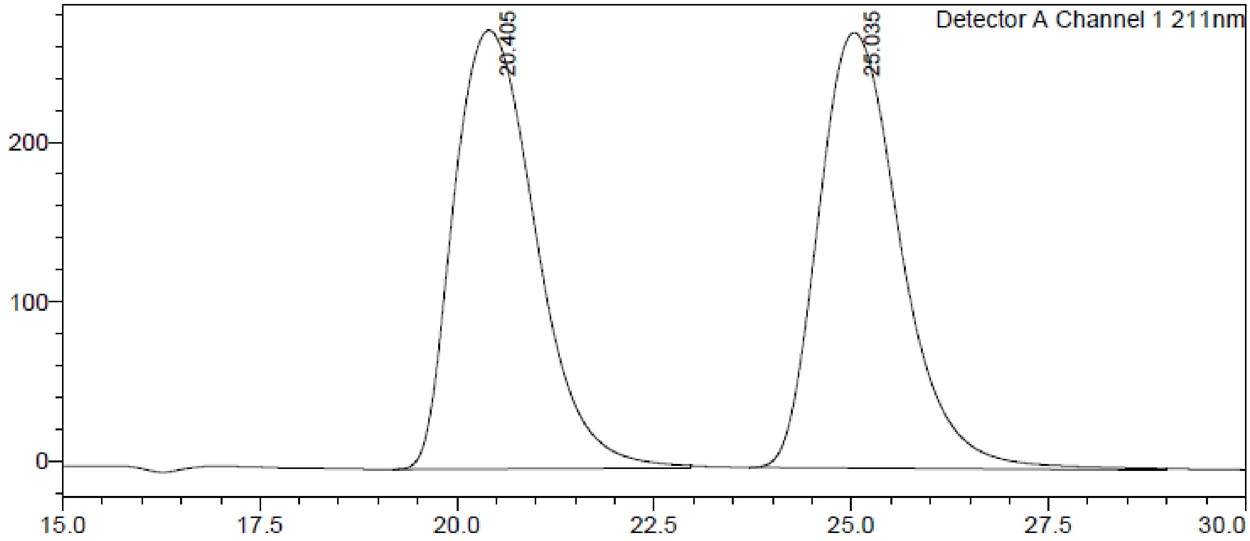

Detector A Channel 1 211nm

Peak#	Ret. Time	Area%
1	20.581	1.805
2	25.321	98.195
Total		100.000

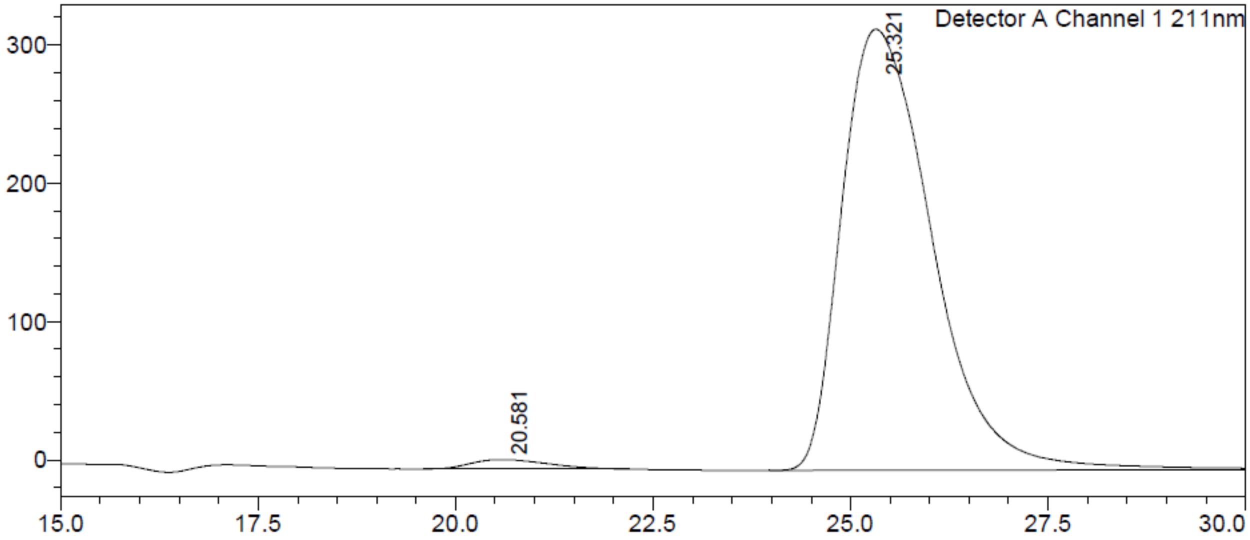

HPLC Data for **27**: Chiralpak AD-H (90:10 hexane : IPA, flow rate 1.00 mLmin^{-1}, 254 nm, 40 °C) t_R (3*R*,3a*R*,9b*S*) : 38.2 min, t_R (3*S*,3a*S*,9b*R*) : 53.4 min, 100:0 er.

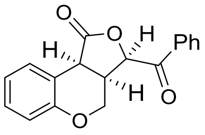

PDA Ch3 254nm

Peak#	Ret. Time	Area%
1	38.660	49.899
2	53.359	50.101
Total		100.000

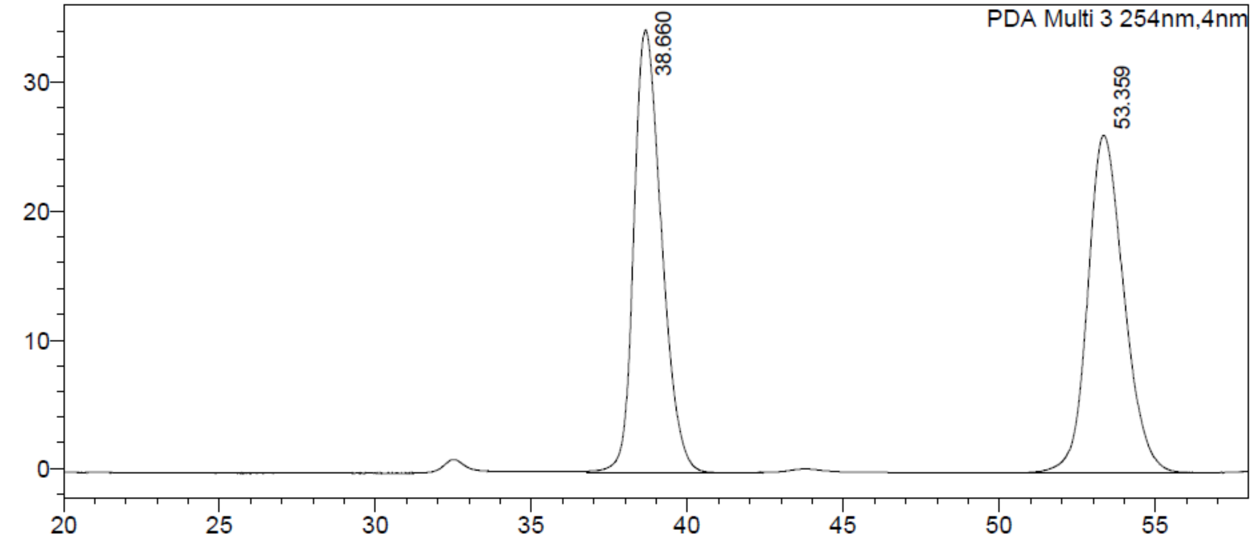

PDA Ch3 254nm

Peak#	Ret. Time	Area%
1	38.173	100.000
Total		100.000

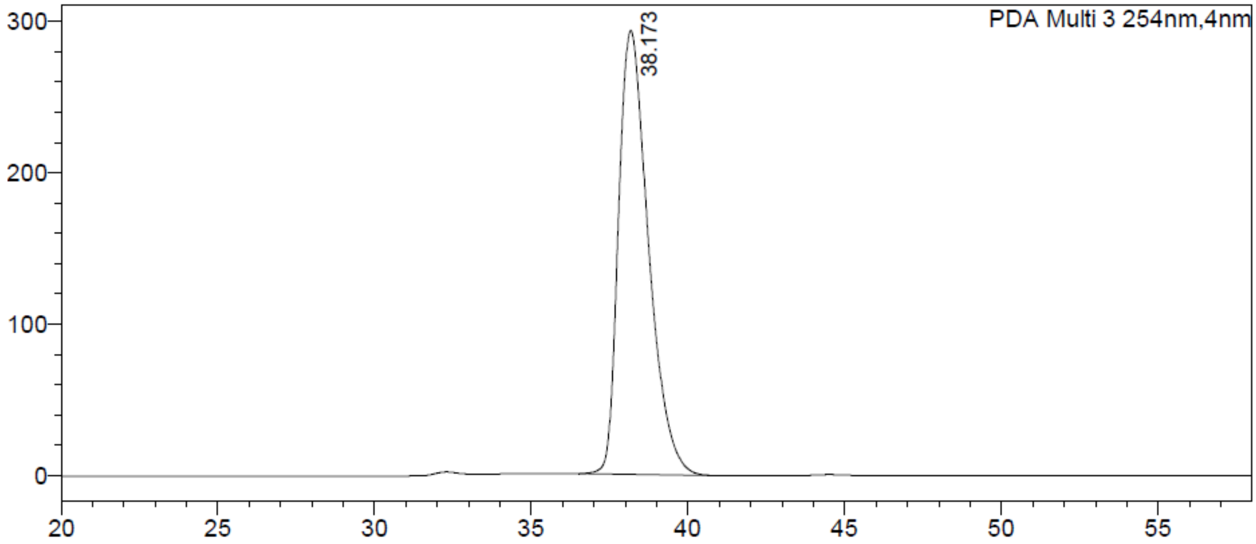

Supplement: Supplementary file 1 [file CC-053-C6CC10178J-s001.pdf]
